# Enzymatic Activity Profiling Using an Ultrasensitive Array of Chemiluminescent Probes for Bacterial Classification and Characterization

**DOI:** 10.1021/jacs.3c11790

**Published:** 2024-02-16

**Authors:** Omri Shelef, Tal Kopp, Rozan Tannous, Maxence Arutkin, Moriah Jospe-Kaufman, Shlomi Reuveni, Doron Shabat, Micha Fridman

**Affiliations:** School of Chemistry, Raymond & Beverly Sackler Faculty of Exact Sciences, Tel Aviv University, Tel Aviv 6997801, Israel

## Abstract

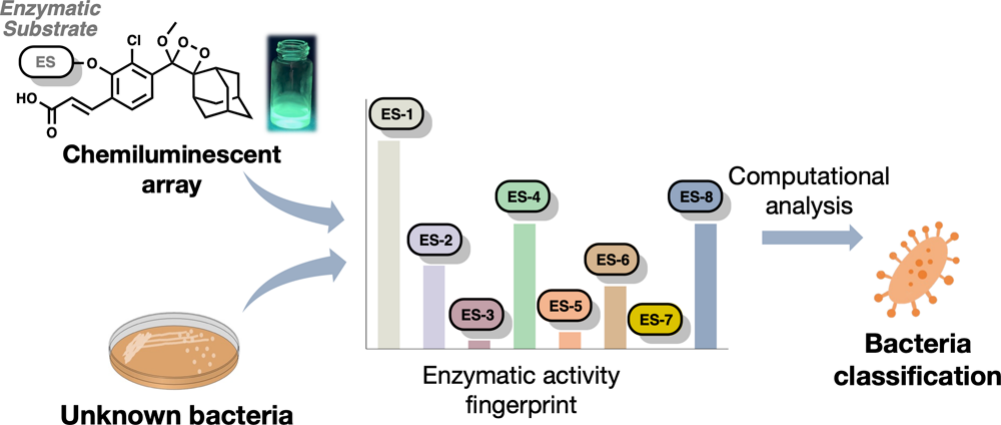

Identification and characterization of bacterial species in clinical and industrial settings necessitate the use of diverse, labor-intensive, and time-consuming protocols as well as the utilization of expensive and high-maintenance equipment. Furthermore, while cutting-edge identification technologies such as mass spectrometry and PCR are highly effective in identifying bacterial pathogens, they fall short in providing additional information for identifying bacteria not present in the databases upon which these methods rely. In response to these challenges, we present a robust and general approach to bacterial identification based on their unique enzymatic activity profiles. This method delivers results within 90 min, utilizing an array of highly sensitive and enzyme-selective chemiluminescent probes. Leveraging our recently developed technology of chemiluminescent luminophores, which emit light under physiological conditions, we have crafted an array of probes designed to rapidly detect various bacterial enzymatic activities. The array includes probes for detecting resistance to the important and large class of β-lactam antibiotics. The analysis of chemiluminescent fingerprints from a diverse range of prominent bacterial pathogens unveiled distinct enzymatic activity profiles for each strain. The reported universally applicable identification procedure offers a highly sensitive and expeditious means to delineate bacterial enzymatic activity fingerprints. This opens new avenues for characterizing and identifying pathogens in research, clinical, and industrial applications.

## Introduction

Accurately identifying microorganisms and comprehensively characterizing their diverse attributes in a reproducible, rapid, and cost-effective manner presents a substantial challenge.^1^ Rapid and precise identification and characterization of pathogens are crucial to ensure accurate diagnosis, effective treatment, and the prevention of outbreaks in clinical, industrial, and agricultural settings.^2^

Clinical microbiology laboratories endeavor to attain precise bacterial identification and determine drug susceptibility profiles.^3,4^ This empowers physicians to make informed decisions swiftly regarding optimal treatment options.^5,6^ The progression of the COVID-19 pandemic has highlighted the pivotal role of research and clinical microbiology laboratories in uncovering the sources and modes of transmission of outbreaks. This essential information is paramount for effectively controlling infectious diseases and averting their widespread dissemination and recurrence.

In recent decades, the field of pathogen identification has experienced significant advancement through the introduction of innovative analytical methods reliant on modern technologies and equipment.^7^ Among these advancements, next-generation sequencing has transformed microbial diagnostics by enabling unbiased and hypothesis-free detection of a wide spectrum of microbial pathogens, both common and rare, without the necessity for prior culture growth.^8^ Another method gaining increasing prominence in clinical microbiology laboratories is mass spectrometry-based techniques, with a particular focus on matrix-assisted laser desorption/ionization time-of-flight mass spectrometry (MALDI-TOF MS).^9^ This method has demonstrated its effectiveness in identifying pathogenic bacteria, including microaerobes, anaerobes, mycobacteria, and fungi.^10^ Its extensive and robust database, coupled with exceptional performance, guarantees rapid and precise outcomes without the necessity for highly specialized laboratory personnel to oversee the procedure. Nonetheless, it is worth noting that this technology has limitations; it can solely identify isolates if the measured spectrum contains peptide mass fingerprints of the pathogen found in a given database, which can diminish its effectiveness when dealing with newly emerging pathogens.^11^ It is also important to highlight that molecular microbiology methods like 16S sequencing and MALDI-TOF do not offer insights into specific pathogenic strain properties, such as drug resistance or virulence levels.^12^ Finally, the widespread adoption of sequencing and mass spectrometry technologies faces obstacles due to their substantial instrumentation costs and infrastructure requirements. These requirements include a stable power supply, maintenance by qualified technicians, a dust-free environment, and a stable climate.^13,14^ As a result, a significant percentage of the world’s population still faces barriers to accessing these advanced technologies.

Traditional practices, such as assessing Gram stain and colony morphology, utilizing acid-fast stains, and conducting spot indole oxidase tests, continue to be widely used around the globe.^7,15^ Nonetheless, owing to the immense diversity of the microbiome and the evolving comprehension of microbiological pathogenicity, these methods frequently lack the precision needed for definitive pathogen identification.^16^ Conventional identification processes, following streaking procedures, are time-consuming, spanning from hours to days, contingent upon the type of the pathogen involved.^17^ As an illustration, diagnosing infectious diarrhea via bacterial culture and identification in stool samples usually demands 3–5 days, rendering it a complex and expensive procedure with limited applicability for point-of-care treatments.^18^

To tackle these challenges, numerous clinical microbiology laboratories have adopted FDA- or EMA-approved biochemical kits, i.e., the Analytical Profile Index (API), for the identification of bacterial pathogens.^19^ These kits entail a series of complementary tests conducted sequentially, guided by a flowchart-based identification procedure. The API identification process is initiated with Gram staining, an oxidase test, and a fermentation test. Depending on the initial findings, the appropriate API kit is chosen for the ultimate identification and categorization of the pathogen. As an example, in the API 20E kit, a plastic strip featuring 20 mini-test chambers containing dehydrated media is utilized, with specific probes employed for each mini-test.^20,21^ The metabolic activities of the introduced organisms, including processes such as carbohydrate fermentation or protein catabolism, trigger color changes during incubation. These color changes are subsequently matched with profile numbers in a commercial codebook (or online resource) to ascertain bacterial species identification. This colorimetric identification process usually necessitates an incubation period of 18–24 h, although certain tests may extend beyond this time frame.^19,21^ Another widely employed method for identifying microbial pathogens involves assessing carbon-source utilization profiles.^22,23^ In this approach, a kit featuring a 96-well plate containing diverse carbon sources is utilized. Like the API method, carbon-source profiling is time-consuming, owing to the essential incubation period for obtaining discernible results and the necessity of choosing suitable growth conditions for the unidentified bacterial pathogen within the sample.

To address the challenges of existing identification methodologies, researchers around the world, including ourselves, have developed a remarkably sensitive and expeditious method for classifying and characterizing bacterial pathogens using chemiluminescent agents.^24−27^ In contrast to fluorescence, chemiluminescence presents distinct advantages, including the absence of the need for external light irradiation. This characteristic leads to an exceptionally minimal background signal and heightened sensitivity, setting it apart from fluorescence-based and colorimetry-based assays.^28,29^

**Figure 1:**
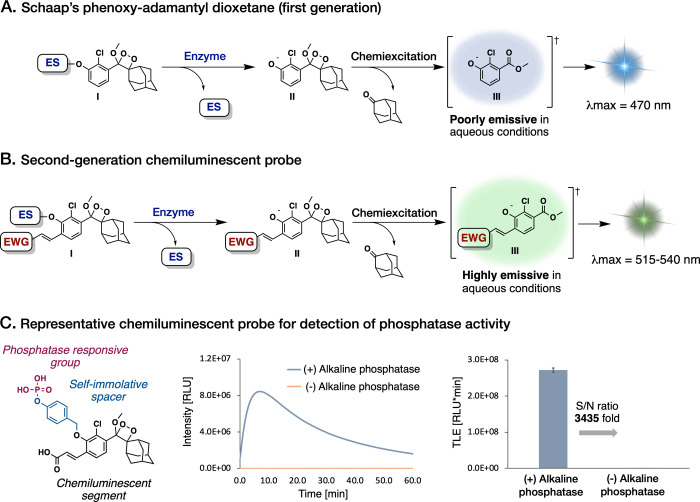
Structures and chemiexcitation pathways of (A) Schaap’s dioxetane-based probes and (B) ortho-substituted phenoxy-1,2-dioxetane probes. (C) Chemical structure, chemiluminescent kinetic profile, and total light emitted (TLE) by a phosphatase-triggered chemiluminescent probe (10 μM) with or without commercial alkaline phosphatase (1 U/mL) in PBS at pH 7.4, 0.1% DMSO, 37 °C (ES, enzymatic substrate; EWG, electron-withdrawing group; S/N, signal-to-noise).

A fascinating category of chemiluminescent compounds, referred to as triggerable phenoxy-dioxetanes, has drawn specific attention (Figure 1A).^30,31^ This feature enables the linkage of light emission to the actions of specific analytes by including a suitable phenol-protecting group as a triggering substrate. Nevertheless, despite their promise, the limited emission intensity of these dioxetane compounds in aqueous solutions has rendered them unsuitable for utilization in biological assays conducted in such settings.^32^ To overcome this hurdle, one of our groups recently achieved a significant breakthrough. It was found that incorporating an acrylate substituent at the *ortho* position of a phenoxy-adamantyl-1,2-dioxetane diminishes the water-quenching effect on the excited benzoate intermediate (Figure 1B).^33^ Furthermore, this alteration enhances the light-emission intensity of the chemiluminescent luminophore by up to 3 orders of magnitude in comparison to the original phenoxy-dioxetanes (Figure 1C). This pivotal advancement has empowered researchers to employ chemiluminescent probes in aqueous solutions without the necessity for additives, thereby broadening their scope for potential applications in biological studies.^34−43^

In this study, we present a novel and general approach for the rapid classification and identification of bacterial pathogens. Our approach harnesses the power of ultrasensitive chemiluminescent probes to create unique enzymatic activity fingerprints. These fingerprints offer a rapid and accurate tool for characterizing key bacterial enzymatic activities that can be used for the identification of diverse bacterial pathogens. Notably, the enzymatic activity fingerprints of unidentified bacteria provide crucial insights into their primary enzymatic activities. This information is pivotal for the characterization and documentation of such bacteria.

## Results and Discussion

### Chemiluminescent Probes Outperform in the Signal-to-Noise Ratio and Limit of Detection Compared to Fluorescent and Colorimetric Probes

**Figure 2:**
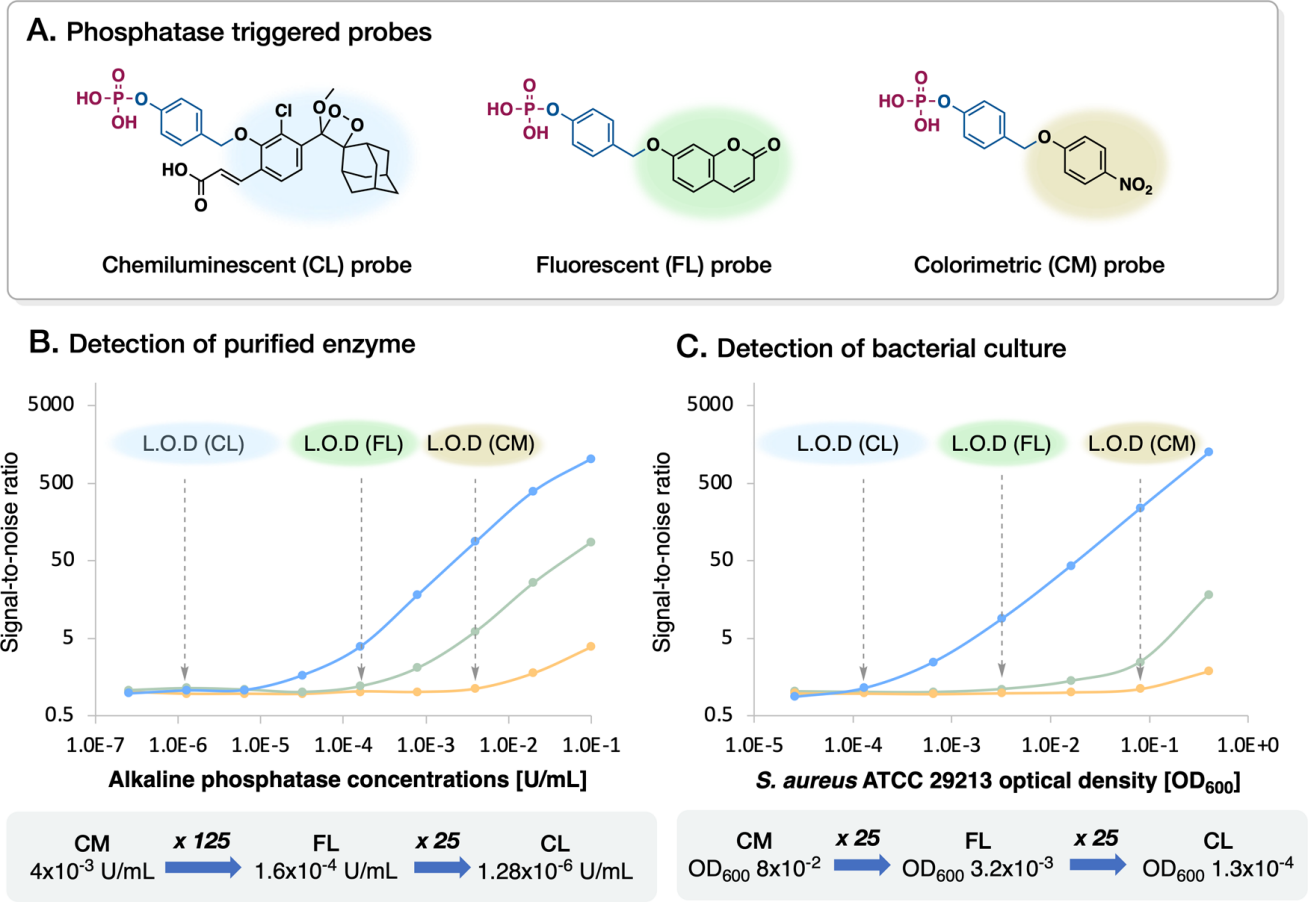
Comparison between the limits of detection of phosphatase-triggered probes. (A) Structures and the used concentrations of chemiluminescent (10 μM), fluorescent (10 μM), and colorimetric (100 μM) phosphatase probes. Limit of detection in the presence of a 5-fold serial dilution of (B) commercial alkaline phosphatase (0.1–2.56 × 10^–7^ U/mL) and (C) *S. aureus* ATCC 29213 (0.4–2.56 × 10^–5^ OD_600_) in PBS pH 7.4, 0.1% DMSO, 37 °C (CL, chemiluminescent; FL, fluorescent; CM, colorimetric; LOD, limit of detection).

To explore the advantages of developing an array of chemiluminescent probes in comparison with fluorescent or colorimetric probes, we evaluated three related probes for their ability to detect specific enzymatic activity. In each of these probes, the same phosphatase-triggering substrate initiates specific light-emitting reactions. These reactions activate one of the three optical signaling mechanisms: *ortho* acrylate-substituted phenoxy-adamantyl-1,2-dioxetane (for chemiluminescence), 7-hydroxy-coumarin (for fluorescence), or a *para*-nitrophenol dye (for colorimetry) (Figure 2A).^44^Phosphatase catalyzed cleavage of the phosphate headgroup substrate results in the formation of a phenolate functional group. This is followed by a subsequent disassembly phase characterized by a 1,6-elimination reaction, leading to the liberation of a quinone-methide molecule. Concurrently, this reaction triggers the emission of the respective optical signal, whether chemiluminescent, fluorescent, or colorimetric, as part of the reporting mechanism.

Activation of the probes with recombinant alkaline phosphatase revealed that the phosphatase limit-of-detection (LOD) value of the chemiluminescent probe was ∼25-fold lower than that of the fluorescent probe, which was in turn ∼125-fold lower than that of the colorimetric probe (Figure 2B and Figures S1–S6). We next compared the performance of the three optical probes in the presence of bacteria. Phosphatase activity was measured while incubating each of the three probes in PBS, pH 7.4, buffer containing cells of *Staphylococcus aureus* (ATCC 29213). The LOD value for the chemiluminescent probe was ∼25-fold lower than that of the fluorescent probe, which was in turn ∼25-fold lower than that of the colorimetric probe (Figure 2C and Figures S7–S9). Moreover, the signal-to-noise ratio of the chemiluminescent probe was ∼70-fold higher than that of the fluorescent probe and ∼673-fold higher than that of the colorimetric probe; hence, chemiluminescence detection based on a phenoxy-adamantyl-1,2-dioxetane unit was chosen as the detection method for the designed array of enzymatic activity sensors.

### Design and Synthesis of an Array of Chemiluminescent Probes for Sensing Catabolic Bacterial Enzymes with High Substrate Specificity

**Figure 3:**
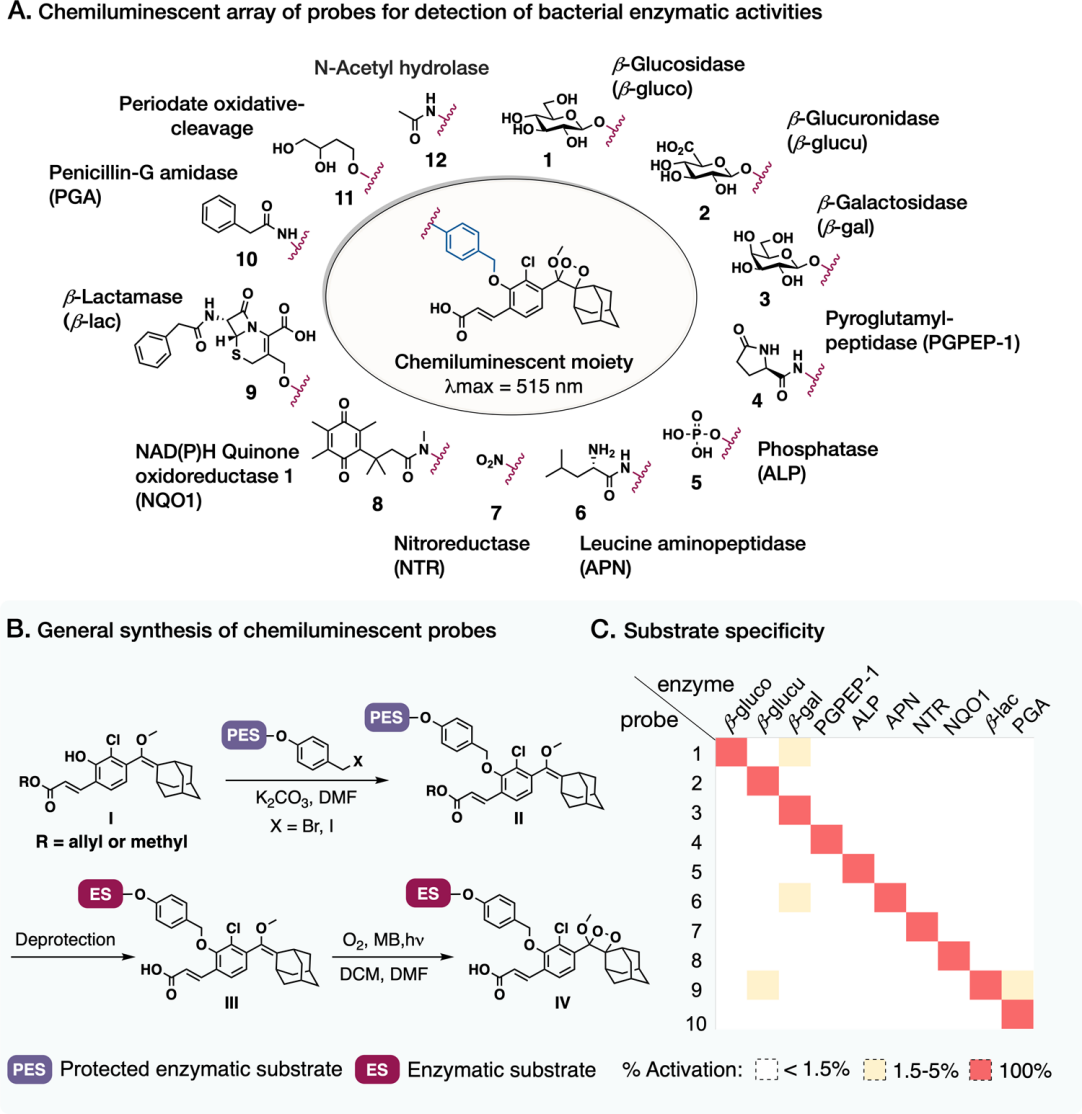
Synthesis and substrate specificity evaluation of the array of chemiluminescent probes. (A) Structures of the 12 chemiluminescent probes comprising the array. (B) General synthetic pathway for the preparation of chemiluminescent probes. (C) Substrate specificity evaluation of the 10 bacterial enzymatic activity sensing probes (10 μM) in the presence of the commercially available probe activating enzymes (β-glucosidase (10 U/mL), β-glucuronidase (1 U/mL), β-galactosidase (1 U/mL), pyroglutamyl-peptidase I (0.05 mg/mL), alkaline phosphatase (1 U/mL), aminopeptidase-M (1 U/mL), nitroreductase (1 mg/mL, 100 μM NADH), NAD(P)H quinone oxidoreductase (0.8 mg/mL, 100 μM NADH), β-lactamase (2 U/mL), and penicillin-G amidase (1 U/mL)).

Taking advantage of the chemiluminescent phosphatase probe’s heightened sensitivity, we constructed an array composed of 12 chemiluminescent probes (depicted in Figure 3A, full syntheses are presented in Schemes S1–S10). This array consists of 10 distinct triggers, each representing a substrate of a different prevalent bacterial enzymatic activity.^45−47^ We also included two control probes: an *N*-acetyl hydrolase probe for nonspecific *N*-deacetylase activity detection and a vicinal-diol oxidative-cleavage probe commonly activated by periodate. The latter oxidative-cleavage probe is unresponsive to bacterial activation and serves as a negative control.

Seven of the 12 chemiluminescent probes were designed especially for this study (probes 1, 2, 4, 9, 10, 11, and 12); β-galactosidase probe 3 is the water-soluble carboxylic acid variant of the acrylonitrile or methyl ester β-galactosidase probes previously described.^33^ Phosphatase probe 5 is an analogue that contains a self-immolative spacer and an ortho-chloride with improved sensitivity compared to the previously published version of this chemiluminescent probe.^33^ Probe 6 for leucine aminopeptidase is the water-soluble carboxylic acid version of previously reported amide and methyl ester versions of this probe.^48,49^ Nitroreductase probe 7 and NAD(P)H quinone oxidoreductase 1-activated probe 8 were previously reported.^50,51^

We proceeded to investigate the chemiluminescent probes’ specificity as substrates for the target enzymes, assessing them across a panel of recombinant enzymes corresponding to the 10 sensed activities (depicted in Figure 3C and Figures S10–S25). The probes within the array exhibited favorable selectivity as substrates for their designated enzymes with a modest background for some of the probes (Figure 3C). Finally, to assess the stability of the probes within the array, we examined their behavior under assay conditions (PBS 100 mM, pH 7.4, 0.1% DMSO, 37 °C, 60 min). The probes demonstrated satisfactory stability, with the β-lactamase probe exhibiting the least stability (∼1% decomposition) and the β-galactosidase probe showcasing the highest stability, with a decomposition of less than 0.00001%. Additionally, DMSO stock solutions of the probes stored at −20 °C remained unchanged for over a year (Figure S26).

### Array of Chemiluminescent Probes Yields Distinct Enzymatic Activity Profiles, Enabling Differentiation between Bacterial Species and Strains within a Species

Employing an array of 12 chemiluminescent probes, we characterized enzymatic activity profiles across a panel encompassing 29 strains representing nine Gram-positive and seven Gram-negative species (Table S1). This panel included prominent bacterial pathogens and representative strains of the *ESKAPE* group.^52^

Of note, the presence or absence, expression level, and intracellular or extracellular localization of individual enzymes in bacteria are all determined by a complex interplay of factors, including the species and strain, growth conditions, and even cell cycle stage. These factors collectively shape the overall enzymatic activity profile, measured by the chemiluminescent array. Moreover, the chemiluminescent probes are incubated with intact cells, meaning that the permeability or exclusion of probes from the intracellular environment plays a crucial role in shaping the measured enzymatic activity fingerprint.^53−55^ The negatively charged chemiluminescent unit (carboxylic acid) and certain triggers (i.e., phosphate or glucuronic acid) can hinder or even completely abrogate probe membrane permeability or uptake depending on the specific bacterial species or strain. Consequently, a significant portion of the signal obtained using the current probes likely originates from the cell surface and secreted enzymes, rather than those located within the cells of the bacteria in the panel.

**Figure 4:**
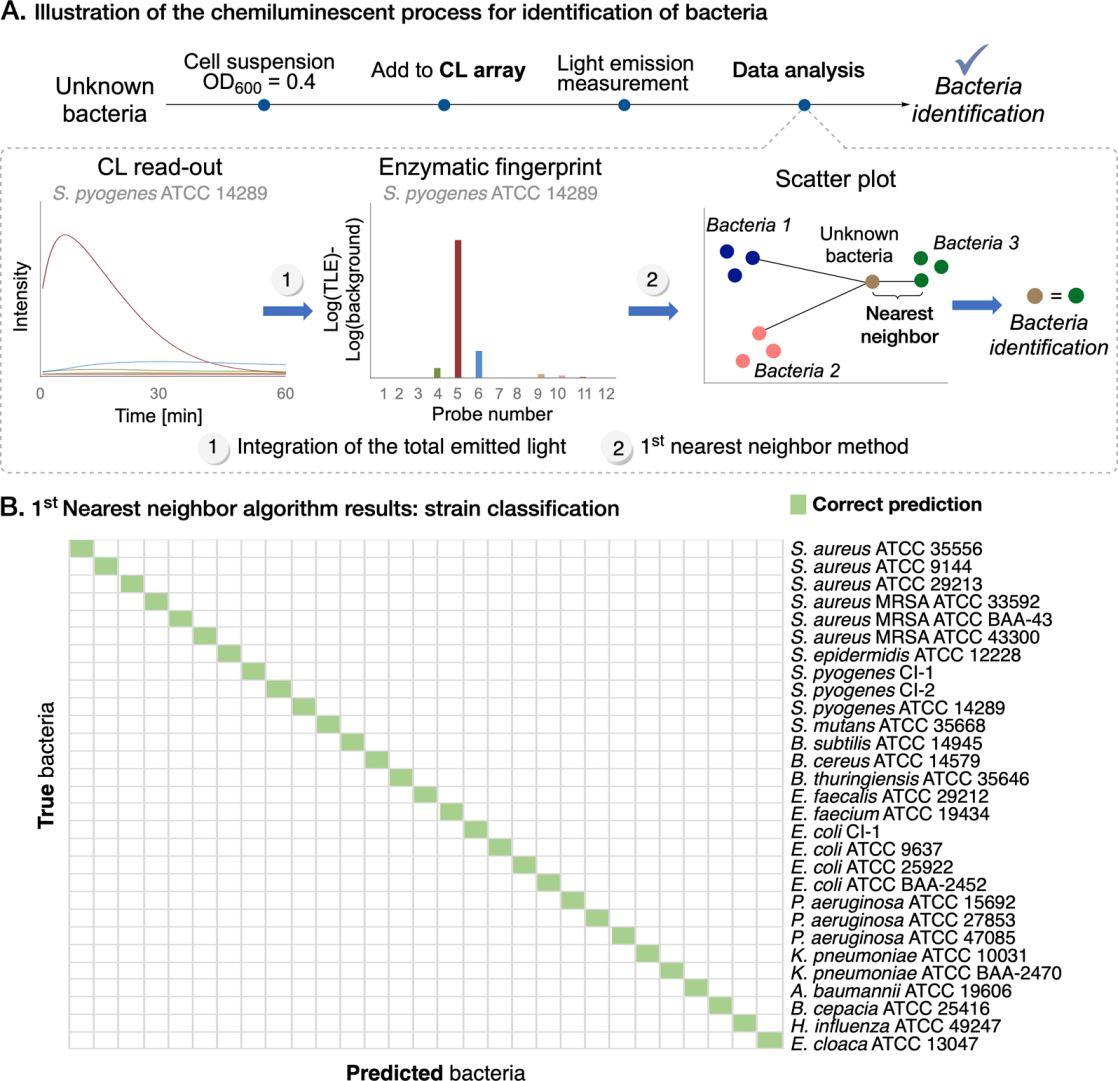
Profiling of bacterial enzymatic activities with chemiluminescent probes. (A) Schematic representation depicting the bacterial identification process, exemplified by the chemiluminescent enzymatic profile of *Streptococcus pyogenes* ATCC 14289. (B) Application of the 1-N-N method to analyze the database, successfully distinguishing between all 29 strains in the panel (TLE, total light emission; CI, clinical isolate).

The streamlined process, taking approximately 1.5 h to complete, is outlined in Figure 4A. This procedure consistently yielded reproducible enzymatic activity fingerprints for each strain, culminating in a comprehensive database comprising of 12-dimensional vectors that encapsulate the total emitted light from each probe over the course of the experiment. We assessed the logarithm of the ratio between the total emitted light and the background light, which we have identified as a background-subtracted value for enzymatic activity. Each strain displayed a distinct enzymatic activity fingerprint based on this metric, underscoring the array’s potential for bacterial species classification and even strain differentiation. Complete light emission profiles of the bacteria are presented in Figures S27–S30.

For an initial assessment of the differentiation among the bacterial species in the panel, principal component analysis (PCA) was conducted, resulting in effective clustering among all bacterial species in the panel (Figures S31 and S32). Subsequently, a quantitative analysis of the resulting database was carried out using the first nearest-neighbor method (1-N-N) based on enzymatic activity log-ratios within the data space (illustrated in Figure 4A).^56^ This proposed methodology provides a direct and efficient means for bacterial identification through examination of chemiluminescent probe data. Application of the 1-N-N analysis to the 12-probe enzymatic activity fingerprint revealed distinct differentiation among all 29 diverse bacterial strains within our panel (see Figure 4B).

We explored the impact of the assay duration on species identification accuracy. A 15 and 30 min chemiluminescence (CL) measurement yielded an accuracy of 89.6% for both. Extending the measurement to 45 and 60 min improved accuracy to 93.1 and 100%, respectively. To assess if comparable accuracy could be achieved with fewer probes, we analyzed the variance in the response of each probe across all bacterial strains in our panel (Figure S33). Intriguingly, the selection of the six most variable probes (5, 4, 2, 7, 3, and 6) proved sufficient to differentiate all 29 diverse bacterial strains in the tested panel with an accuracy of 89.6%.

We subsequently evaluated the performance of the chemiluminescent array-based process against the commercial API method, employing two relevant kits: the API20E for *E. coli* ATCC 25922, *E. coli* ATCC 9637, and *E. coli* ATCC BAA-2452 and the API Staph 20 for *S. aureus* ATCC 29213. The API method yielded identification results similar to those of our method. However, it necessitated distinct API kits for each bacterial species, and the identification was limited to the species level. Moreover, the API-based assay required 24 h to complete. In contrast, the chemiluminescent array-based method allows for universal strain-level identification within a 90 min time frame (Figures S34–S36).

To investigate the utility of the chemiluminescent array in characterizing the enzymatic activity fingerprint of samples containing more than one species, we measured the chemiluminescent fingerprint of mixed samples featuring varying ratios of two bacterial species. We chose to focus on mixtures of *E. coli* and *S. aureus*, a coinfection frequently observed in the blood of COVID-19 patients.^57^ A previous study reported a microbiological investigations on 8649 (17.7%) out of 48,902 COVID-19 patients, with clinically significant results for 1107 patients. The subsequent analysis revealed *E. coli* and *S. aureus* as the most common causative agents of bloodstream infections in hospitalized COVID-19 patients. The chemiluminescent measurements of samples with varying bacterial ratios of the two bacteria aimed to determine whether the resulting fingerprints accurately reflected the combined enzymatic activity of both bacteria or whether they revealed a novel fingerprint unrelated to either individual species.

**Figure 5:**
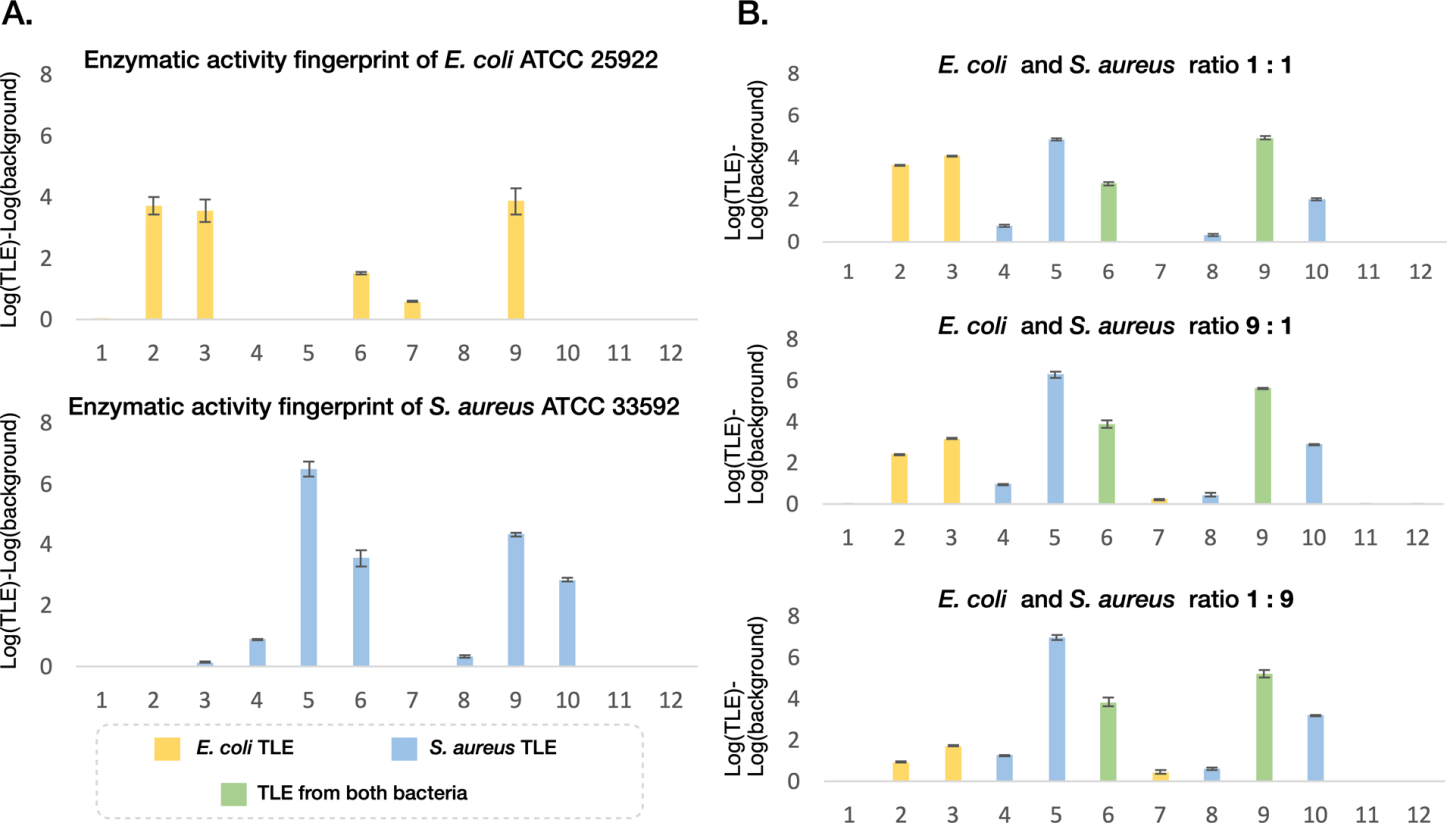
Evaluation of the enzymatic activity fingerprint of bacterial mixtures. (A) Log total light emission profiles of the chemiluminescent array of *E. coli* ATCC 25922 (top) and *S. aureus* MRSA ATCC 33592 (bottom). (B) Log total light emission fingerprints of the chemiluminescent array of *E. coli* ATCC 25922 and *S. aureus* MRSA ATCC 33592 mixtures 1:1, 9:1, and 1:9, respectively.

Notably, the fingerprints exhibited signals from probes activated by both species as well as probes predominantly activated by either *E. coli* or *S. aureus* (Figure 5). The intensity of the chemiluminescent signal from probes activated by the characteristic dominant activity of each species nonlinearly intensified with the respective ratio of each bacterium in the mixture. These findings underscore the efficacy of the chemiluminescent array method for characterizing the enzymatic activity fingerprints of samples containing more than one bacterial species. These findings underscore the versatility and potential of the chemiluminescent array of probes in studying and characterizing more complex microorganism systems, such as entire microbiomes by exploring their distinctive enzymatic activity fingerprints.

**Figure 6:**
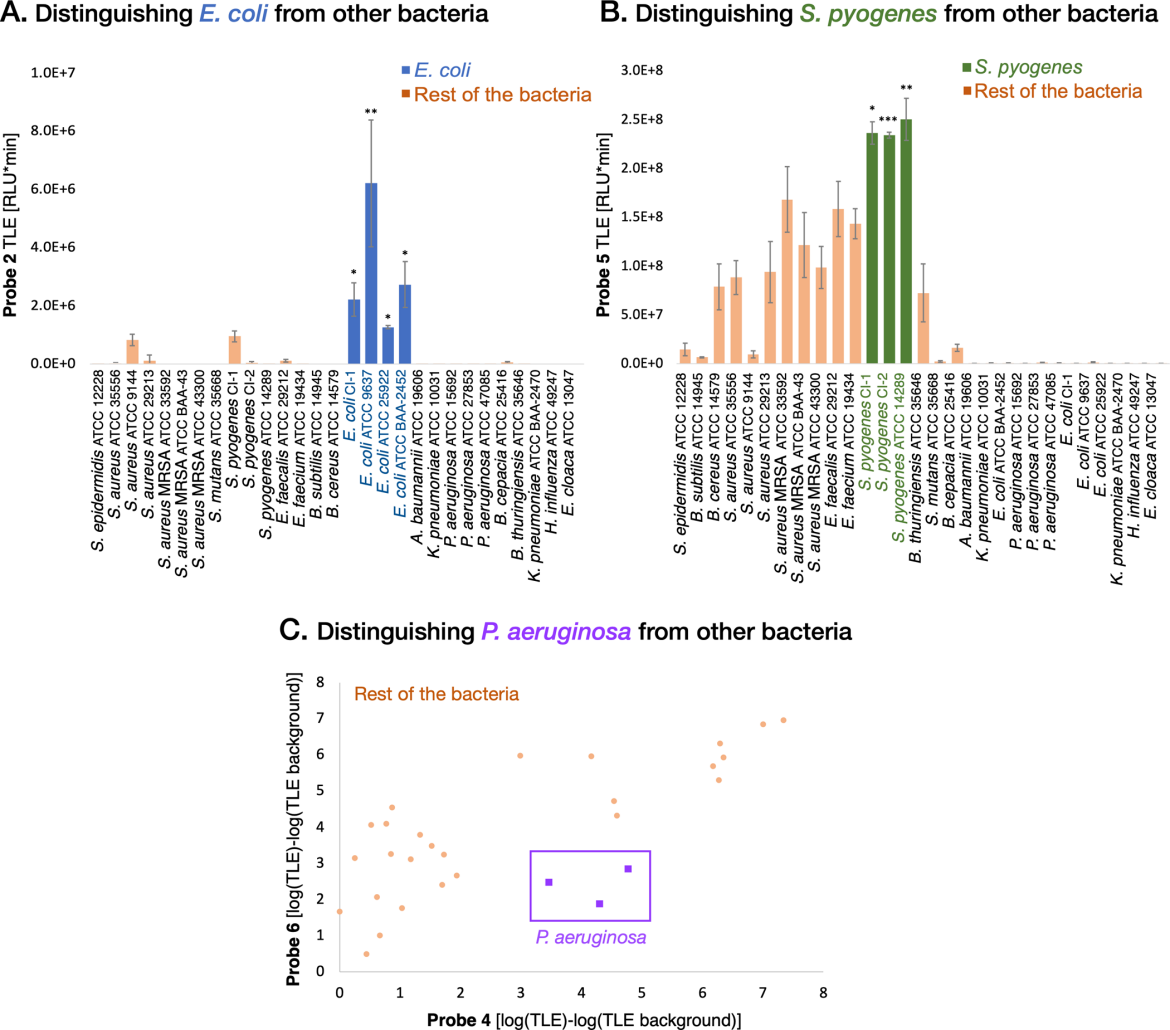
Several species in the panel can be distinguished based on increased activation of one or two chemiluminescent probes in the array. (A) TLE by the β-glucuronidase probe 2; (B) TLE by the phosphatase probe 5; and (C) scatter plot of the TLE by the pyroglutamyl peptidase probe 4 and leucine aminopeptidase probe 6 from the 29 strains of bacteria. The reported results are the mean values derived from a minimum of three independent experiments, each performed in triplicate.

Through comparative analyses of array TLEs across the various bacteria in the panel, several unexpected discoveries emerged. Notably, when examining the mean β-glucuronidase activity across all *E. coli* strains in the panel, a significantly higher value was observed compared to those of other bacteria (as demonstrated in Figure 6A and Figure S37). This elevated activity facilitated the differentiation of *E. coli* strains within the panel based on β-glucuronidase activity alone. This is in agreement with prior reports that designate β-glucuronidase as a marker for *E. coli* presence.^46^ Harking back to the mid-1970s, Kilian and Bulow scrutinized clinical *E. coli* isolates, discovering that around 97% exhibit β-glucuronidase production, while the majority of other coliform bacteria do not.^58^ A total of 460 humans, 105 cows, and 55 horses with *E. coli* isolates were tested. The results showed 95.5% β-glucuronidase-positive isolates after 24 h and 99.5% positive after 28 h of incubation.

Similarly, the TLE of phosphatase probe 5 was significantly higher for the *S. pyogenes* strains in the panel than for all other strains (Figure 6B and Figure S38). *S. pyogenes*, also known as group A *Streptococcus*, is a group of Gram-positive bacteria that can be carried in human throats or skin, and it is responsible for more than 500,000 deaths worldwide annually.^59^ The conventional tests for the identification of *S. pyogenes* in clinical samples involve blood agar plates that are screened for the presence of β-hemolytic colonies.^60^ The typical appearance of *S. pyogenes* colonies after 24 h of incubation at 35–37 °C is dome-shaped with a smooth or moist surface and clear margins. It is noteworthy that automated bacterial identification by MALDI-TOF has limitations in distinguishing among closely related taxa within group A streptococci, to which *S. pyogenes* belongs.^61^ Several point-of-care tests for the detection of *S. pyogenes* in throat swabs using rapid automated PCR technology have received FDA clearance to date, yet the use of PCR for diagnosing streptococcal throat infections remains low.^62^ The high activity detected by chemiluminescent probe 5 suggests that testing for high levels of phosphatase could potentially provide an affordable substitute for the detection of *S. pyogenes*.

*Pseudomonas aeruginosa* is yet another bacterium that can be identified using less than the 12 probes in the array. Prior studies have indicated that *P. aeruginosa* exhibits pyroglutamyl aminopeptidase activity.^63^ Interestingly, using solely the pyroglutamyl aminopeptidase probe yielded a high success rate (∼90%) in distinguishing the three *P. aeruginosa* strains from all of the other bacteria within our panel. Moreover, a dual-probe strategy involving pyroglutamyl aminopeptidase and leucine aminopeptidase achieved ∼97% identification accuracy (as illustrated in Figure 6C and Figure S39).

Next, we investigated the capability of the chemiluminescence-based enzymatic activity fingerprinting method to classify strains of bacteria that were not part of the database, even though they belong to species already represented (Table S1). Specifically, we focused on three strains: *S. aureus* ATCC 33591, an *E. coli* clinical isolate, and *P. aeruginosa*. To assess their similarity to known strains, we adopted a statistical approach, which involved representing each strain in the original data set as a Gaussian cloud within the 12-dimensional space corresponding to log-ratios of the TLE from each probe. This representation facilitated the ranking of the similarity of new bacteria to existing strains in our data set and enabled the statistical testing of the hypothesis that they are identical.

**Figure 7:**
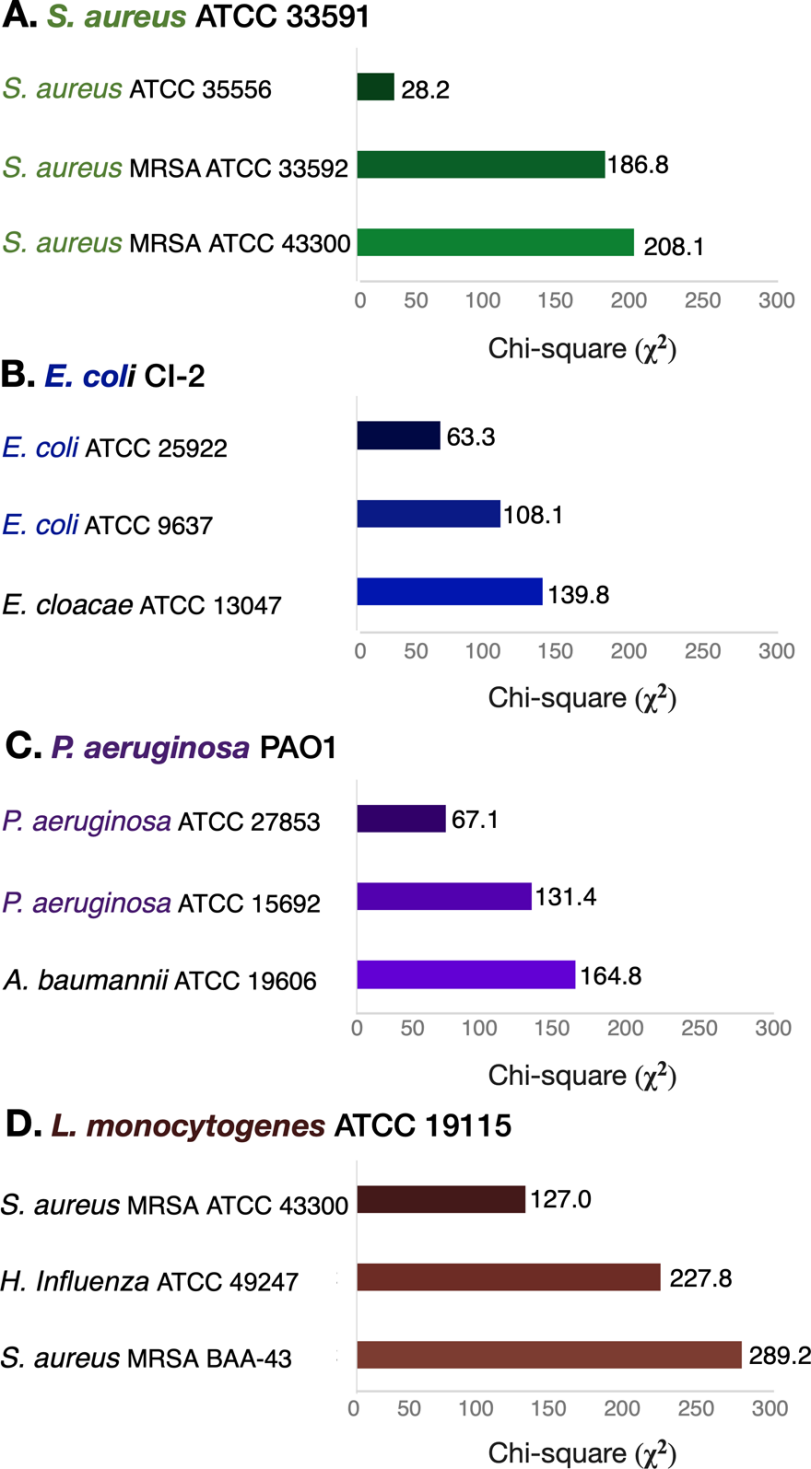
Top three most similar strains for four unknown bacteria that were not included in the original panel: (A) *S. aureus* ATCC 33591 classified with the highest resemblance as *S. aureus*, (B) *E. coli* clinical isolate-2 (CI-2) classified with the highest resemblance as *E. coli*, and (C) *P. aeruginosa* PAO1 classified with the highest resemblance as *P. aeruginosa*. (D) *L. monocytogenes* ATCC 19115 belonging to a species not represented in the database was classified with the highest resemblance as *S. aureus* MRSA 43300. The enzymatic activity fingerprints of the four unknowns are presented in Figure S40.

Using the χ^2^ metric, we conducted a thorough comparison of the enzymatic activity fingerprints of the selected strains with those in our panel. The results of this analysis are summarized in Figure 7. Notably, the statistical findings consistently demonstrated that all three examined bacteria exhibit the nearest resemblance to known panel bacteria from the same species. It is important to emphasize that in all cases, the *p*-values resulting from the χ^2^ test indicated a rejection of the hypothesis that the unknown bacteria were present in the database. Instead, our analysis consistently highlighted the closest strains from the same bacterial families as the most resembling. The tested *S. aureus* ATCC 33591 showed the highest resemblance to *S. aureus* ATCC 35556, while the *E. coli* clinical isolate closely resembled *E. coli* ATCC 25922. Similarly, the enzymatic profile of the *P. aeruginosa* PAO1 demonstrated a resemblance to *P. aeruginosa* ATCC 27853. These results demonstrate the effectiveness of the chemiluminescence-based enzymatic activity fingerprinting method in categorizing and identifying unknown bacteria.

Finally, we further expanded the scope of our investigation by introducing a bacterium strain belonging to a species not originally represented in the database: the Gram-positive pathogen *Listeria monocytogenes* ATCC 19115. This strain, like all others tested previously, displayed a distinct enzymatic fingerprint. The χ^2^ metric score indicated that its closest match within the database was the Gram-positive *S. aureus* ATCC 43300 (Figure 7D). Of note, the proximity between the enzymatic profiles of *L. monocytogenes* ATCC 19115 and *S. aureus* ATCC 43300, as measured by the χ^2^ metric score, was not as high as the proximity observed between the three unknown strains (belonging to species already represented in the database) and their closest counterparts in the database. Notably, both *L. monocytogenes* and *S. aureus* are Gram-positive pathogens. This illustrates that while unknowns not represented in the panel cannot be directly identified, their enzymatic activity fingerprint offers valuable insights that, in turn, can be utilized to identify close resemblances to other bacteria.

## Conclusions

In summary, our study introduces a robust and cost-effective method for the identification and characterization of bacteria, addressing the challenges posed by labor-intensive protocols and expensive and difficult-to-maintain equipment. This approach holds promise for widespread adoption in microbiology laboratories worldwide. The development of a 12-probe array of highly sensitive and enzyme-selective chemiluminescent probes enables the rapid acquisition of bacteria’s enzymatic activity fingerprints in 90 min. In contrast to methods confined to database comparisons, when encountering bacteria or samples containing multiple bacteria not represented in the enzymatic fingerprint database, the enzymatic activity profile of the unknown can still yield valuable information. This information sheds light on key enzymatic activity similarities found in other species. By expediting bacterial identification and characterization while offering essential information about their primary enzymatic activities, our method can also suggest potential new approaches for combination therapy using enzyme inhibitors. In summary, the method presented represents a significant advancement in rapid and highly sensitive diagnosis, offering potential applications in fundamental research, clinical settings, and industry.

## Supporting Information Available

General methods: synthetic procedures and characterization of the new chemiluminescent probes in the array (1–6, 9–12), alongside the colorimetric and fluorescent probes for alkaline phosphatase. Biological evaluation: general information and procedures, list of bacterial strains, limit-of-detection experiments procedure, substrate specificity experiments, procedure for the chemiluminescent measurement of bacterial enzymatic activity fingerprints. Computational methods: computational general methods, data acquisition and processing, 1-N-N analysis, details of the chi-squared (χ^2^) resemblance ranking and test. Supplementary figures: NMR spectra; HPLC spectra of key compounds (PDF)
