# Supplementary material for: Enzymatic Activity Profiling Using an Ultrasensitive Array of Chemiluminescent Probes for Bacterial Classification and Characterization
Source: J Am Chem Soc. 2024 Feb 16;146(8):5263–73. doi: 10.1021/jacs.3c11790 (PMC10910560; doi:10.1021/jacs.3c11790)
Supplement: Supplementary file 1 — ja3c11790_si_001.pdf [file ja3c11790_si_001.pdf]

Supporting Information

Enzymatic Activity Profiling Using an Ultra-Sensitive Array of Chemiluminescent Probes for Bacterial Classification and Characterization

Omri Shelef,[a†] Tal Kopp,[a†] Rozan Tannous,[a†] Maxence Artukin,[a] Moriah Jospe-Kaufman,[a] Shlomi Reuveni,[a*] Doron Shabat [a*] and Micha Fridman [a*].

[a]School of Chemistry, Raymond and Beverly Sackler Faculty of Exact Sciences, Tel-Aviv University, Tel-Aviv 69978 Israel.

[†]These authors contributed equally to this study.

*Correspondence to: Shlomi Reuveni, Email: shlomire@tauex.tau.ac.il; Doron Shabat, Email: chdoron@tauex.tau.ac.il; Micha Fridman, Email: mfridman@tauex.tau.ac.il.

Table of Contents

SYNTHETIC PROCEDURES AND CHARACTERIZATION OF THE CHEMILUMINESCENT PROBES **5**

β-Glucosidase probe 1 5

β-Glucuronidase probe 2 7

β-Galactosidase probe 3 9

Pyroglutamyl aminopeptidase probe 4 9

Phosphatase probe 5 11

Leucine aminopeptidase probe 6 13

β-Lactamase probe 9 14

Penicillin-G amidase probe 10 15

Periodate oxidative-cleavage probe 11 17

N-Acetyl hydrolase probe 12 18

Fluorescent phosphatase probe - Compound 17 19

Colorimetric phosphatase probe - Compound 19 20

BIOLOGICAL EVALUATION: GENERAL INFORMATION AND PROCEDURES **22**

List of bacteria strains 22

L.O.D experiments procedure 23

Enzymatic substrate specificity experiments 23

Chemiluminescent measurements of bacterial enzymatic activity fingerprints 23

Chemiluminescent measurements of enzymatic activity fingerprint in bacterial mixtures 23

COMPUTATIONAL METHODS **24**

General methods 24

Data acquisition and processing 24

1-N-N Analysis 25

Details of the chi-squared ($\chi 2$) resemblance ranking and test. 25

SUPPLEMENTARY FIGURES **27**

NMR SPECTRA 50

HPLC SPECTRA OF KEY COMPOUNDS 79

REFERENCES 99

General methods

All reactions requiring anhydrous conditions were performed under an Argon atmosphere. All reactions were carried out at room temperature unless stated otherwise. Chemicals and solvents were either A.R. grade or purified by standard techniques. Thin-layer chromatography (TLC): silica gel plates Merck 60 F254: compounds were visualized by irradiation with UV light. Column chromatography (FC): silica gel Merck 60 (particle size 0.040-0.063 mm), eluent given in parentheses. Reverse-phase high-pressure liquid chromatography (RP-HPLC): C18 5u, 250x4.6mm, eluent given in parentheses. Preparative RP-HPLC: C18 5u, 250x21mm, eluent given in parentheses. ^{1}H-NMR spectra were measured using Bruker Avance operated at 400MHz. ^{13}C-NMR spectra were measured using Bruker Avance operated at 100 MHz. Chemical shifts were reported in ppm on the δ scale relative to a residual solvent ($CDCl_3$: δ = 7.26 for ^{1}H-NMR and 77.16 for ^{13}C-NMR, DMSO-d_6: δ = 2.50 for ^{1}H-NMR and 39.52 for ^{13}C-NMR). Multiplicities are reported with the following abbreviations: br, broad; s, singlet; d, doublet; t, triplet; dt, doublet of triplets; dd, doublet of doublets; ddd, doublet of doublet of doublets; qd, quartet of doublets; m, multiplet; eq, equatorial; ax, axial. Coupling constants (J) are given in Hertz. Mass spectra were measured on Waters Xevo TQD. Chemiluminescence was recorded on Molecular Devices Spectramax iD3. Fluorescence was recorded on Tecan infinite 200 Pro. All chemicals, unless otherwise stated, were obtained from commercial sources. Light irradiation for photochemical reactions: LED PAR38 lamp (19W, 3000K).

Abbreviations

ACN- Acetonitrile, **Ag_2O**- Silver oxide, **$CHCl_3$**- Chloroform, **DCM**- dichloromethane, **DIPEA**- N,N-Diisopropylethylamine, **DMBA**- Dimethyl barbituric acid, **DMF**- N,N' –Dimethylformamide, **EEDQ**- N-Ethoxycarbonyl-2-ethoxy-1,2-dihydroquinoline, **EtOAc**- Ethyl acetate, **Hex**- Hexanes, **iPrOH**- Isopropyl alcohol, **K_2CO_3**- Potassium carbonate, **LiOH**- Lithium hydroxide **MB**- Methylene blue **MeOH**- Methanol, **NH_4Cl**- ammonium chloride, **$NaHCO_3$**- Sodium bicarbonate, **$Na_2S_2O_3$**- Sodium Thiosulfate, **Na_2SO_4**- Sodium Sulfate, **$NaBH_4$**- Sodium borohydride, **NaI**- Sodium Iodide, **PBr_3**- Phosphorus tribromide, **$Pd(PPh_3)_4$**- Tetrakis(triphenylphosphine)palladium, **THF**- Tetrahydrofuran, **TMS-Cl** - Trimethylsilyl chloride.

Synthetic procedures and characterization of the chemiluminescent probes

Synthesis of **Nitroreductase probe 7** and **NAD(P)H Quinone oxidoreductase-1 probe 8** were synthesized according to known procedures[1, 2].

β-Glucosidase probe 1

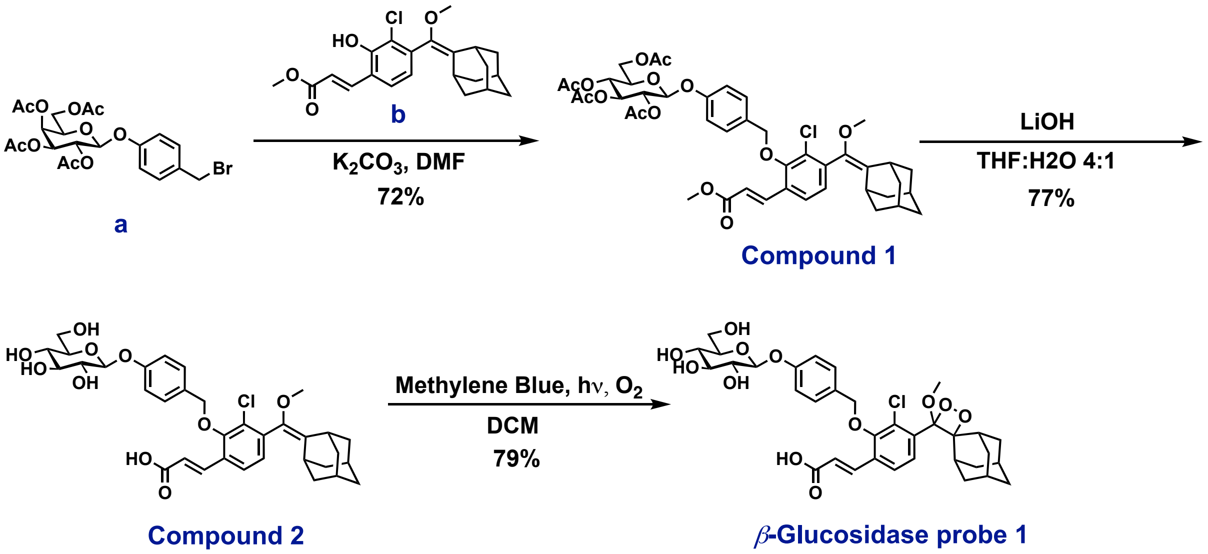

Scheme S1. Synthesis of ***β*-glucosidase probe 1**.

Compound 1

Phenol enol ether **b**[3] (40 mg, 0.10 mmol, 1.2 eq.) and K_2CO_3 (21 mg, 0.15 mmol, 1.5 eq.) were dissolved in DMF (1 mL). The solution was stirred for 5 minutes before the addition of compound **a**[4] (44 mg, 0.08 mmol, 1 eq.). The reaction mixture was stirred at room temperature and monitored by TLC (EtOAc/Hex mixture). Upon completion, the reaction mixture was diluted with EtOAc (100 mL) and washed with 0.1M HCl (50 mL) and brine (50 mL). The organic layer was separated, dried over Na_2SO_4, and evaporated under reduced pressure. The crude product was purified by column chromatography on silica gel (EtOAc: Hex 6:4). Compound **1** was obtained as a white solid (61 mg, 72% yield). MS (ES-): m/z calc. for $C_{43}H_{49}ClO_{14}$: 824.28; found: 847.7 $[M+Na]^+$. ^1H NMR (400 MHz, $CDCl_3$) δ 7.94 (d, *J* = 16.2 Hz, 1H), 7.46 (d, *J* = 8.3 Hz, 3H), 7.09 (d, *J* = 8.0 Hz, 1H), 7.03 (d, *J* = 8.6 Hz, 2H), 6.47 (d, *J* = 16.2 Hz, 1H), 5.33 – 5.28 (m, 1H), 5.23 – 5.14 (m, *J* = 9.8 Hz, 1H), 5.14 – 5.07 (m, 1H), 4.96 (d, *J* = 3.5 Hz, 2H), 4.37 – 4.27 (m, 1H), 4.19 (dd, *J* = 12.3, 2.4 Hz, 1H), 3.89 (ddd, *J* = 10.0, 5.2, 2.4 Hz, 1H), 3.82 (s, 3H), 3.34 (s, 3H), 3.30 (d, *J* = 4.3 Hz, 1H), 2.11 (s, 3H), 2.09 (s, 4H), 2.07 (s, 3H), 2.06 (s, 3H), 2.01 – 1.67 (m, 13H).^{13}C NMR (100 MHz, $CDCl_3$) δ 170.6, 170.3, 169.4, 169.3, 167.1, 157.1, 153.7, 139.4, 138.8, 138.2, 132.5, 131.1, 130.4, 127.8, 125.0, 119.9, 117.1, 99.2, 75.6, 72.7, 72.1, 71.2, 68.3, 61.9, 57.3, 51.8, 39.2, 39.0, 38.6, 37.0, 32.9, 29.7, 28.3, 28.2, 20.7, 20.6.

Compound 2

Compound **1** (61 mg, 0.07 mmol, 1 eq.) and LiOH (18 mg, 0.7 mmol, 10 eq.) were dissolved in 1 mL solution of THF: H_2O (4:1). The reaction mixture was stirred at 40 °C and monitored by RP-HPLC. Upon completion, the solvent was concentrated under reduced pressure, and the product was purified by preparative RP-HPLC (mobile phase: acetonitrile in H_2O containing 0.1% TFA; gradient from 70 to 100%; flow rate: 20 mL/min; retention time: 6 min). Compound **2** was obtained as a white solid (50 mg, 77% yield). MS (ES+): m/z calc. for $C_{34}H_{39}ClO_{10}$: 642.22; found:641.6 $[M-H]^-$ ^1H NMR (400 MHz, $CDCl_3$) δ 7.94 (d, *J* = 16.2 Hz, 1H), 7.46 (d, *J* = 8.3 Hz, 3H), 7.09 (d, *J* = 8.0 Hz, 1H), 7.03 (d, *J* = 8.6 Hz, 2H), 6.47 (d, *J* = 16.2 Hz, 1H), 5.33 – 5.28 (m, 1H), 5.23 – 5.14 (m, *J* = 9.8 Hz, 1H), 5.14 – 5.07 (m, 1H), 4.96 (d, *J* = 3.5 Hz, 2H), 4.37 – 4.27 (m, 1H), 4.19 (dd, *J* = 12.3, 2.4 Hz, 1H), 3.89 (ddd, *J* = 10.0, 5.2, 2.4 Hz, 1H), 3.82 (s, 3H), 3.34 (s, 3H), 3.30 (d, *J* = 4.3 Hz, 1H), 2.11 (s, 3H), 2.09 (s, 4H), 2.07 (s, 3H), 2.06 (s, 3H), 2.01 – 1.67 (m, 13H).^{13}C NMR (100 MHz, $CDCl_3$) δ 170.6, 170.3, 169.4, 169.3, 167.1, 157.1, 153.7, 139.4, 138.8, 138.2, 132.5, 131.1, 130.3, 127.8, 125. 1, 119.9, 117.1, 99.2, 75.6, 72.7, 72.1, 71.2, 68.3, 61.9, 57.3, 51.8, 39.2, 39.0, 38.6, 37.0, 32.9, 29.7, 28.3, 28.2, 20.7, 20.6.

***β*-Glucosidase probe 1**

Compound **2** (10 mg, 0.02 mmol) and a catalytic amount of methylene blue (~1 mg) were dissolved in 10 mL of DCM. Oxygen was bubbled through the solution while irradiating with yellow light. The reaction was monitored by RP-HPLC. Upon completion, the solvent was concentrated under reduced pressure, and the product was purified by preparative RP-HPLC (mobile phase: acetonitrile in H_2O containing 0.1% TFA; gradient from 70 to 100%; flow rate: 20 mL/min; retention time: 9 min). ***β*-glucosidase probe 1** was obtained as a white solid (8.3 mg, 79% yield). MS (ES-): m/z calc. for $C_{34}H_{39}ClO_{12}$: 674.21; found:673.6 $[M-H]^-$ ^1H NMR (400 MHz, $CDCl_3$) δ 7.61 (d, *J* = 16.1 Hz, 1H), 7.34 (d, *J* = 8.1 Hz, 1H), 7.24 (d, *J* = 8.3 Hz, 2H), 7.05 – 6.94 (m, *J* = 8.9 Hz, 3H), 6.16 (d, *J* = 16.1 Hz, 1H), 5.00 – 4.84 (m, *J* = 24.8, 6.6 Hz, 3H), 3.91 (d, *J* = 10.7 Hz, 1H), 3.86 – 3.54 (m, *J* = 21.4, 12.8, 6.7 Hz, 9H), 3.53 – 3.44 (m, *J* = 8.5 Hz, 1H), 3.30 (s, 3H), 3.24 (s, 1H), 2.05 (s, 1H), 1.97 – 1.60 (m, 12H).^{13}C NMR (100 MHz, $CDCl_3$) δ 169.1, 157.4, 153.6, 139.7, 139.4, 138.0, 132.6, 130.9, 130.1, 129.6, 127.7, 124. 8, 119.4, 116.7, 100.4, 76.2, 76.0, 73.2, 69.5, 61.3, 57.2, 39.1, 39.0, 38.6, 37.0, 32.9, 29.7, 28.3, 28.1.

β-Glucuronidase probe 2

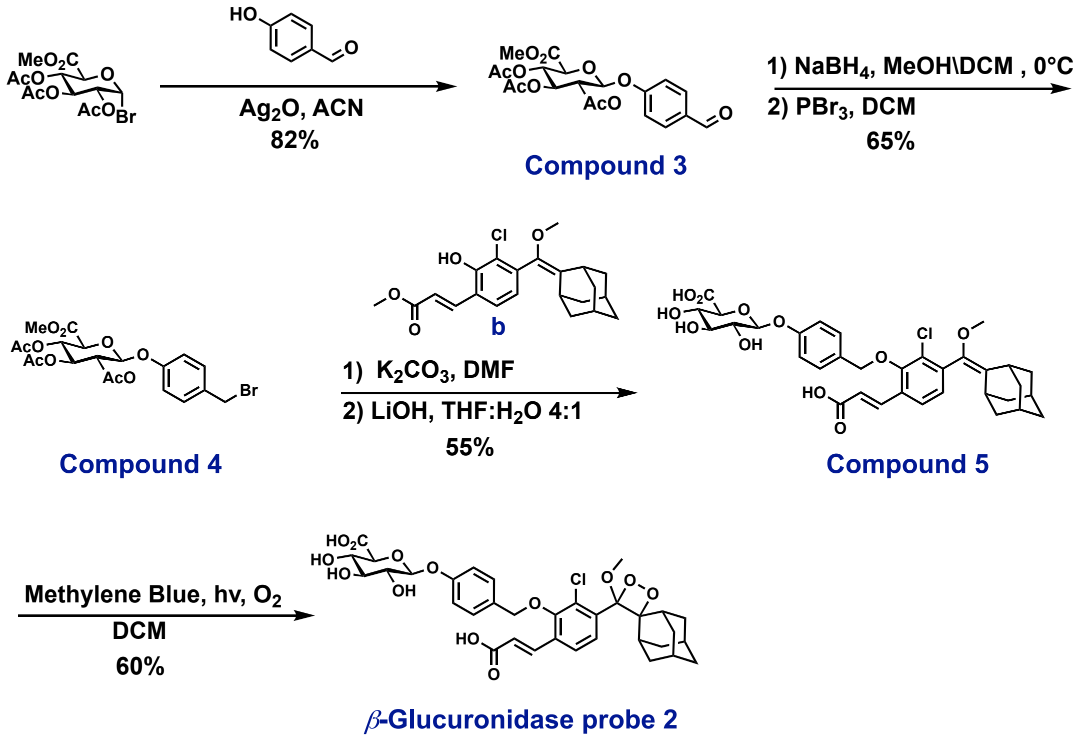

Scheme S2. Synthesis of ***β*-glucuronidase probe 2**.

Compound 3

Ag_2O (145 mg, 0.63 mmol, 2.5 eq.) was added to Acetobromo-α-D-glucuronic acid methyl ester (100 mg, 0.25 mmol, 1 eq.) and 4-Hydroxybenzaldehyde (34 mg, 0.28 mmol, 1.1 eq.) dissolved in ACN. The reaction mixture was stirred at room temperature and monitored by TLC. Upon completion, ACN was evaporated under reduced pressure and the crude product was purified by column chromatography on silica gel (EtOAc: Hex 3:7) to afford compound **3** (82 mg, 82% yield) as an off-white solid. ^{1}H NMR (400 MHz, $CDCl_3$) δ 9.89 (s, 1H), 7.83 (d, *J* = 8.8 Hz, 2H), 7.09 (d, *J* = 8.7 Hz, 2H), 5.38 – 5.32 (m, 2H), 5.30 – 5.27 (m, 2H), 4.28 – 4.21 (m, 1H), 3.74 – 3.67 (m, 4H), 2.06 – 2.00 (m, 12H). ^{13}C NMR (100 MHz, $CDCl_3$) δ 190.9, 170.2, 169.5, 169.3, 166.9, 161.2, 132.0, 117.0, 98.1, 72.8, 71.7, 71.0, 69.0, 53.2, 20.7. The ^{1}H-NMR and ^{13}C-NMR data are in agreement with the literature values.[5]

Compound 4

Compound **3** (35 mg, 0.08 mmol, 1 eq.) was dissolved in 1 mL of MeOH and DCM (1:1) and cooled to 0℃. $NaBH_4$ (3.4 mg, 0.09 mmol, 1.1 eq.) was added to the reaction mixture and the reaction was allowed to warm up to room temperature. The reaction mixture was stirred at room temperature and monitored by TLC (EtOAc: Hex 6:4). Upon completion, the reaction mixture was diluted with EtOAc (100 mL) and washed with NH_4Cl (50 mL) and brine (50 mL). The organic layer was separated, dried over Na_2SO_4, and evaporated under reduced pressure. The crude mixture was reacted without further purification. The crude was dissolved in DCM followed by dropwise addition of PBr_3 (14 µL, 0.15mmol, 1.8 eq.). The reaction was monitored by TLC (EtOAc: Hex 6:4). Upon completion, the reaction mixture was diluted with EtOAc, and washed with saturated $NaHCO_3$ followed by brine. The organic layer was separated, dried over Na_2SO_4, and filtered and the solvent was evaporated under reduced pressure, the crude product was purified by column chromatography on silica gel (EtOAc: Hex 6:4) to afford

compound **4** (70 mg, 65% yield) a yellow oil. ^{1}H NMR (400 MHz, $CDCl_3$) δ 7.32 (d, *J* = 8.0 Hz, 2H), 6.95 (d, *J* = 8.0 Hz, 2H), 5.38 – 5.24 (m, 3H), 5.15 (d, *J* = 7.1 Hz, 1H), 4.47 (s, 1H), 4.18 (d, *J* = 8.0 Hz, 1H), 3.72 (s, 3H), 2.04 (s, 9H). ^{13}C NMR (101 MHz, $CDCl_3$) δ 170.2, 169.5, 169.4, 167.0, 156.7, 133.1, 130.6, 117.4, 99.0, 72.8, 71.9, 71.2, 69.2, 53.1, 33.2, 29.8, 20.7.

Compound 5

Phenol enol ether **b** (20 mg, 0.04 mmol, 1 eq.) and K_2CO_3 (9 mg, 0.06 mmol, 1.5 eq.) were dissolved in DMF (0.5 mL). The solution was stirred for 5 minutes before compound **4** (16 mg, 0.04 mmol, 1 eq.) was added. The reaction mixture was stirred at room temperature and monitored by TLC (EtOAc: Hex 6:4). Upon completion, the reaction mixture was diluted with EtOAc (100 mL) and washed with saturated NH_4Cl (50 mL) and brine (50 mL). The organic layer was separated, dried over Na_2SO_4, and evaporated under reduced pressure. The crude mixture was reacted without further purification. The crude and LiOH (9.5 mg, 0.39 mmol, 10 eq.) were dissolved in 1 mL solution of THF: H_2O (4:1). The reaction mixture was stirred at 60 °C and monitored by RP-HPLC. Upon completion, the solvent was concentrated under reduced pressure and the product was purified by preparative RP-HPLC (mobile phase: acetonitrile in H_2O containing 0.1% TFA; gradient from 30 to 100%; flow rate: 20 mL/min; retention time: 7 min). Compound **5** was obtained as a white solid (14 mg, 55% yield). MS (ES+): m/z calc. for $C_{34}H_{37}ClO_{11}$: 656.20; found: 679.5 $[M+Na]^+$. ^{1}H NMR (400 MHz, $CDCl_3$) δ 7.56 – 7.45 (m, *J* = 16.0 Hz, 1H), 7.23 (s, 1H), 7.12 – 7.04 (m, 2H), 6.99 – 6.87 (m, *J* = 7.0 Hz, 3H), 6.08 (d, *J* = 15.9 Hz, 2H), 5.05 – 4.92 (m, 1H), 4.83 – 4.62 (m, 2H), 4.24 – 4.07 (m, 1H), 3.97 – 3.73 (m, 3H), 3.27 – 3.16 (m, 4H), 2.04 – 1.97 (m, 2H), 1.93 – 1.56 (m, 12H). ^{13}C NMR (100 MHz, $CDCl_3$) δ 172.5, 169.9, 157.4, 153.7, 140.4, 139.5, 138.3, 132.6, 131.2, 130.4, 130.0, 129.7, 127.8, 124.9, 119.1, 117.1, 100.5, 76.3, 75.6, 74.4, 72.9, 71.5, 57.3, 39.2, 39.14, 38.8, 38.7, 38.7, 37.1, 33.0, 29.8, 28.3.

***β*-Glucuronidase probe 2**

Compound **5** (10 mg, 0.02 mmol) and a catalytic amount of methylene blue (~1 mg) were dissolved in 10 mL of DCM. Oxygen was bubbled through the solution while irradiating with yellow light. The reaction was monitored by RP-HPLC. Upon completion, the solvent was concentrated under reduced pressure and the product was purified by preparative RP-HPLC (mobile phase: acetonitrile in H_2O containing 0.1% TFA; gradient from 30 to 100%; flow rate: 20 mL/min; retention time: 5 min). ***β*-glucuronidase probe 2** was obtained as a white solid (6 mg, 60% yield). MS (ES-): m/z calc. for $C_{34}H_{37}ClO_{13}$: 688.19; found: 687.3 $[M-H]^-$. MS (ES+): m/z calc. for $C_{34}H_{37}ClO_{13}$: 688.19; found: 711.5 $[M-Na]^+$. ^{1}H NMR (400 MHz, DMSO) δ 7.93 (d, *J* = 8.3 Hz, 1H), 7.79 – 7.72 (m, 2H), 7.38 (d, *J* = 7.0 Hz, 2H), 7.01 (d, *J* = 8.4 Hz, 2H), 6.64 (d, *J* = 16.1 Hz, 1H), 5.49 – 5.24 (m, 2H), 5.03 (d, *J* = 7.2 Hz, 1H), 4.86 (s, 2H), 3.94 – 3.82 (m, 2H), 3.09 (s, 3H), 2.87 (s, 1H), 2.22 (d, *J* = 11.2 Hz, 1H), 2.01 (s, 1H), 1.87 (d, *J* = 15.3 Hz, 2H), 1.73 – 1.51 (m, 8H), 1.46 (d, *J* = 11.1 Hz, 1H), 1.32 (d, *J* = 11.9 Hz, 1H), 1.24 – 1.15 (m, 2H). ^{13}C NMR (100 MHz, DMSO) δ 170.7, 167.7, 157.8, 154.2, 137.3, 134.6, 131.9, 130.8, 130.8, 129.8, 129.00 127.5, 126.7, 123.6, 116.7, 111.8, 100.5, 96.0, 76.3, 76.0, 73.5, 71.9, 50.0, 46.9, 39.7, 39.5, 36.5, 33.7, 32.2, 31.7, 31.5, 27.4, 26.1, 25.8.

β-Galactosidase probe 3

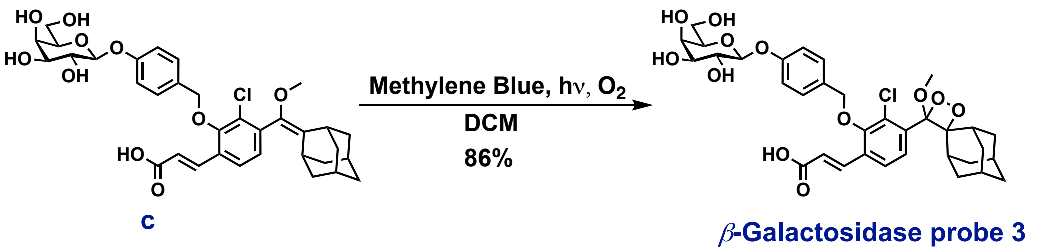

Scheme S3. Synthesis of ***β*-galactosidase probe 3**.

Compound **c** (10 mg, 0.02 mmol) and a catalytic amount of methylene blue (~1 mg) were dissolved in 10 mL of DCM. Oxygen was bubbled through the solution while irradiating with yellow light. The reaction was monitored by RP-HPLC. Upon completion, the solvent was concentrated under reduced pressure and the product was purified by preparative RP-HPLC (mobile phase: acetonitrile in H_2O containing 0.1% TFA; gradient from 70 to 100%; flow rate: 20 mL/min; retention time: 5 min). ***β*-galactosidase probe 3** was obtained as a white solid (9.0 mg, 86% yield). MS (ES+): m/z calc. for $C_{34}H_{39}ClO_{12}$: 674.21; found:675.6 $[M-H]^+$, 697.6 $[M-Na]^+$. ^1H NMR (400 MHz, DMSO) δ 7.95 (d, J = 8.4 Hz, 1H), 7.83 – 7.72 (m, J = 12.2, 6.2 Hz, 2H), 7.38 (d, J = 7.6 Hz, 2H), 7.04 (d, J = 8.6 Hz, 2H), 6.65 (d, J = 16.1 Hz, 1H), 4.93 – 4.79 (m, 3H), 3.70 (d, J = 3.3 Hz, 2H), 3.61 – 3.38 (m, 6H), 3.12 (s, 3H), 2.89 (s, 1H), 2.24 (d, J = 11.9 Hz, 1H), 1.91 (s, 1H), 1.77 – 1.15 (m, 12H). ^{13}C NMR (100 MHz, DMSO) δ 167.6, 158.2, 154.0, 137.2, 134.5, 131.8, 130.6, 129.3, 128.8, 127.3, 126.5, 123.4, 116.6, 111.7, 101.4, 95.9, 75.9, 73.7, 70.7, 68.6, 60.8, 49.9, 36.3, 33.7, 33.5, 32.3, 32.1, 31.6, 31.3, 26.0, 25.6.

Pyroglutamyl aminopeptidase probe 4

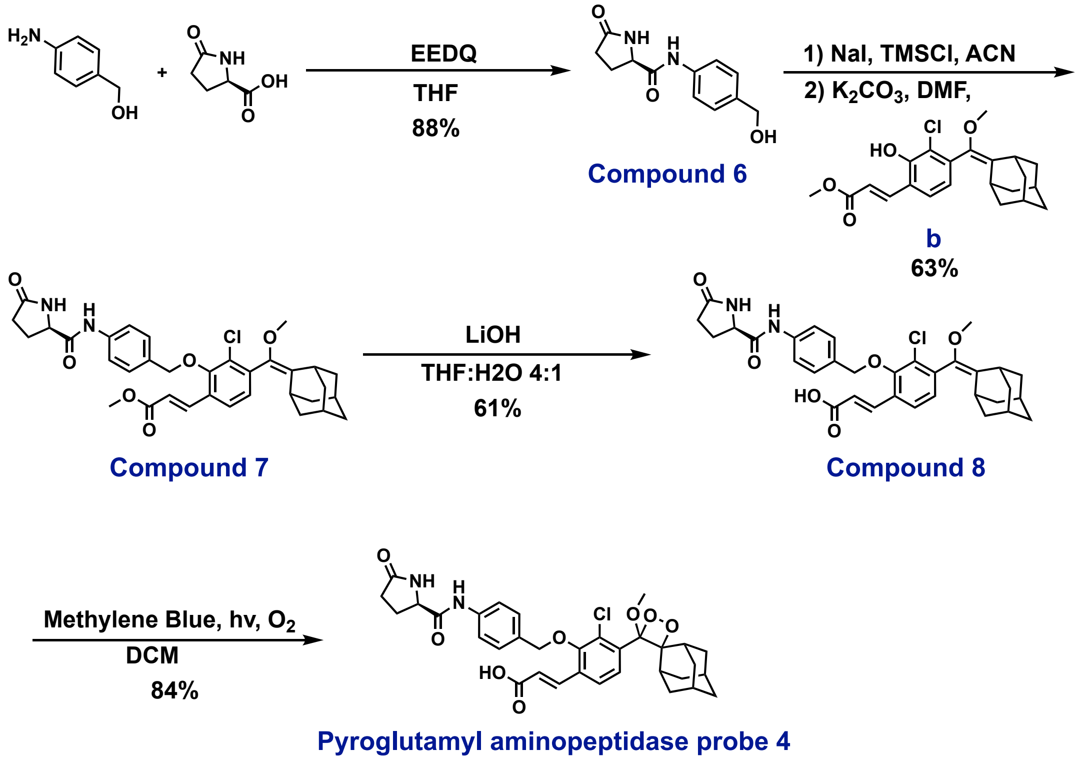

Scheme S4. Synthesis of **pyroglutamyl aminopeptidase probe 4**.

Compound 6

The 4-amino benzyl alcohol (262 mg, 2.13 mmol, 1.1 eq.) and L-pyroglutamic Acid (250 mg, 1.93 mmol, 1 eq.) were dissolved in 3 mL of THF followed by the addition of EEDQ (957 mg, 3.87 mmol, 2 eq.). The reaction mixture was stirred at room temperature and monitored by TLC (EtOAc: Hex mixture). Upon completion, the reaction mixture was filtered and washed three times with Et_2O, to afford compound **6** (400 mg, 88% yield) as a white solid. ^1H NMR (400 MHz, DMSO) δ 10.00 (s, 1H), 7.88 (s, 1H), 7.56 (d, J = 8.5 Hz, 2H), 7.25 (d, J = 8.6 Hz, 2H), 5.10 (t, J = 5.7 Hz, 1H), 4.43 (d, J = 5.6 Hz, 2H), 4.18 (dd, J = 8.5, 4.4 Hz, 1H), 2.41 – 1.91 (m, 4H). ^{13}C NMR (100 MHz, DMSO) δ 177.9, 171.6, 138.1, 137.9, 127.4, 119.6, 63.0, 56.8, 29.7, 25.8.

Compound 7

Compound **6** (400 mg, 1.71 mmol, 1 eq.) was dissolved in 4 mL of ACN and cooled to 0 ℃. Sodium Iodide (764 mg, 5.13 mmol, 3 eq.) was added followed by the rapid addition of TMS-Cl (647 µl, 5.13 mmol, 3 eq.). The reaction was allowed to warm up to room temperature and monitored by TLC (EtOAc: Hex mixture). Upon completion, the reaction mixture was diluted with EtOAc, and washed with saturated $Na_2S_2O_3$ followed by brine. The organic layer was separated, dried over Na_2SO_4, filtered and the solvent was evaporated under reduced pressure, the crude product was reacted without further purification. Phenol enol ether **b**[3] (796 mg, 2.05 mmol, 1.2 eq.) and K_2CO_3 (354 mg, 2.56 mmol, 1.5 eq.) were dissolved in DMF (5 mL). The solution was stirred for 5 minutes before crude product was added. The reaction mixture was stirred at room temperature and monitored by TLC (EtOAc:Hex mixture). Upon completion, the reaction mixture was diluted with EtOAc (100 mL) and washed with 0.1M HCl (50 mL) and brine (50 mL). The organic layer was separated, dried over Na_2SO_4, and evaporated under reduced pressure. The crude product was purified by column chromatography on silica gel (EtOAc: Hex mixture) to afford compound **7** (650 mg, 63% yield) as a white solid. MS (ES+): m/z calc. for $C_{34}H_{37}ClN_2O_6$: 604.23; found:627.6 $[M+Na]^+$. ^1H NMR (400 MHz, CDCl3) δ 8.89 (s, 1H), 7.88 (d, J = 16.2 Hz, 1H), 7.64 (d, J = 8.4 Hz, 2H), 7.49 (s, 1H), 7.45 – 7.38 (m, J = 8.2, 4.1 Hz, 3H), 7.08 (d, J = 8.0 Hz, 1H), 6.42 (d, J = 16.2 Hz, 1H), 4.95 (d, J = 3.7 Hz, 2H), 4.32 – 4.26 (m, J = 8.4, 4.9 Hz, 1H), 3.79 (s, 3H), 3.32 (s, 3H), 3.28 (s, 1H), 2.60 – 2.25 (m, 4H), 2.08 (s, 1H), 2.00 – 1.65 (m, 12H). ^{13}C NMR (100 MHz, $CDCl_3$) δ 179.6, 170.5, 167.2, 153.7, 139.4, 138.9, 138.2, 137.9, 132.5, 132.3, 129.8, 129.7, 127.8, 125.1, 120.1, 119.8, 75.7, 57.9, 57.3, 51.8, 39.2, 39.0, 38.6, 37.0, 32.9, 29.7, 29.5, 28.3, 28.2, 25.8.

Compound 8

Compound **7** (100 mg, 0.16 mmol, 1 eq.) and LiOH (39 mg, 1.6 mmol, 10 eq.) were dissolved in 1 mL solution of THF: H_2O (4:1). The reaction mixture was stirred at room temperature and monitored by RP-HPLC. Upon completion, the solvent was concentrated under reduced pressure and the product was purified by preparative RP-HPLC (mobile phase: acetonitrile in H_2O containing 0.1% TFA; gradient from 70 to 100%; flow rate: 20 mL/min; retention time: 7.5 min). Compound **8** was obtained as a white solid (59 mg, 61% yield). MS (ES+): m/z calc. for $C_{33}H_{35}ClN_2O_6$: 590.22; found:613.5 $[M+Na]^+$. ^1H NMR (400 MHz, DMSO) δ 10.10 (s, 1H), 7.86 (s, 1H), 7.76 (d, *J* = 7.1 Hz, 1H), 7.73 (s, 1H), 7.61 (d, *J* = 8.5 Hz, 2H), 7.36 (d, *J* = 8.5 Hz, 2H), 7.08 (d, *J* = 8.0 Hz, 1H), 6.55 (d, *J* = 16.1 Hz, 1H), 4.93 (d, *J* = 16.3 Hz, 2H), 4.17 (dd, *J* = 8.5, 4.3 Hz, 1H), 3.20 (s, 3H), 3.16 (s, 1H), 2.51 – 2.43 (m, 2H), 2.37 – 2.27 (m, 1H), 2.23 – 2.09 (m, 2H), 1.99 – 1.63 (m, 13H). ^{13}C NMR (100 MHz, $CDCl_3$) δ 170.8, 168.9, 153.4, 139.3, 138.0,

137. 9, 132.6, 132.0, 130.0, 129.7, 127.8, 124.9, 120.1, 119.8, 76.0, 57.6, 57.2, 39.1, 39.0, 38.6, 37.0, 32.9, 29.7, 29.4, 28.3, 28.1, 25.4.

Pyroglutamyl aminopeptidase probe 4

Compound **8** (10 mg, 0.02 mmol) and a catalytic amount of methylene blue (~1 mg) were dissolved in 10 mL of DCM. Oxygen was bubbled through the solution while irradiating with yellow light. The reaction was monitored by RP-HPLC. Upon completion, the solvent was concentrated under reduced pressure, and the product was purified by preparative RP-HPLC (mobile phase: acetonitrile in H_2O containing 0.1% TFA; gradient from 70 to 100%; flow rate: 20 mL/min; retention time: 6 min). **Pyroglutamyl aminopeptidase probe 4** was obtained as a white solid (8.8 mg, 84% yield). MS (ES+): m/z calc. for $C_{33}H_{35}ClN_2O_8$: 622.21; found:645.5 $[M-H]^+$. ^1H NMR (400 MHz, $CDCl_3$) δ 8.81 (s, 1H), 7.97 – 7.89 (m, 1H), 7.66 (dd, J = 26.2, 16.1 Hz, 1H), 7.56 (t, J = 6.5 Hz, 2H), 7.19 (dd, J = 12.2, 8.4 Hz, 2H), 6.27 (dd, J = 21.5, 16.1 Hz, 1H), 5.06 (dd, J = 31.5, 11.4 Hz, 1H), 4.85 (dd, J = 39.4, 11.3 Hz, 1H), 4.42 (s, 1H), 3.28 (d, J = 4.8 Hz, 3H), 3.06 (s, 1H), 2.67 – 2.26 (m, 4H), 2.04 (s, 1H), 1.94 – 1.24 (m, 12H). ^{13}C NMR (100 MHz, $CDCl_3$) δ 181.3, 181.2, 171.0, 169.7, 139.8, 139.7, 138.0, 135.4, 132.1, 131.9, 131.6, 130.4, 130.3, 128.9, 127.7, 124.8, 120.0, 111.7, 96.4, 58.5, 49.8, 36.6, 33.9, 33.6, 32.7, 32.23, 31.6, 29.6, 26.1, 25.9, 25.5.

Phosphatase probe 5

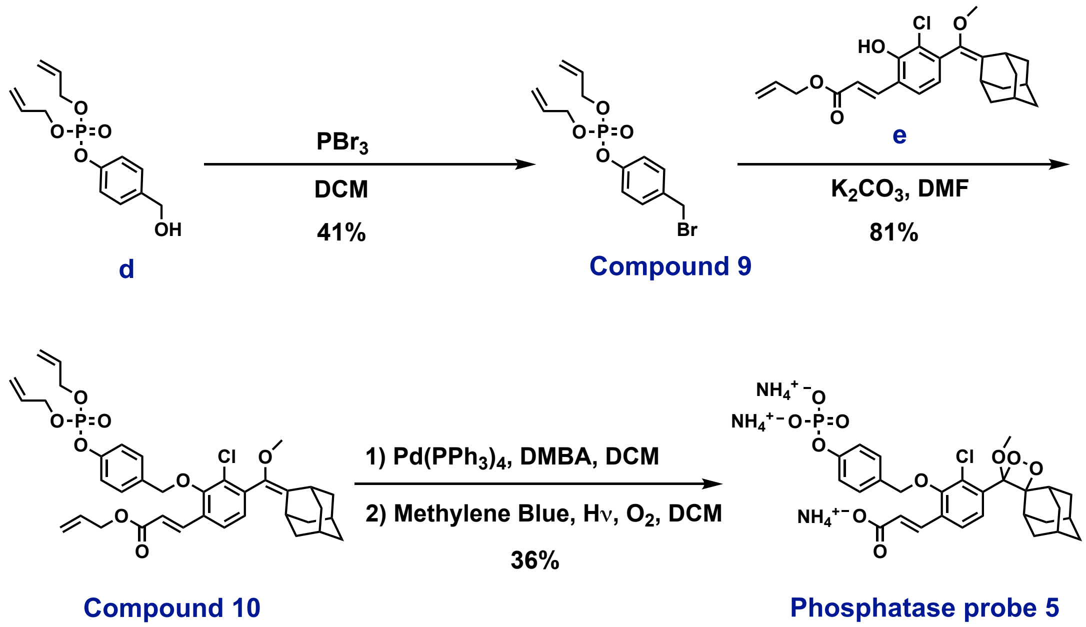

Scheme S5. Synthesis of **phosphatase probe 5**.

Compound 9

Compound **d** (100 mg, 0.35 mmol, 1 eq.) was dissolved in 1 mL DCM and cooled to 0℃ followed by dropwise addition of PBr_3 (60 µl, 0.63 mmol, 1.8 eq.). The reaction was monitored by TLC (EtOAc: Hex mixture). Upon completion, the reaction mixture was diluted with EtOAc, and washed with saturated $NaHCO_3$ followed by brine. The organic layer was separated, dried over Na_2SO_4, filtered and the solvent was evaporated under reduced pressure, the crude product was purified by column chromatography on silica gel (EtOAc: Hex gradient) to afford compound **9** (51 mg, 41% yield) as a yellow oil. MS (ES+): m/z calc. for $C_{13}H_{16}BrO_4P$: 346.00; found:371.2 $[M-H]^+$. ^1H NMR (400 MHz, $CDCl_3$) δ 7.26 (d, *J* = 8.5 Hz, 2H), 7.12 (d, *J* = 7.8 Hz, 2H), 5.94 – 5.81 (m, 2H), 5.33 (dd, *J* = 17.1, 1.4 Hz, 2H), 5.22 (dd, *J* = 10.4, 1.1 Hz, 2H), 4.63 – 4.52 (m, 6H). ^{13}C NMR (100 MHz, $CDCl_3$) δ 132.0, 131.9, 130.5, 120.4, 120.4, 118.8, 69.0, 68.9, 32.6.

Compound 10

Phenol enol ether **e**[6] (51 mg, 0.12 mmol, 1.2 eq) and K_2CO_3 (25 mg, 0.18 mmol, 1.5 eq) were dissolved in DMF (1 mL). The solution was stirred for 5 minutes before adding compound 9 (42 mg, 0.12 mmol, 1 eq). The reaction mixture was stirred at room temperature and monitored by TLC (EtOAc/Hex mixture). Upon completion, the reaction mixture was diluted with EtOAc (100 mL) and washed with 0.1M HCl (50 mL) and brine (50 mL). The organic layer was separated, dried over Na_2SO_4, and evaporated under reduced pressure. The crude product was purified by column chromatography on silica gel (EtOAc: Hex gradient). Compound **10** was obtained as a white solid (83 mg, 81% yield). MS (ES+): m/z calc. for $C_{37}H_{42}ClO_8P$: 680.23; found: 703.6 $[M+H]^+$. ^1H NMR (400 MHz, $CDCl_3$) δ 7.96 (d, J = 16.2 Hz, 1H), 7.49 – 7.43 (m, 3H), 7.23 (d, J = 7.8 Hz, 2H), 7.08 (d, J = 8.0 Hz, 1H), 6.48 (d, J = 16.2 Hz, 1H), 6.05 – 5.88 (m, 3H), 5.41 – 5.34 (m, 3H), 5.32 – 5.24 (m, 3H), 4.96 (d, J = 3.9 Hz, 2H), 4.71 (dt, J = 5.7, 1.2 Hz, 2H), 4.68 – 4.61 (m, 4H), 3.32 (s, 3H), 3.27 (s, 1H), 2.07 (s, 1H), 1.99 – 1.63 (m, 12H).^{13}C NMR (100 MHz, $CDCl_3$) δ 166.2, 153.6, 150.8, 150.7, 139.4, 138.8, 138.3, 132.9, 132.5, 132.2, 132.1, 132.0, 130.3, 129.8, 129.5, 127.9, 125.1, 120.2, 120.1, 118.7, 118.3, 75.3, 68.9, 68.8, 65.3, 57.3, 39.2, 39.0, 38.6, 37.0, 32.9, 29.7, 28.3, 28.2.

Phosphatase probe 5

Compound **10** (30 mg, 0.4 mmol, 1 eq.) was dissolved in DCM (2 mL), followed by the addition of DMBA (20 mg, 0.13 mmol, 3 eq.) and tetrakis(triphenylphosphine)palladium(5.1 mg, 0.004 mmol, 0.1 eq.). The reaction was stirred at room temperature and monitored by RP-HPLC. Upon completion, additional DCM was added (8 mL), followed by the addition of a catalytic amount of methylene blue. Then, oxygen was bubbled through the solution while irradiating with yellow light. The reaction was monitored by RP-HPLC. Upon completion, the solvent was concentrated under reduced pressure, and the product was purified by preparative RP-HPLC (mobile phase: acetonitrile in H_2O containing 0.1% TFA; gradient from 70 to 100%; flow rate: 20 mL/min; retention time: 5 min). The **phosphatase probe 5** was obtained as a white solid (9.4 mg, 36% yield). MS (ES-): m/z calc. for $C_{28}H_{30}ClO_{10}P$: 592.13; found:591.4 $[M-H]^-$. ^1H NMR (400 MHz, DMSO) δ 7.97 (d, J = 8.4 Hz, 1H), 7.82 – 7.74 (m, 2H), 7.46 (d, J = 8.5 Hz, 2H), 7.19 (dd, J = 8.6, 1.0 Hz, 2H), 6.66 (d, J = 16.1 Hz, 1H), 4.89 (d, J = 5.0 Hz, 2H), 3.13 (s, 3H), 2.89 (s, 1H), 2.25 (d, J = 12.7 Hz, 1H), 1.92 (s, 1H), 1.78 – 1.31 (m, 11H). ^{13}C NMR (100 MHz, DMSO) δ 168.4, 163.6, 155.0, 154.9, 153.9, 140.4, 135.6, 133.9, 132.8, 130.4, 129.09, 128.7, 127.2, 126.1, 120.2, 112.1, 111.7, 95.8, 76.1, 49.7, 36.3, 33.8, 33.5, 32.3, 32.12, 31.6, 31.3, 29.9, 29.34, 27.4, 27.1, 26.0, 25.7. ^{31}P NMR (162 MHz, $CDCl_3$) δ -6.32.

Leucine aminopeptidase probe 6

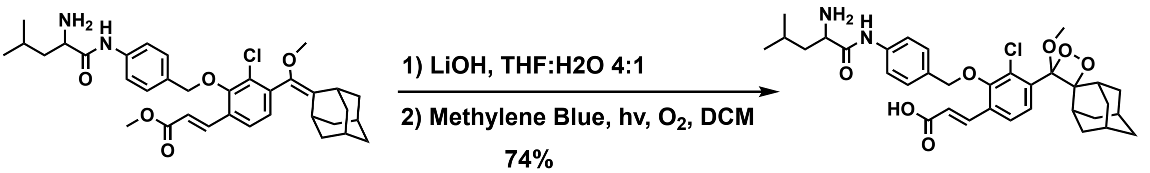

f

Leucine aminopeptidase probe 6

Scheme S6. Synthesis of **leucine aminopeptidase probe 6**.

Compound **f**[7] (25 mg, 0.04 mmol, 1 eq) and LiOH (10 mg, 0.41 mmol, 10 eq) were dissolved in 1 mL solution of THF: H_2O (4:1). The reaction mixture was stirred at room temperature and monitored by RP-HPLC. Upon completion, the reaction mixture was diluted with EtOAc (100 mL) and washed with 0.1M HCl (50 mL) and brine (50 mL). The organic layer was separated, dried over Na_2SO_4, and evaporated under reduced pressure. The crude product was reacted without further purification, and a catalytic amount of methylene blue (~1 mg) was dissolved in 10 mL of DCM. Oxygen was bubbled through the solution while irradiating with yellow light. The reaction was monitored by RP-HPLC. Upon completion, the solvent was concentrated under reduced pressure and the product was purified by preparative RP-HPLC (mobile phase: acetonitrile in H_2O containing 0.1% TFA; gradient from 50 to 100%; flow rate: 20 mL/min; retention time: 11 min). **leucine aminopeptidase probe 6** was obtained as a white solid (19 mg, 80% yield). MS (ES-): m/z calc. for $C_{34}H_{41}ClN_2O_7$: 624.26; found:625.7 $[M-H]^-$. ^1H NMR (400 MHz, DMSO) δ 10.59 (s, 1H), 8.24 (s, 2H), 7.94 (d, J = 8.4 Hz, 1H), 7.83 – 7.70 (m, 2H), 7.62 (d, J = 8.4 Hz, 2H), 7.43 (d, J = 8.4 Hz, 2H), 6.63 (d, J = 16.1 Hz, 1H), 4.89 (dd, J = 20.5, 10.8 Hz, 2H), 3.91 (s, 1H), 3.10 (s, 3H), 2.87 (s, 1H), 2.21 (d, J = 11.5 Hz, 1H), 1.90 (s, 1H), 1.78 – 1.50 (m, 11H), 1.45 (d, J = 11.8 Hz, 1H), 1.31 (d, J = 4.3 Hz, 1H), 1.27 – 1.11 (m, 2H), 0.92 (d, J = 2.4 Hz, 6H). ^{13}C NMR (100 MHz, 5% MeOD in $CDCl_3$) δ 170.0, 168.6, 154.4, 140.4, 137.3, 135.6, 132.6, 131.8, 130.3, 129.0, 127.6, 124.9, 121.0, 119.9, 111.7, 96.4, 53.5, 49.7, 40.5, 36.6, 33.9, 33.6, 32.6, 32.2, 31.5, 29.7, 26.1, 25.8, 24.4, 22.2, 22.1.

β-Lactamase probe 9

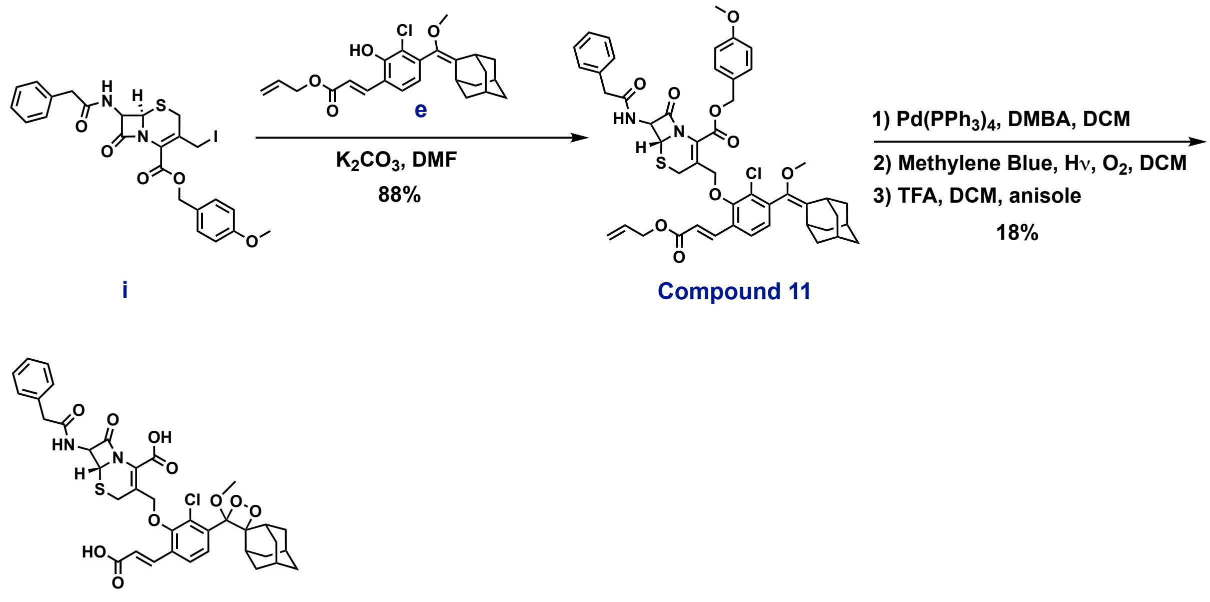

β-Lactamase probe 9

Scheme S7. Synthesis of ***β*-lactamase probe 9**.

Compound 11

Phenol enol ether **e** (57 mg, 0.14 mmol, 1.2 eq.) and K_2CO_3 (24 mg, 0.17 mmol, 1.5 eq.) were dissolved in DMF (1 mL). The solution was stirred for 5 minutes followed by the addition of compound **i**[8] (66 mg, 0.11 mmol, 1 eq.). The reaction mixture was stirred at room temperature and monitored by TLC (EtOAc/Hex mixture). Upon completion, the reaction mixture was diluted with EtOAc (100 mL) and washed with 0.1M HCl (50 mL) and brine (50 mL). The organic layer was separated, dried over Na_2SO_4, and evaporated under reduced pressure. The crude product was purified by column chromatography on silica gel (EtOAc: Hex gradient). Compound **11** was obtained as a white solid (75 mg, 88% yield). MS (ES+): m/z calc. for $C_{48}H_{49}ClN_2O_9S$: 864.28; found: 865.8 $[M+H]^+$, 887.8 $[M+Na]^+$. ^{1}H NMR (400 MHz, DMSO) δ 9.13 (d, *J* = 8.3 Hz, 1H), 7.81 (d, *J* = 8.0 Hz, 1H), 7.32 – 7.17 (m, 8H), 7.12 (d, *J* = 8.1 Hz, 1H), 6.85 (d, *J* = 8.7 Hz, 2H), 6.71 (d, *J* = 16.1 Hz, 1H), 5.97 (ddt, *J* = 17.2, 10.7, 5.5 Hz, 1H), 5.71 (dd, *J* = 7.9, 5.0 Hz, 1H), 5.33 (dd, *J* = 17.2, 1.6 Hz, 1H), 5.23 (dd, *J* = 10.4, 1.4 Hz, 1H), 5.12 (d, *J* = 4.9 Hz, 1H), 5.03 (d, *J* = 6.0 Hz, 2H), 4.92 – 4.83 (m, 1H), 4.74 – 4.69 (m, 1H), 4.69 – 4.66 (m, 2H), 3.92 (d, *J* = 18.1 Hz, 1H), 3.81 (d, *J* = 18.2 Hz, 1H), 3.71 (s, 3H), 3.56 (d, *J* = 13.9 Hz, 1H), 3.48 (d, *J* = 13.9 Hz, 1H), 3.19 (s, 3H), 3.16 (s, 1H), 1.96 – 1.59 (m, 13H). ^{13}C NMR (100 MHz, $CDCl_3$) δ 171.1, 166.4, 164.8, 161.2, 159.9, 138.7, 138.3, 133.7, 132.1, 129.4, 129.2, 128.7, 128.1, 127.7, 126.7, 125.0, 120.4, 118.5, 113.9, 72.6, 67.9, 65.4, 59.2, 57.3, 57.3, 55.3, 43.4, 39.1, 38.6, 37.0, 32.9, 29.7, 28.3, 26.5.

β-Lactamase probe 9

Compound **11** (75 mg, 0.08 mmol, 1 eq.) was dissolved in DCM (2 mL), followed by the addition of DMBA (40 mg, 0.26 mmol, 3 eq.) and tetrakis(triphenylphosphine)palladium (10 mg, 0.008 mmol, 0.1 eq.). The reaction was stirred at room temperature and monitored by RP-HPLC. Upon completion, additional DCM was added (8 mL), followed by the addition of a catalytic amount of methylene blue. Then, oxygen was bubbled through the solution while irradiating with yellow light. The reaction was monitored by RP-HPLC. Upon completion, anisole (75 μL, 0.8 mmol, 10 eq.) and TFA (200 μL, 2% v/v) were added to the reaction mixture. The reaction was monitored by RP-HPLC. Upon completion, the solvent was concentrated under reduced pressure and the product was purified by preparative RP-HPLC (mobile phase: acetonitrile in H_2O containing 0.1% TFA; gradient from 70 to 100%; flow rate: 20 mL/min; retention time: 8 min). The ***β*-lactamase probe 9** was obtained as a white solid (12 mg, 18% yield). MS (ES-): m/z calc. for $C_{37}H_{37}ClN_2O_{10}S$: 736.19; found:735.6 $[M-H]^-$. ^1H NMR (400 MHz, DMSO) δ 9.13 (dd, J = 8.3, 2.8 Hz, 1H), 7.94 (dd, J = 8.4, 2.4 Hz, 1H), 7.83 – 7.70 (m, 2H), 7.33 – 7.26 (m, 3H), 7.25 – 7.21 (m, 1H), 7.13 – 7.04 (m, 1H), 6.87 – 6.79 (m, 1H), 6.64 (dd, J = 16.1, 1.3 Hz, 1H), 5.75 – 5.65 (m, 1H), 5.13 (dd, J = 16.8, 4.9 Hz, 1H), 4.87 (t, J = 10.5 Hz, 1H), 4.77 – 4.61 (m, 1H), 3.99 – 3.85 (m, 1H), 3.77 (s, 1H), 3.70 (d, J = 2.0 Hz, 1H), 3.63 – 3.43 (m, 2H), 3.12 (s, 3H), 2.88 (s, 1H), 2.29 – 2.16 (m, 1H), 1.90 (s, 1H), 1.77 – 1.43 (m, 8H), 1.34 (d, J = 6.8 Hz, 1H), 1.26 – 1.12 (m, 2H). ^{13}C NMR (100 MHz, $CDCl_3$) δ 171.1, 166.4, 164.8, 161.2, 159.9, 138.7, 138.3, 133.7, 132.1, 130.6, 129.4, 129.2, 128.1, 127.7, 126.7, 125.0, 120.4, 118.5, 113.9, 72.6, 67.9, 65.4, 59.3, 57.3, 55.3, 43.4, 39.1, 38.6, 37.0, 32.9, 29.7, 28.34, 26.5.

Penicillin-G amidase probe 10

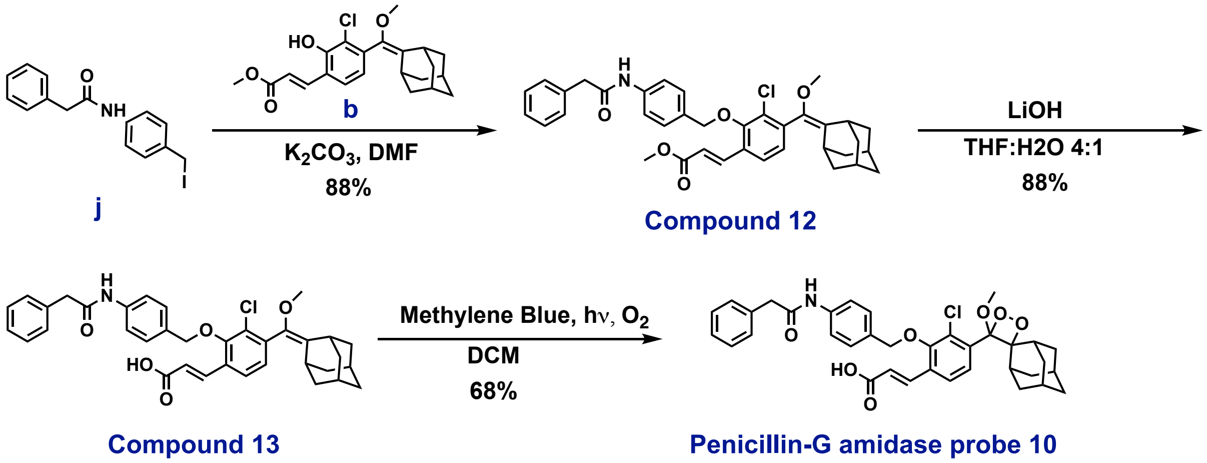

Scheme S8. Synthesis of **penicillin-G amidase probe 10**.

Compound 12

Phenol enol ether **b** (125 mg, 0.27 mmol, 1.2 eq.) and K_2CO_3 (47 mg, 0.34 mmol, 1.5 eq.) were dissolved in DMF (1 mL). The solution was stirred for 5 minutes followed by the addition of compound **j** (60 mg, 0.23 mmol, 1 eq.). The reaction mixture was stirred at room temperature and monitored by TLC (EtOAc/Hex mixture). Upon completion, the reaction mixture was diluted with EtOAc (100 mL) and washed with 0.1M HCl (50 mL) and brine (50 mL). The organic layer was separated, dried over Na_2SO_4, and evaporated under reduced pressure. The crude product was purified by column chromatography on silica gel (EtOAc: Hex gradient). Compound **12** was obtained as a white solid (122 mg, 88% yield). MS (+): m/z calc. for

$C_{37}H_{38}ClNO_5$: 611.24; found: 634.6 $[M+H]^+$. ^1H NMR (400 MHz, $CDCl_3$) δ 7.90 (d, J = 16.2 Hz, 1H), 7.48 – 7.32 (m, 10H), 7.25 (s, 1H), 7.07 (d, J = 8.0 Hz, 1H), 6.44 (d, J = 16.2 Hz, 1H), 4.96 (d, J = 4.8 Hz, 2H), 3.79 (s, 3H), 3.75 (s, 2H), 3.32 (s, 3H), 3.29 (s, 1H), 2.07 (s, 1H), 2.02 – 1.65 (m, 12H). ^{13}C NMR (100 MHz, $CDCl_3$) δ 169.1, 167.1, 153.6, 139.4, 138.9, 138.1, 138.0, 134.4, 132.4, 131.9, 129.8, 129.7, 129.5, 129.3, 127.8, 127.7, 125.1, 119.9, 119.6, 75.7, 68.0, 57.2, 51.8, 44.9, 39.2, 39.0, 38.6, 37.1, 32.9, 30.3, 29.7, 28.4, 28.2, 25.6.

Compound 13

Compound **12** (50 mg, 0.08 mmol, 1 eq.) and LiOH (20 mg, 0.8 mmol, 10 eq.) were dissolved in 1 mL solution of THF: H_2O (4:1). The reaction mixture was stirred at room temperature and monitored by RP-HPLC. Upon completion, the solvent was concentrated under reduced pressure, and the product was purified by preparative RP-HPLC (mobile phase: acetonitrile in H_2O containing 0.1% TFA; gradient from 70 to 100%; flow rate: 20 mL/min; retention time: 13.5 min). Compound **13** was obtained as a white solid (43 mg, 88% yield). MS (ES+): m/z calc. for $C_{36}H_{36}ClNO_5$: 597.23; found: 620.5 $[M+Na]^+$. ^1H NMR (400 MHz, DMSO) δ 10.22 (s, 1H), 7.75 (dd, *J* = 12.1, 6.1 Hz, 2H), 7.58 (d, *J* = 8.5 Hz, 2H), 7.33 (d, *J* = 8.6 Hz, 2H), 7.30 (d, *J* = 1.9 Hz, 2H), 7.08 (d, *J* = 8.0 Hz, 1H), 6.55 (d, *J* = 16.1 Hz, 1H), 4.91 (d, *J* = 18.4 Hz, 2H), 3.62 (s, 2H), 3.19 (s, 3H), 3.16 (s, 1H), 2.48 (d, *J* = 1.8 Hz, 4H), 1.94 – 1.82 (m, 5H), 1.80 – 1.58 (m, 8H). ^{13}C NMR (100 MHz, $CDCl_3$) δ 171.0, 169.5, 153.7, 140.8, 139.4, 138.5, 138.0, 134.3, 132.6, 131.9, 129.9, 129.5, 129.2, 127.8, 127.7, 125.1, 120.0, 119.2, 75.9, 57.3, 44.7, 39.2, 39.0, 38.6, 37.1, 32.1, 29.7, 29.3, 28.2.

Penicillin-G amidase probe 10

Compound **13** (50 mg, 0.08 mmol) and a catalytic amount of methylene blue (~1 mg) were dissolved in 10 mL of DCM. Oxygen was bubbled through the solution while irradiating with yellow light. The reaction was monitored by RP-HPLC. Upon completion, the solvent was concentrated under reduced pressure and the product was purified by preparative RP-HPLC (mobile phase: acetonitrile in H_2O containing 0.1% TFA; gradient from 70 to 100%; flow rate: 20 mL/min; retention time: 11 min). The **penicillin-G amidase probe 10** was obtained as a white solid (35 mg, 68% yield). MS (ES+): m/z calc. for $C_{36}H_{36}ClNO_7$: 629.22; found:652.6 $[M+Na]^+$. ^1H NMR (400 MHz, $CDCl_3$) δ 7.96 – 7.85 (m, 2H), 7.57 (d, J = 8.9 Hz, 2H), 7.48 (d, J = 8.3 Hz, 2H), 7.42 – 7.30 (m, 6H), 6.40 (d, J = 16.1 Hz, 1H), 4.93 (d, J = 2.8 Hz, 2H), 3.74 (s, 2H), 3.25 (s, 3H), 3.05 (s, 1H), 2.34 (d, J = 12.1 Hz, 1H), 2.02 (s, 1H), 1.94 – 1.57 (m, 8H), 1.49 (d, J = 11.1 Hz, 1H), 1.36 (s, 2H). ^{13}C NMR (101 MHz, $CDCl_3$) δ 170.8, 169.7, 140.2, 138.2, 135.6, 134.3, 131.6, 131.5, 130.0, 129.5, 129.2, 129.0, 127.8, 127.7, 125.3, 120.3, 120.1, 96.4, 76.1, 49.8, 44.7, 36.6, 33.9, 33.6, 32.6, 32.2, 31.6, 31.5, 26.2, 25.8.

Periodate oxidative-cleavage probe 11

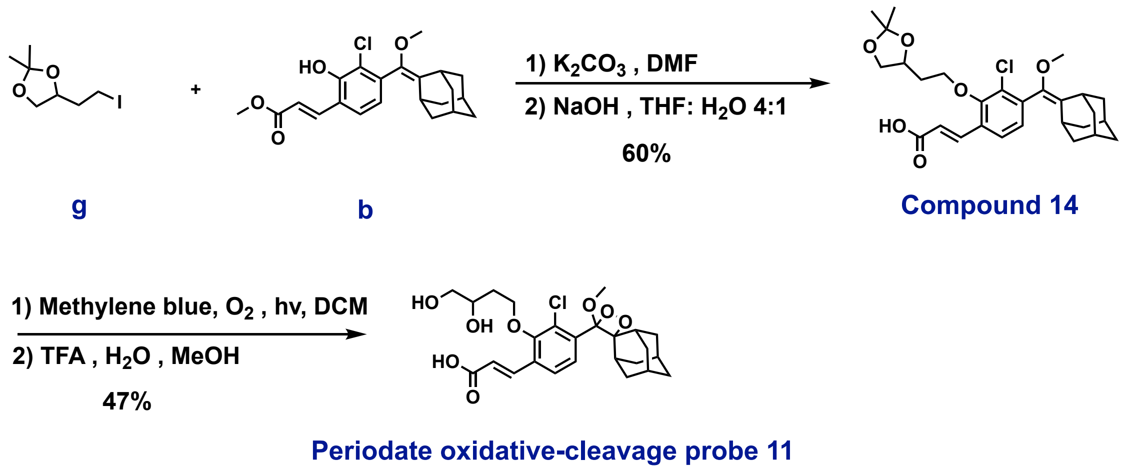

Scheme S9. Synthesis of **periodate oxidative-cleavage probe 11**.

Compound 14

Compound **g**[9] (13 mg, 0.05 mmol, 1.0 eq) was added to a solution of phenolic enol-ether **b** (23 mg, 0.06 mmol, 1.2 eq.) and K_2CO_3 (8.5 mg, 0.06 mmol, 1.2 eq.) in DMF (1 mL). The reaction was stirred at 40°C and monitored by TLC. Upon completion, the mixture was diluted with EtOAc (100 mL) and washed with 0.5M HCl (50 mL). The organic layer was separated, washed with brine, dried over Na_2SO_4 and evaporated under reduced pressure. The crude residue was dissolved in 5 mL THF : H_2O (4:1) followed by the addition of NaOH (20 mg, 0.5 mmol, 10 eq.). The reaction mixture was stirred for 2 hours at 50°C and monitored by RP-HPLC. Upon completion, the mixture was diluted with EtOAc (100 mL) and washed with 0.5M HCl (50 mL). The organic layer was separated, washed with brine, dried over Na_2SO_4, and evaporated under reduced pressure. The crude product was purified by column chromatography on silica gel (EtOAc: Hex gradient) to afford compound **14** (19 mg, 60% yield) as an off-white solid. ^1H NMR (400 MHz, $CDCl_3$) δ 7.68 (d, J = 16.1 Hz, 1H), 7.42 (d, J = 8.0 Hz, 1H), 6.98 (d, J = 8.0 Hz, 1H), 6.47 (d, J = 16.1 Hz, 1H), 4.44 – 4.35 (m, 1H), 4.19 (dd, J = 8.1, 6.0 Hz, 1H), 4.08 – 3.92 (m, 2H), 3.26 (s, 3H), 3.20 (s, 1H), 2.14 – 2.07 (m, 2H), 2.03 (s, 1H), 1.96 – 1.58 (m, 13H), 1.35 (d, J = 19.0 Hz, 6H). ^{13}C NMR (100 MHz, $CDCl_3$) δ 173.3, 153.6, 139.4, 136.9, 134.9, 132.5, 130.3, 129.2, 127.6, 125.9, 124.8, 118.0, 116.1, 109.8, 73.7, 71.1, 69.5, 57.1, 39.0, 38.9, 38.6, 38.5, 37.0, 33.8, 32.8, 29.6, 26.7, 25.4.

Periodate oxidative-cleavage probe 11

Compound 14 (10 mg, 0.05 mmol) and a catalytic amount of methylene blue were dissolved in 10 mL of DCM. Then, oxygen was bubbled through the solution while irradiating with yellow light. The reaction was monitored by RP-HPLC. Upon completion, the solvent was concentrated under reduced pressure. The crude product was dissolved in MeOH (3 mL) followed by the addition of H_2O (3 mL, 1% TFA). Once the acetonide's hydrolysis terminated (monitored by RP-HPLC) the solvent was concentrated under reduced pressure and the product was purified by preparative RP-HPLC (mobile phase: acetonitrile in H_2O containing 0.1% TFA; gradient from 50 to 100%; flow rate: 20 mL/min; retention time: 9 min). The **periodate oxidative-cleavage probe 11** was obtained as a white solid (4.6 mg, 47% yield). MS (ES-): m/z calc. for $C_{25}H_{31}ClO_8$: 494.17; found:493.6 $[M-H]^-$. ^1H NMR (400 MHz, $CDCl_3$) δ 8.13 (d, J = 16.2 Hz, 1H), 7.94 (d, J = 8.3 Hz, 1H), 7.63 (d, J = 8.4 Hz, 1H), 6.55 (d, J = 16.2 Hz, 1H), 4.37 (s, 1H), 4.17 – 4.02 (m, 3H), 3.83 (d, J = 10.5 Hz, 1H), 3.65 (d, J = 7.6 Hz, 1H),

3.23 (d, J = 6.0 Hz, 3H), 3.04 (s, 1H), 2.30 (s, 1H), 2.12 – 1.95 (m, 2H), 1.75 (m, 9H), 1.48 (d, J = 12.8 Hz, 1H), 1.40 – 1.27 (m, 2H). ^{13}C NMR (100 MHz, $CDCl_3$) δ 170.8, 154.7, 140.3, 135.8, 130.6, 129.0, 127.7, 125.0, 120.2, 111.6, 96.4, 70.9, 69.0, 67.0, 49.7, 36. 6, 33.9, 33.6, 32.8, 32.6, 32.2, 31.6, 31.5, 26.1, 25.8.

N-Acetyl hydrolase probe 12

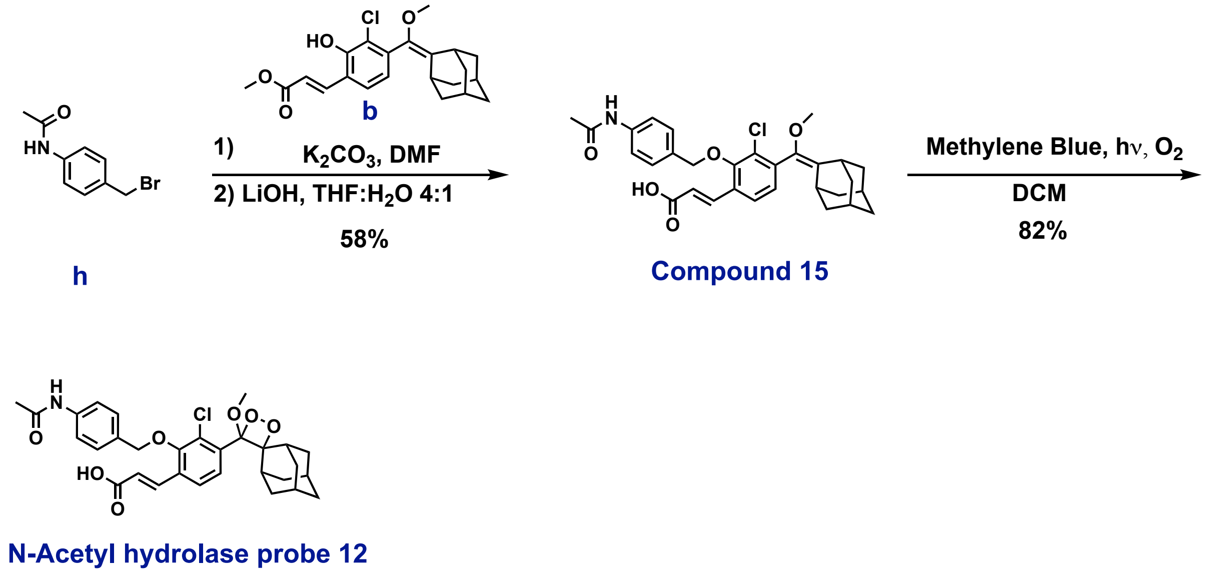

Scheme S10. Synthesis of **N-acetyl hydrolase probe 12**.

Compound 15

Phenol enol ether **b** (46 mg, 0.12 mmol, 1 eq.) and K_2CO_3 (24 mg, 0.17 mmol, 1.5 eq.) were dissolved in DMF (0.5 mL). The solution was stirred for 5 minutes before adding compound **h** (27 mg, 0.12 mmol, 1 eq.). The reaction mixture was stirred at room temperature and monitored by TLC (EtOAc: Hex mixture). Upon completion, the reaction mixture was diluted with EtOAc (100 mL) and washed with saturated NH_4Cl (50 mL) and brine (50 mL). The organic layer was separated, dried over Na_2SO_4, and evaporated under reduced pressure. The crude mixture was reacted without further purification. The crude and LiOH (9.5 mg, 0.39 mmol, 10 eq.) were dissolved in 1 mL solution of THF: H_2O (4:1). The reaction mixture was stirred at 50 °C and monitored by RP-HPLC. Upon completion, the solvent was concentrated under reduced pressure, and the product was purified by preparative RP-HPLC (mobile phase: acetonitrile in H_2O containing 0.1% TFA; gradient from 70 to 100%; flow rate: 20 mL/min; retention time: 10 min). Compound **15** was obtained as a white solid (36 mg, 58% yield). MS (ES+): m/z calc. for $C_{30}H_{32}ClNO_5$: 521.20; found: 544.5 $[M+Na]^+$. ^{1}H NMR (400 MHz, 5% MeOD in $CDCl_3$) δ 7.92 (dd, J = 20.5, 12.2 Hz, 2H), 7.81 (s, 1H), 7.56 (d, J = 8.2 Hz, 3H), 7.39 (d, J = 8.2 Hz, 2H), 6.39 (d, J = 16.1 Hz, 1H), 4.94 (d, J = 2.6 Hz, 3H), 3.26 (s, 3H), 3.06 (s, 1H), 2.36 (d, J = 12.2 Hz, 1H), 2.20 (s, 3H), 2.03 (s, 1H), 1.79 (ddd, J = 55.0, 37.2, 15.0 Hz, 8H), 1.50 (d, J = 12.7 Hz, 1H), 1.37 (s, 2H). ^{13}C NMR (100 MHz, 5% MeOD in CDCl3) δ 169.5, 169.0, 153.5, 139.5, 139.3, 138.4, 138.0, 132.6, 131.5, 129.9, 129.7, 127. 8, 125.0, 120.0, 119.8, 76.0, 57.2, 39.1, 39.0, 38.6, 37.0, 32.9, 29.7, 28.3, 28.2, 24.1.

N-Acetyl hydrolase probe 12

Compound **15** (10 mg, 0.02 mmol) and a catalytic amount of methylene blue (~1 mg) were dissolved in 10 mL of DCM. Oxygen was bubbled through the solution while irradiating with yellow light. The reaction was monitored by RP-HPLC. Upon completion, the solvent was concentrated under reduced pressure, and the product was purified by preparative RP-HPLC (mobile phase: acetonitrile in H_2O containing 0.1% TFA; gradient from 70 to 100%; flow rate: 20 mL/min; retention time: 8 min). The **N-acetyl hydrolase probe 12** was obtained as a white solid (8.7 mg, 82% yield). MS (ES+): m/z calc. for $C_{30}H_{32}ClNO_7$: 553.19; found:576.5 $[M-H]^+$. ^{1}H NMR (400 MHz, $CDCl_3$) δ 7.92 (dd, J = 20.5, 12.2 Hz, 2H), 7.81 (s, 1H), 7.57 (t, J = 8.2 Hz, 3H), 7.39 (d, J = 8.2 Hz, 2H), 6.39 (d, J = 16.1 Hz, 1H), 5.00 – 4.89 (m, 2H), 3.26 (d, J = 6.4 Hz, 3H), 3.06 (s, 1H), 2.36 (d, J = 12.2 Hz, 1H), 2.20 (s, 3H), 2.03 (s, 1H), 1.97 – 1.24 (m, 12H). ^{13}C NMR (100 MHz, $CDCl_3$) δ 170.6, 169.1, 154.1, 140.2, 138.5, 135.6, 131.6, 131.4, 130.2, 129.0, 127.8, 125.2, 120.2, 120.2, 111.7, 96.4, 76.16, 49.8, 36.6, 33.9, 33.6, 32.6, 32.2, 31.6, 31.5, 26.2, 25.8, 24.5.

Fluorescent phosphatase probe - Compound 17

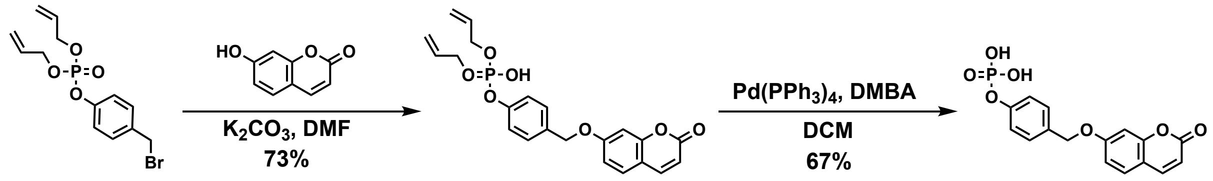

Compound 9 Compound 16 Compound 17

Scheme S11. Synthesis of **fluorescent phosphatase probe** - Compound 17.

Compound 16

Umbelliferone (12 mg, 0.07 mmol, 1.2 eq.) and K_2CO_3 (12 mg, 0.09 mmol, 1.5 eq.) were dissolved in DMF (1 mL). The solution was stirred for 5 minutes followed by the addition of compound **9** (21 mg, 0.06 mmol, 1 eq.). The reaction mixture was stirred at room temperature and monitored by TLC (EtOAc/Hex mixture). Upon completion, the reaction mixture was diluted with EtOAc (100 mL) and washed with 0.1M HCl (50 mL) and brine (50 mL). The organic layer was separated, dried over Na_2SO_4, and evaporated under reduced pressure. The crude product was purified by column chromatography on silica gel (EtOAc: Hex gradient). Compound **16** was obtained as a yellow oil (18 mg, 73% yield). ^{1}H NMR (400 MHz, DMSO) δ 7.99 (d, *J* = 9.5 Hz, 1H), 7.64 (d, *J* = 8.6 Hz, 1H), 7.52 (d, *J* = 8.5 Hz, 2H), 7.25 (dd, *J* = 8.6, 1.0 Hz, 2H), 7.08 (d, *J* = 2.4 Hz, 1H), 7.02 (dd, *J* = 8.6, 2.4 Hz, 1H), 6.29 (d, *J* = 9.5 Hz, 1H), 6.01 – 5.87 (m, 2H), 5.36 (dd, *J* = 17.2, 1.6 Hz, 2H), 5.25 (dd, *J* = 10.5, 1.4 Hz, 2H), 5.21 (s, 2H), 4.64 (ddt, *J* = 8.4, 5.4, 1.4 Hz, 4H). ^{13}C NMR (100 MHz, $CDCl_3$) δ 161.7, 161.3, 155.8, 143.50, 132.7, 131.9, 131.9, 129.1, 128.9, 120.4, 120.3, 118.8, 113.25, 112.8, 101.97, 69.8, 69.0, 68.9. ^{31}P NMR (162 MHz, DMSO) δ -6.27.

Compound 17

Compound **16** (18 mg, 0.04 mmol, 1 eq.) was dissolved in DCM (2 mL), followed by the addition of DMBA (21 mg, 0.13 mmol, 3 eq.) and tetrakis(triphenylphosphine)palladium(5.1 mg, 0.004 mmol, 0.1 eq.). The reaction was stirred at room temperature and monitored by RP-HPLC. Upon completion, the solvent was concentrated under reduced pressure, and the product was purified by preparative RP-HPLC (mobile phase: acetonitrile in H_2O containing 0.1% TFA; gradient from 30 to 100%; flow rate: 20 mL/min; retention time: 5 min). Compound **17** was obtained as a white solid (9.8 mg, 67% yield). MS (ES-): m/z calc. for $C_{16}H_{13}O_7P$: 348.04; found: 347.3 $[M-H]^-$. ^1H NMR (400 MHz, MeOD) δ 7.89 (d, J = 9.5 Hz, 1H), 7.55 (d, J = 8.4 Hz, 1H), 7.48 (d, J = 8.5 Hz, 2H), 7.25 (dd, J = 8.6, 1.1 Hz, 2H), 7.03 – 6.96 (m, 2H), 6.26 (d, J = 9.5 Hz, 1H), 5.18 (s, 2H), 1.31 (s, 1H). ^{13}C NMR (100 MHz, MeOD) δ 162.2, 162.0, 155.8, 151.4, 144.4, 132.8, 129.2, 128.9, 120.3, 113.2, 112.3, 101.5, 69.7. ^{31}P NMR (162 MHz, MeOD) δ -5.24.

Colorimetric phosphatase probe - Compound 19

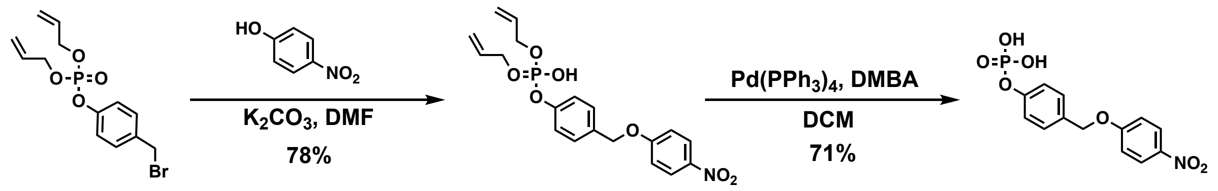

Compound 9 **Compound 18** **Compound 19**

Scheme S12. Synthesis of **colorimetric phosphatase probe** - Compound 19.

Compound 18

The 4-nitrophenol (9.7 mg, 0.07 mmol, 1.2 eq.) and K_2CO_3 (12 mg, 0.09 mmol, 1.5 eq.) were dissolved in DMF (1 mL). The solution was stirred for 5 minutes followed by the addition of compound **9** (21 mg, 0.06 mmol, 1 eq.). The reaction mixture was stirred at room temperature and monitored by TLC (EtOAc/Hex mixture). Upon completion, the reaction mixture was diluted with EtOAc (100 mL) and washed with 0.1M HCl (50 mL) and brine (50 mL). The organic layer was separated, dried over Na_2SO_4, and evaporated under reduced pressure. The crude product was purified by column chromatography on silica gel (EtOAc: Hex gradient). Compound **18** was obtained as a yellow oil (22 mg, 78% yield). ^1H NMR (400 MHz, $CDCl_3$) δ 8.26 – 8.14 (m, 2H), 7.39 (dd, J = 18.4, 6.4 Hz, 2H), 7.32 – 7.22 (m, 2H), 7.07 – 6.96 (m, 2H), 5.96 (ddd, J = 22.6, 10.8, 5.6 Hz, 2H), 5.38 (dt, J = 14.7, 7.3 Hz, 2H), 5.27 (dt, J = 12.2, 6.1 Hz, 2H), 5.14 (s, 2H), 4.66 (tt, J = 17.1, 6.3 Hz, 4H). ^{13}C NMR (100 MHz, $CDCl_3$) δ 163.5, 150.7, 150.7, 141.8, 132.3, 132.0, 132.0, 129.0, 126.0, 120.5, 120.4, 118. 8, 114.8, 70.0, 68.9, 68.9.

Compound 19

Compound **18** (16 mg, 0.04 mmol, 1 eq.) was dissolved in DCM (2 mL), followed by the addition of DMBA (21 mg, 0.13 mmol, 3 eq.) and tetrakis(triphenylphosphine)palladium(5.1 mg, 0.004 mmol, 0.1 eq.). The reaction was stirred at room temperature and monitored by RP-HPLC. Upon completion, the solvent was concentrated under reduced pressure, and the product was purified by preparative RP-HPLC (mobile phase: acetonitrile in H_2O containing 0.1% TFA; gradient from 30 to 100%; flow rate: 20 mL/min; retention time: 7 min). **Compound 19** was obtained as a white solid (8.7 mg, 67% yield). MS (ES-): m/z calc. for $C_{13}H_{12}NO_7P$: 325.04; found:324.2 $[M-H]^-$. ^1H NMR (400 MHz, 10% MeOD in $CDCl_3$) δ 8.22 (d, J = 9.3 Hz, 2H), 7.47 (d, J = 8.5 Hz, 2H), 7.26 (d, J = 7.7 Hz, 2H), 7.16 (d, J = 9.3 Hz, 2H), 5.21 (s, 2H). ^{13}C NMR (100 MHz, 10% MeOD in $CDCl_3$) δ 163.7, 151.4, 141.7, 131.8, 129.0, 126.0, 120.7, 114.9, 70.2, 50.0, 49.8, 49.6, 49.4, 49.2, 49.0, 48.8. ^{31}P NMR (162 MHz, 10% MeOD in $CDCl_3$) δ -4.77 (s).

Biological evaluation: general information and procedures

List of bacteria strains

	#	Species	Strain	Source	Growth condition
Gram-positive bacteria					
1	1	*Streptococcus pyogenes*	14289	Clinical isolate	BHI, 37°C
	2	*Streptococcus pyogenes*	CI-1	Clinical isolate	BHI, 37°C
	3	*Streptococcus pyogenes*	CI-2	Clinical isolate	BHI, 37°C
2	4	*Streptococcus mutans*	35668	ATCC	BHI, 37°C
3	5	*Staphylococcus aureus*	35556	ATCC	LB, 37°C
	6	*Staphylococcus aureus*	9144	ATCC	LB, 37°C
	7	*Staphylococcus aureus*	29213	ATCC	LB, 37°C
	8	*Staphylococcus aureus* (MRSA)	33591	ATCC	BHI, 37°C
	9	*Staphylococcus aureus* (MRSA)	33592	ATCC	BHI, 37°C
	10	*Staphylococcus aureus* (MRSA)	BAA-43	ATCC	BHI, 37°C
	11	*Staphylococcus aureus* (MRSA)	43300	ATCC	BHI, 37°C
4	12	*Staphylococcus epidermidis*	12228	ATCC	LB, 37°C
5	13	*Bacillus cereus*	14579	ATCC	LB, 30°C
6	14	*Bacillus subtilis*	14945	ATCC	LB, 30°C
7	15	*Bacillus thuringiensis*	35646	ATCC	LB, 30°C
8	16	*Enterococcus faecalis*	29212	ATCC	LB, 37°C
9	17	*Enterococcus faecium*	19434	ATCC	LB, 37°C
10	18	*Listeria monocytogenes*	19115	ATCC	BHI, 37°C
Gram-negative bacteria					
11	19	*Escherichia coli*	25922	ATCC	LB, 37°C
	20	*Escherichia coli*	9637	ATCC	LB, 37°C
	21	*Escherichia coli*	CI-1	Daniel Kahne	LB, 37°C
	22	*Escherichia coli*	CI-2	Clinical isolate	LB, 37°C
	23	*Escherichia coli*	BAA-2452	ATCC	LB, 37°C
12	24	*Pseudomonas aeruginosa*	47085	ATCC	LB, 37°C
	25	*Pseudomonas aeruginosa*	15692	ATCC	LB, 37°C
	26	*Pseudomonas aeruginosa*	PAO1	-	LB, 37°C
	27	*Pseudomonas aeruginosa*	27853	ATCC	LB, 37°C
13	28	*Klebsiella pneumoniae*	BAA-2470	ATCC	LB, 37°C
	29	*Klebsiella pneumoniae*	10031	ATCC	LB, 37°C
14	30	*Acinetobacter baumannii*	19606	ATCC	LB, 37°C
15	31	*Haemophilus influenza*	49247	ATCC	*sBHI, 37°C
16	32	*Burkholderia cepacia*	25416	ATCC	LB, 30°C
17	33	*Enterobacter cloaca*	13047	ATCC	LB, 30°C

* Supplemented BHI with hemin and nicotinamide adenine dinucleotide.

Table S1. Strains information.

L.O.D experiments procedure

Staphylococcus aureus ATCC 29213 was cultured in LB at 37°C for 18 hours under aerobic conditions. The initial culture was rinsed with PBS (centrifuged at 5000 rpm,10 minutes), and the resulting bacterial pellet was resuspended in 4 mL of PBS to facilitate a 1:5 dilution experiment. For the subsequent procedure, a 96-well plate was utilized, with each well initially loaded with 50μL of the phosphatase chemiluminescent probe 5 (20μM, 0.1% DMSO v/v in PBS pH 7.4), Phosphatase colorimetric probe (compound 17, 100μM, 0.1% DMSO v/v in PBS pH 7.4), or phosphatase fluorescence probe (compound 19, 20μM, 0.1% DMSO v/v in PBS pH 7.4). Subsequently, 50μL of bacterial aliquot was introduced into each well*,* marking the commencement of the 1:5 dilution experiment, which was initiated with an OD_{600} of 0.4. The ensuing chemiluminescence signal was monitored over 30 minutes of incubation at 37°C using a Molecular Devices Spectramax i3x. Simultaneously, the fluorescence signal was recorded during the same timeframe at the same temperature using a Tecan Infinite 200 Pro.

Enzymatic substrate specificity experiments

The specificity of each chemiluminescent probe was assessed in the presence of ten commercially available recombinant enzymes. The chemiluminescent intensity of each probe [10 µM in PBS (pH 7.4), 0.1% DMSO, 37°C] was measured in conjunction with the following commercially available recombinant enzymes: *β*-glucosidase (from almonds) [10 U/mL], *β*-glucuronidase (from *E. coli*) [1 U/mL], *β*-galactosidase from *E. coli* [1 U/mL], pyroglutamyl-peptidase I from *E. coli* [0.05 mg/mL], alkaline phosphatase from bovine intestinal mucosa [1 U/mL], aminopeptidase-M from porcine kidney [1 U/mL], nitroreductase from *E. coli* [1 mg/mL, 100μM NADH], NQO1 from *E. coli* [0.8 mg/mL, 100μM NADH], *β*-lactamase from *E. coli* [2 U/mL], penicillin-G amidase (from *E. coli)* [1 U/mL].

Chemiluminescent measurements of bacterial enzymatic activity fingerprint

All bacterial strains were cultured in LB/BHI broth at either 37°C or 30°C for 18 hours under aerobic conditions. Subsequently, the initial culture was subjected to a PBS wash (centrifuged at 5000 rpm,10 minutes), and the bacterial pellet obtained was reconstituted in 4 mL of PBS, aiming for an OD_{600} of 0.8. Following this, a 96-well plate was utilized, and each well was pre-loaded with 50 μL of each chemiluminescent probe (20 μM, 0.1% DMSO v/v in PBS pH 7.4). Next, 50 μL of bacterial aliquot was introduced into each well, bringing the final OD_{600} to 0.4. The resultant chemiluminescence signal was monitored using a Molecular Devices Spectramax iD3 over the course of 1 hour of incubation at 37°C.

Chemiluminescent measurements of enzymatic activity fingerprint in bacterial mixtures

Staphylococcus aureus MRSA ATCC 33592 and *Escherichia coli* ATCC 25922 were each cultured in LB at 37°C for 18 hours under aerobic conditions. The initial cultures of both bacteria were subjected to a PBS wash (centrifuged at 5000 rpm,10 minutes), and the bacterial pellets obtained were reconstituted in 4 mL of PBS, aiming for an OD_{600} of 0.8. Following this, a 96-well plate was utilized, and each well was pre-loaded with 50 μL of each chemiluminescent probe (20 μM, 0.1% DMSO v/v in PBS pH 7.4). Next, 50 μL of bacteria mixture aliquot (1:1,9:1, 1:9 of *E. coli*, MRSA respectively) was introduced into each well. The resultant chemiluminescence signal was monitored using a Molecular Devices Spectramax iD3 over the course of 1 hour of incubation at 37°C.

Computational methods

General methods

The 1-N-N classification method was executed using MATLAB's K-Nearest Neighbors (KNN) algorithm, where K was set to 1, and the Euclidean distance metric was employed for similarity measurement. Furthermore, MATLAB's built-in Principal Component Analysis (PCA) implementation was utilized for visualization, primarily aimed at dimensionality reduction of the dataset. To comprehensively evaluate the efficacy of our approach, the MATLAB KNN classifier was applied to all 4094 potential probe combinations, with K set to 1 and the Euclidean distance metric. This enabled measurement of classification accuracy across all probe combinations, facilitating a robust assessment of the method's performance.

Data acquisition and processing

The data acquisition process involved meticulous aggregation from multiple sources. For each measurement, raw data were gathered by evaluating three distinct wells harboring the target bacteria, contrasted with three control wells that lacked bacteria and served as background references. The processing of these raw data entailed a series of steps. Initially, the mean light intensity value was calculated across measurements from the three wells containing bacteria and similarly for the control wells. Following this, the light intensity was integrated over a one-hour interval in the presence and absence of bacteria. A crucial stage involved subtracting the logarithm of TLE gathered from the control wells (termed "Control") from the logarithm of the TLE gathered from the bacteria-containing wells. To ensure consistency, any negative log

ratios were set to zero. This process culminated in the generation of a 12-dimensional vector for each strain, with entries corresponding to the distinct probes in our array.

1-N-N Analysis

1-N-N Analysis computational analysis of data involved the manipulation of 12-dimensional vectors containing background-subtracted log-intensity data. To accomplish this, the K-Nearest Neighbors (KNN) algorithm was employed. Specifically, the MATLAB implementation of KNN was utilized with K set to 1. In congruence with this choice, the Euclidean distance was adopted as the metric to facilitate pairwise comparisons between data points. The KNN algorithm facilitates informed labeling of unknown data points within the test set. This is achieved by assigning to each unknown data point the label of its nearest counterpart within the training set. To ensure the robustness and reliability of outcomes, the dataset of enzymatic profiles underwent a randomized split, resulting in two distinct data sets: the training set encompassing 72% of the data and the test set comprising the remaining 28%. This partitioning strategy is designed to enhance precision and dependability in the classification results for log-intensity data. To guarantee comprehensive coverage and robust evaluation, it is important to note that the partitioning strategy employed also ensures that each bacterium within the dataset is included in both the training and test set, thereby allowing for a thorough assessment of the classification performance. By employing this approach, effective categorization of new data points is achieved through their proximity to reference points within the training set.

Details of the chi-squared (χ^2) resemblance ranking and test

Definition of the Resemblance Test: The methodology employed in this study focuses on the characterization and classification of bacterial strains based on their enzymatic profiles within a 12-dimensional space. It is grounded in the assumption that these profiles conform to Gaussian distributions. To assess the degree of similarity between an unfamiliar bacterial strain and those cataloged within our dataset, we employ a probability-centered approach. Central to this approach is the Chi-Squared (χ^2) metric, a statistical measure that quantifies deviations from reference statistics and is crucial for evaluating the resemblance of unknown bacteria to those within a reference dataset. The degrees of freedom for the χ^2statistic is set to 12, corresponding to the 12 dimensions of the enzymatic profile space. This systematic methodology serves as the foundation for probabilistic classification and allows for the identification and categorization of novel bacteria based on their enzymatic profiles.

Data Retrieval: Enzymatic profile data for known bacterial species and the enzymatic profile of an unknown bacterium are retrieved from a structured Excel dataset.

Parameter Extraction: For each known bacterial species, mean vectors and variances are computed to characterize their respective Gaussian distributions. These parameters are pivotal for subsequent analyses.

Calculation of Chi-Squared Statistics: The Chi-Squared statistic is computed by centering and normalizing the mean vectors and variances of the known bacterial species and the unknown bacterium. Careful precautions are taken to prevent division by zero.

Hypothesis Testing: To determine the extent of dissimilarity between the unknown bacterium and the reference species, Chi-Squared statistics and associated p-values are calculated. These p-values are essential for hypothesis testing.

Probabilistic Classification: The Chi-Squared statistics are used to rank the similarity between the unknown bacterium and the reference species. This ranking provides a quantitative measure of resemblance.

Statistical analysis: Data are presented as means (three or more replicates) ± SD (error bars). All statistical analyses were performed using the two-tailed Student's t-test unless otherwise indicated. Differences were considered significant at $p < 0.05$.

Supplementary Figures

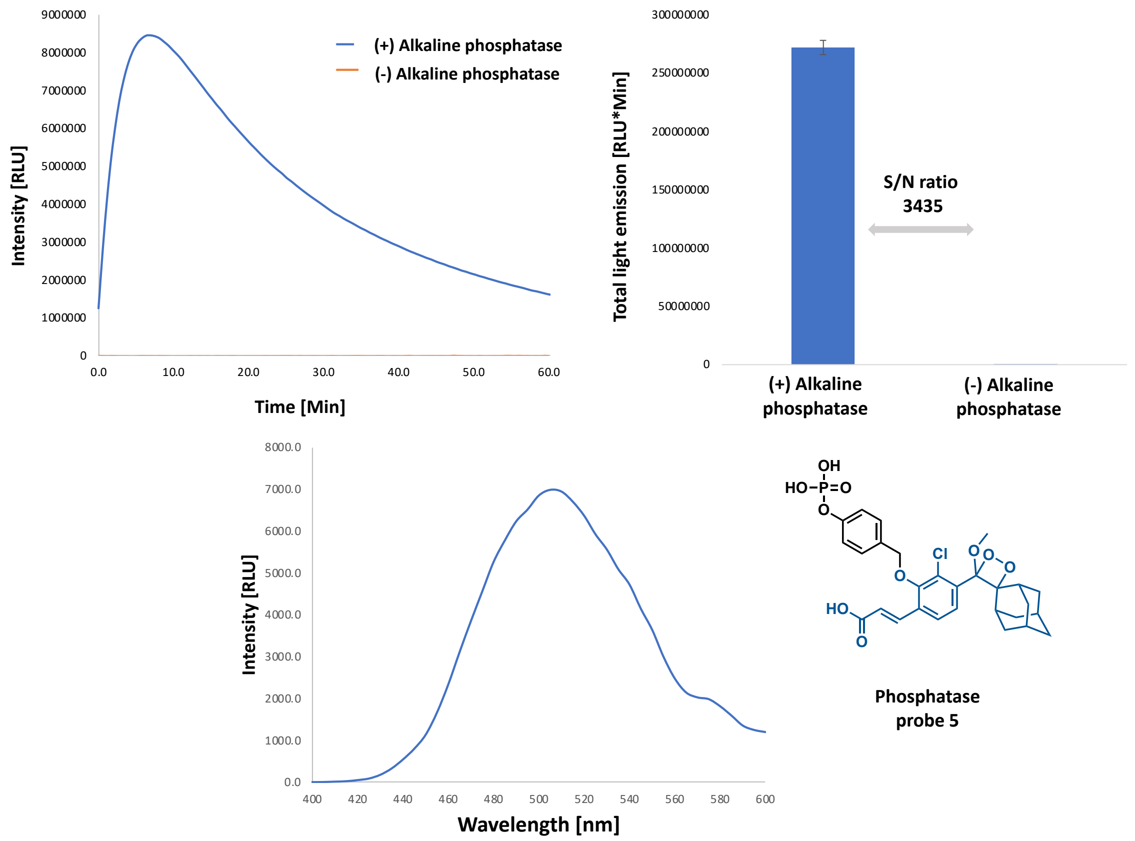

Figure S1. Measurement of chemiluminescent **phosphatase probe 5**. Kinetic profile (left) and total light emission (right) of the **phosphatase probe 5** [10 μM] in the presence and absence of alkaline phosphatase [1 U/mL] in PBS (pH 7.4), 0.1% DMSO, 37°C. Chemiluminescent emission spectra (bottom) of the **phosphatase probe 5** [10 μM] in the presence of alkaline phosphatase [1 U/mL] in PBS (pH 7.4), 0.1% DMSO, 37°C.

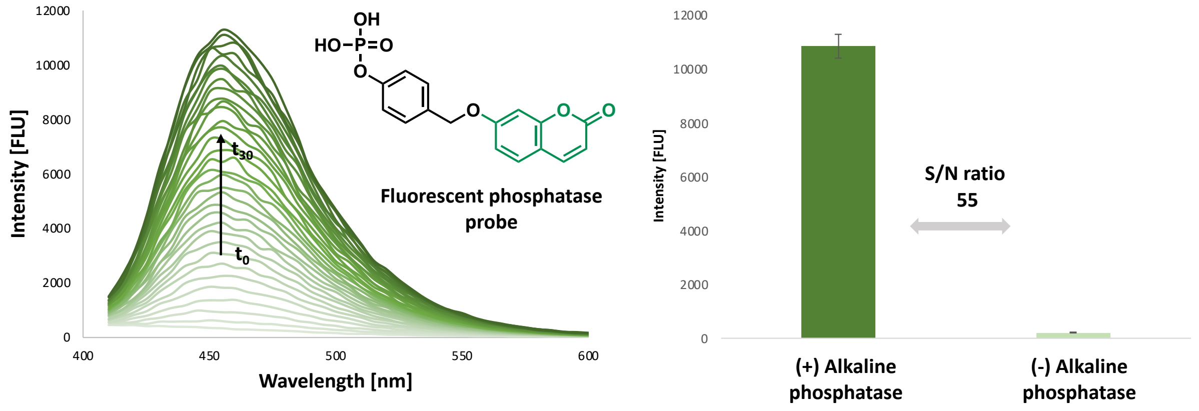

Figure S2. Measurement of **fluorescent phosphatase probe** (compound 17). Emission spectra (left) total light emission (right) of the **fluorescent phosphatase probe** (compound 17) [10 μM] in the presence and absence of alkaline phosphatase [1 U/mL] in PBS (pH 7.4), 0.1% DMSO, 37°C. FLU – fluorescence unit.

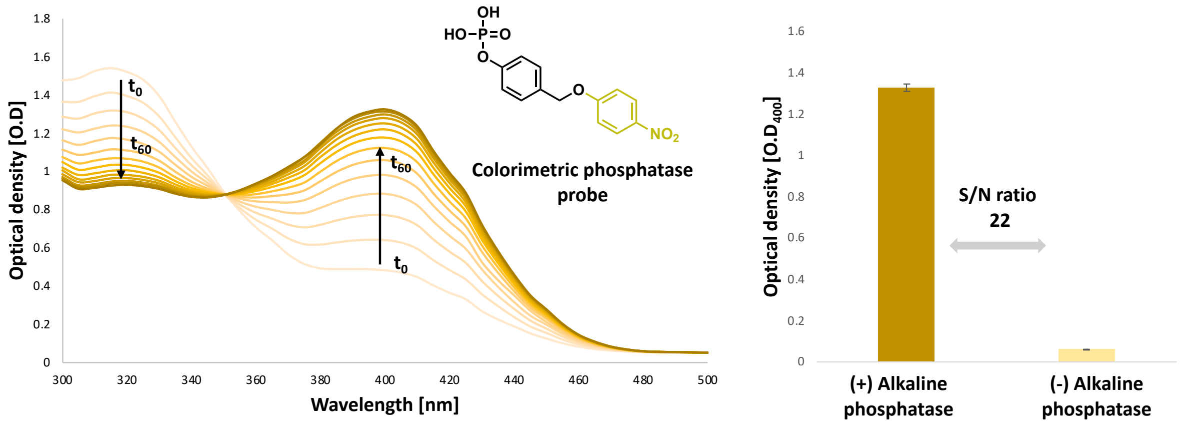

Figure S3. Measurement of **colorimetric phosphatase probe** (compound 19). Absorbance kinetic profile (left) and maximum absorbance wavelength (400nm) (right) of the **colorimetric phosphatase probe** (compound 19) [100 μM] in the presence and absence of alkaline phosphatase [1 U/mL] in PBS (pH 7.4), 0.1% DMSO, 37°C.

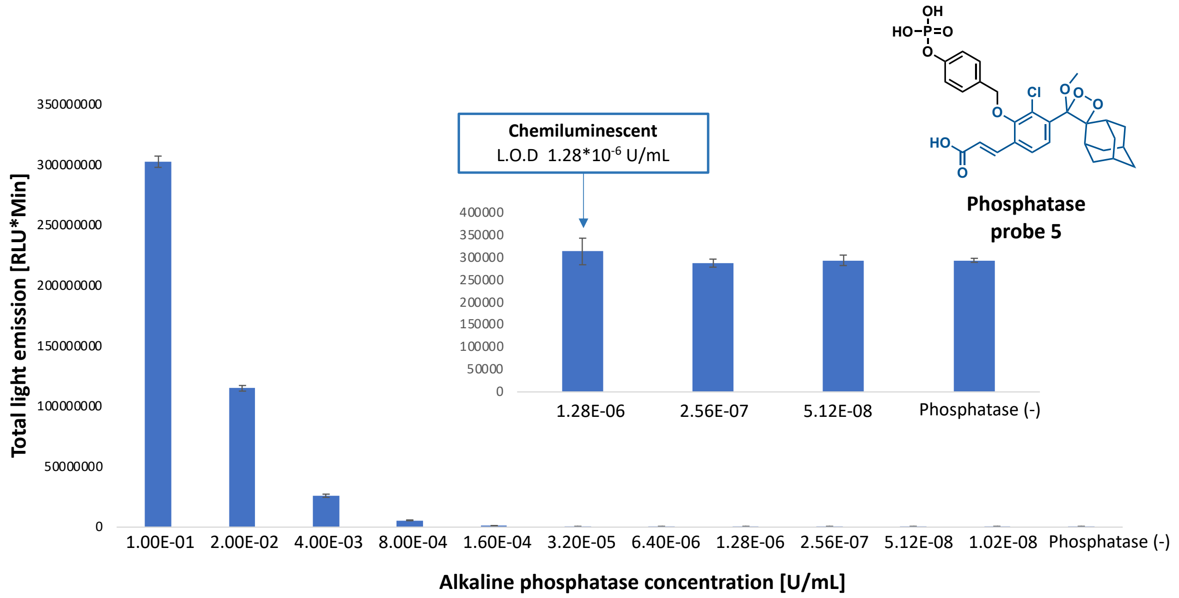

Figure S4. Total light emission of the chemiluminescent **phosphatase probe 5** [10 μM] in the presence and absence of various concentrations of alkaline phosphatase [$1.02*10^{-8}$ U/mL – 0.1 U/mL] in PBS (pH 7.4), 0.1% DMSO, 37°C. L.O.D was defined as control ± 3SD (standard deviation).

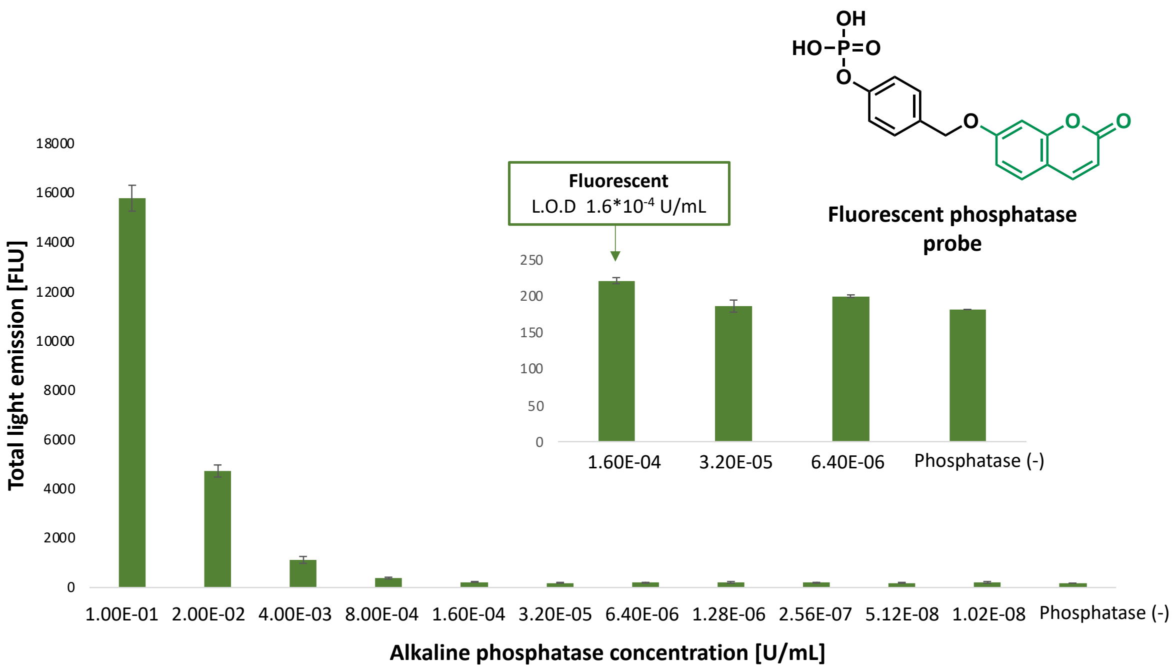

Figure S5. Total light emission of the **fluorescent phosphatase probe** (compound 17) [10 μM] in the presence and absence of various concentrations of alkaline phosphatase [1.02*10^{-8} U/mL – 0.1 U/mL] in PBS (pH 7.4), 0.1% DMSO, 37°C. L.O.D was defined as control ± 3SD (standard deviation). FLU – fluorescent unit.

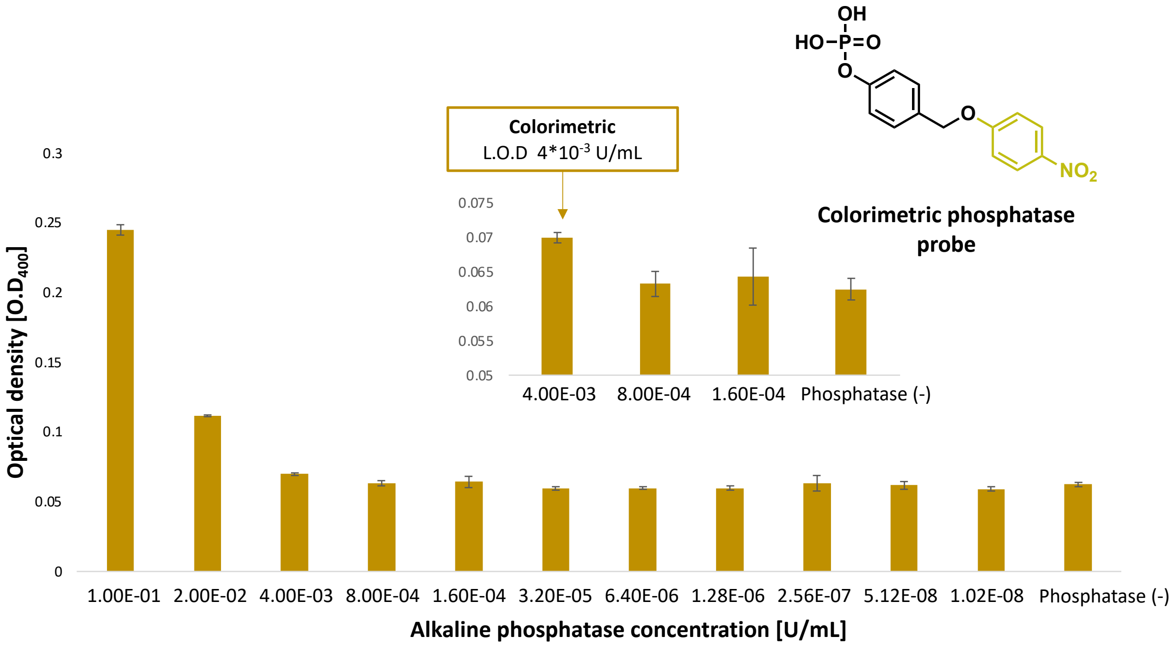

Figure S6. Absorbance values at 400 nm of the **colorimetric phosphatase probe** (compound 19) [10 μM] in the presence and absence of various concentrations of alkaline phosphatase [1.02*10^{-8} U/mL – 0.1 U/mL] in PBS (pH 7.4), 0.1% DMSO, 37°C. L.O.D was defined as control ± 3SD (standard deviation).

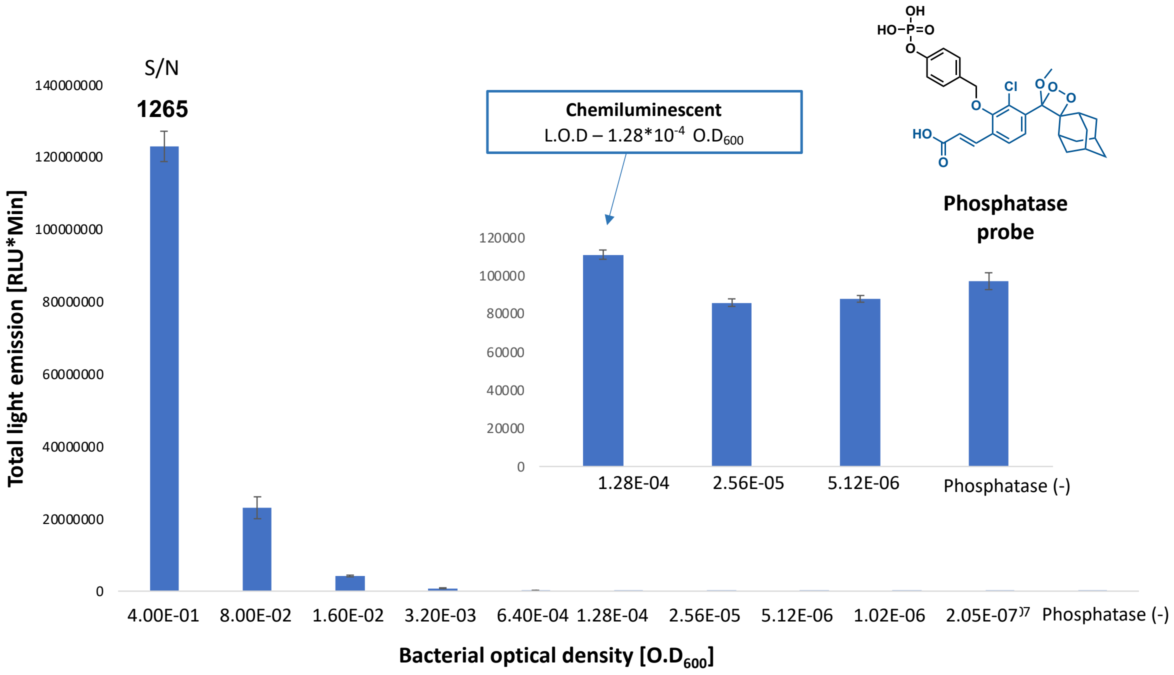

Figure S7. Total light emission of the chemiluminescent **phosphatase probe 5** [10 μM] in the presence and absence of various bacterial optical density of *S. aureus* ATCC 29213 [2.05*10-7 OD_{600} – 0.4 OD_{600}] in PBS (pH 7.4), 0.1% DMSO, 37°C. L.O.D was defined as control ± 3SD (standard deviation).

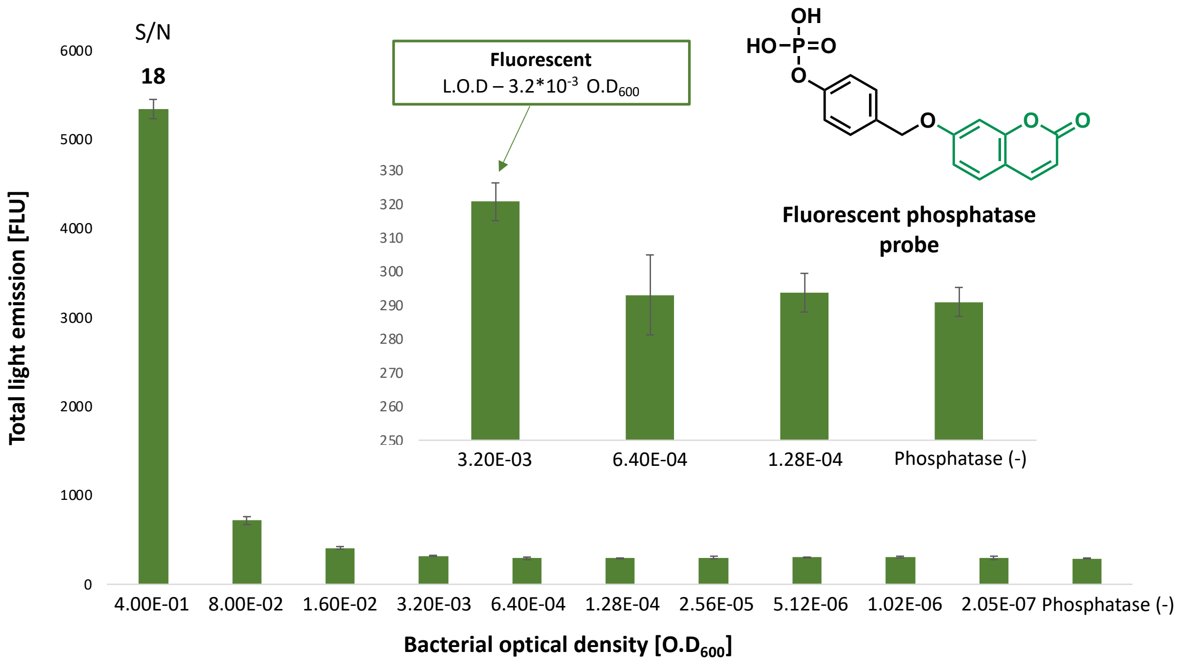

Figure S8. Total light emission of the **fluorescent phosphatase probe** (compound 17) [10 μM] in the presence and absence of various bacterial optical density of *S. aureus* ATCC 29213 [2.05*10-7 OD_{600} – 0.4 OD_{600}] in PBS (pH 7.4), 0.1% DMSO, 37°C. L.O.D was defined as control ± 3SD (standard deviation). FLU - fluorescence unit.

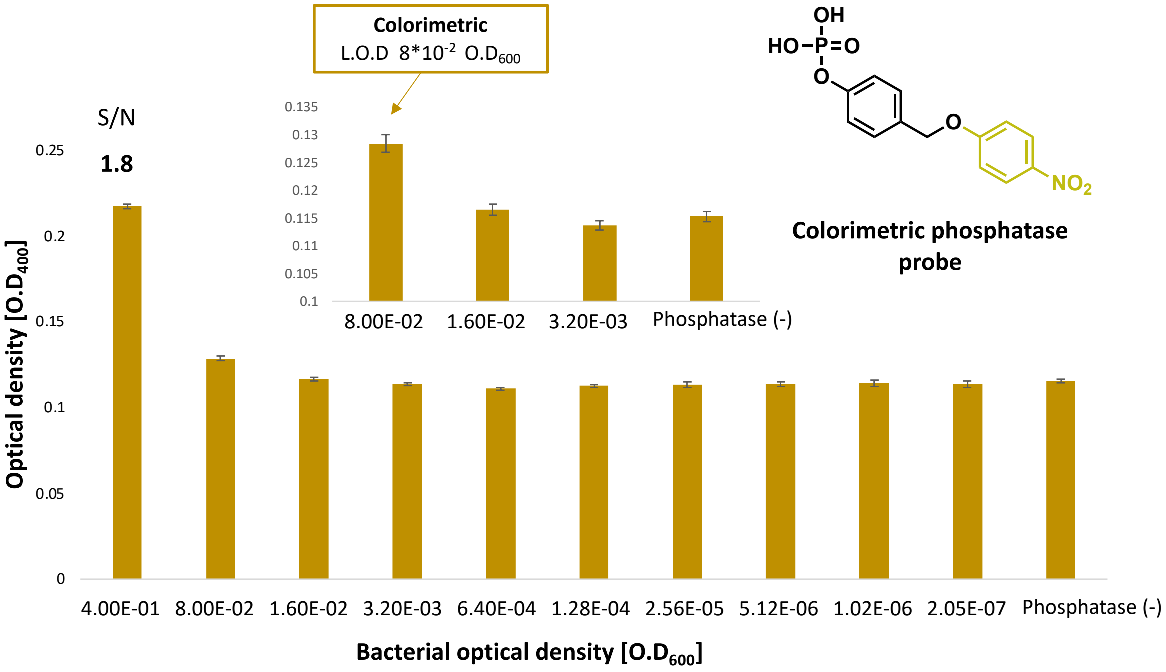

Figure S9. Absorbance values at 400 nm of the **colorimetric phosphatase probe** (compound 19) [10 μM] in the presence and absence of various bacterial optical density of *S. aureus* ATCC 29213 [2.05*10^{-7} OD_{600} – 0.4 OD_{600}] in PBS (pH 7.4), 0.1% DMSO, 37°C. L.O.D was defined as control ± 3SD (standard deviation).

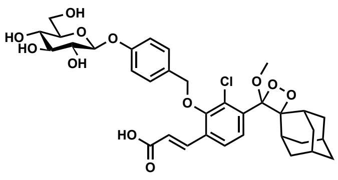

β-Glucosidase probe 1

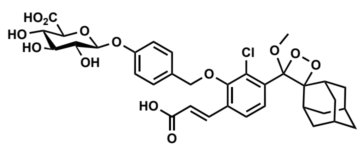

β-Glucuronidase probe 2

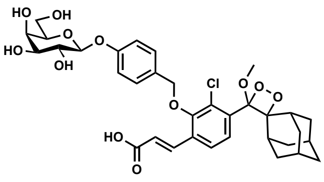

β-Galactosidase probe 3

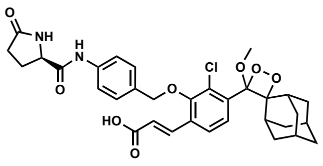

Pyroglutamyl aminopeptidase probe 4

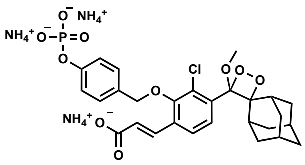

Phosphatase probe 5

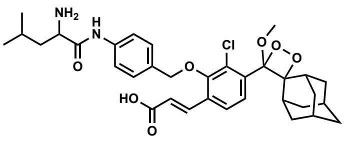

Leucine aminopeptidase probe 6

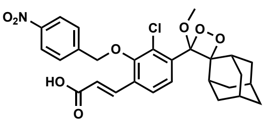

Nitroreductase probe 7

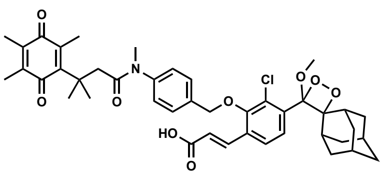

NAD(P)H quinone oxidoreductase 1 probe 8

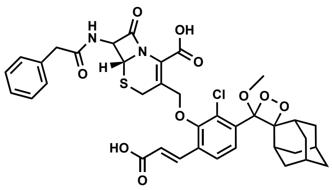

β-Lactamase probe 9

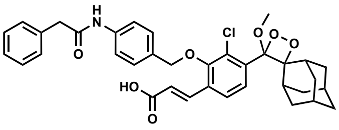

Penicillin-G amidase probe 10

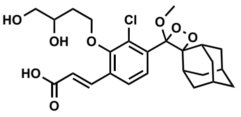

Periodate oxidative-cleavage probe 11

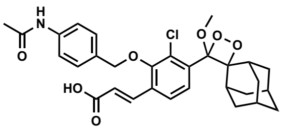

N-Acetyl hydrolase probe 12

Figure S10. Structure of the 12 chemiluminescent probes.

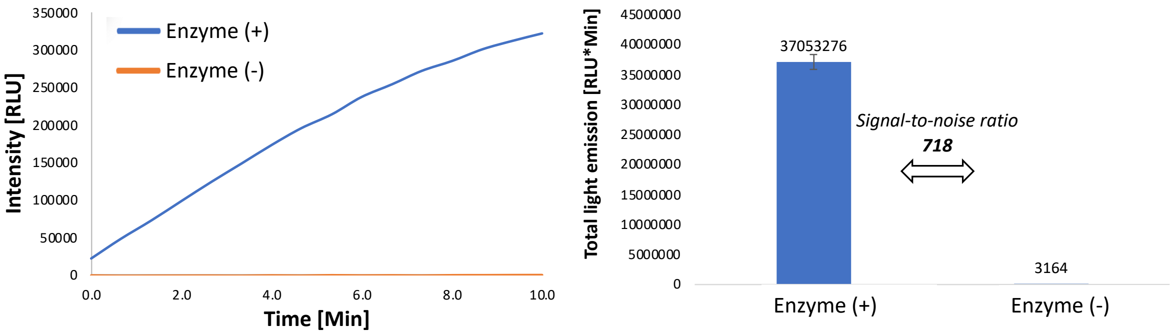

Figure S11. Chemiluminescence kinetic profile (Left) and total light emission (Right) ***β*-glucosidase probe 1** [10μM] in PBS (pH 7.4), 0.1% DMSO, 37°C with and without *β*-glucosidase from almonds [10 U/mL], purchased from Sigma-Aldrich. *S/N ratio is defined as the ratio between the total light emitted during 10 minutes with and without *β*-glucosidase.

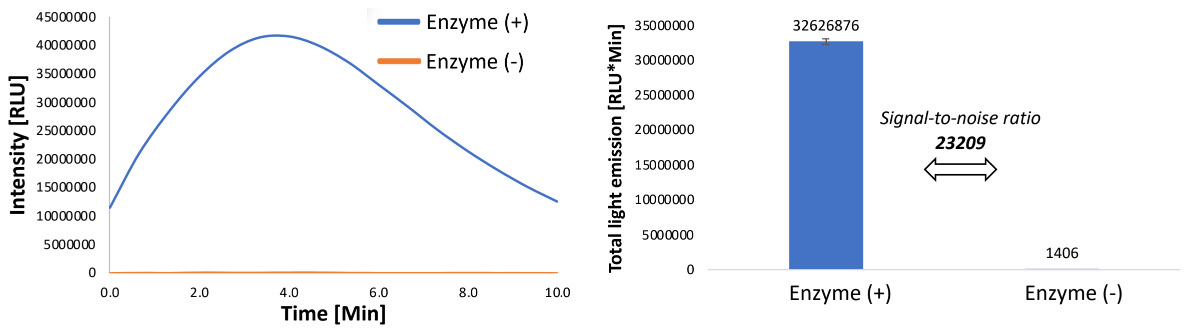

Figure S12. Chemiluminescence kinetic profile (Left) and total light emission (Right) of the ***β*-glucuronidase probe 2** [10μM] in PBS (pH 7.4), 0.1% DMSO, 37°C with and without *β*-glucuronidase from *E. coli* [1 U/mL], purchased from Sigma-Aldrich. *S/N ratio is defined as the ratio between the total light emitted during 10 minutes with and without *β*-glucuronidase.

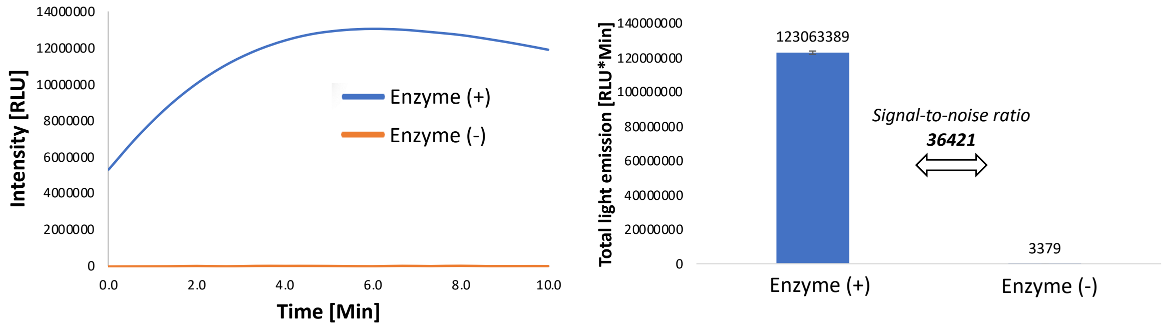

Figure S13. Chemiluminescence kinetic profile (Left) and total light emission (Right) of the ***β*-galactosidase probe 3** [10μM] in PBS (pH 7.4), 0.1% DMSO, 37°C with and without *β*-galactosidase from *E. coli* [1 U/mL], purchased from Sigma-Aldrich. *S/N ratio is defined as the ratio between the total light emitted during 10 minutes with and without *β*-galactosidase.

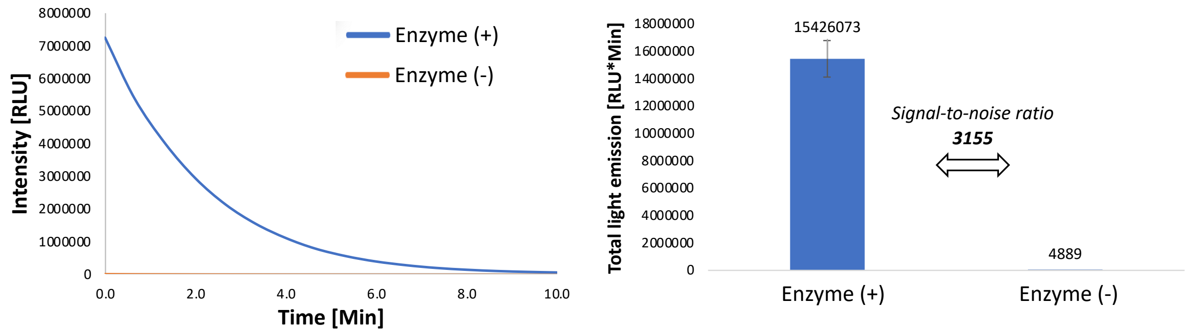

Figure S14. Chemiluminescence kinetic profile (Left) and total light emission (Right) of the **pyroglutamyl-peptidase I probe 4** [10μM] in PBS (pH 7.4), 0.1% DMSO, 37°C with and without pyroglutamyl-peptidase I from *E. coli* [0.05 mg/mL], purchased from R&D systems. *S/N ratio is defined as the ratio between the total light emitted during 10 minutes with and without pyroglutamyl-peptidase I.

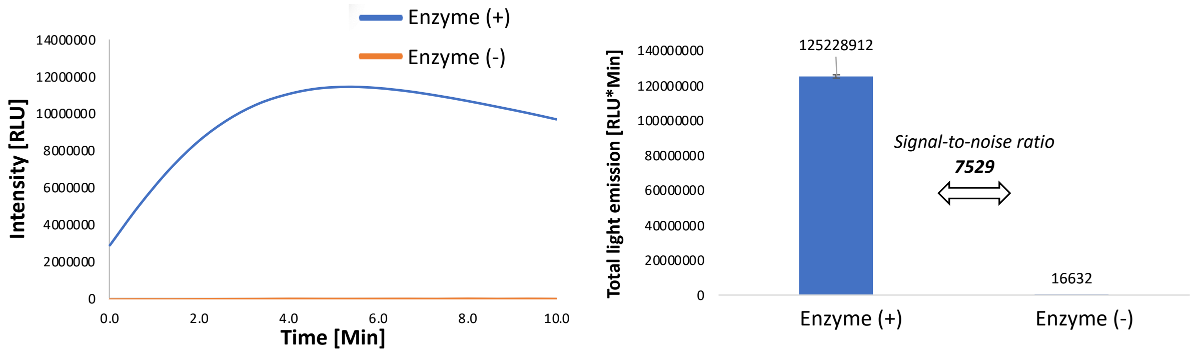

Figure S15. Chemiluminescence kinetic profile (Left) and total light emission (Right) of the **phosphatase probe 5** [10μM] in PBS (pH 7.4), 0.1% DMSO, 37°C with and without alkaline phosphatase from bovine intestinal mucosa [1 U/mL], purchased from Sigma-Aldrich. *S/N ratio is defined as the ratio between the total light emitted during 10 minutes with and without alkaline phosphatase.

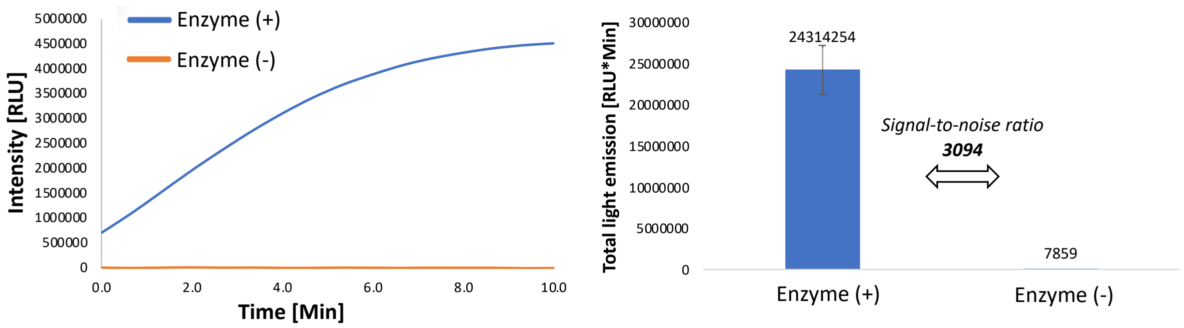

Figure S16. Chemiluminescence kinetic profile (Left) and total light emission (Right) of the **leucine probe 6** [10μM] in PBS (pH 7.4), 0.1% DMSO, 37°C with and without aminopeptidase-M from Porcine Kidney [1 U/mL], purchased from Merck Millipore. *S/N ratio is defined as the ratio between the total light emitted during 10 minutes with and without aminopeptidase-M.

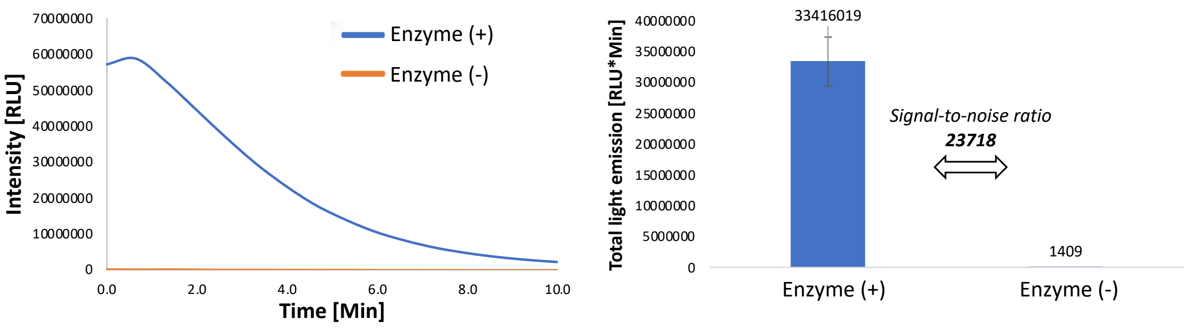

Figure S17. Chemiluminescence kinetic profile (Left) and total light emission (Right) of the **nitroreductase probe 7** [10μM] in PBS (pH 7.4), 0.1% DMSO, 37°C with and without nitroreductase from *E. coli* [1 mg/mL, 100μM NADH], purchased from Sigma-Aldrich. *S/N ratio is defined as the ratio between the total light emitted during 10 minutes with and without nitroreductase.

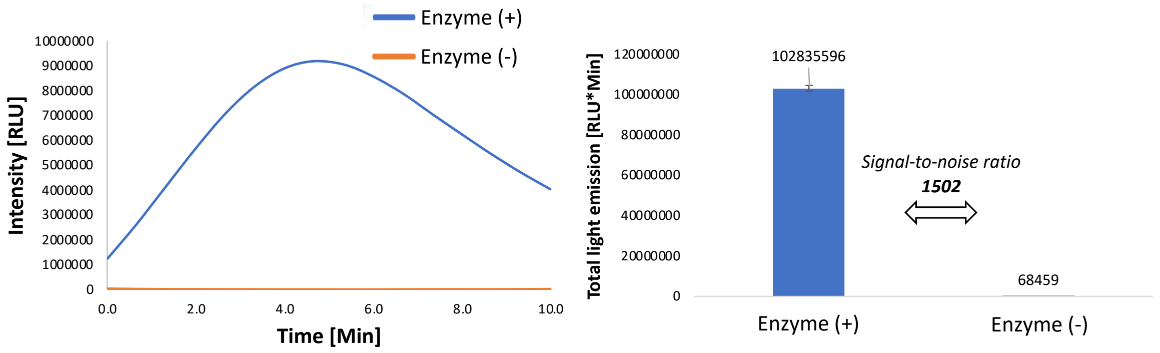

Figure S18. Chemiluminescence kinetic profile (Left) and total light emission (Right) of the **NAD(P)H quinone oxidoreductase 1 probe 8** [10μM] in PBS (pH 7.4), 0.1% DMSO, 37°C with and without NAD(P)H quinone oxidoreductase 1 from *E. coli* [0.8 mg/mL, 100μM NADH], purchased from Sigma-Aldrich.*S/N ratio is defined as the ratio between the total light emitted during 10 minutes with and without NAD(P)H quinone oxidoreductase 1.

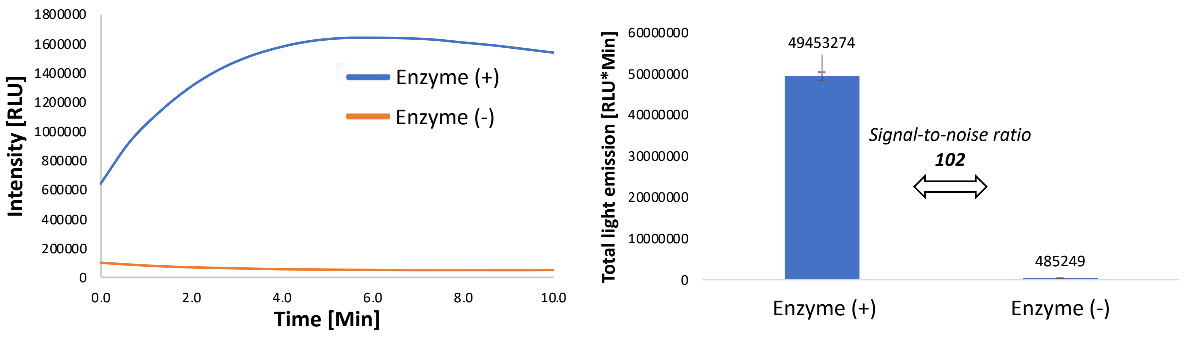

Figure S19. Chemiluminescence kinetic profile (Left) and total light emission (Right) of the ***β*-lactamase probe 9** [10μM] in PBS (pH 7.4), 0.1% DMSO, 37°C with and without *β*-lactamase from *E. coli* [2 U/mL], purchased from Biosynth.*S/N ratio is defined as the ratio between the total light emitted during 10 minutes with and without *β*-lactamase.

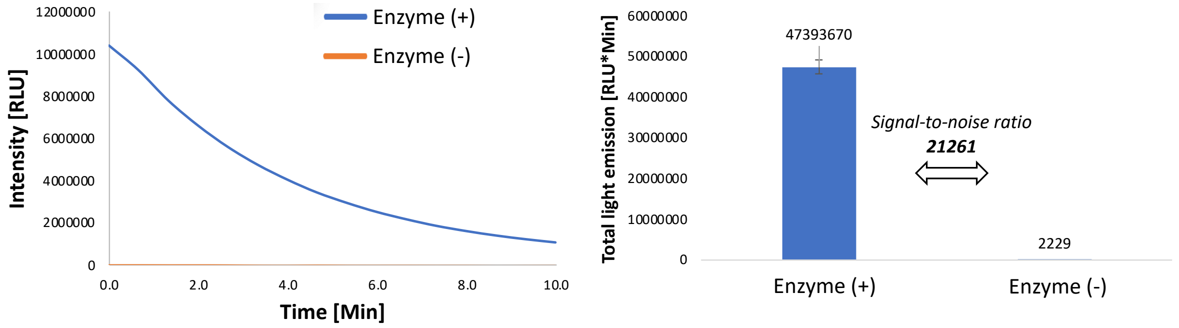

Figure S20. Chemiluminescence kinetic profile (Left) and total light emission (Right) of the **penicillin G-amidase probe 10** [10μM] in PBS (pH 7.4), 0.1% DMSO, 37°C with and without penicillin G-amidase from *E. coli* [1 U/mL], purchased from Sigma-Aldrich. *S/N ratio is defined as the ratio between the total light emitted during 10 minutes with and without penicillin G-amidase.

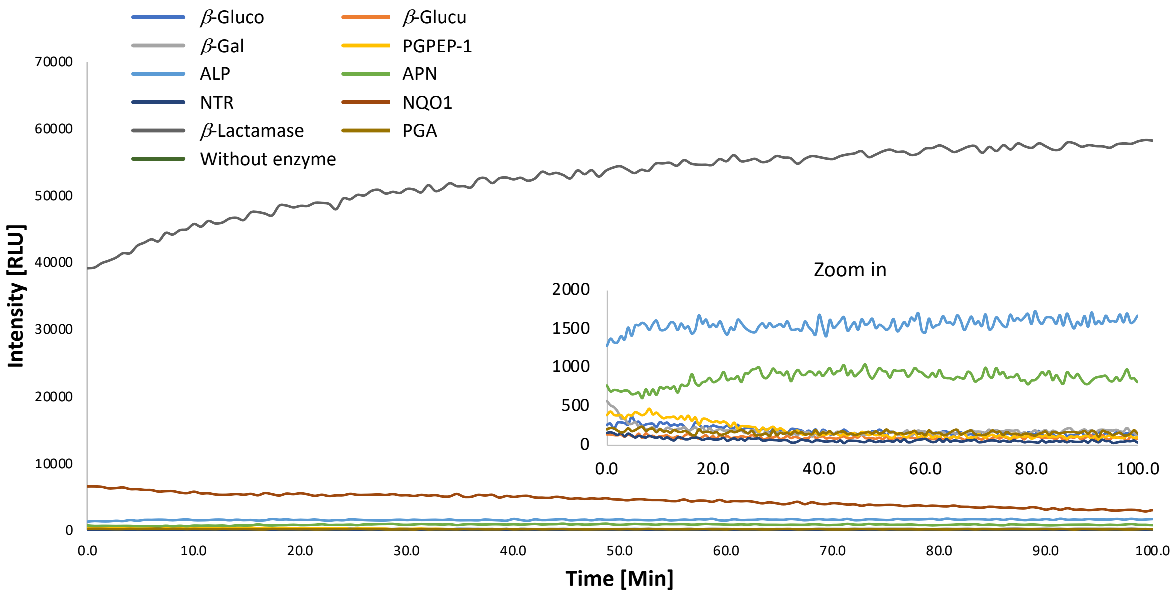

Figure S21. Background evaluation. Chemiluminescence kinetic profile of the chemiluminescent array [10µM] in PBS (pH 7.4), 0.1% DMSO, 37°C without enzymes.

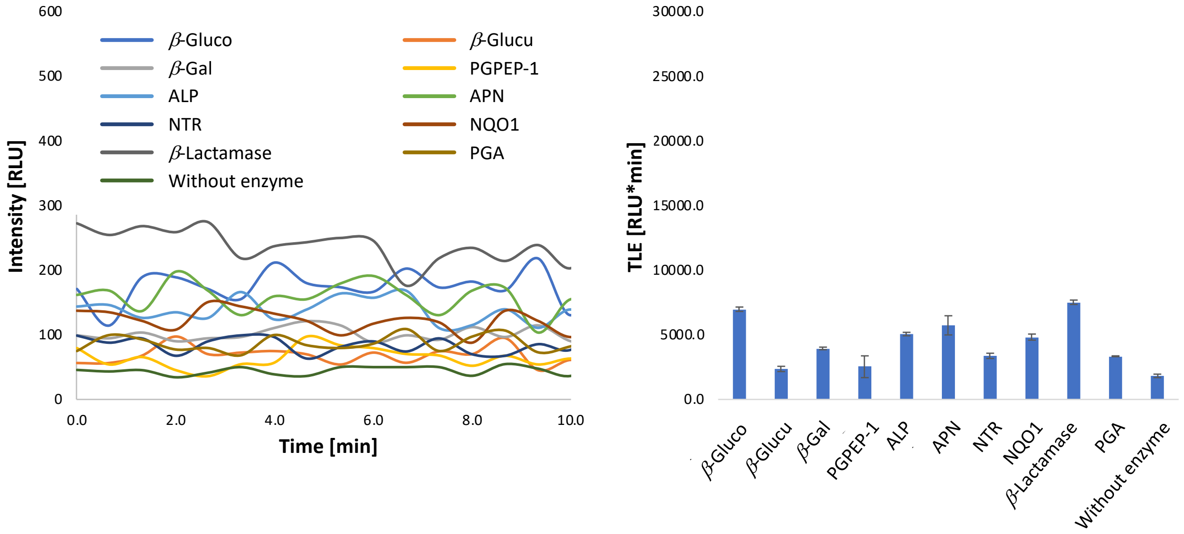

Figure S22. Periodate oxidative-cleavage probe 11 in the presence of 10 commercially available recombinant enzymes. The chemiluminescent intensity of each probe (left) and the total emitted light during 10 minutes (right) [10 µM in PBS (pH 7.4), 0.1% DMSO, 37°C] was measured in the presence of each of the following commercially available recombinant enzymes: β-glucosidase (from almonds)[10 U/mL], β-glucuronidase from *E. coli* [1 U/mL], β-galactosidase from *E. coli* [1 U/mL], pyroglutamyl-peptidase I from *E. coli* [0.05 mg/mL], alkaline phosphatase from bovine intestinal mucosa [1 U/mL], aminopeptidase-M from porcine kidney [1 U/mL], nitroreductase from *E. coli* [1 mg/mL, 100µM NADH], NADH NQO1 from *E. coli* [0.8 mg/mL, 100µM NADH], β-lactamase from *E. coli* [2 U/mL], penicillin G-amidase from *E. coli* [1 U/mL].

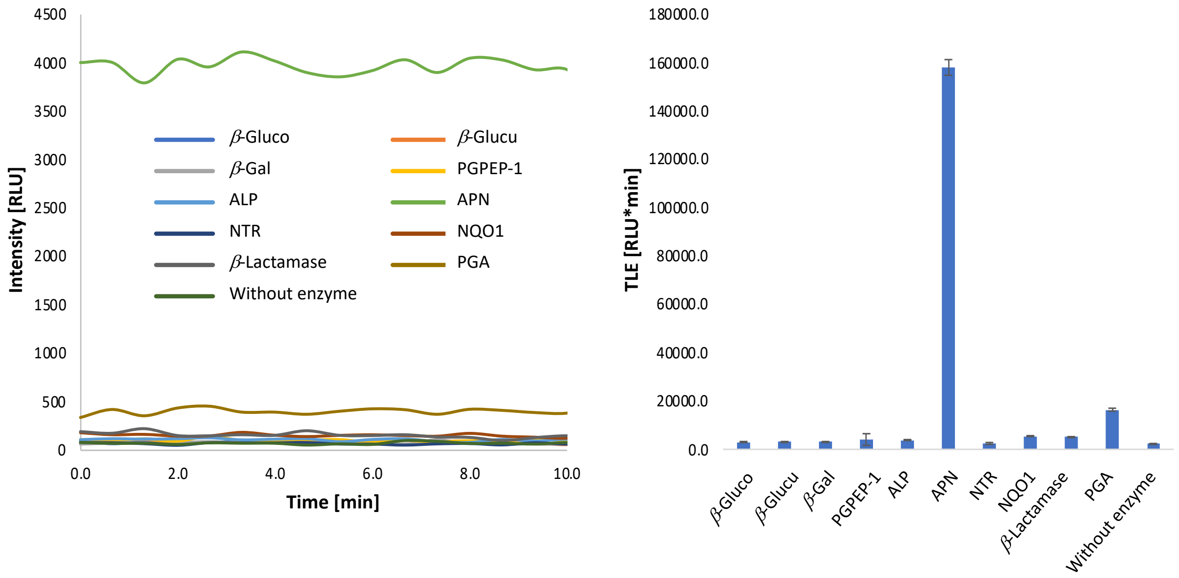

Figure S23. N-Acetyl hydrolase probe 12 in the presence of 10 commercially available recombinant enzymes. The chemiluminescent intensity of each probe (left), and the total emitted light during 10 minutes (right). A concentration of 10 μM of each probe in PBS (pH 7.4), 0.1% DMSO, 37°C was measured in the presence of each of the following commercially available recombinant enzymes: *β*-glucosidase from almonds [10 U/mL], *β*-glucuronidase from *E. coli* [1 U/mL], *β*-galactosidase from *E. coli* [1 U/mL], pyroglutamyl-peptidase I from *E. coli* [0.05 mg/mL], alkaline phosphatase from bovine intestinal mucosa [1 U/mL], aminopeptidase-M (from Porcine Kidney [1 U/mL], nitroreductase from *E. coli* [1 mg/mL, 100μM NADH], NADH NQO1 from *E. coli* [0.8 mg/mL, 100μM NADH], *β*-lactamase from *E. coli* [2 U/mL], penicillin G-amidase from *E. coli* [1 U/mL].

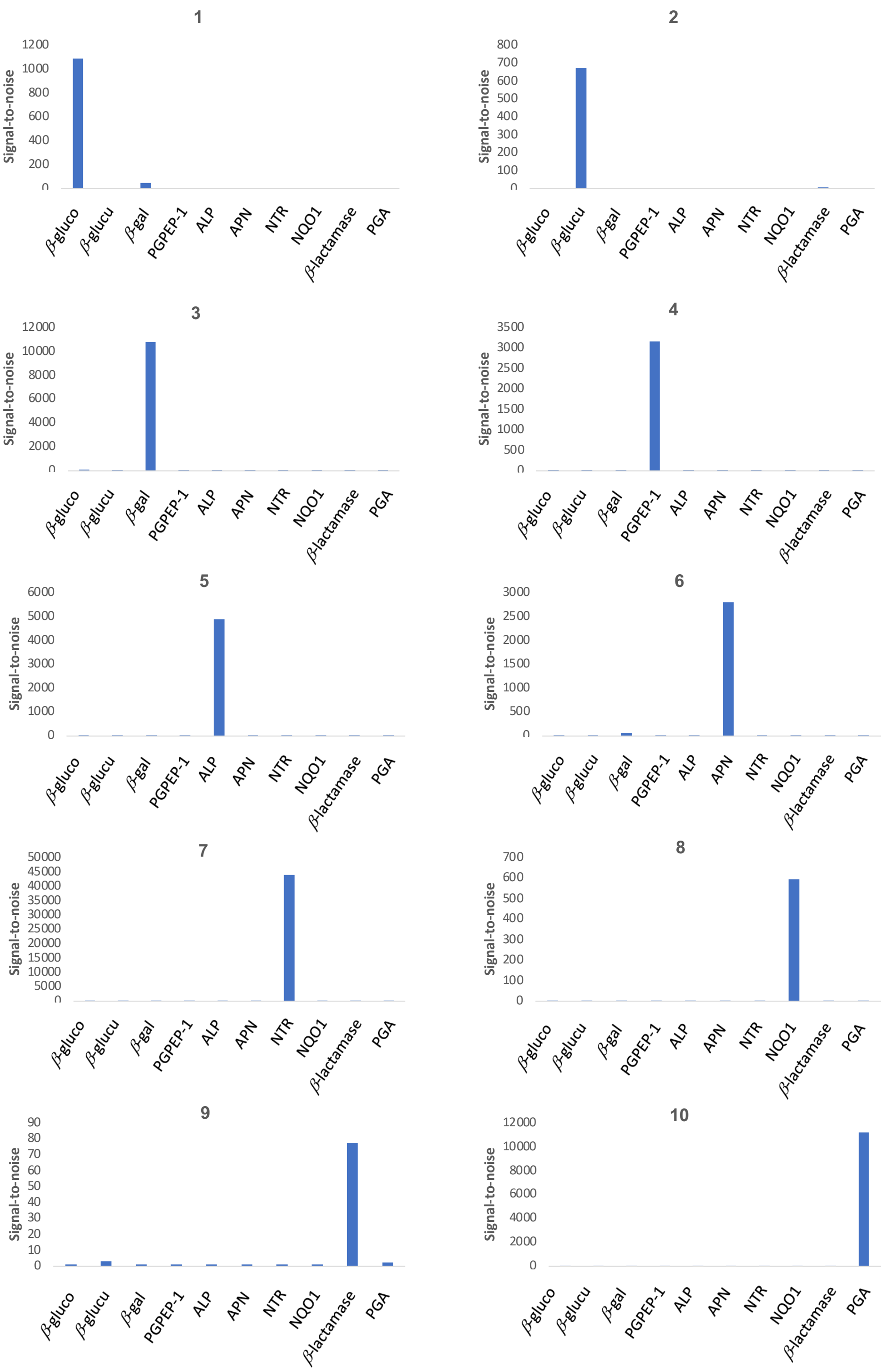

Figure S24. The selectivity of each of the chemiluminescent probes was evaluated in the presence of 10 commercially available recombinant enzymes. The chemiluminescent intensity of each probe [10 μM in PBS (7.4 pH), 0.1% DMSO, 37°C] was measured in the presence of each of the following commercially available recombinant enzymes: *β*-glucosidase (from almonds)[10 U/mL], *β*-glucuronidase from *E. coli* [1 U/mL], *β*-galactosidase from *E. coli* [1 U/mL], pyroglutamyl-peptidase I from *E. coli* [0.05 mg/mL], alkaline phosphatase from bovine intestinal mucosa [1 U/mL], aminopeptidase-M from Porcine Kidney [1 U/mL], nitroreductase from *E. coli* [1 mg/mL, 100μM NADH], NADH NQO1 from *E. coli* [0.8 mg/mL, 100μM NADH], *β*-lactamase from *E. coli* [2 U/mL], penicillin G-amidase from *E. coli* [1 U/mL].

Enzyme / Probe	β-Glucosidase	β-Glucuronidase	β-Galactosidase	Pyroglutamyl aminopeptidase	Phosphatase	Leucine aminopeptidase	Nitroreductase	NAD(P)H quinone oxidoreductase 1	β-Lactamase	Penicillin-G amidase
1	100.0	0.1	4.2	0.2	0.4	0.3	0.1	0.1	0.1	0.1
2	0.3	100.0	0.2	0.3	0.2	0.2	0.2	0.3	0.9	0.2
3	0.8	0.0	100.0	0.0	0.0	0.0	0.1	0.0	0.0	0.0
4	0.0	0.1	0.0	100.0	0.1	0.1	0.0	0.0	0.1	0.1
5	0.0	0.0	0.1	0.0	100.0	0.0	0.5	0.1	0.1	0.5
6	0.0	0.4	1.9	0.0	0.0	100.0	0.0	0.1	0.2	0.2
7	0.0	0.0	0.0	0.0	0.0	0.0	100.0	0.0	0.0	0.0
8	0.2	0.2	0.2	0.2	0.2	0.1	0.1	100.0	0.2	0.2
9	1.4	4.1	1.3	1.4	1.4	1.3	1.4	1.4	100.0	2.9
10	0.0	0.0	0.0	0.0	0.0	0.4	0.0	0.0	0.0	100.0

Figure S25. Selectivity evaluation results in a confusion matrix. Values represent the percentage of activation compared to the chemiluminescent probe with its complementary enzyme. Activation levels of 100% are highlighted in red, and activation levels between 1.5-5% are highlighted in yellow.

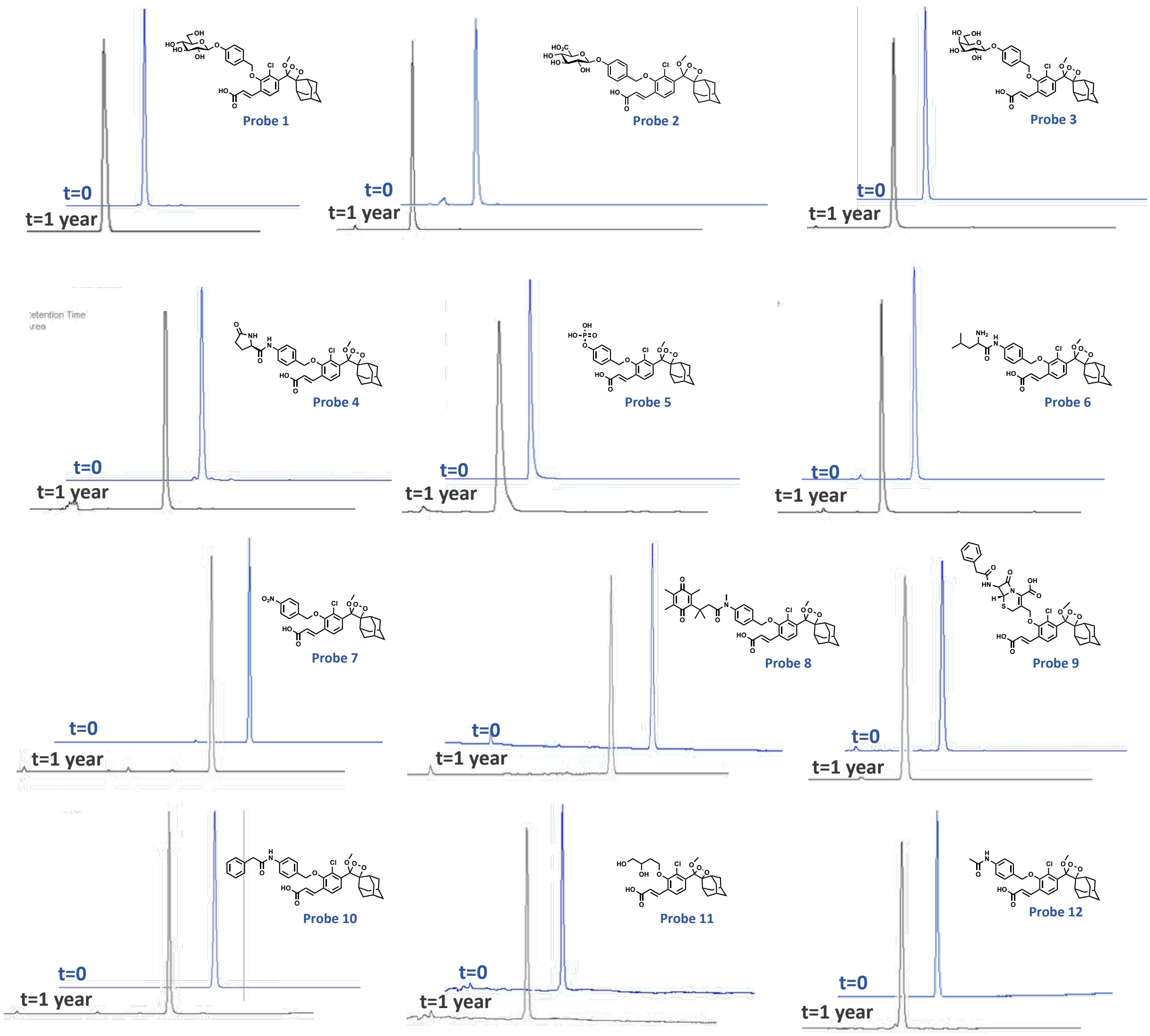

Figure S26. Probe stability information: RP-HPLC was employed to assess the stability of stock solutions of probes 1-12 in DMSO. The blue curve spectra represent the stock solution immediately after preparation (t=0), while the black spectra represent the probe stock solution after one year of storage at -20°C.

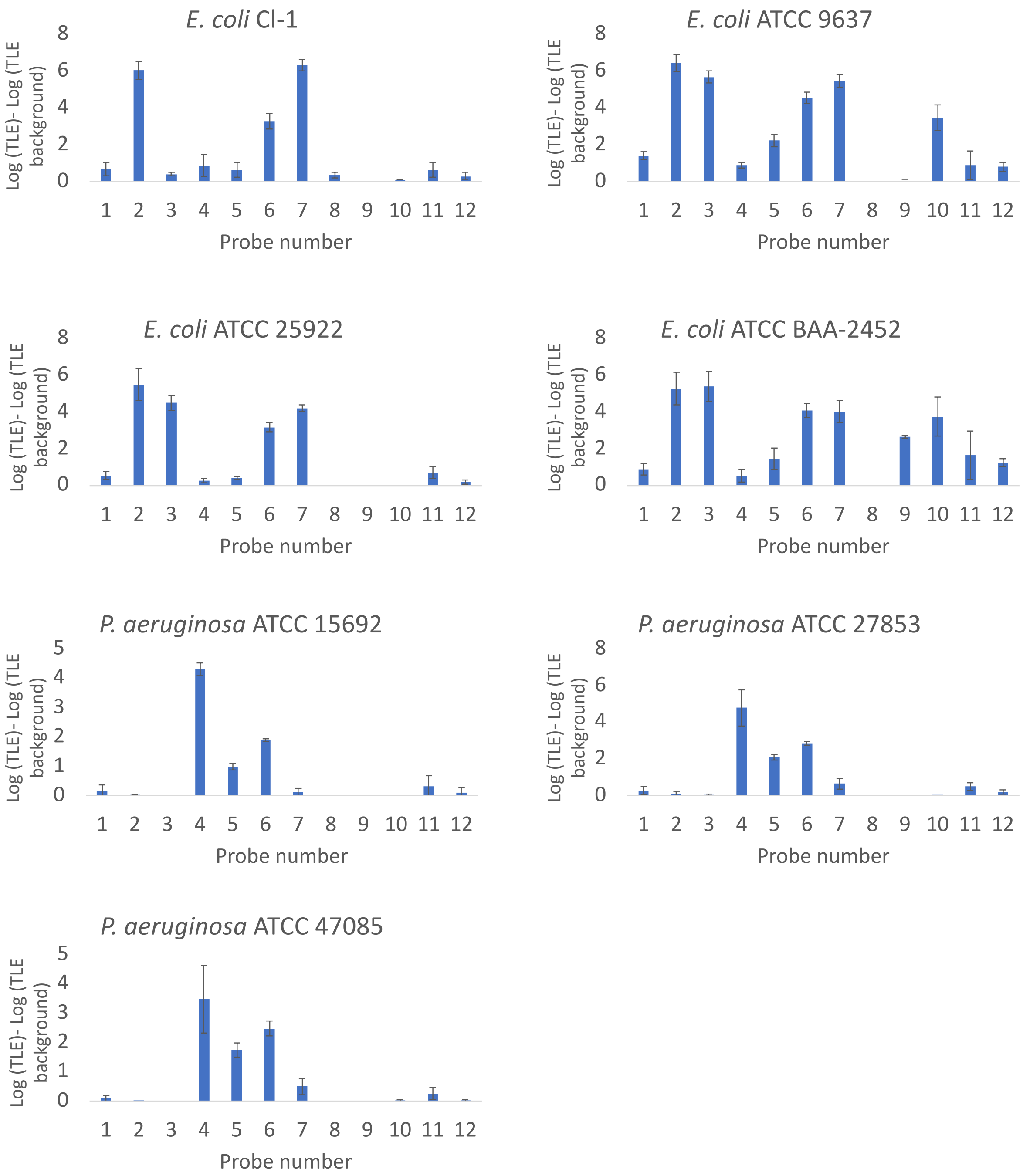

Figure S27. Log total light emission profiles of the chemiluminescent array with seven out of the 29 strains of bacteria, [OD_{600}= 0.4], in PBS (pH 7.4), 0.1% DMSO, 37°C. The background represents probe without bacteria.

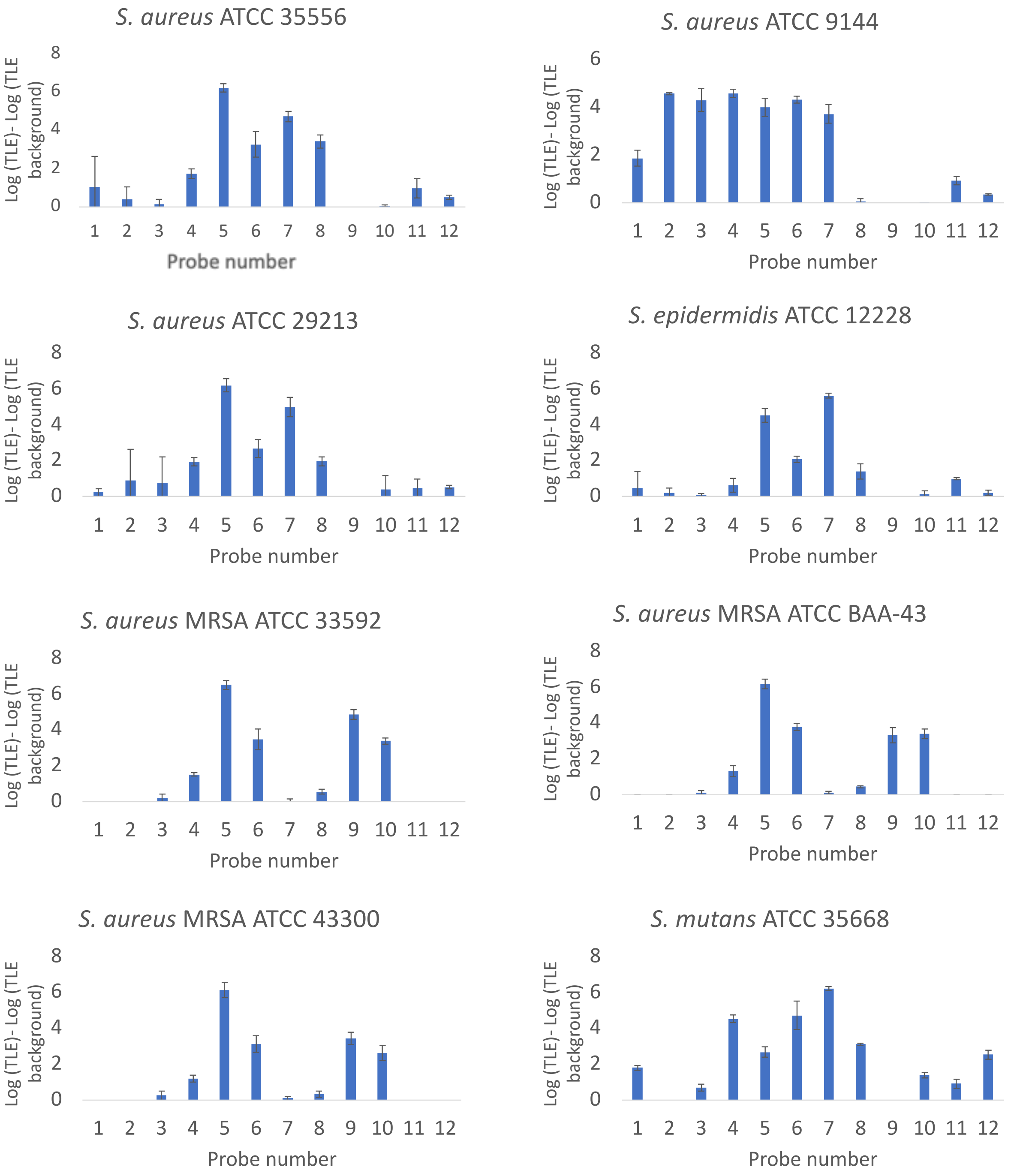

Figure S28. Log total light emission profiles of the chemiluminescent array with eight out of the 29 strains of bacteria, [OD_{600} 0.4], in PBS (pH 7.4), 0.1% DMSO, 37°C. The background represents probe without bacteria.

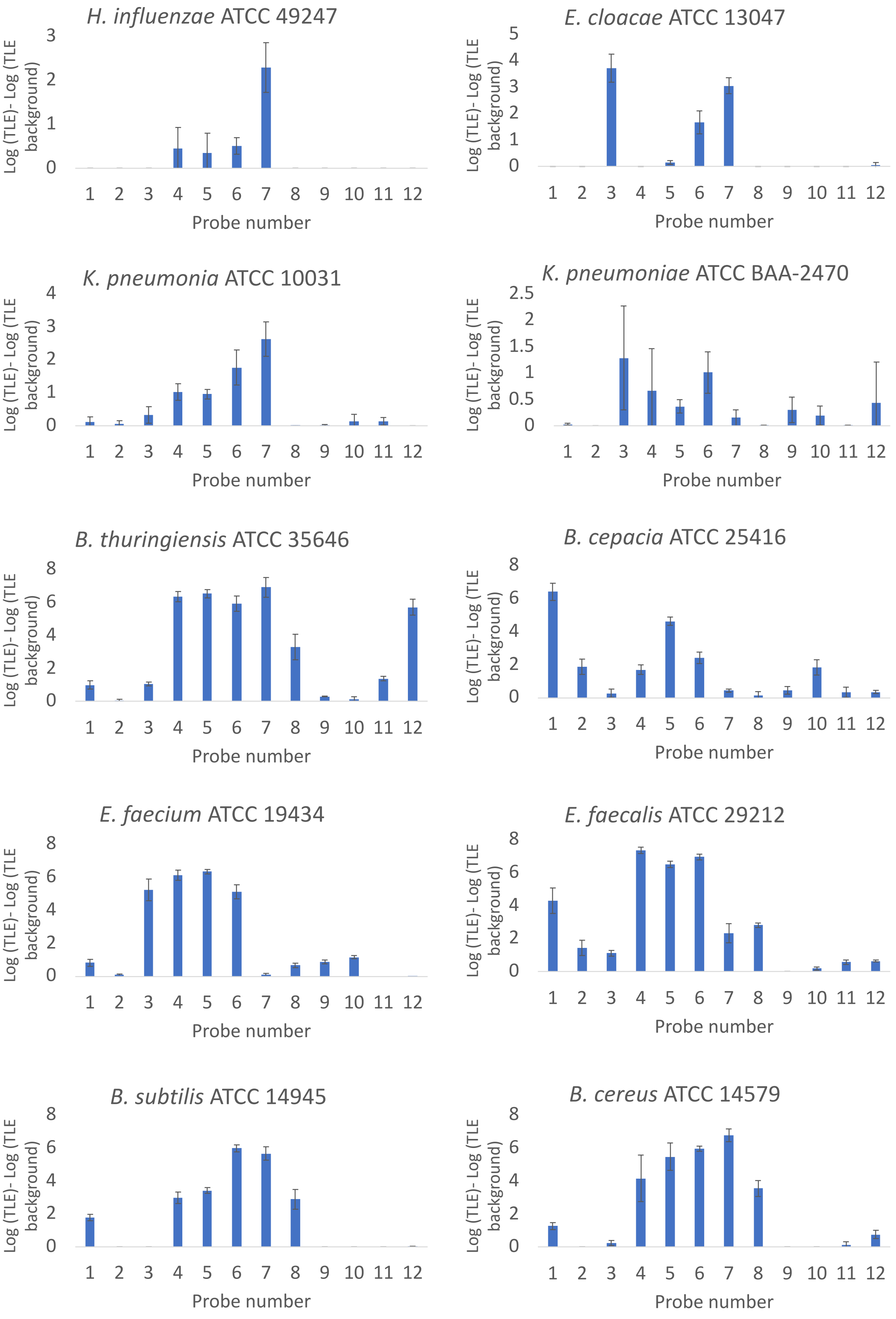

Figure S29. Log total light emission profiles of the chemiluminescent array with 10 out of the 29 strains of bacteria, [OD_{600}= 0.4], in PBS (pH 7.4), 0.1% DMSO, 37°C. The background represents probe without bacteria.

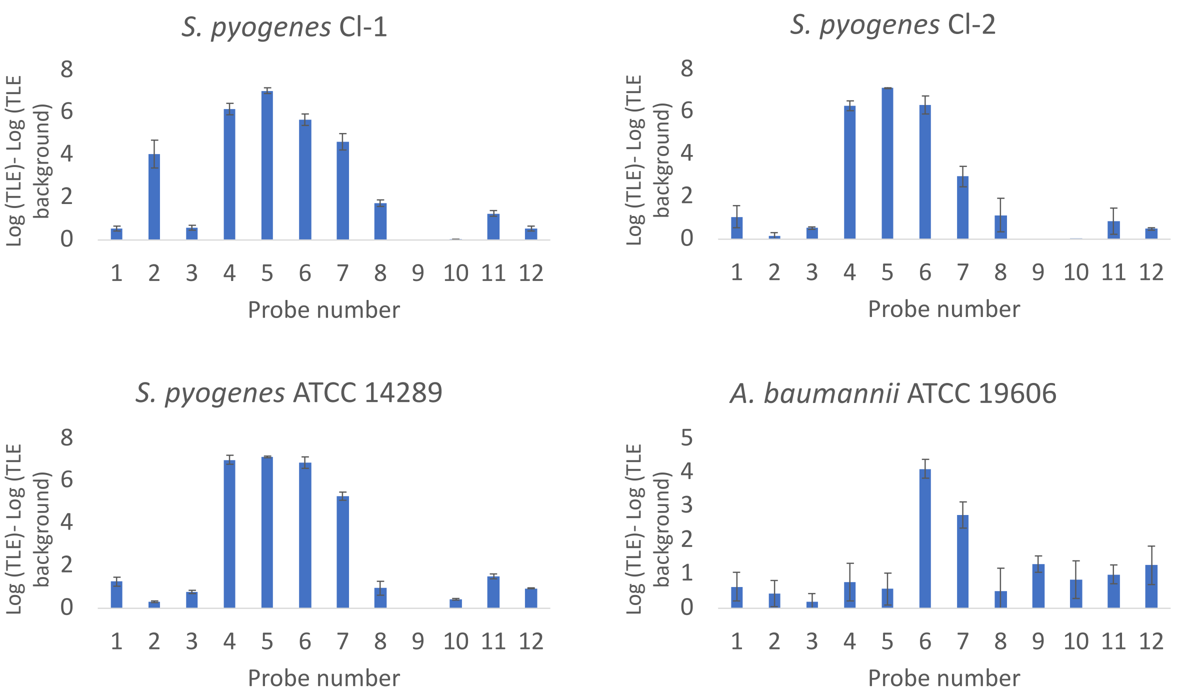

Figure S30. Log total light emission profiles of the chemiluminescent array with four out of the 29 strains of bacteria, [OD_{600}= 0.4], in PBS (pH 7.4), 0.1% DMSO, 37°C. The background represents probe without bacteria.

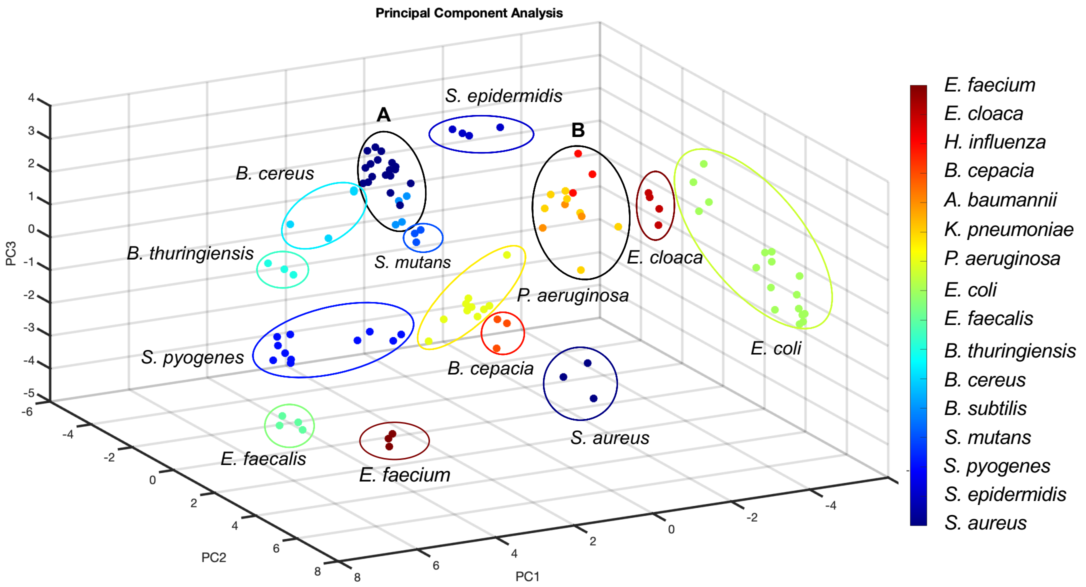

Figure S31. PCA differentiation between the species in the panel.

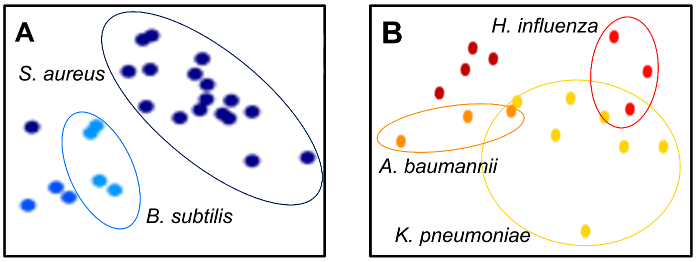

Figure S32. Magnification of the PCA results for **A** and **B** in the full PCA (Figure S31).

Probe Number	Variance [Log(TLE)-Log(Background)]
5	6.76
4	5.71
2	4.67
7	4.37
3	3.61
6	3.17
8	1.93
1	1.88
12	1.19
10	0.94
9	0.29
11	0.24

Figure S33. Probe variability. The variance in each probe's response was measured across all 29 bacterial strains in the panel. Initially, the mean for each bacterium's log (TLE)-log(background) of the three independent measurements was calculated for each probe. The standard deviation of the 29 values (representing 29 bacteria for each probe) was calculated. Finally, the variance was calculated as the squares of the standard deviation.

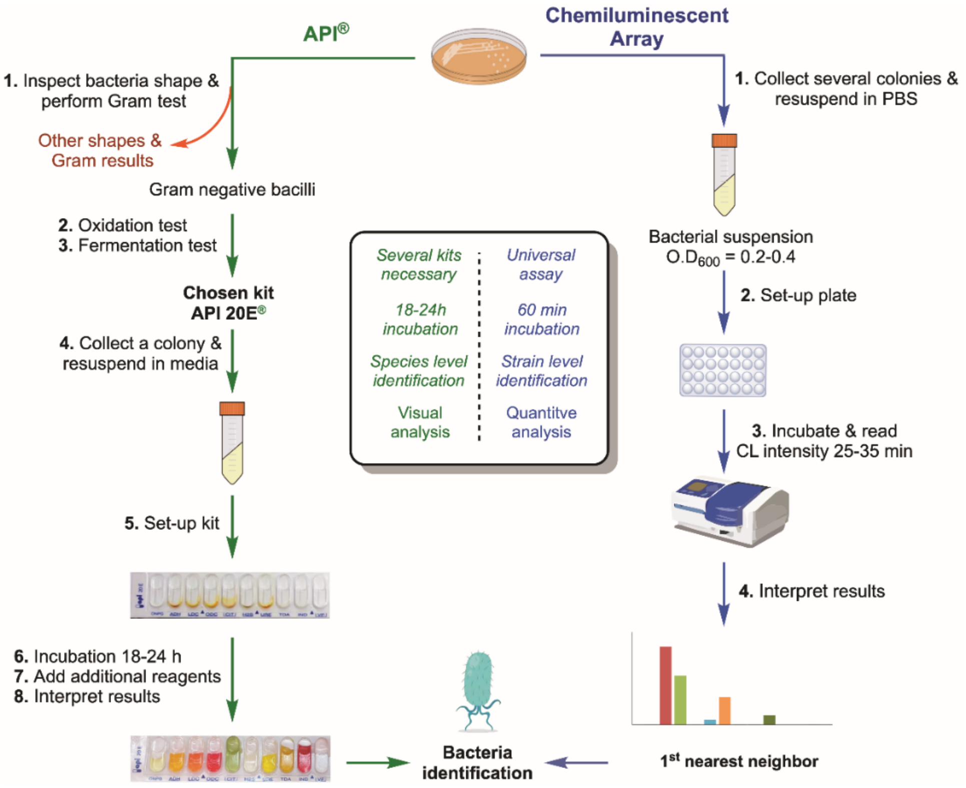

Figure S34. Schematic comparison between the commercially available bacterial identification method analytical profile index (API) and the chemiluminescent-based assay.

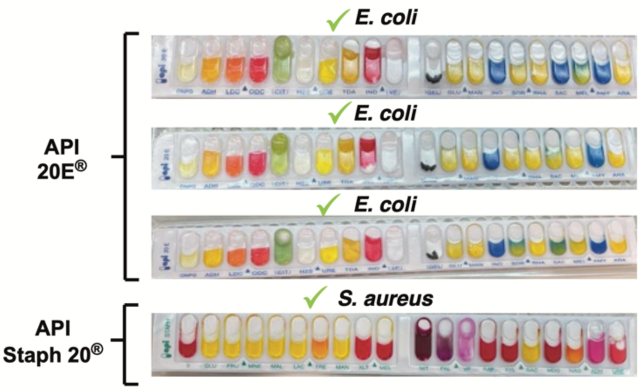

Figure S35. Images of the analytical profile index (API) results.

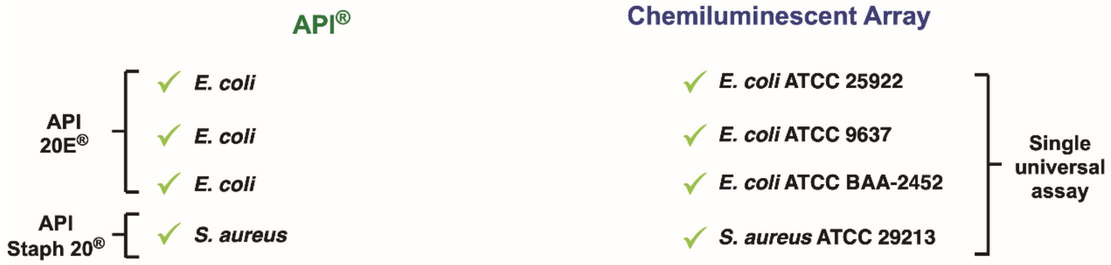

Figure S36. Comparison between the identification results achieved by the analytical profile index (API) and the chemiluminescent-based assay.

probe 1	probe 2	probe 3	probe 4	probe 5	probe 6	probe 7	probe 8	probe 9	probe 10	probe 11	probe 12	Test Accuracy (%)	Number of probes
	✓											100	1
✓	✓											100	2
✓			✓									97	2
	✓	✓										97	2
	✓		✓									100	2
	✓			✓								100	2
	✓				✓							97	2
	✓									✓		100	2
	✓										✓	100	2
	✓					✓						100	2
	✓						✓					100	2
	✓							✓				100	2
	✓								✓			100	2
			✓		✓							97	2
				✓		✓						97	2
				✓					✓			97	2
					✓			✓				97	2
						✓	✓					100	2
✓	✓	✓										100	3
✓	✓		✓									100	3
✓	✓			✓								100	3
✓	✓				✓							100	3
✓	✓									✓		100	3
✓	✓										✓	97	3
✓	✓					✓						100	3
✓	✓						✓					100	3
✓	✓							✓				100	3
✓	✓								✓			100	3
✓		✓	✓									97	3
✓		✓		✓								97	3

Figure S37. *E. coli* differentiation results. The first 30 combinations result in an accuracy higher than 95% for differentiating *E. coli* from other bacteria in the panel. Check-mark- probes indicate the probes combinations that were used in the 1-nearest neighbor analysis.

probe 1	probe 2	probe 3	probe 4	probe 5	probe 6	probe 7	probe 8	probe 9	probe 10	probe 11	probe 12	Test Accuracy (%)	Number of probes
				✓								100	1
						✓						97	1
✓				✓								100	2
✓					✓							100	2
✓						✓						97	2
	✓	✓										97	2
	✓			✓								100	2
	✓				✓							97	2
		✓	✓									100	2
		✓		✓								100	2
		✓				✓						97	2
		✓					✓					97	2
			✓	✓								97	2
			✓			✓						100	2
				✓	✓							100	2
				✓						✓		100	2
				✓							✓	100	2
				✓		✓						97	2
				✓			✓					100	2
				✓				✓				100	2
				✓					✓			100	2
					✓	✓						100	2
					✓		✓					97	2
						✓		✓				97	2
✓	✓	✓										97	3
✓	✓			✓								100	3
✓	✓				✓							97	3
✓		✓	✓									97	3
✓		✓		✓								100	3
✓		✓			✓							100	3

Figure S38. *S. pyogenes* differentiation results. The first 30 combinations result in an accuracy higher than 95% for differentiating *S. pyogenes* from other bacteria in the panel. Check-mark- probes indicate the probe combinations that were used in the 1-nearest neighbor analysis.

probe 1	probe 2	probe 3	probe 4	probe 5	probe 6	probe 7	probe 8	probe 9	probe 10	probe 11	probe 12	Test Accuracy (%)	Number of probes
✓			✓									100	2
✓				✓								97	2
✓						✓						97	2
		✓	✓									97	2
		✓		✓								100	2
		✓				✓						97	2
		✓					✓					97	2
			✓	✓								97	2
			✓		✓							97	2
			✓								✓	97	2
			✓			✓						100	2
			✓				✓					100	2
				✓	✓							97	2
				✓		✓						100	2
				✓					✓			97	2
						✓		✓				100	2
						✓			✓			97	2
✓	✓		✓									100	3
✓	✓					✓						97	3
✓		✓	✓									100	3
✓		✓		✓								97	3
✓		✓									✓	97	3
✓		✓				✓						100	3
✓			✓	✓								100	3
✓			✓		✓							97	3
✓			✓							✓		100	3
✓			✓								✓	100	3
✓			✓			✓						97	3
✓			✓				✓					97	3
✓			✓					✓				100	3

Figure S39. *P. aeruginosa* differentiation results. The first 30 combinations result in an accuracy higher than 95% for differentiating *P. aeruginosa* from other bacteria in the panel. Check-mark- probes indicate the probe combinations that were used in the 1-nearest neighbor analysis.

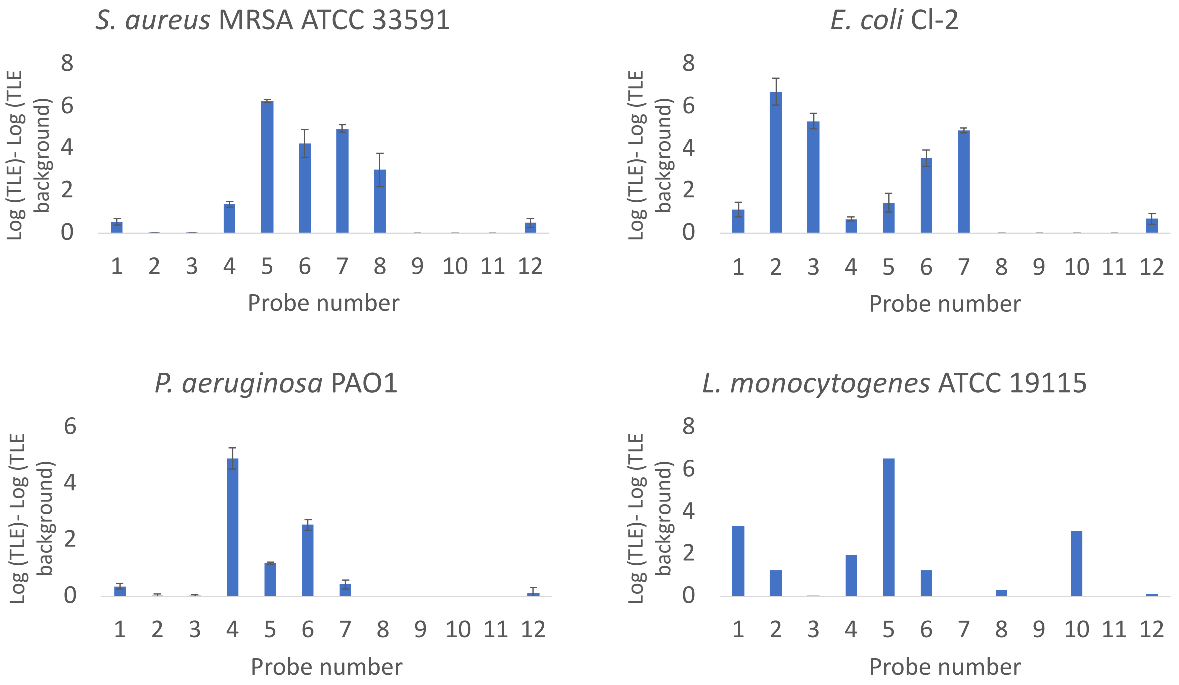

Figure S40. Log total light emission profiles of the chemiluminescent array with four bacteria not initially included in the panel, [OD_{600}= 0.4], in PBS (pH 7.4), 0.1% DMSO, 37°C. The background represents probe without bacteria.

NMR spectra

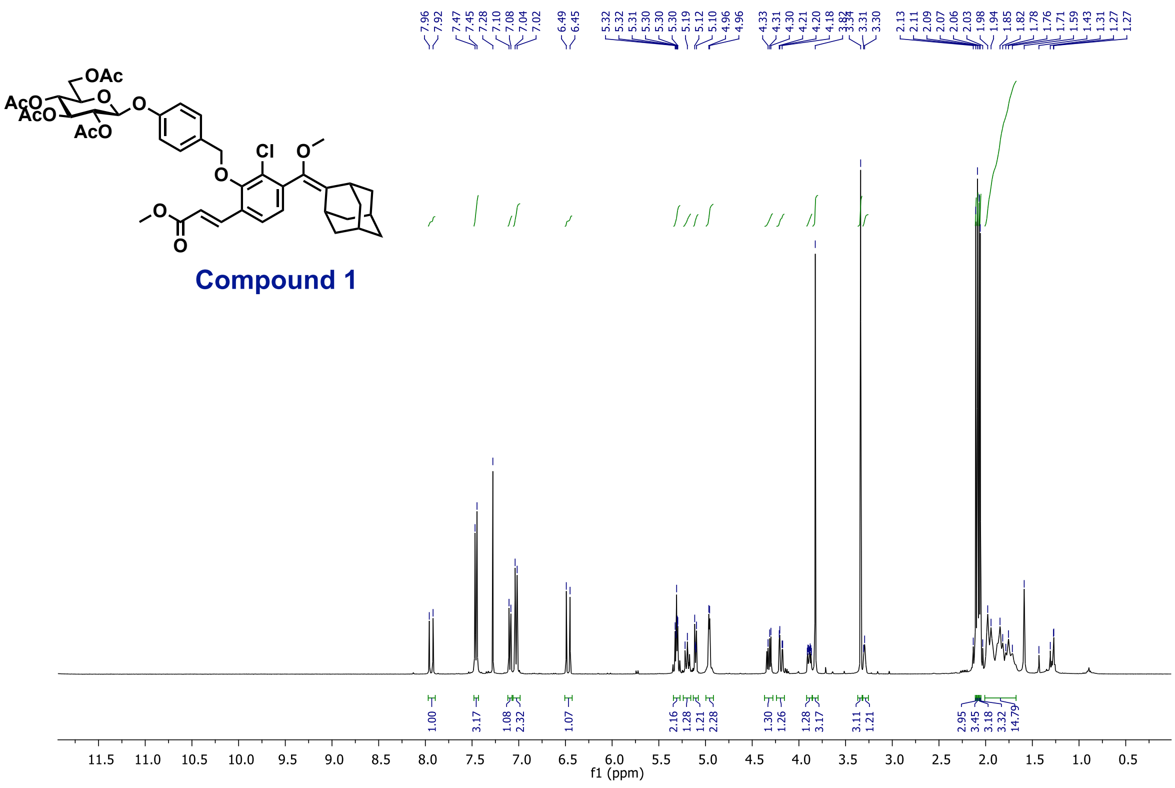

Figure S41. ^{1}H-NMR (400 MHz, $CDCl_3$) spectra of compound **1**.

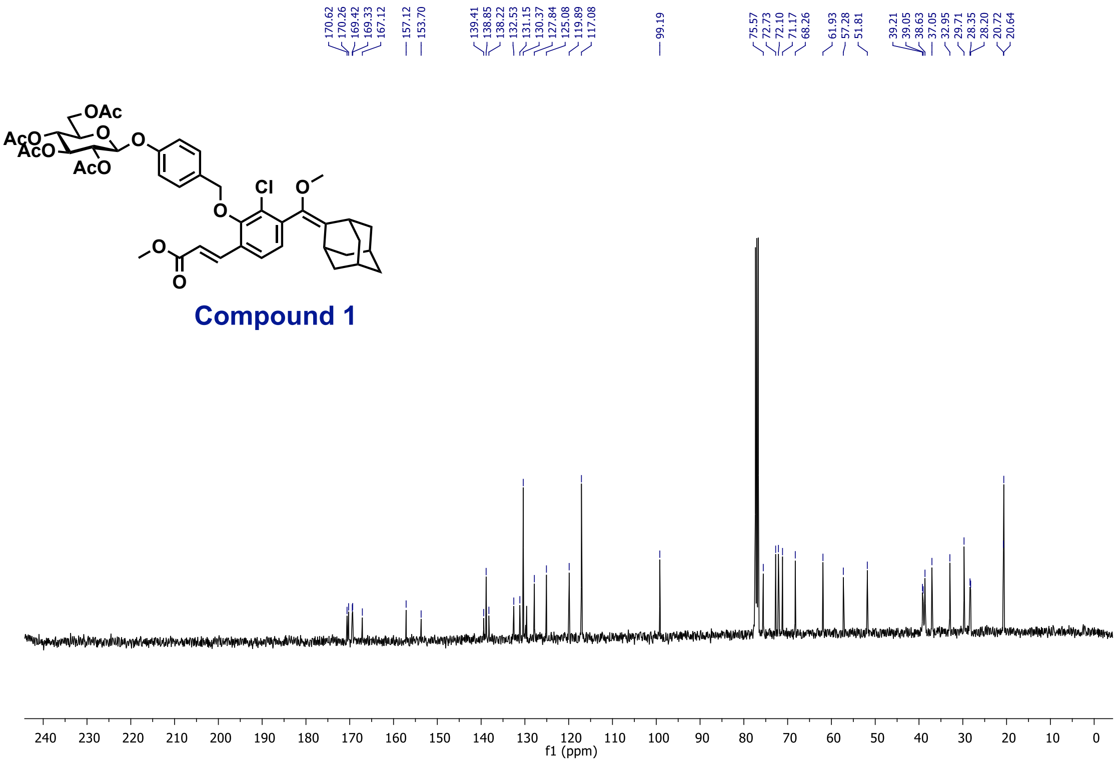

Figure S42. ^{13}C-NMR (100 MHz, $CDCl_3$) spectra of compound **1**.

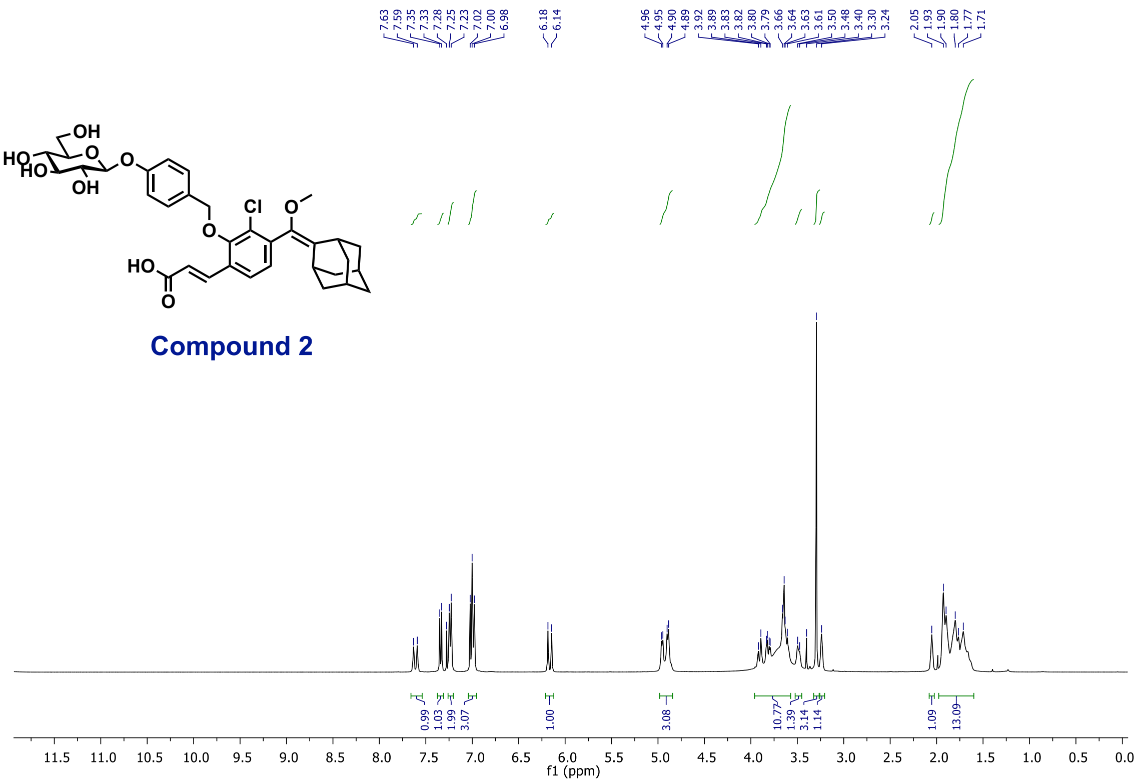

Compound 2

Figure S43. ^{1}H-NMR (400MHz, $CDCl_3$) spectra of compound **2**.

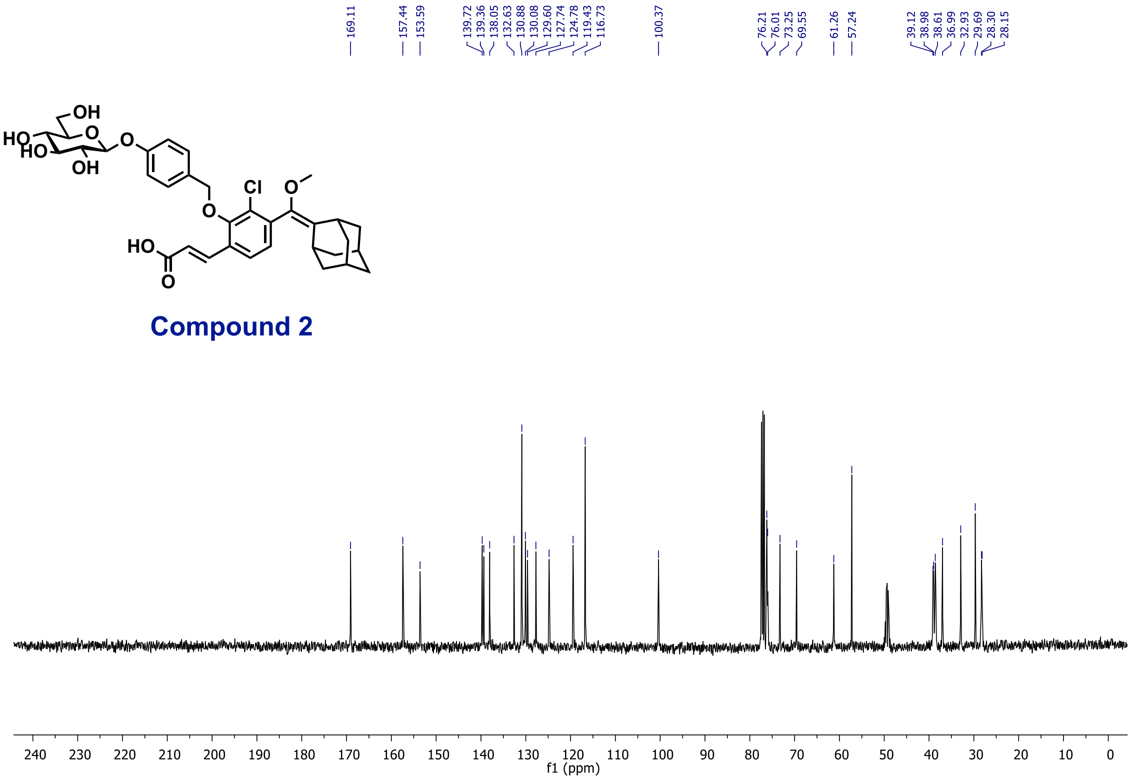

Compound 2

Figure S44. ^{13}C-NMR (100 MHz, $CDCl_3$) spectra of compound **2.**

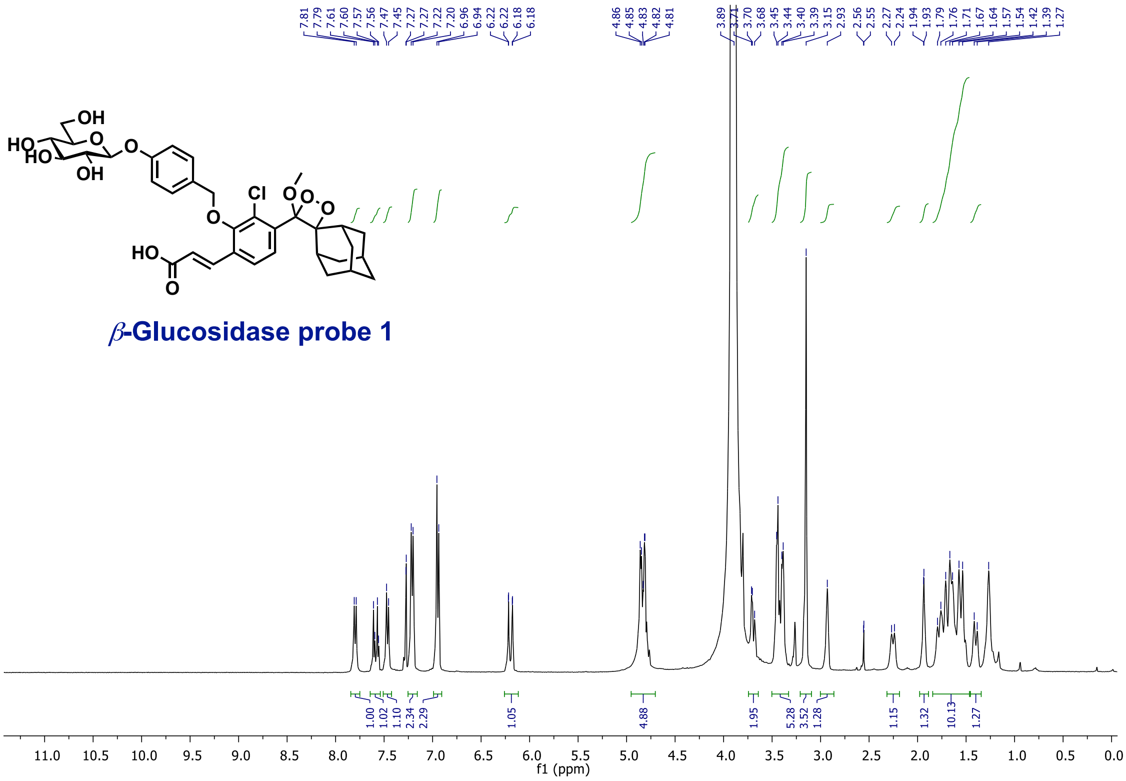

Figure S45. ^{1}H-NMR (400MHz, $CDCl_3$) spectra of ***β*-glucosidase probe 1**.

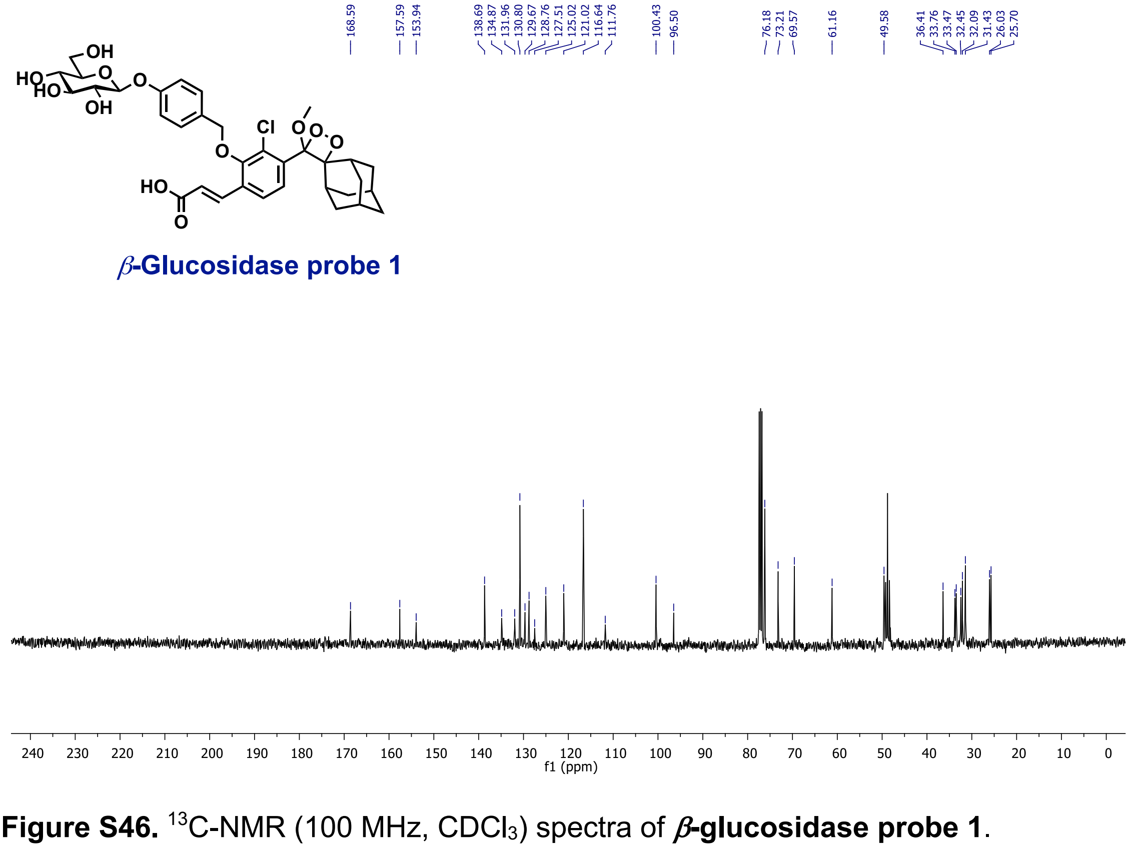

Figure S46. ^{13}C-NMR (100 MHz, $CDCl_3$) spectra of ***β*-glucosidase probe 1**.

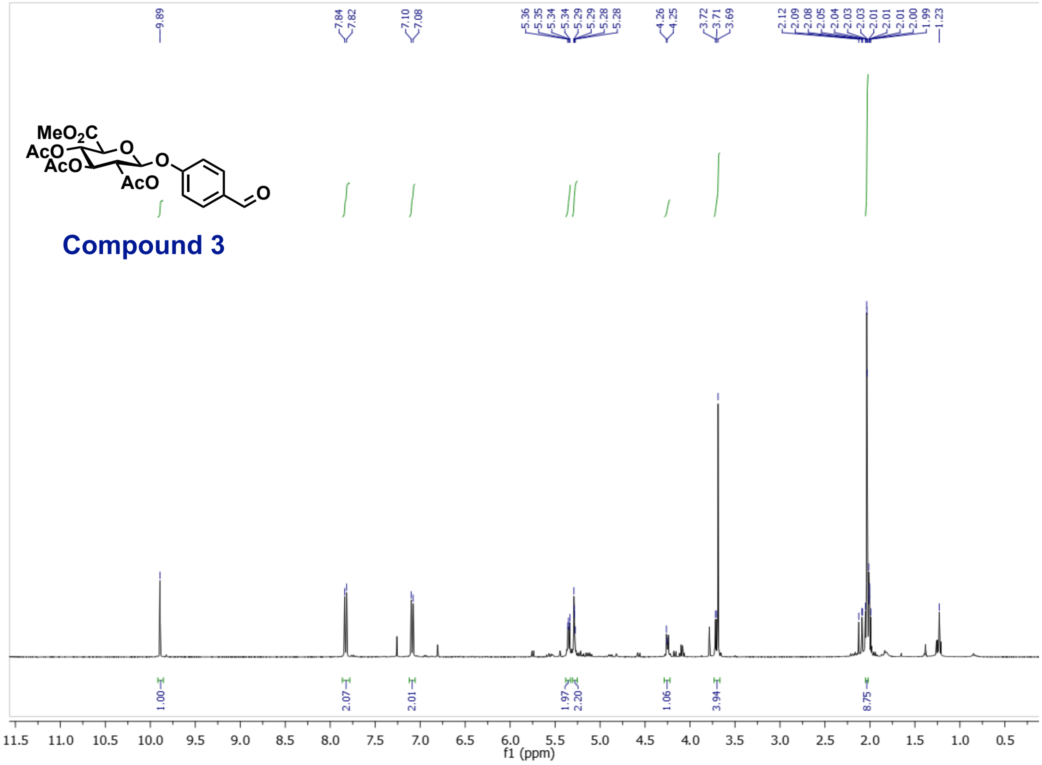

Figure S47. ^{1}H-NMR (400MHz, $CDCl_3$) spectra of compound **3**.

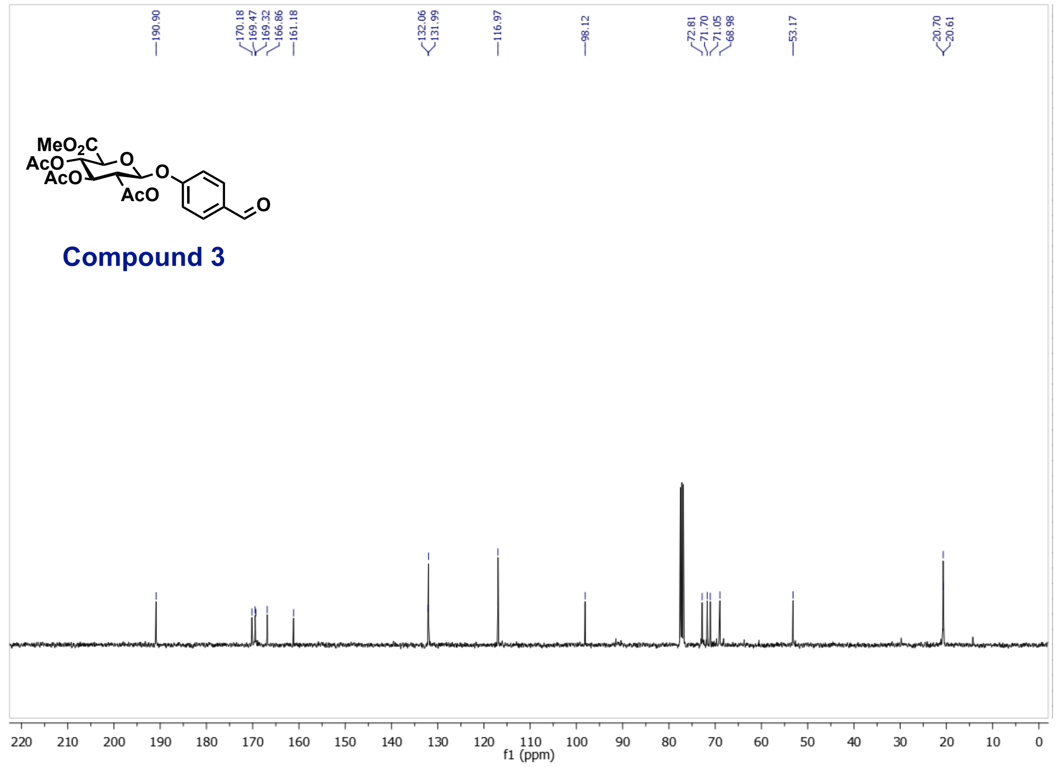

Figure S48. ^{13}C-NMR (100 MHz, $CDCl_3$) spectra of compound **3**.

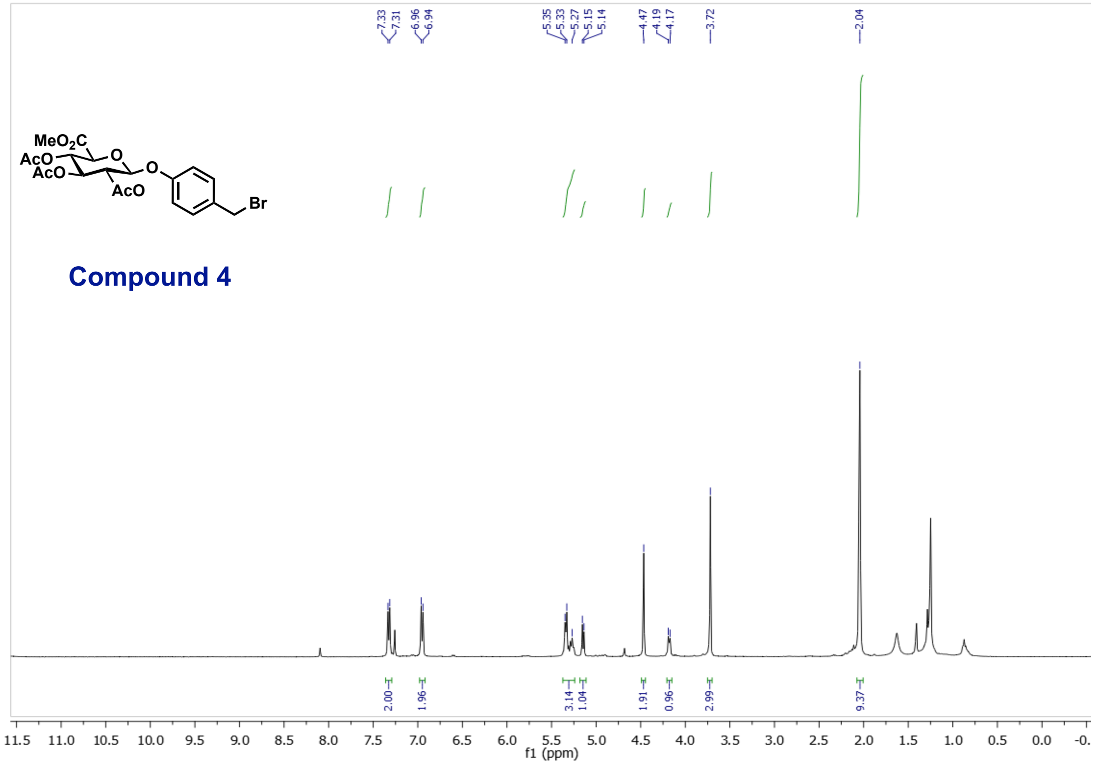

Figure S49. ^{1}H-NMR (400MHz, $CDCl_3$) spectra of compound **4**.

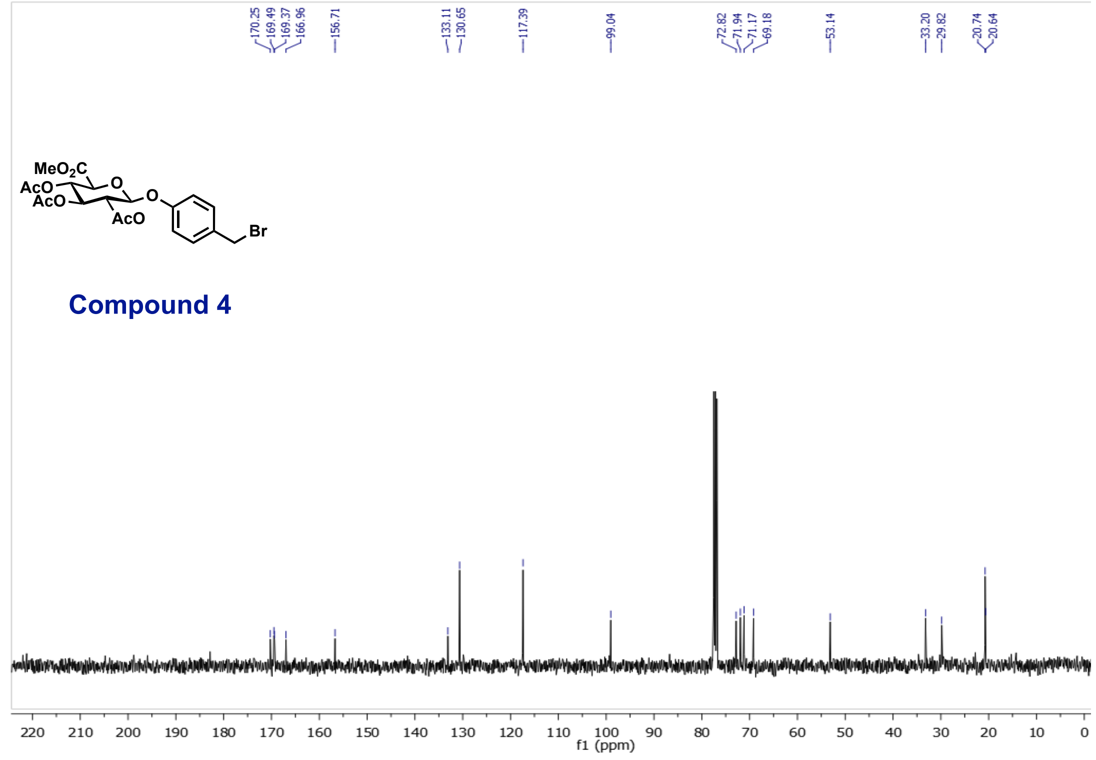

Figure S50. ^{13}C-NMR (100 MHz, $CDCl_3$) spectra of compound **4**.

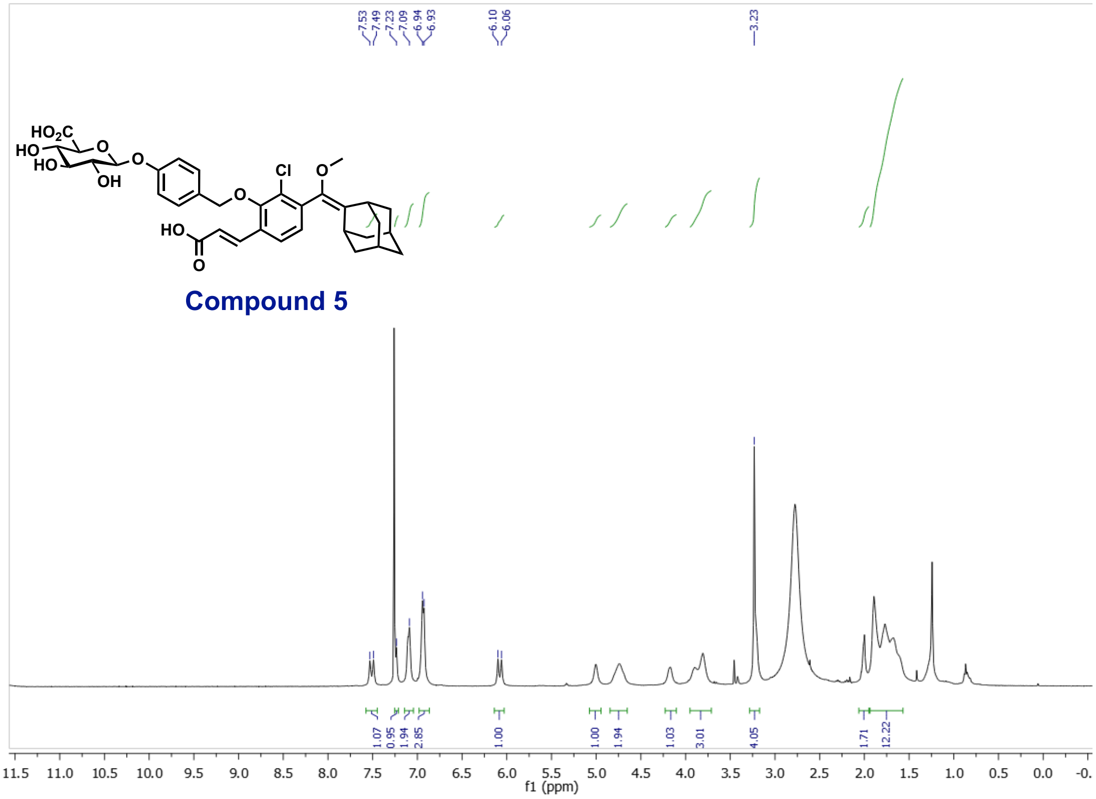

Figure S51. ^{1}H-NMR (400MHz, $CDCl_3$) spectra of compound **5**.

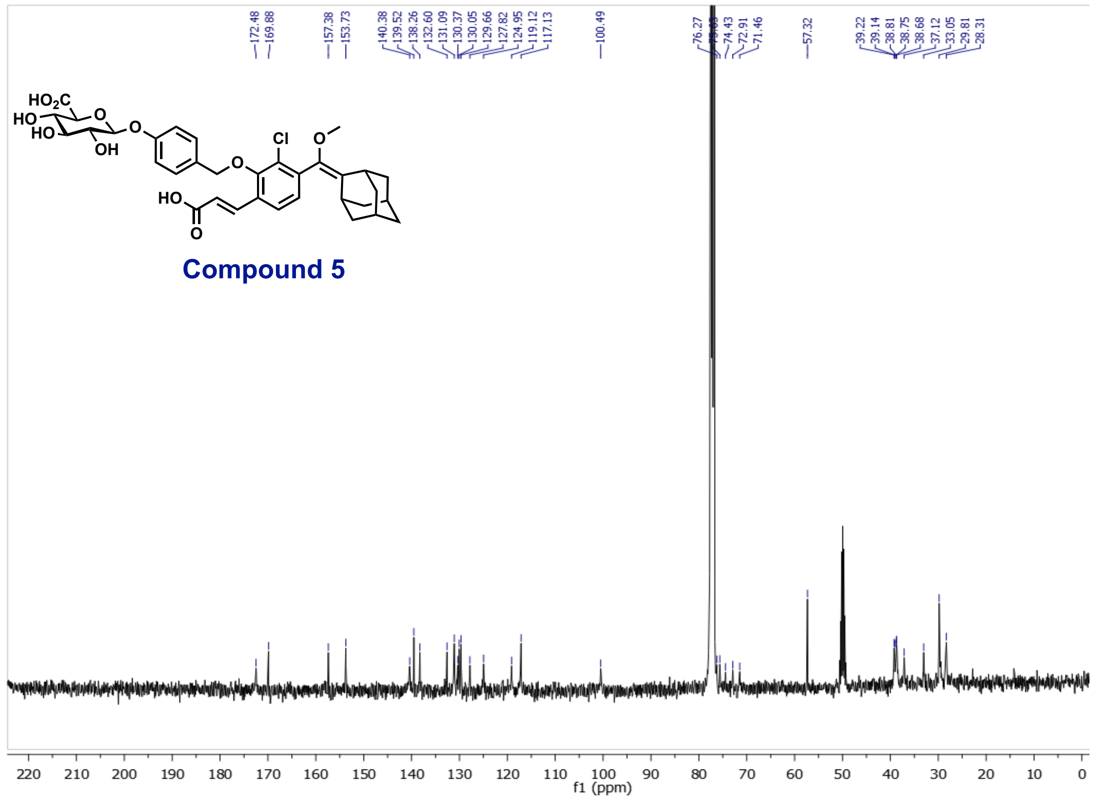

Figure S52. ^{13}C-NMR (100 MHz, $CDCl_3$) spectra of compound **5**.

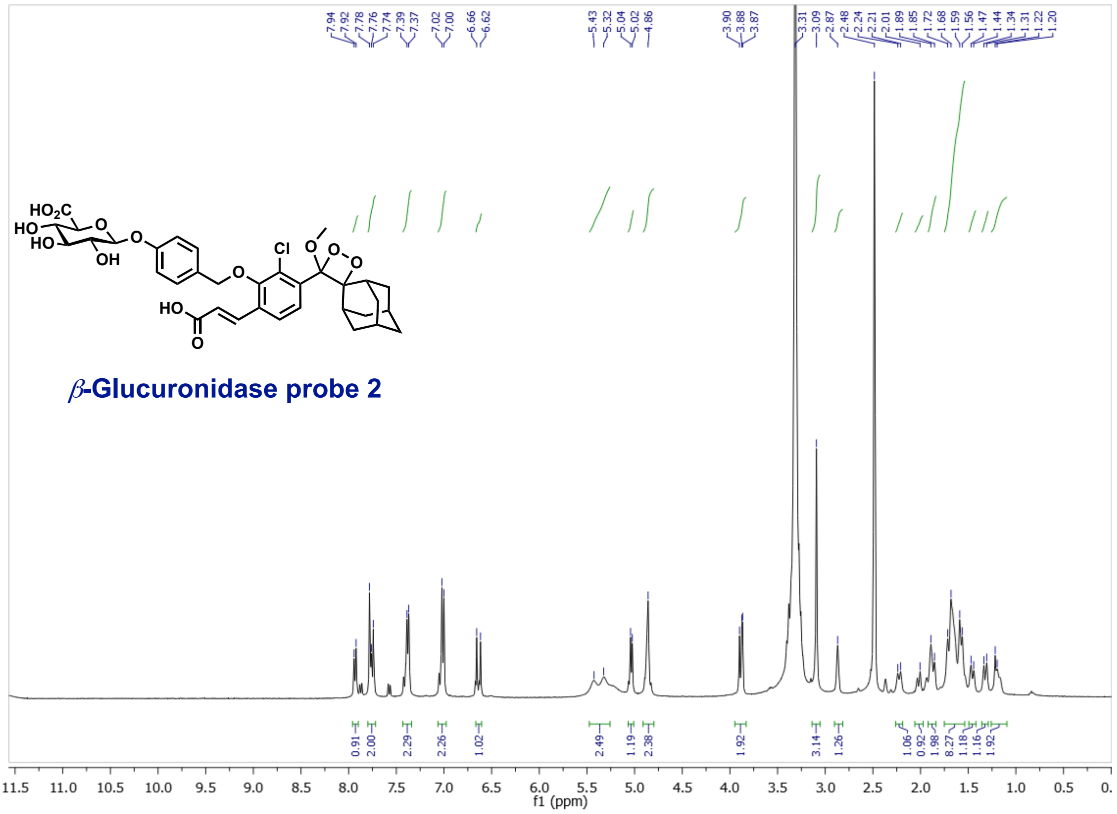

Figure S53. ^{1}H-NMR (400MHz, DMSO) spectra of ***β*-glucuronidase probe 2**.

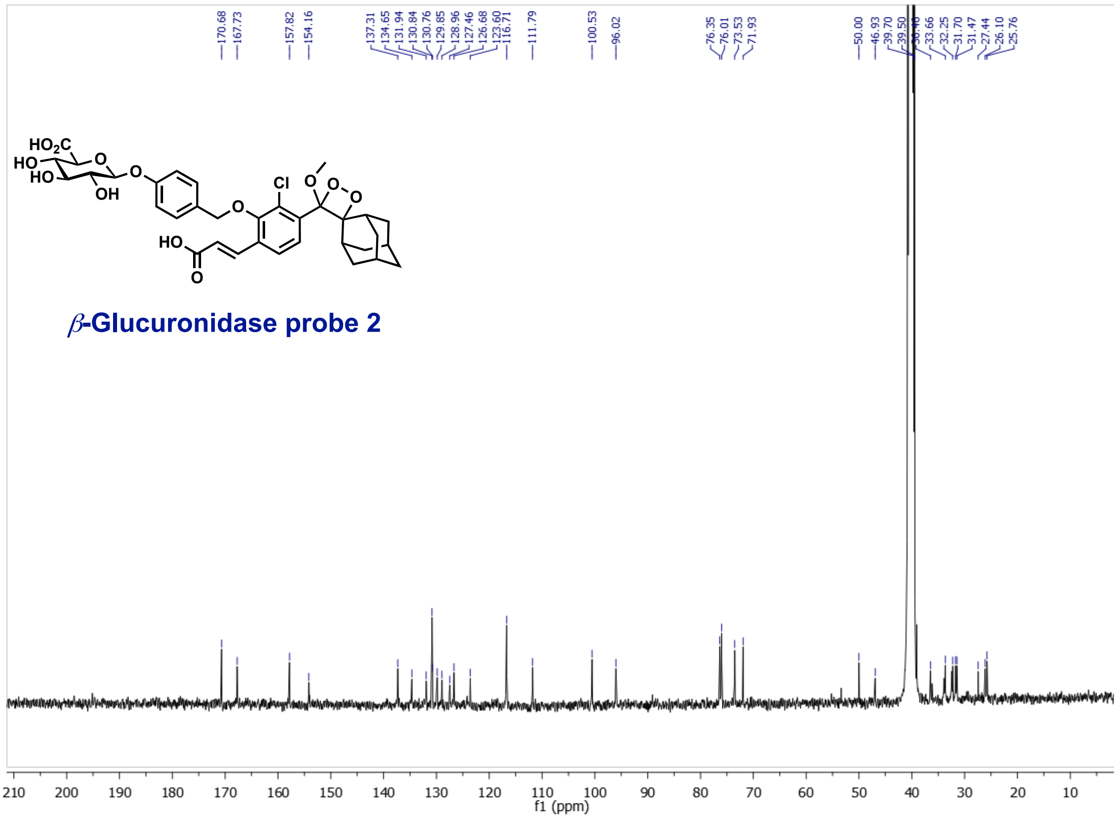

Figure S54. ^{13}C-NMR (100 MHz, DMSO) spectra of ***β*-glucuronidase probe 2**.

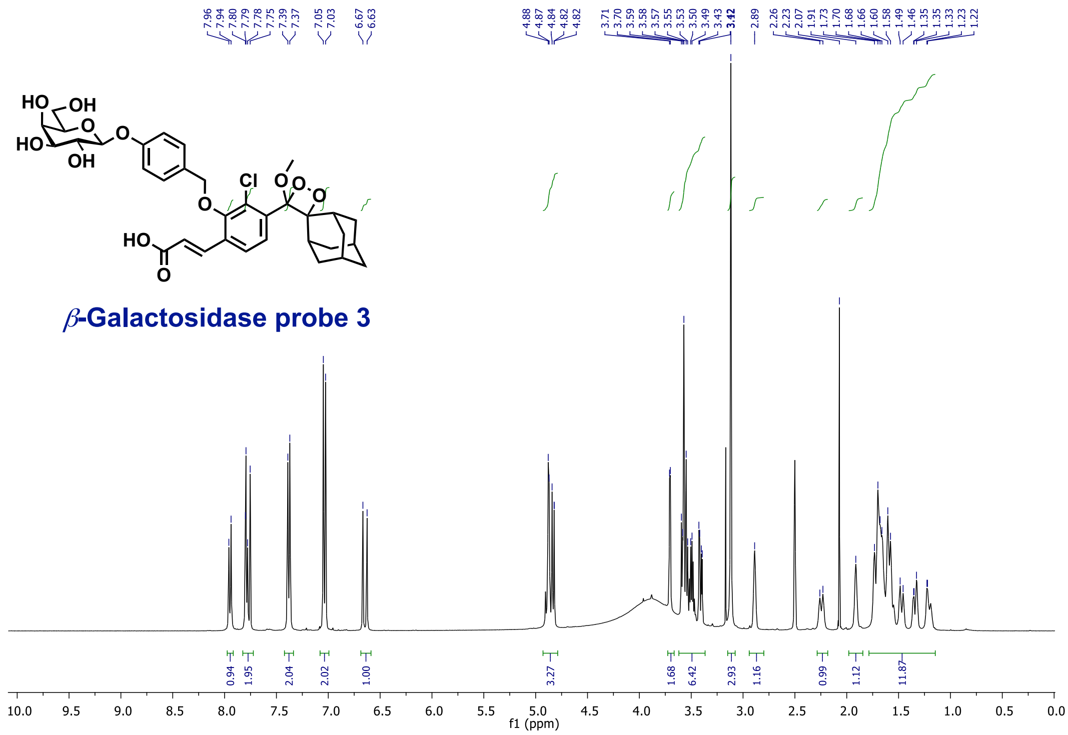

Figure S55. ^{1}H-NMR (400MHz, DMSO) spectra of ***β*-galactosidase probe 3**.

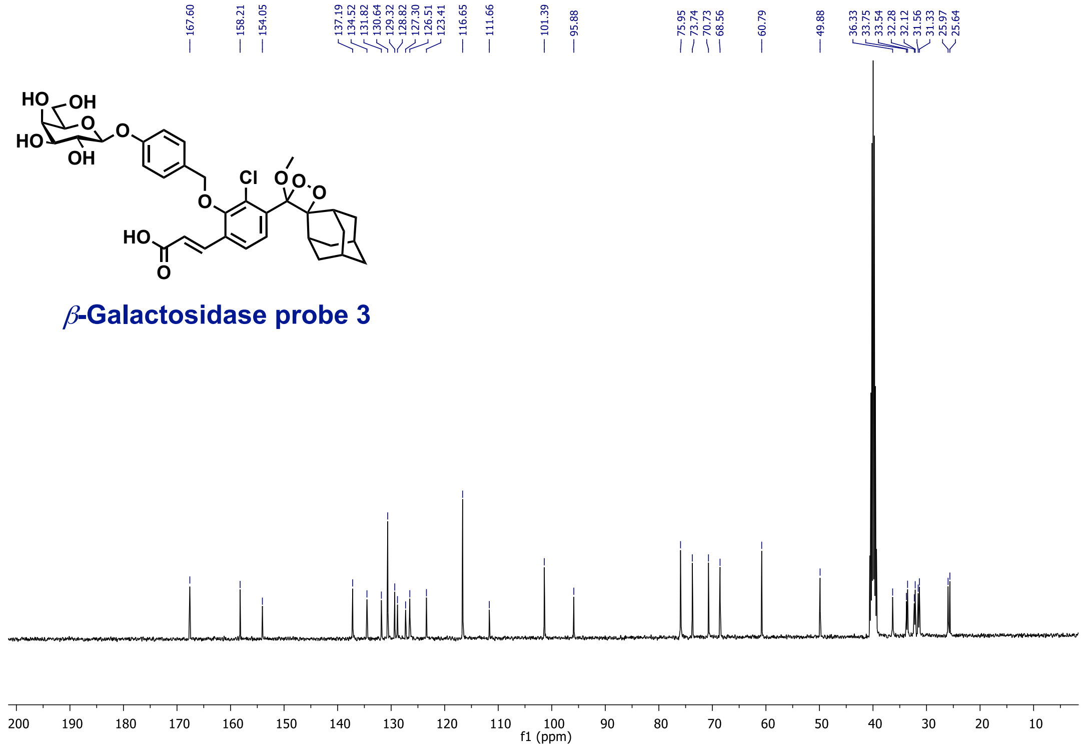

Figure S56. ^{13}C-NMR (100 MHz, DMSO) spectra ***β*-galactosidase probe 3**.

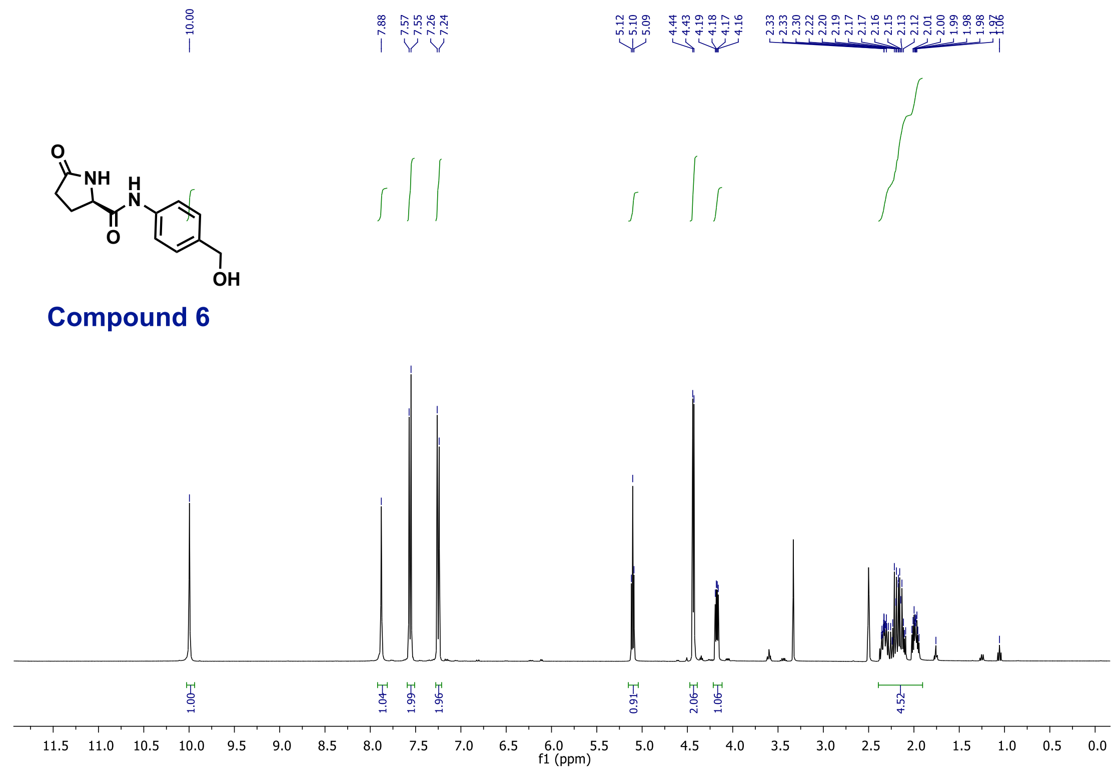

Figure S57. ^{1}H-NMR (400MHz, DMSO) spectra of compound **6**.

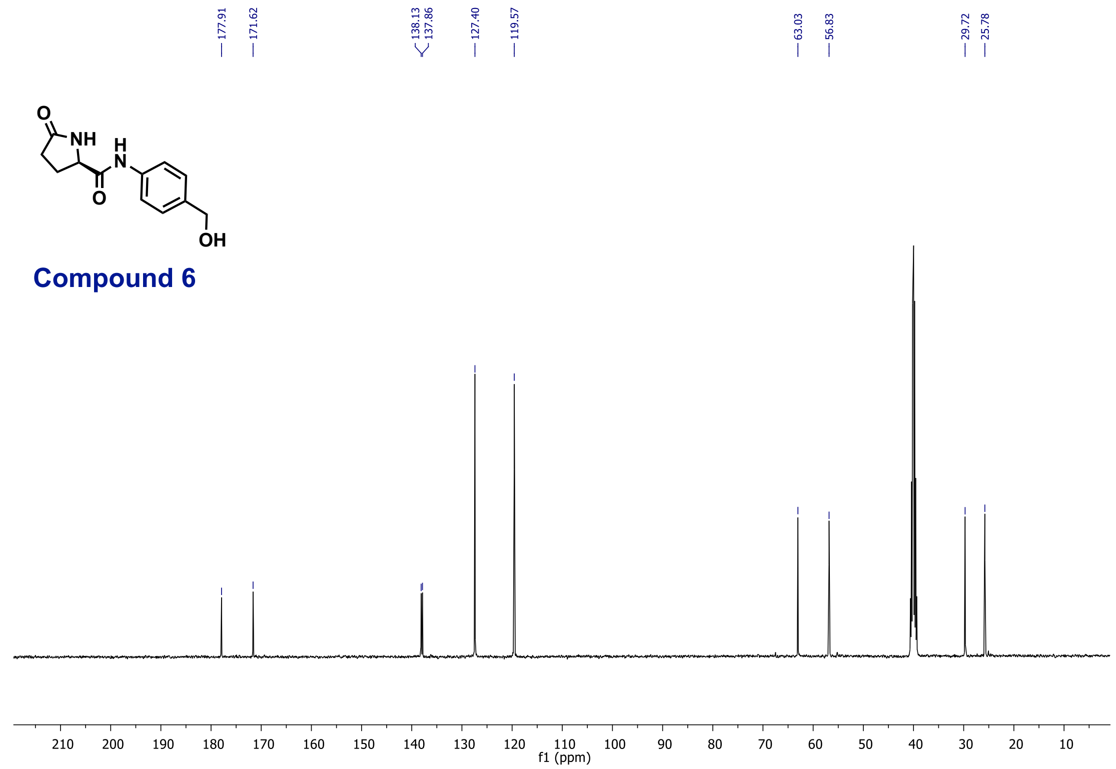

Figure S58. ^{13}C-NMR (100 MHz, DMSO) spectra of **6**.

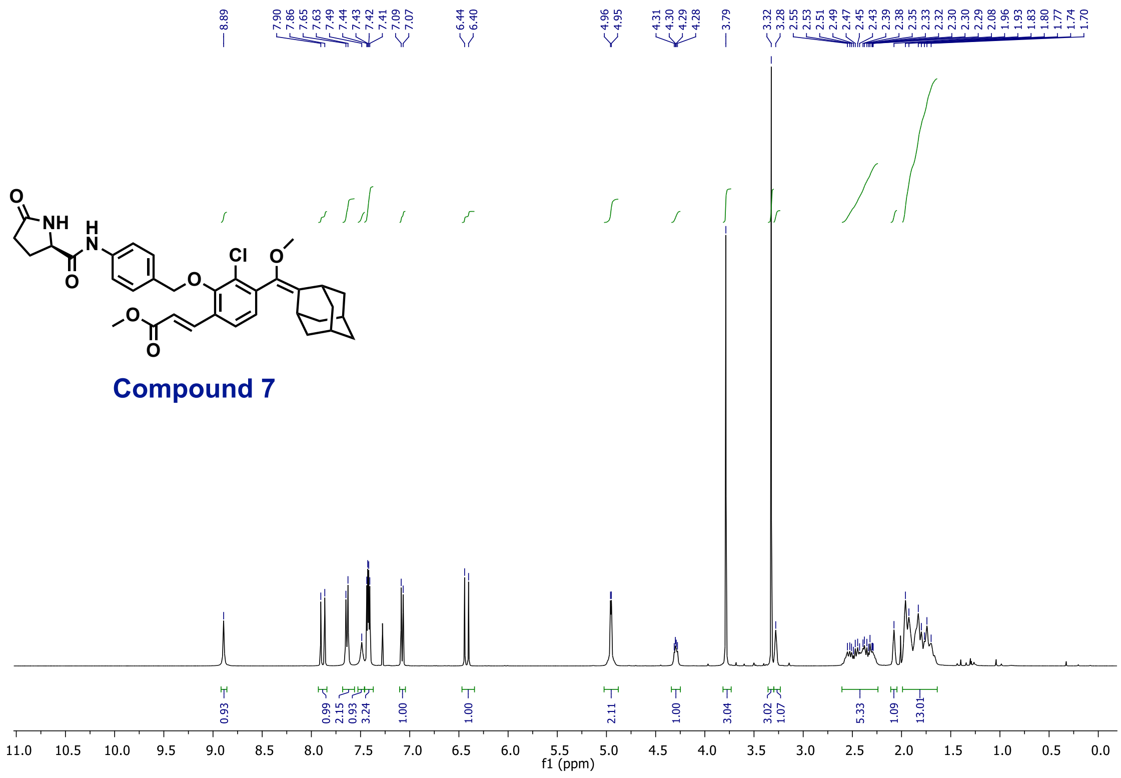

Figure S59. ^{1}H-NMR (400MHz, $CDCl_3$) spectra of compound **7**.

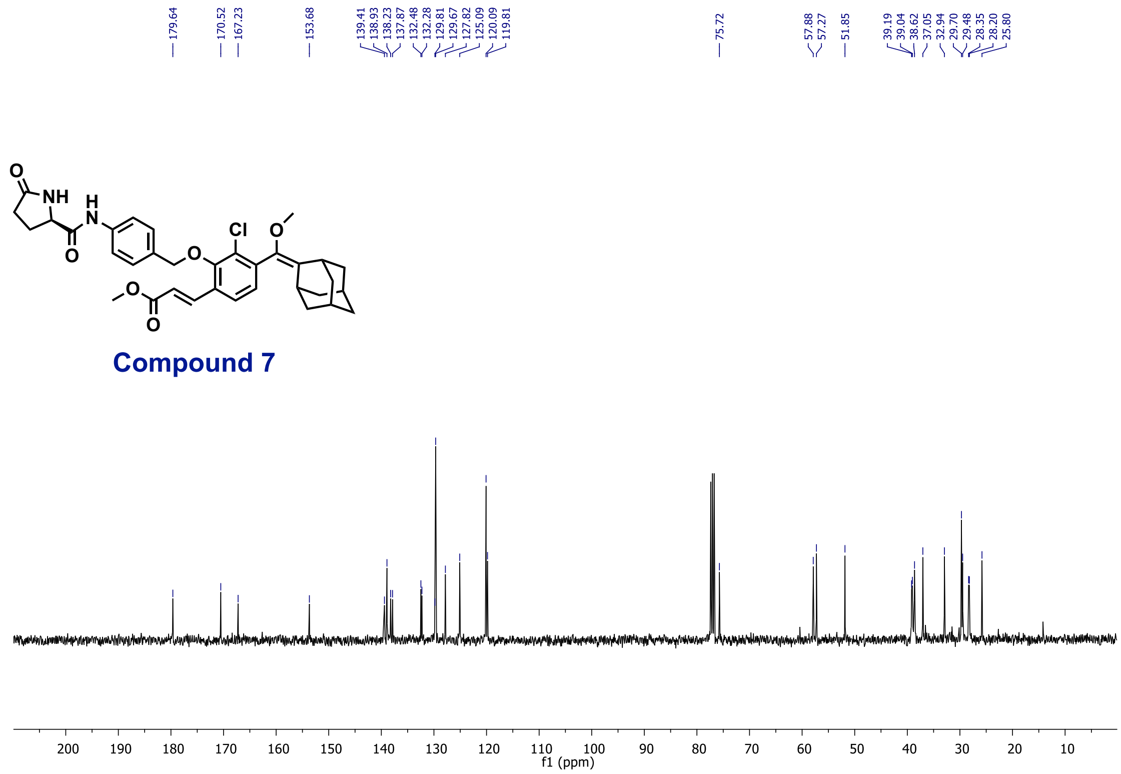

Figure S60. ^{13}C-NMR (100 MHz, $CDCl_3$) spectra of compound **7**.

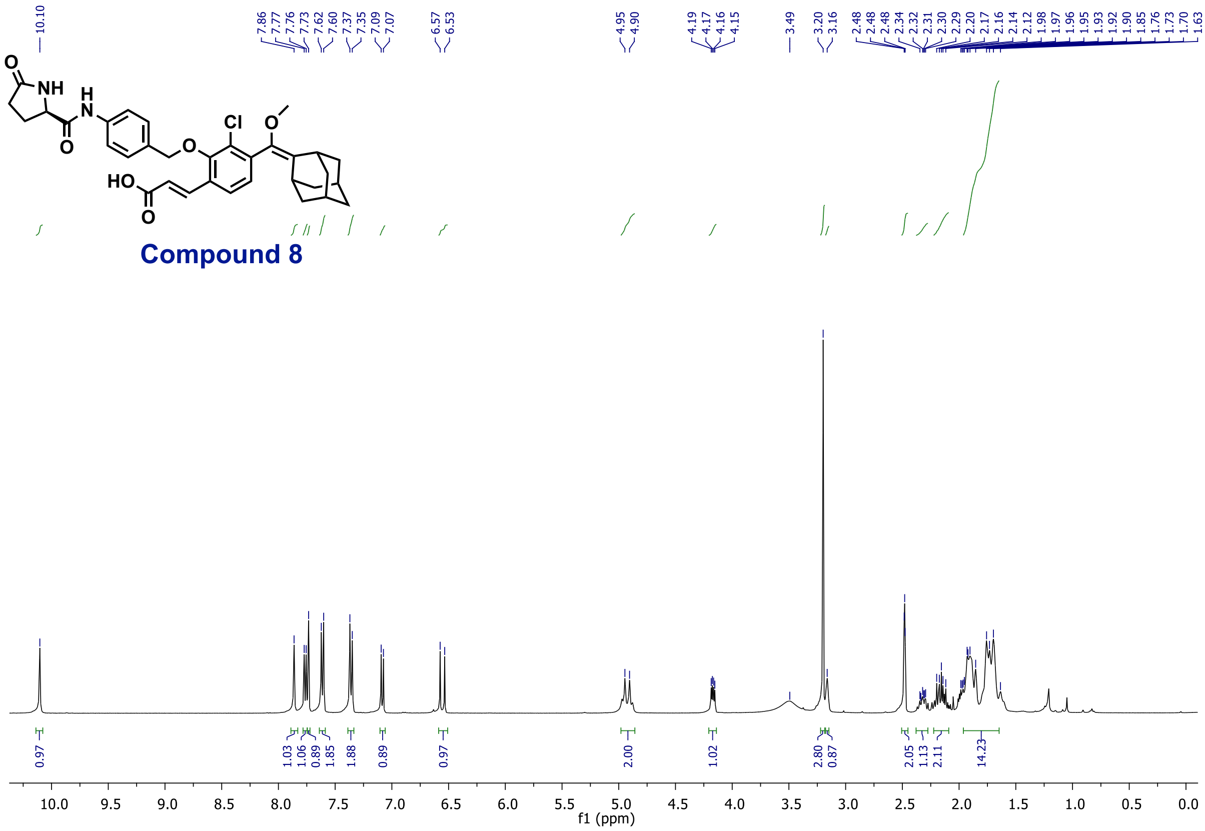

Figure S61. ^{1}H-NMR (400MHz, DMSO) spectra of compound **8**.

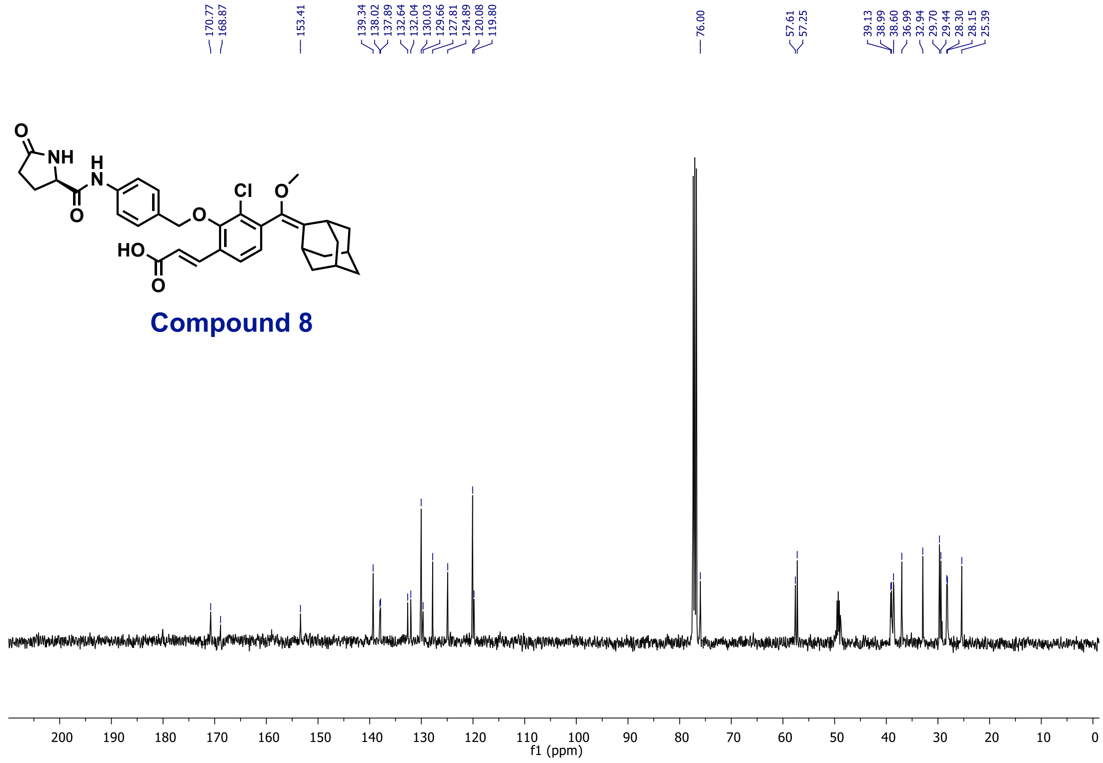

Figure S62. ^{13}C-NMR (100 MHz, $CDCl_3$) spectra of compound **8**.

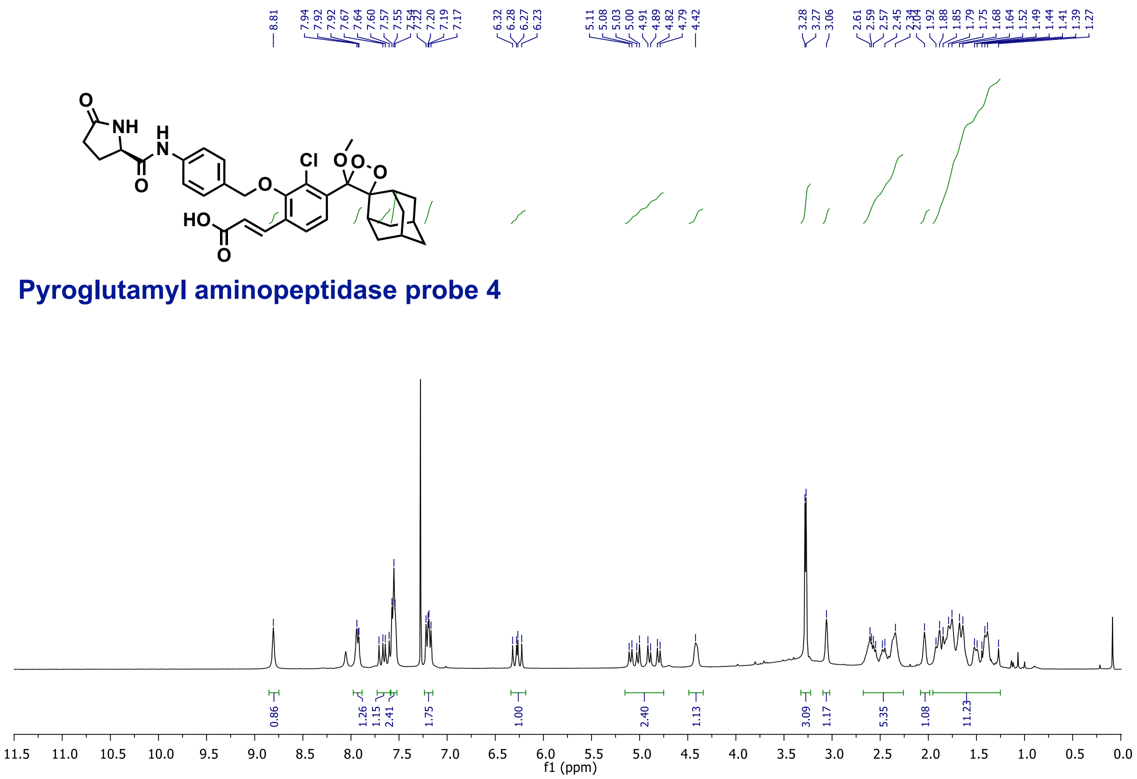

Figure S63. ^{1}H-NMR (400MHz, $CDCl_3$) spectra of **pyroglutamyl aminopeptidase probe 4**.

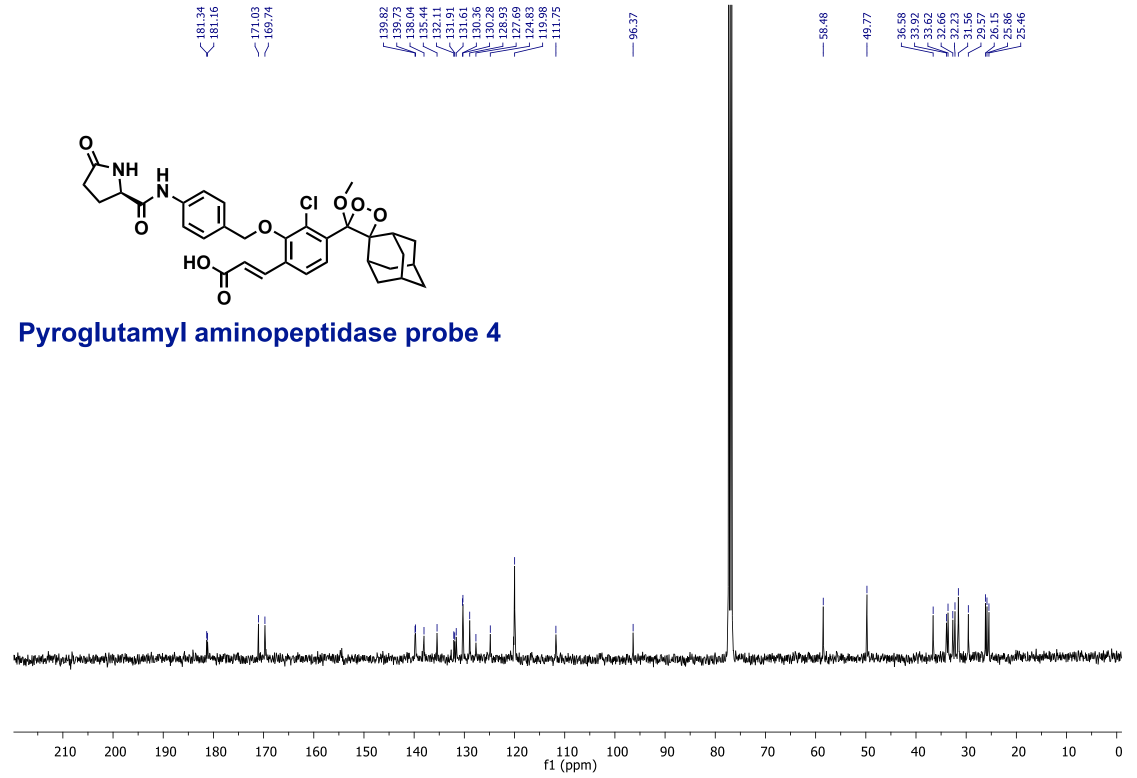

Figure S64. ^{13}C-NMR (100 MHz, $CDCl_3$) spectra of **pyroglutamyl aminopeptidase probe 4**.

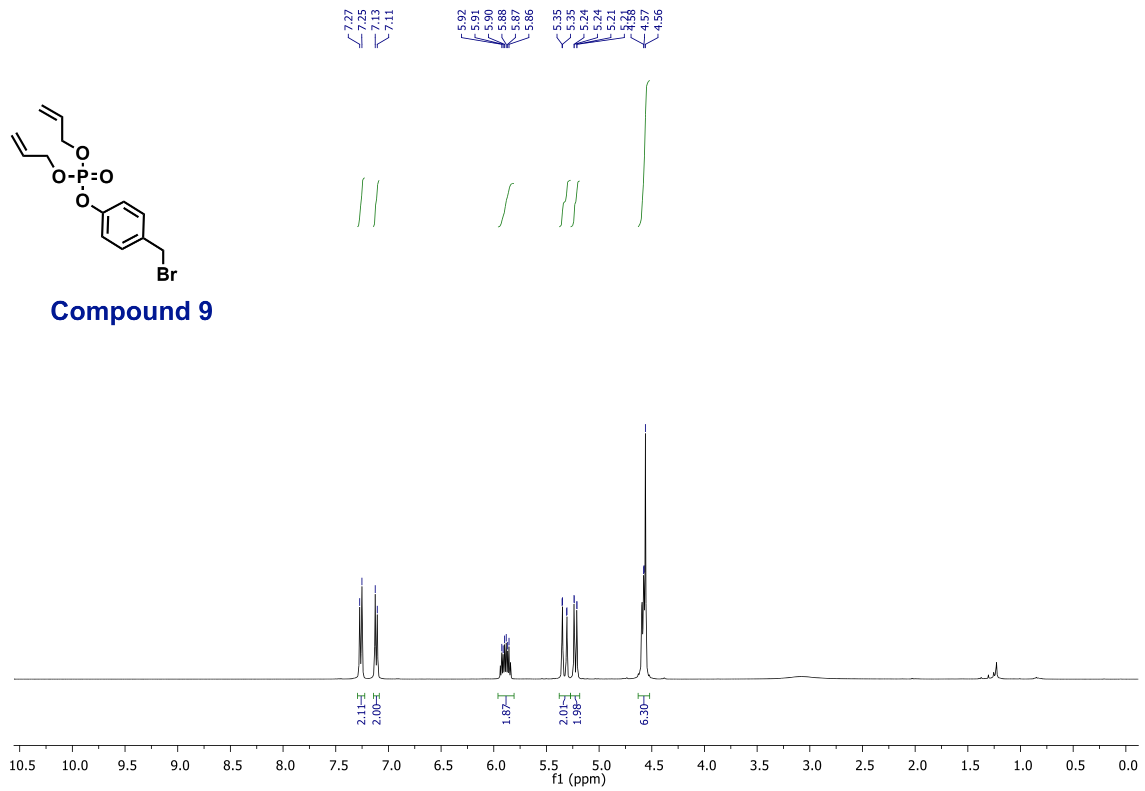

Figure S65. ^{1}H-NMR (400MHz, $CDCl_3$) spectra of compound **9**.

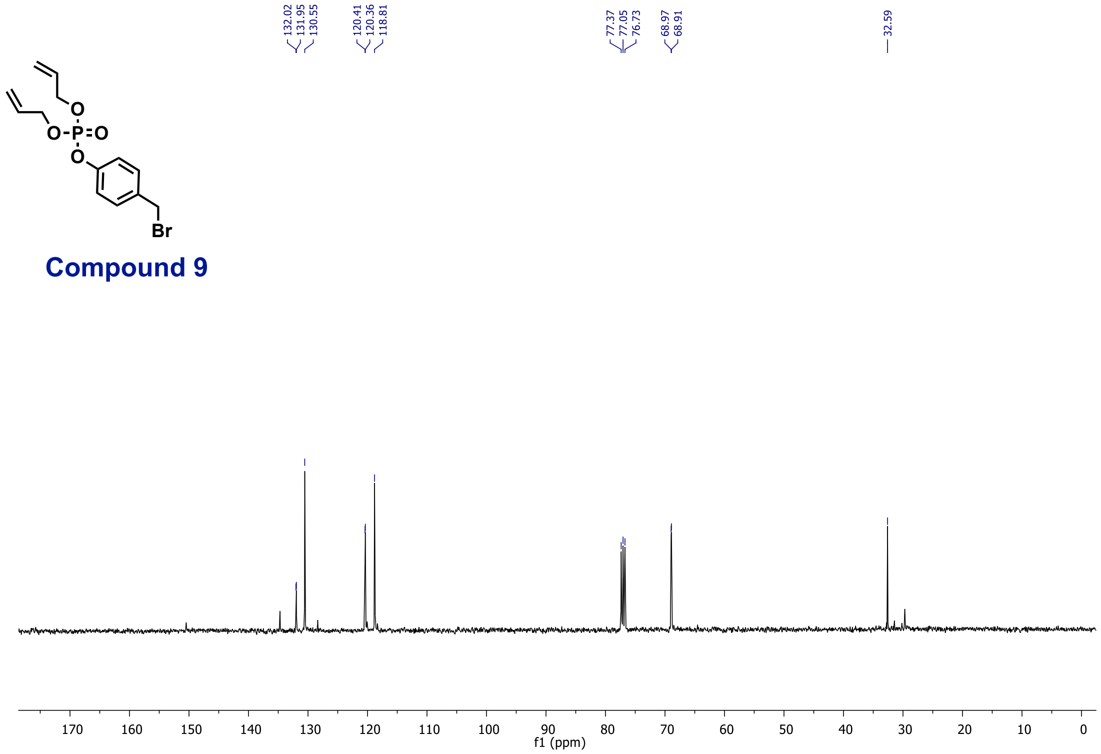

Figure S66. ^{13}C-NMR (100 MHz, $CDCl_3$) spectra of compound **9**.

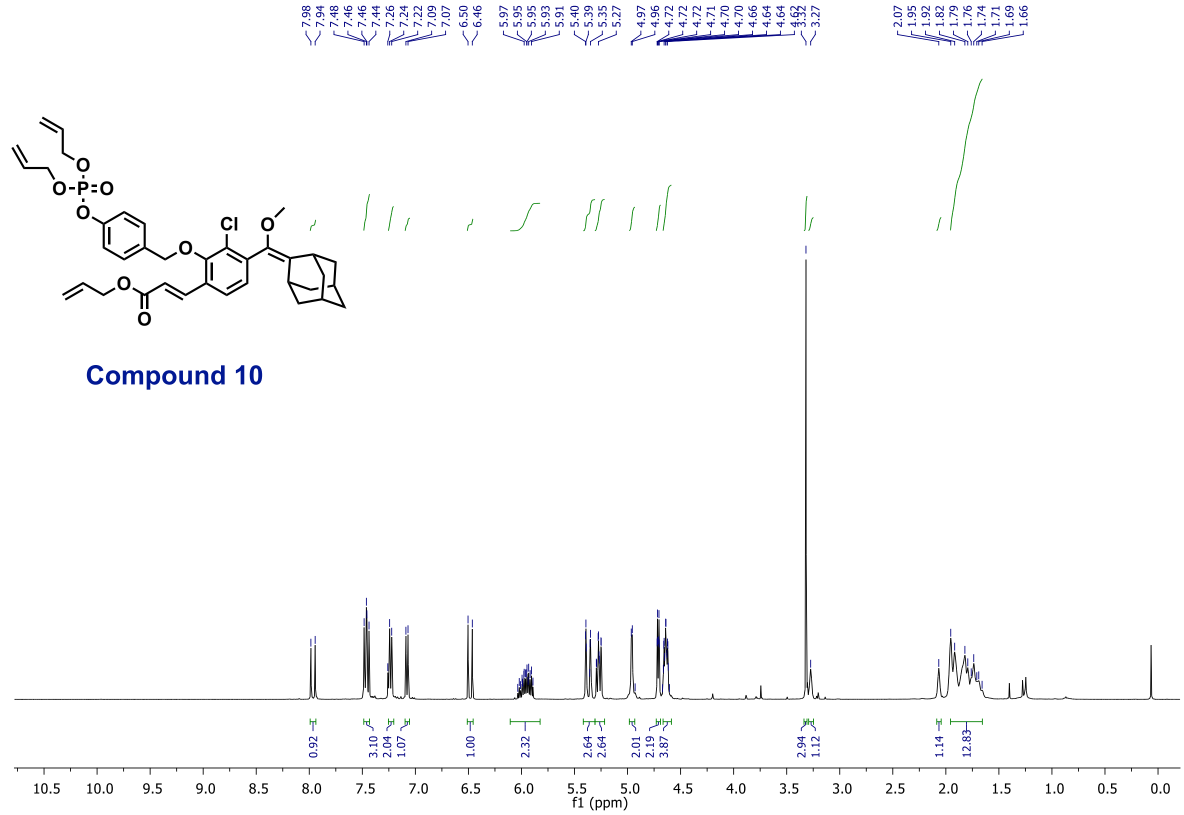

Figure S67. ^{1}H-NMR (400MHz, $CDCl_3$) spectra of compound **10**.

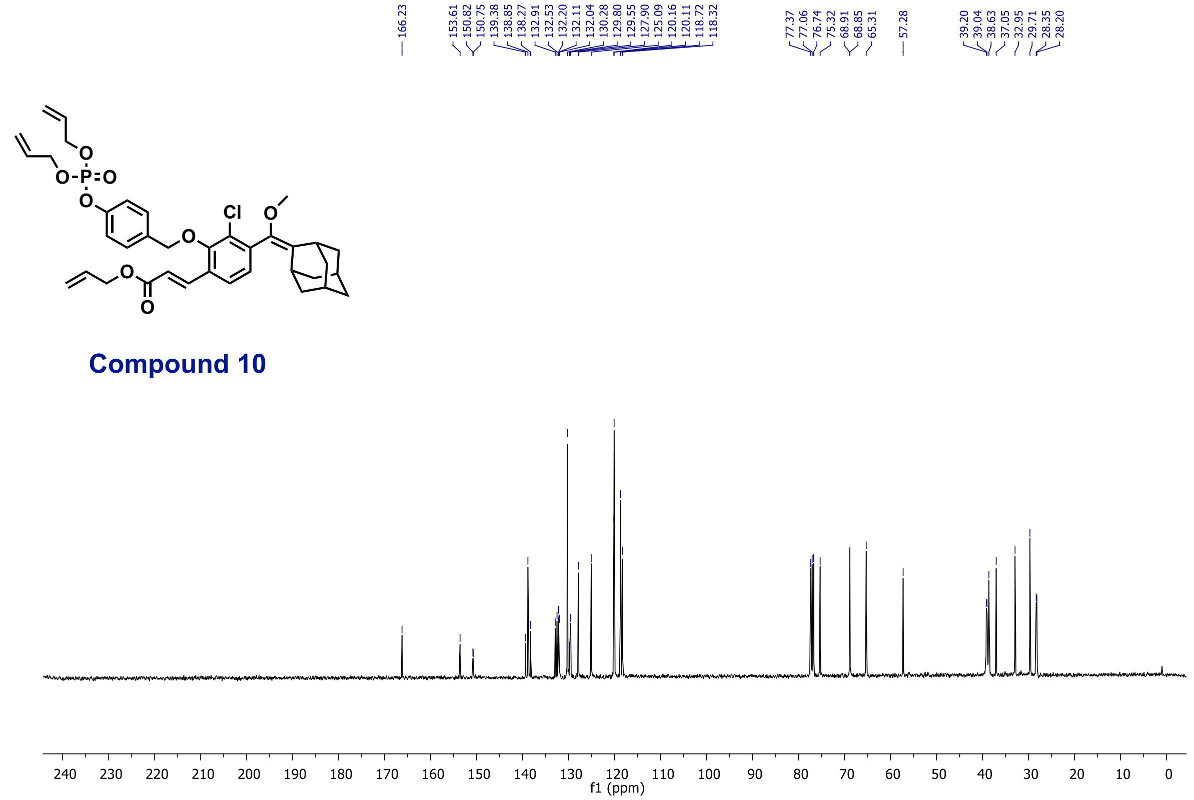

Figure S68. ^{13}C-NMR (100 MHz, $CDCl_3$) spectra of compound **10**.

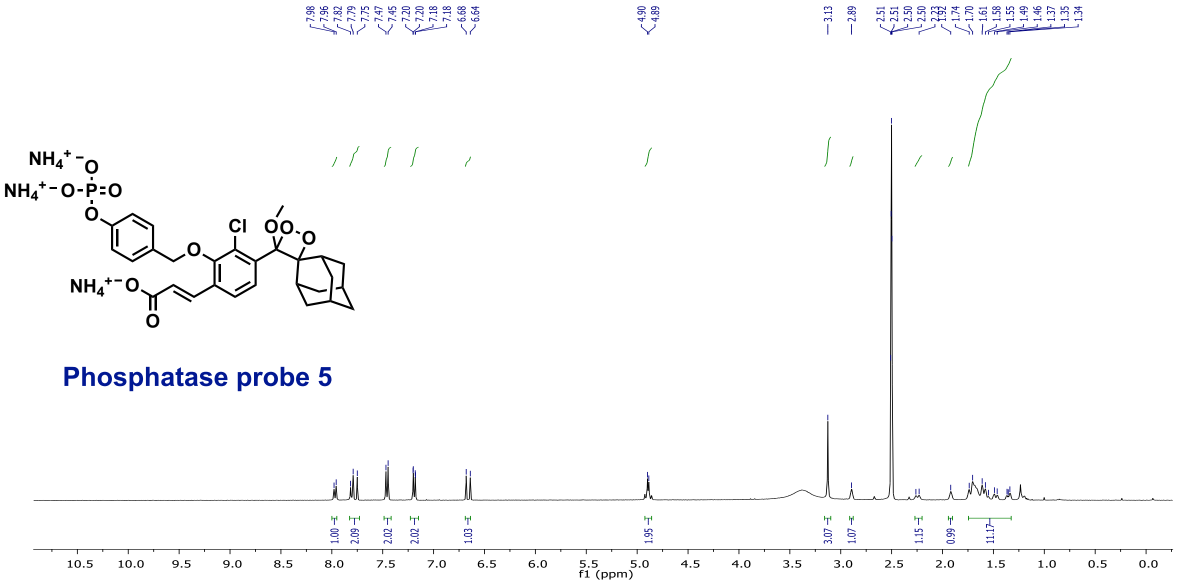

Figure S69. ^{1}H-NMR (400MHz, DMSO) spectra of **phosphatase probe 5**.

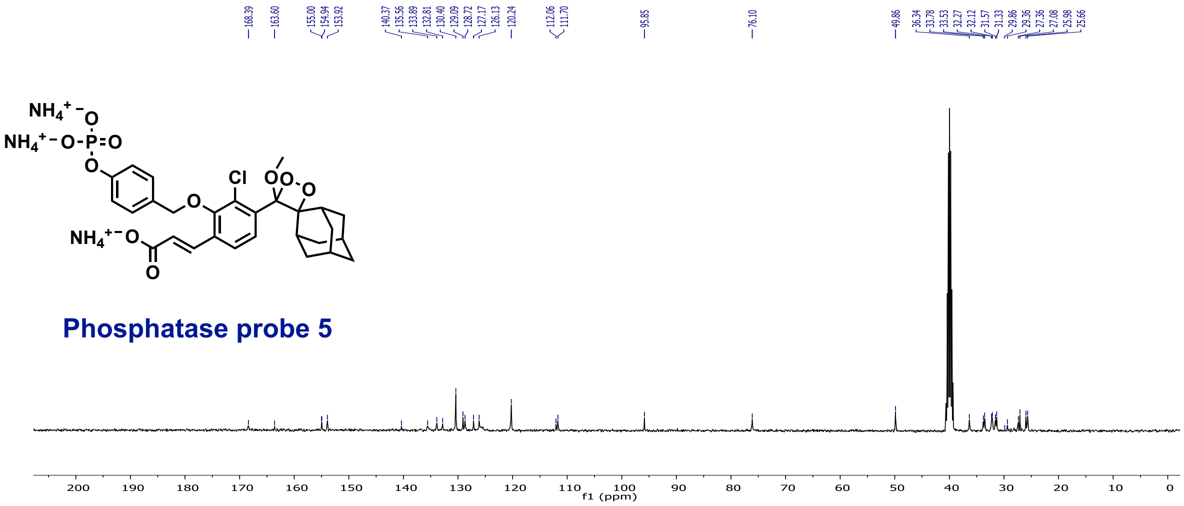

Figure S70. ^{13}C-NMR (100 MHz, DMSO) spectra of **phosphatase probe 5**.

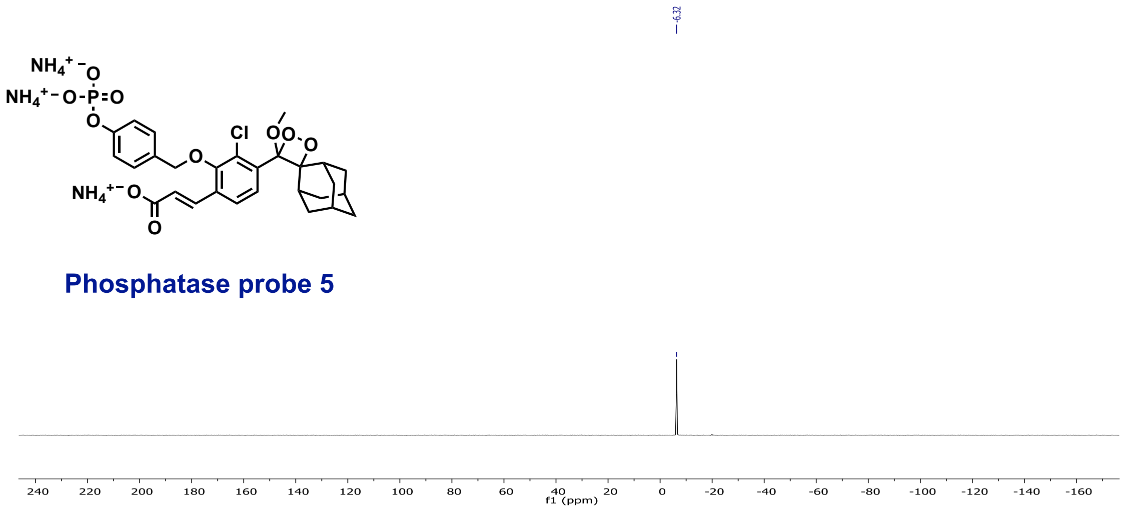

Figure S71. ^{31}P-NMR (100 MHz, DMSO) spectra of **phosphatase probe 5**.

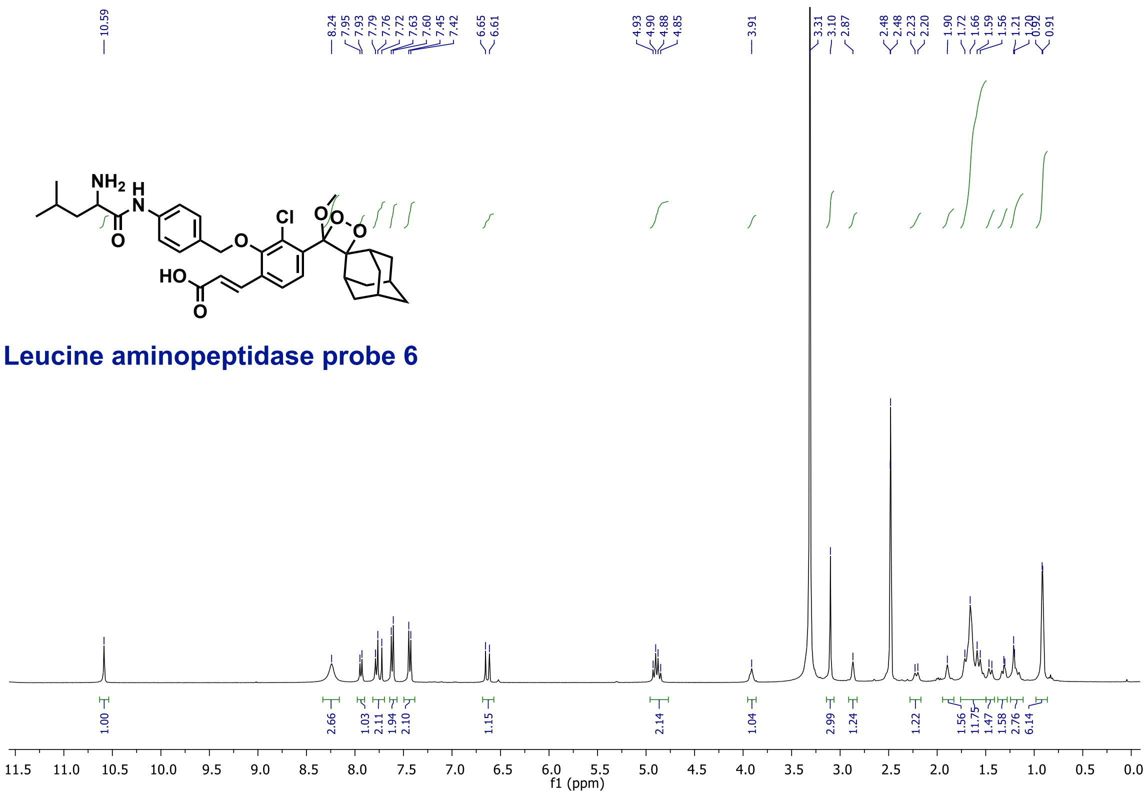

Figure S72. ^{1}H-NMR (400MHz, DMSO) spectra of **Leucine aminopeptidase probe 6**.

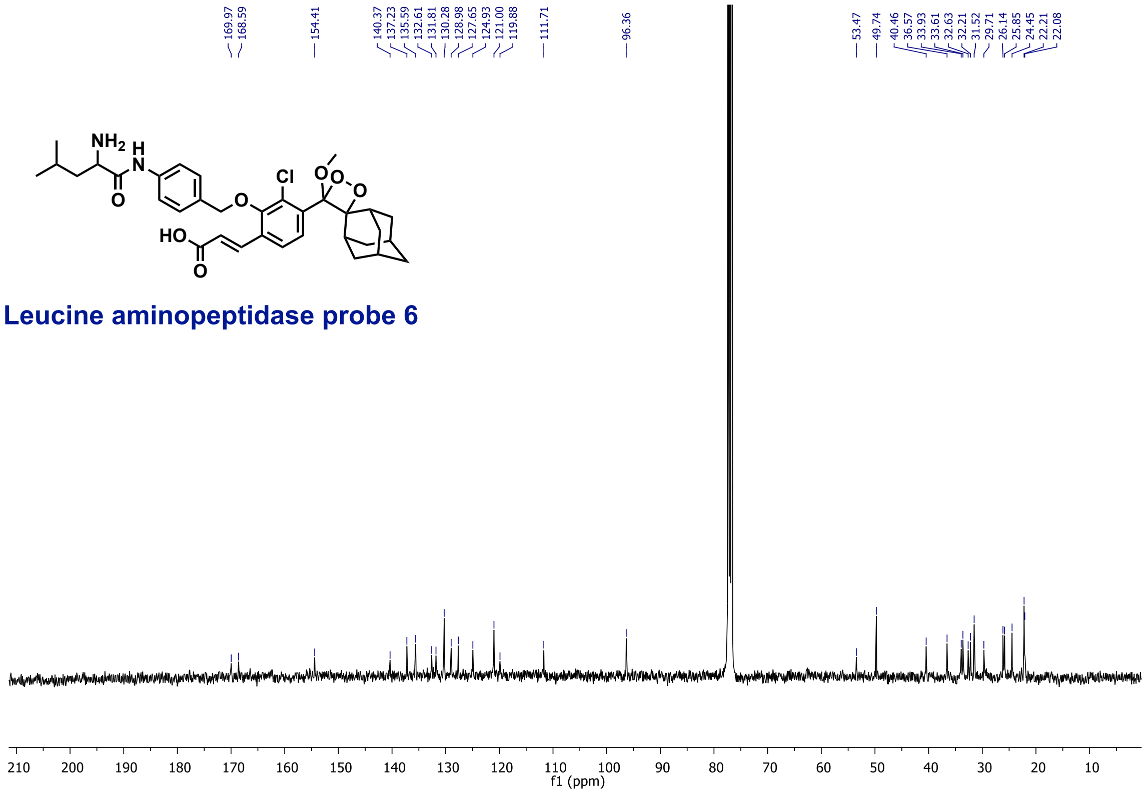

Figure S73. ^{13}C-NMR (100 MHz, 5% MeOD in $CDCl_3$) spectra of **Leucine aminopeptidase probe 6**.

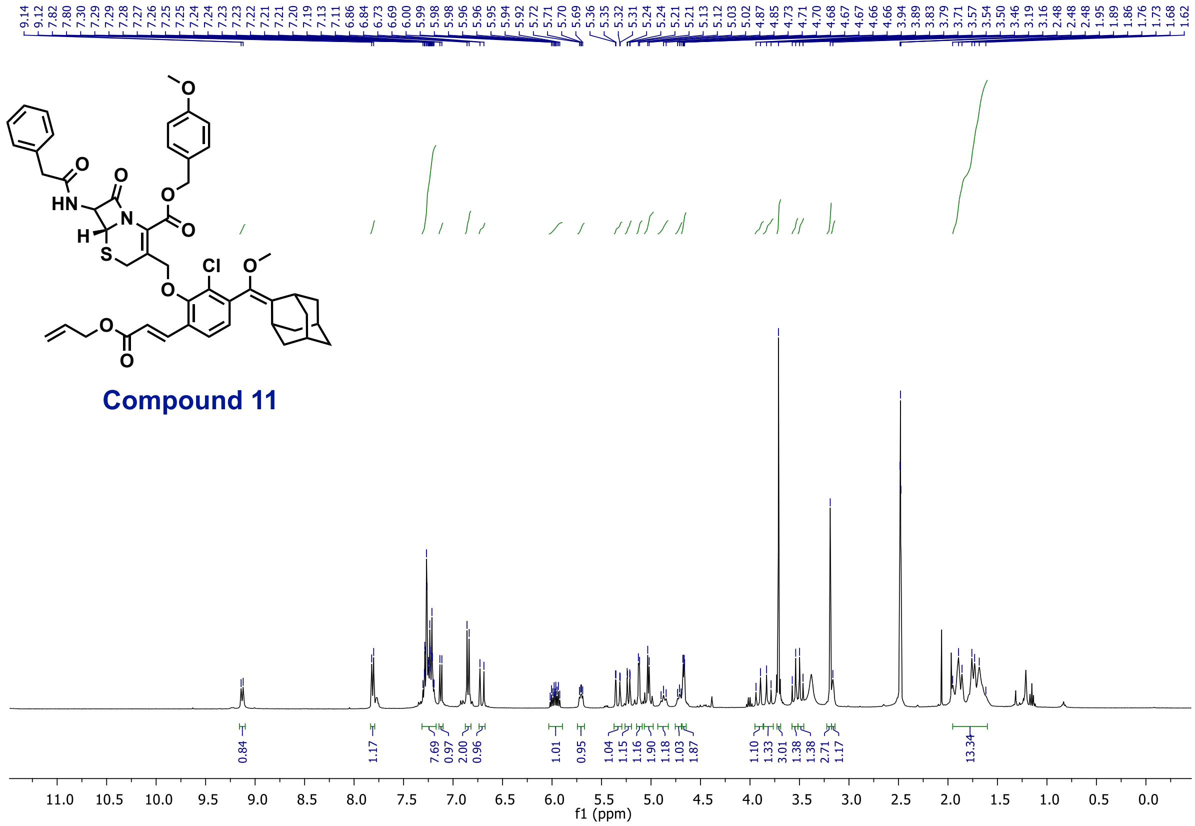

Figure S74. ^{1}H-NMR (400MHz, DMSO) spectra of compound **11**.

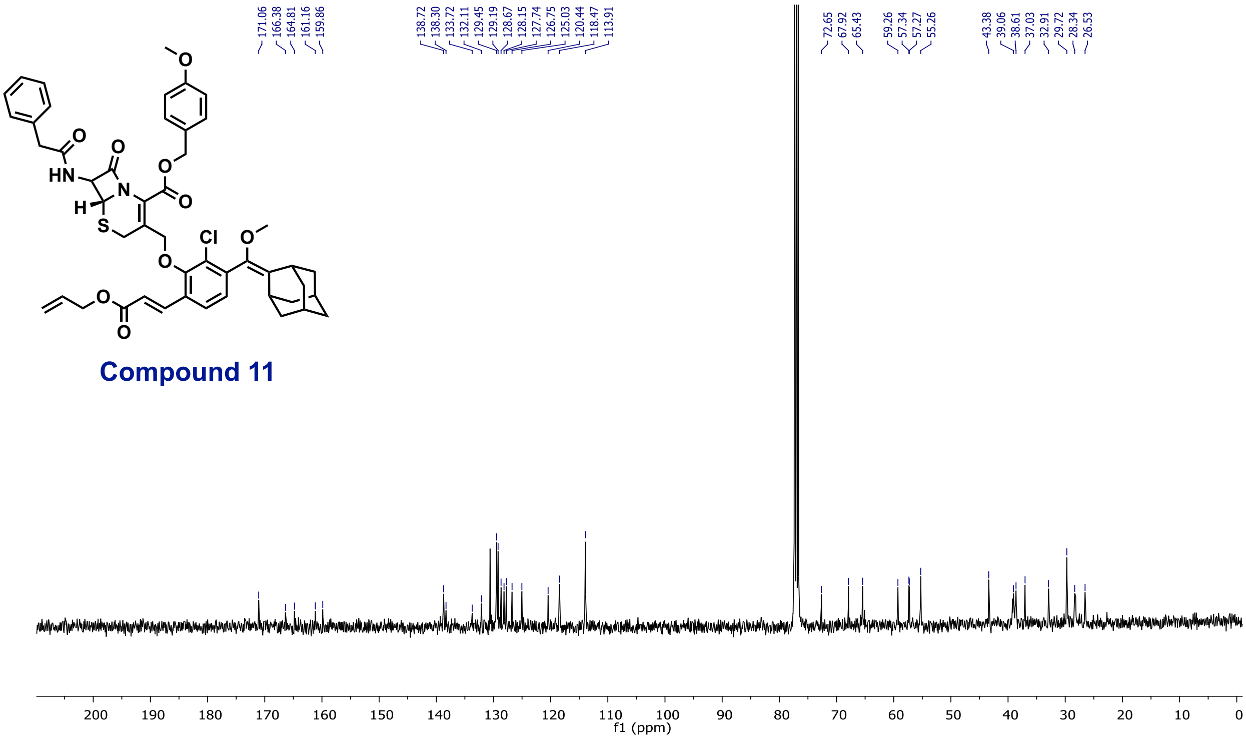

Figure S75. ^{13}C-NMR (100 MHz, $CDCl_3$) spectra of compound **11**.

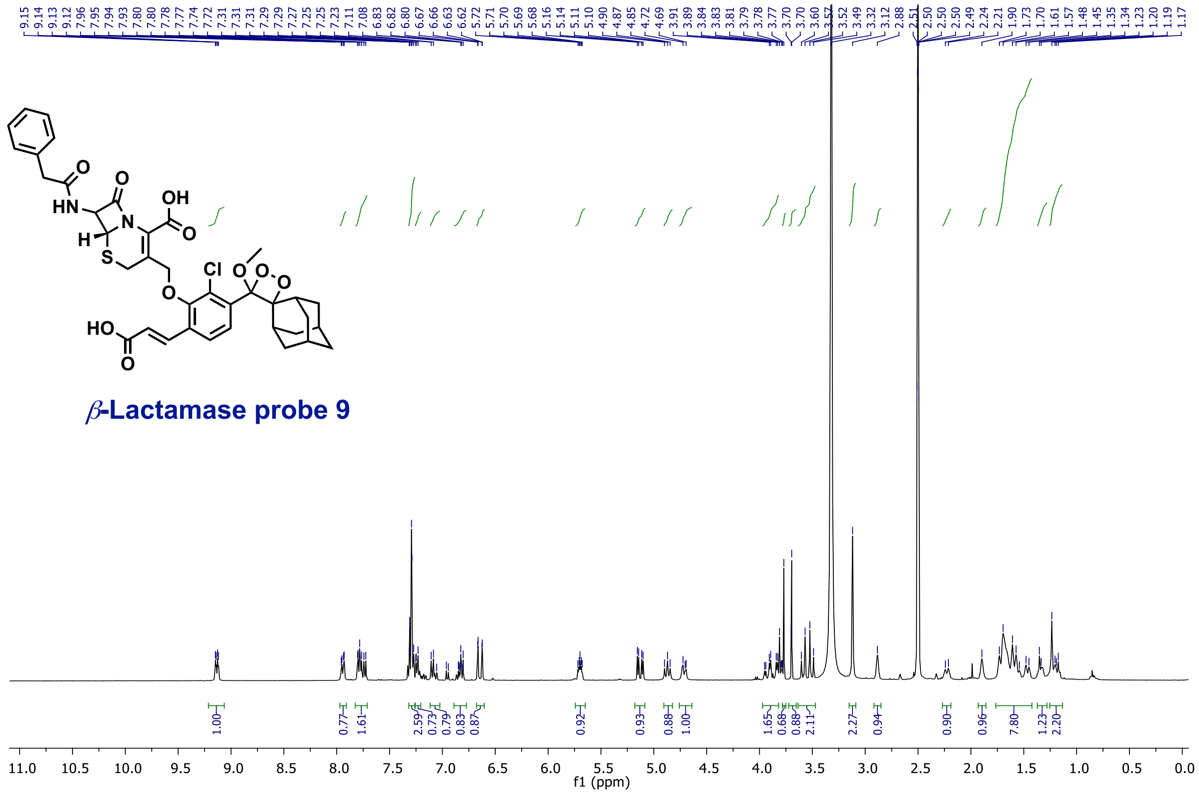

Figure S76. ^{1}H-NMR (400MHz, DMSO) spectra of ***β*-lactamase probe 9**.

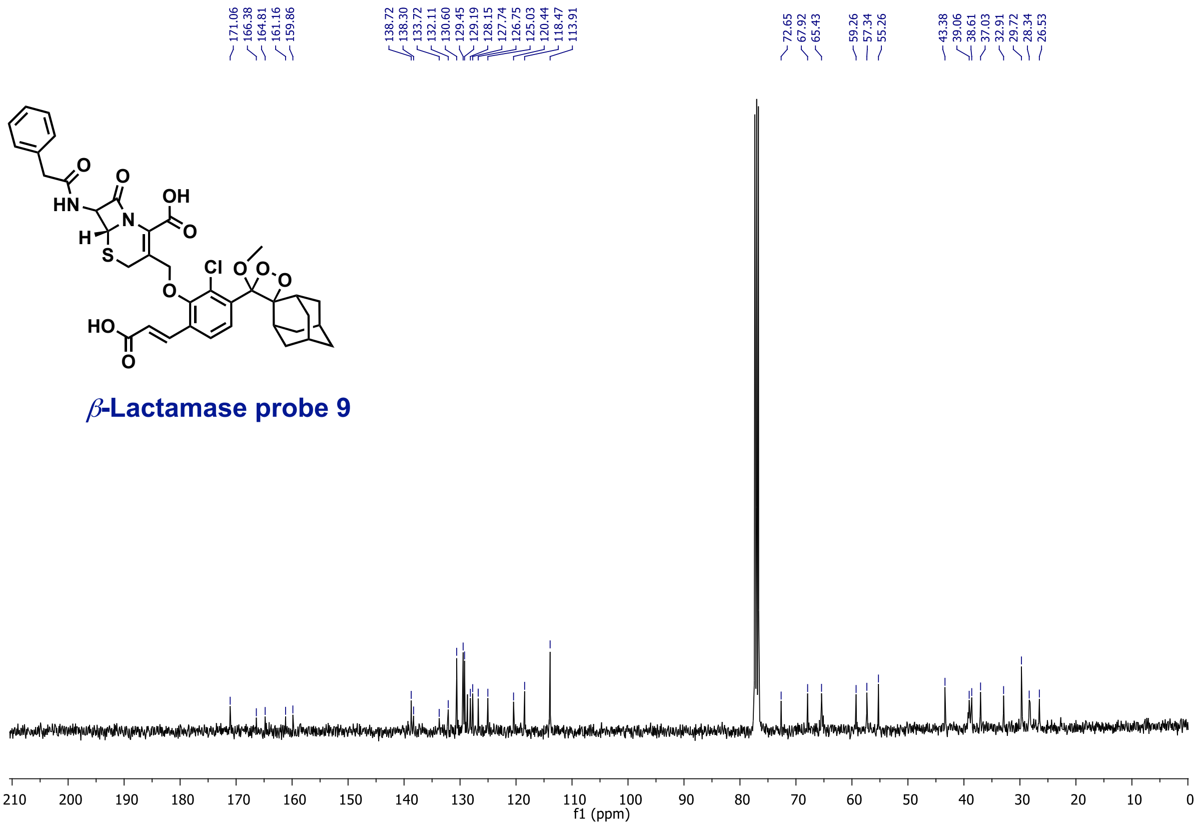

Figure S77. ^{13}C-NMR (100 MHz, $CDCl_3$) spectra of ***β*-lactamase probe 9**.

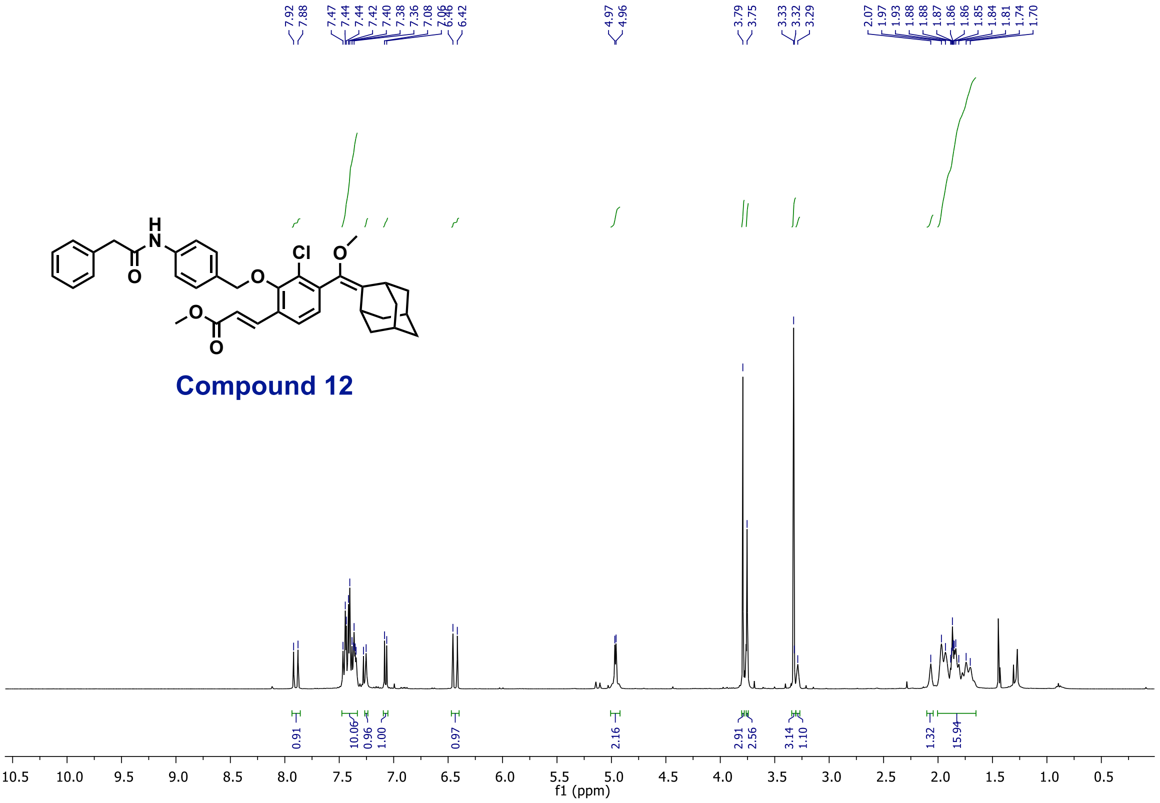

Figure S78. ^{1}H-NMR (400MHz, $CDCl_3$) spectra of compound **12**.

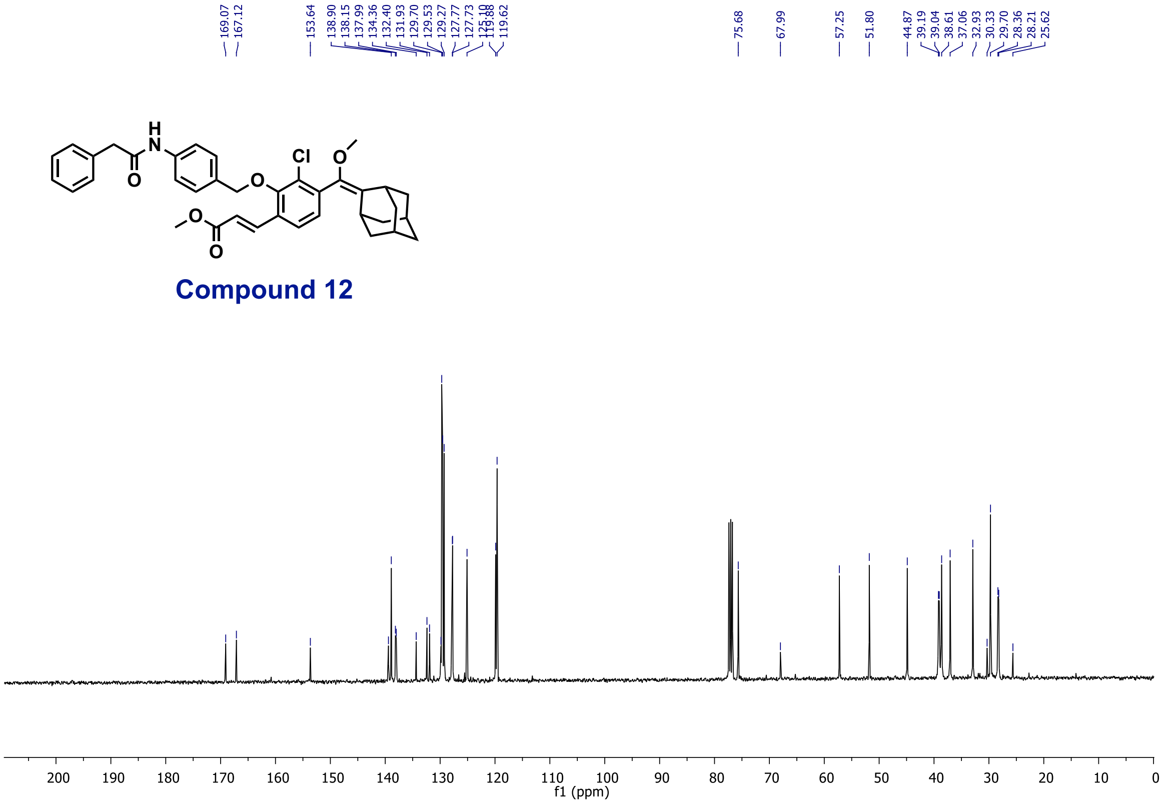

Figure S79. ^{13}C-NMR (100 MHz, $CDCl_3$) spectra of compound **12**.

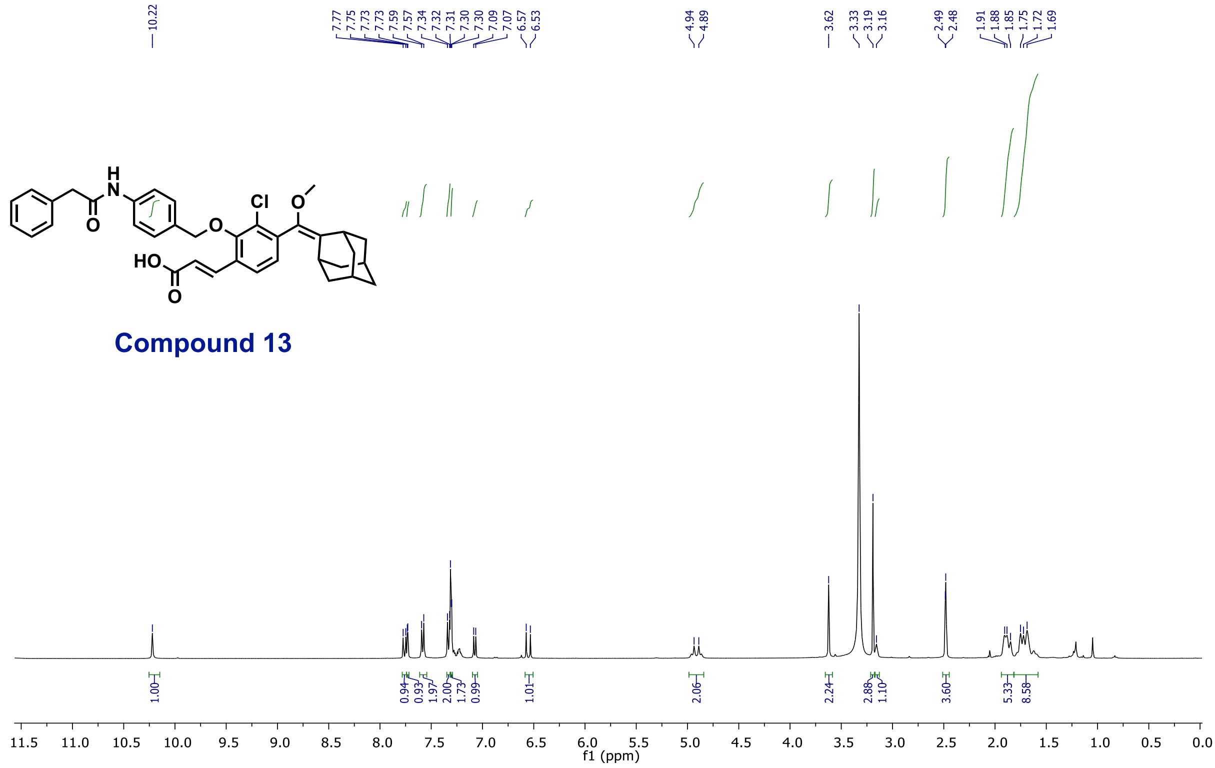

Figure S80. ^{1}H-NMR (400MHz, DMSO) spectra of compound **13**.

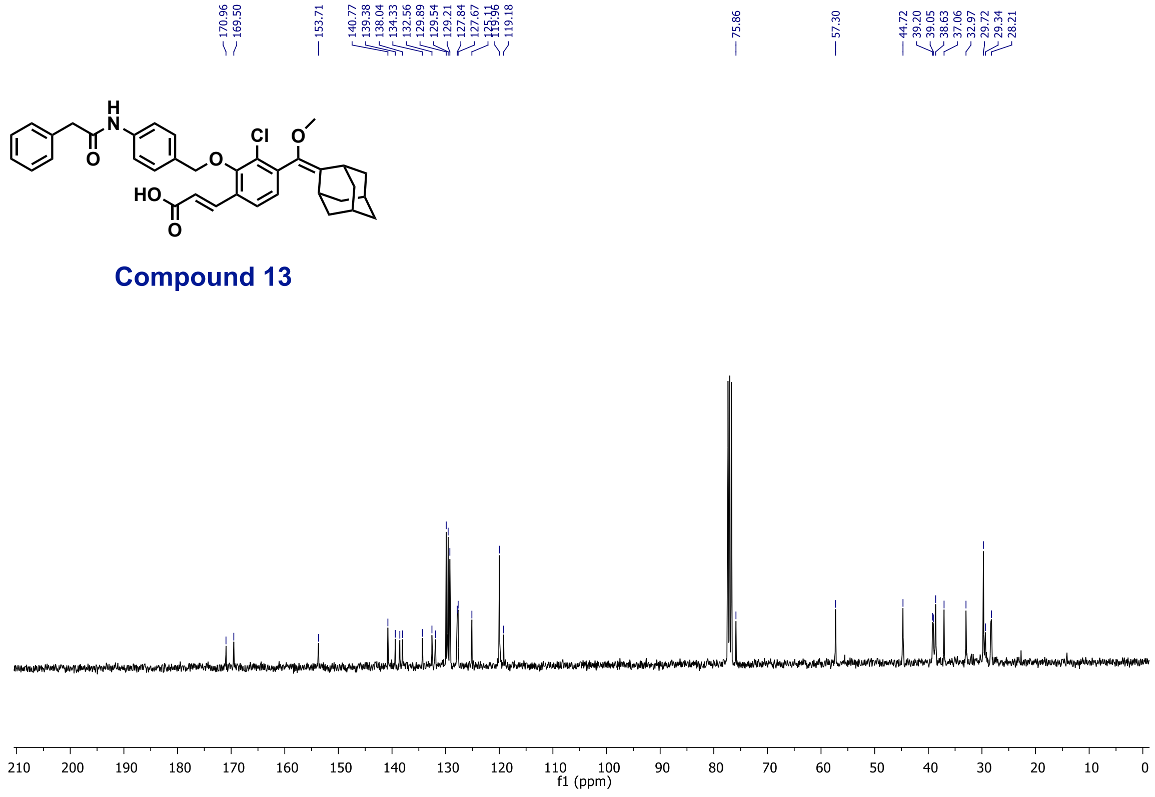

Figure S81. ^{13}C-NMR (100 MHz, $CDCl_3$) spectra of compound **13**.

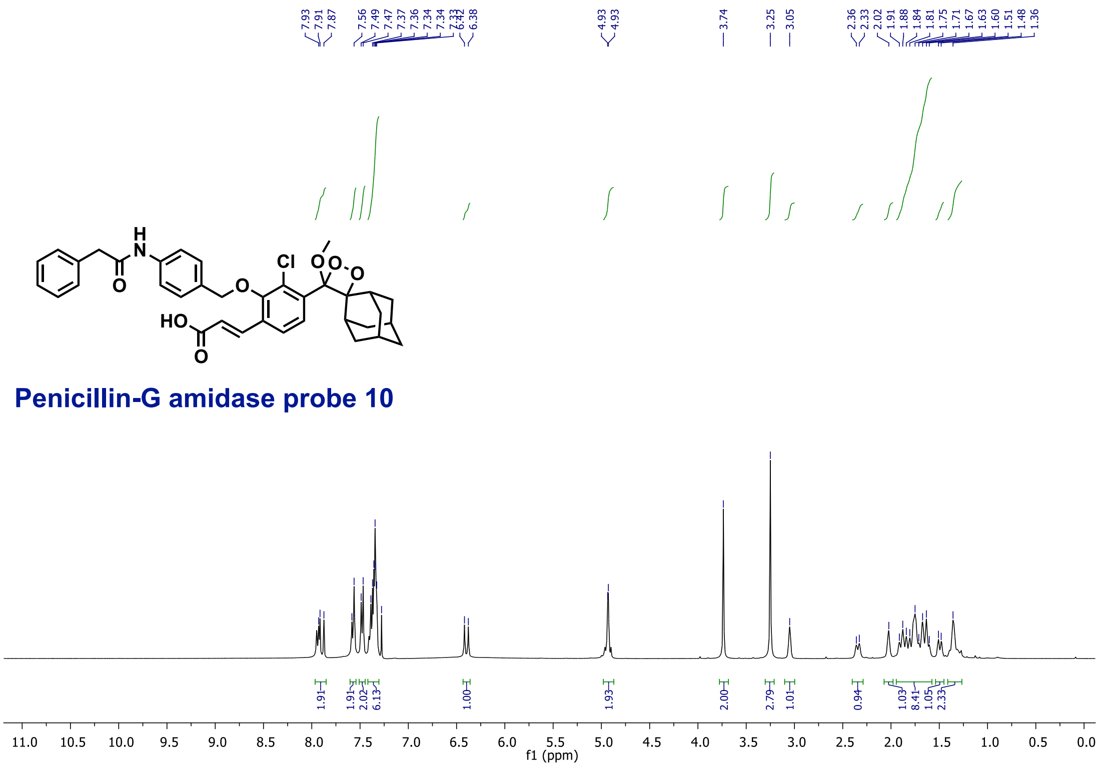

Penicillin-G amidase probe 10

Figure S82. ^{1}H-NMR (400MHz, $CDCl_3$) spectra of **Penicillin-G amidase probe 10**.

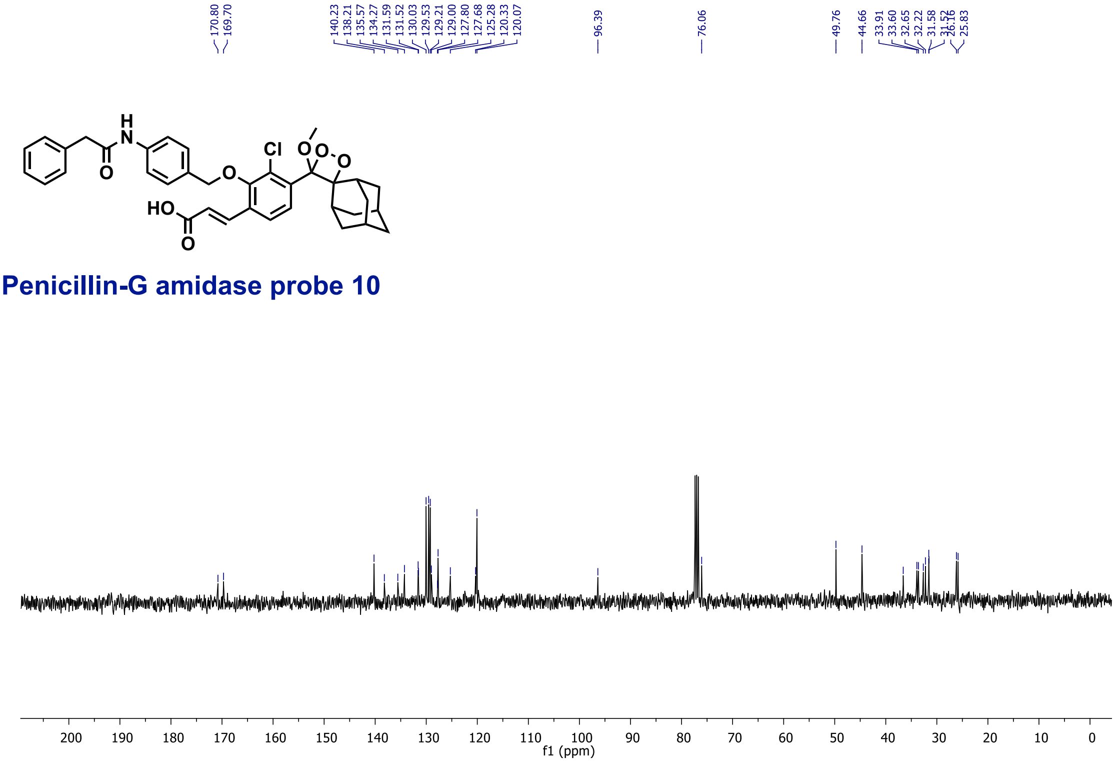

Penicillin-G amidase probe 10

Figure S83. ^{13}C-NMR (100 MHz, $CDCl_3$) spectra of **Penicillin-G amidase probe 10**.

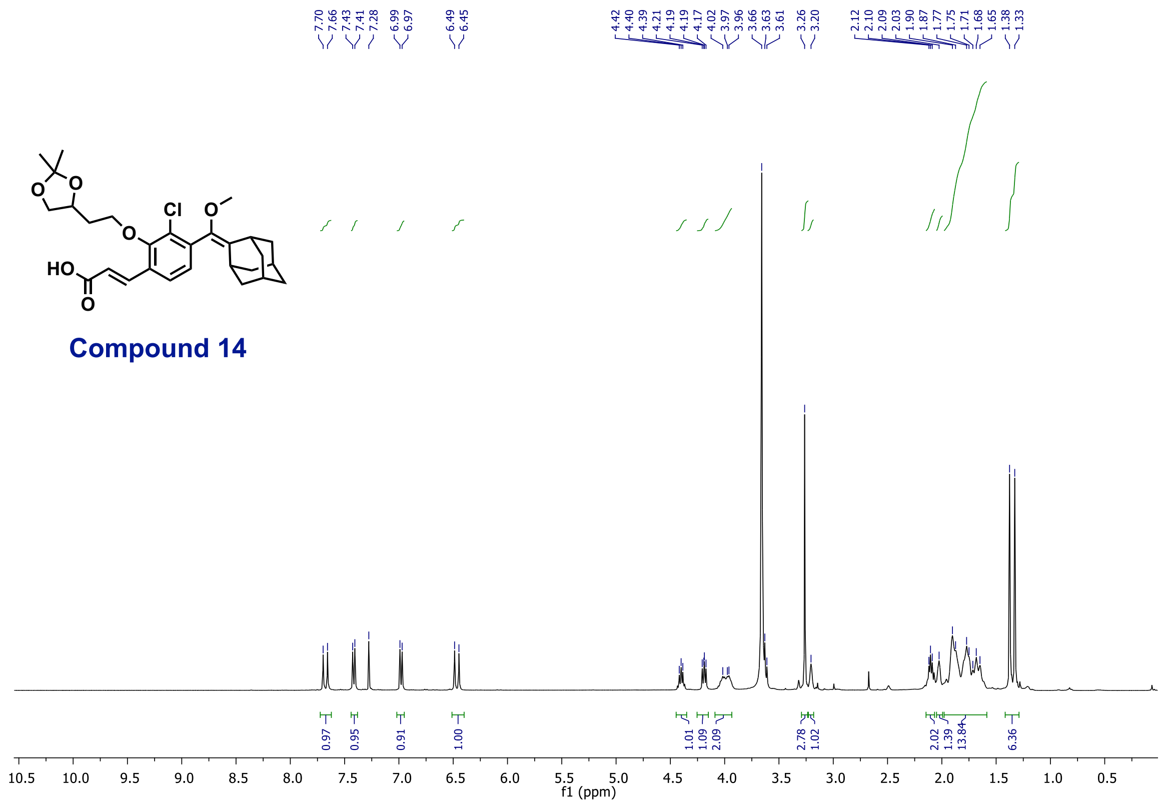

Figure S84. ^{1}H-NMR (400MHz, $CDCl_3$) spectra of compound **14**.

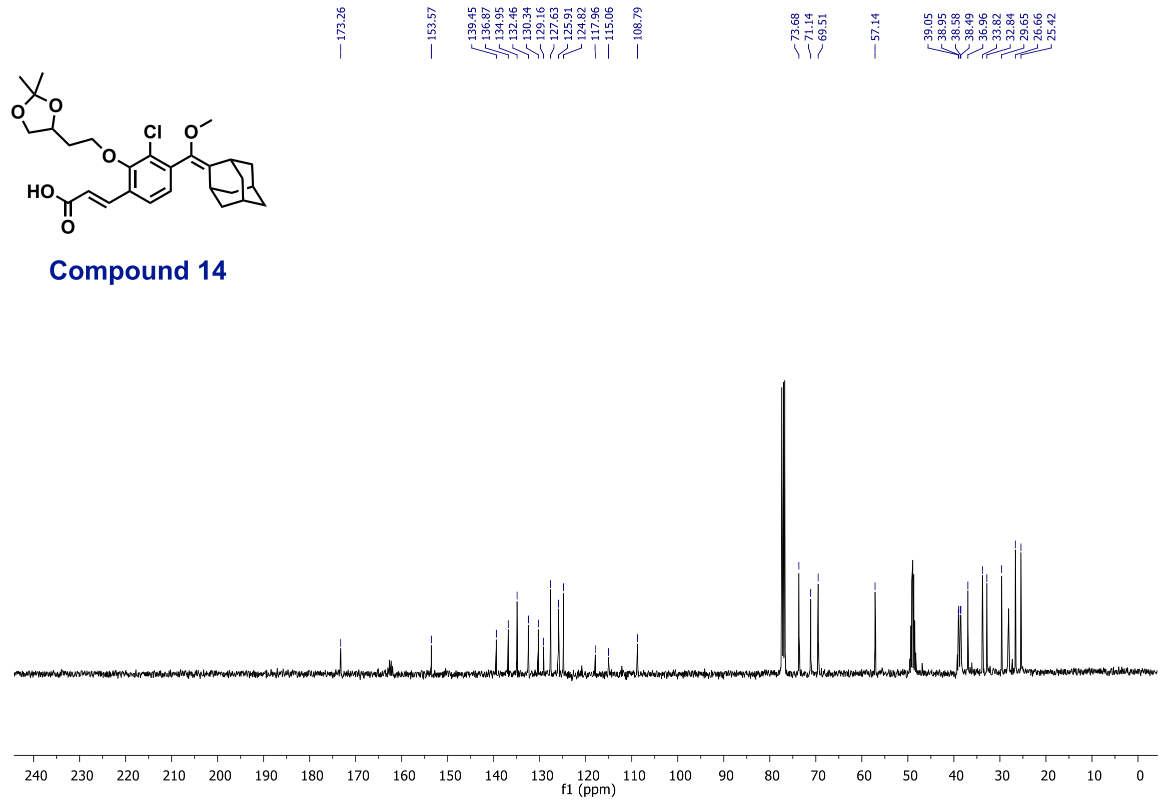

Figure S85. ^{13}C-NMR (100 MHz, $CDCl_3$) spectra of compound **14**.

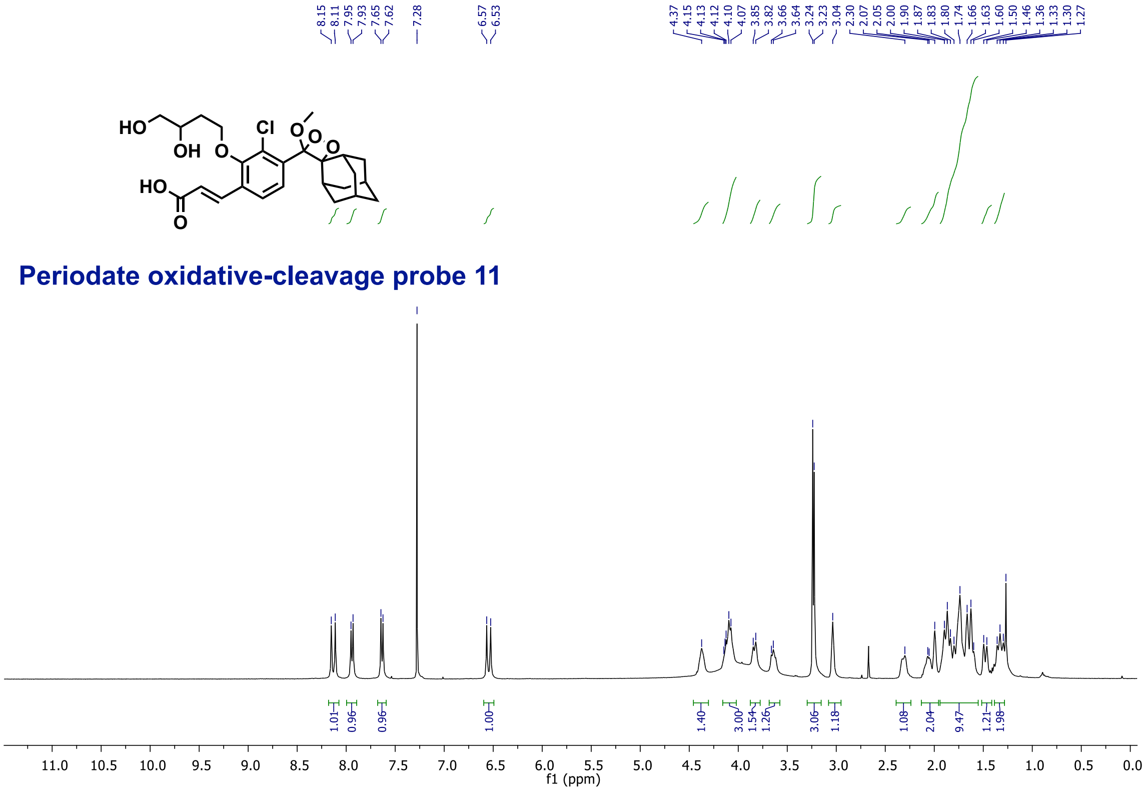

Figure S86. ^{1}H-NMR (400MHz, $CDCl_3$) spectra of **Periodate oxidative-cleavage probe 11**.

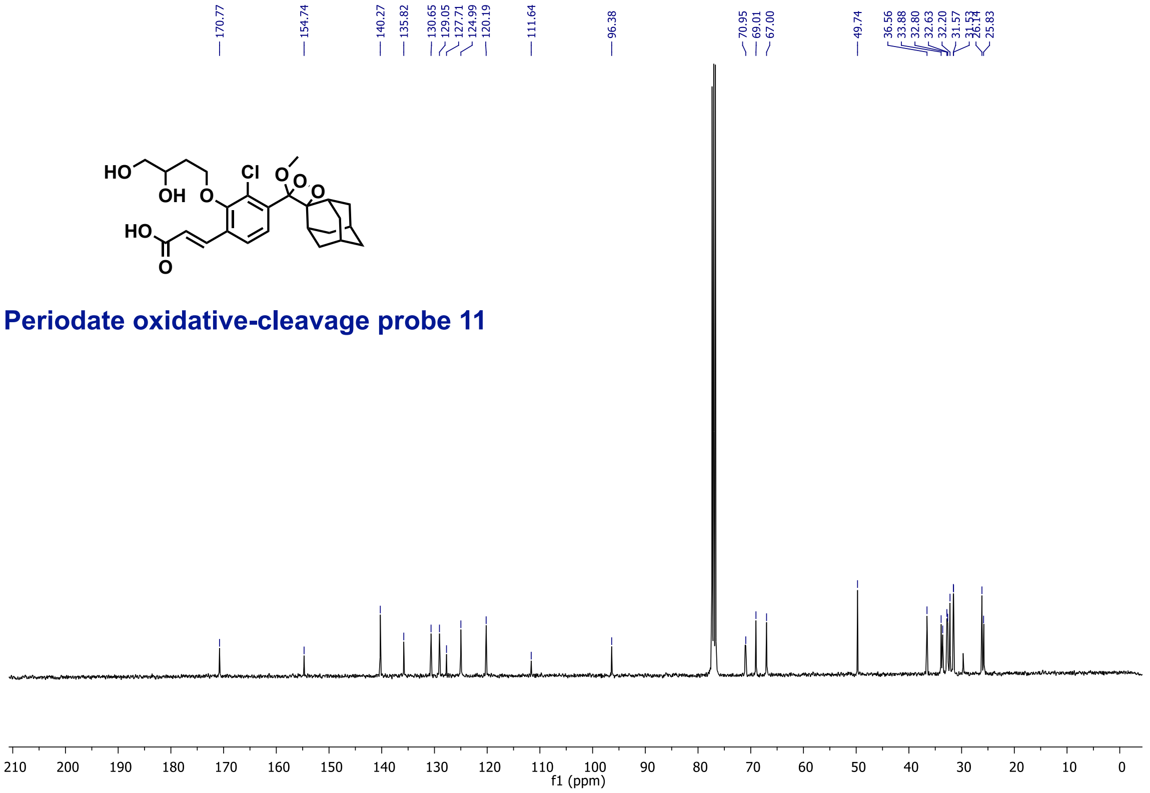

Figure S87. ^{13}C-NMR (100MHz, $CDCl_3$) spectra of **Periodate oxidative-cleavage probe 11**.

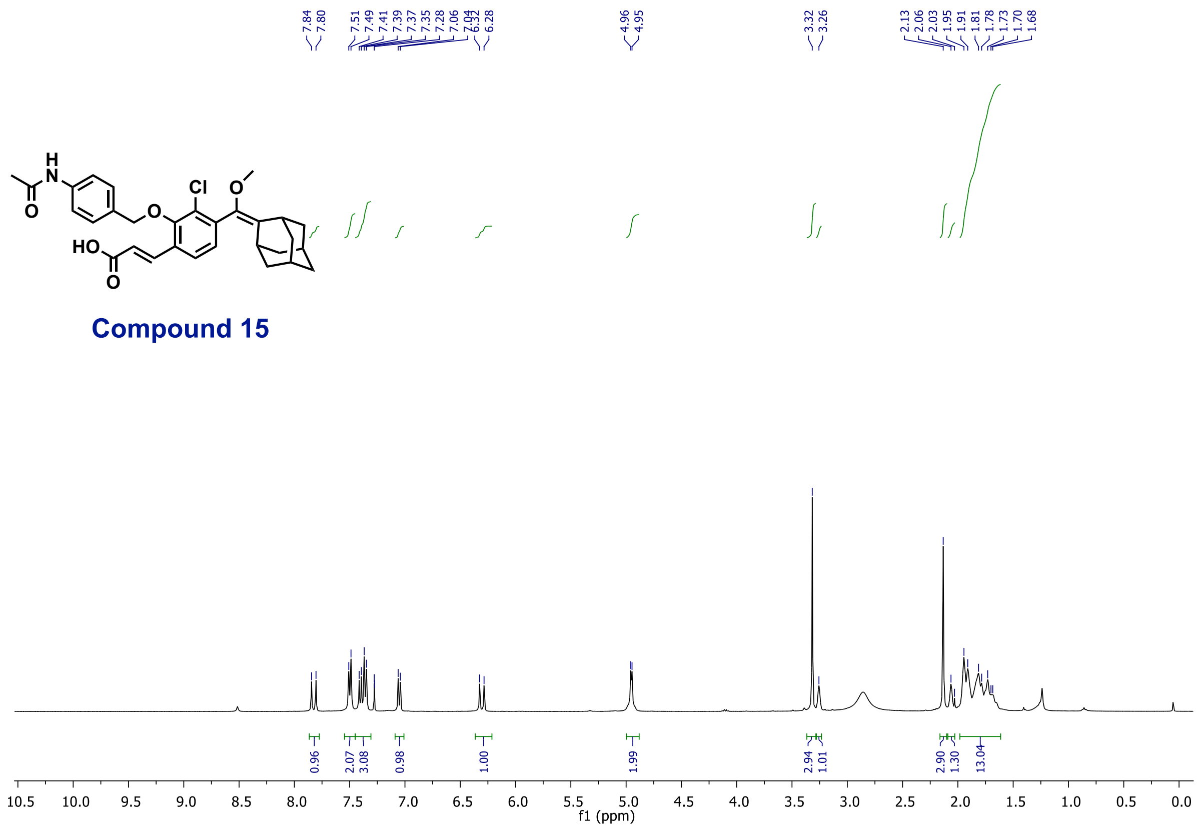

Figure S88. ^{1}H-NMR (400MHz, $CDCl_3$) spectra of compound **15**.

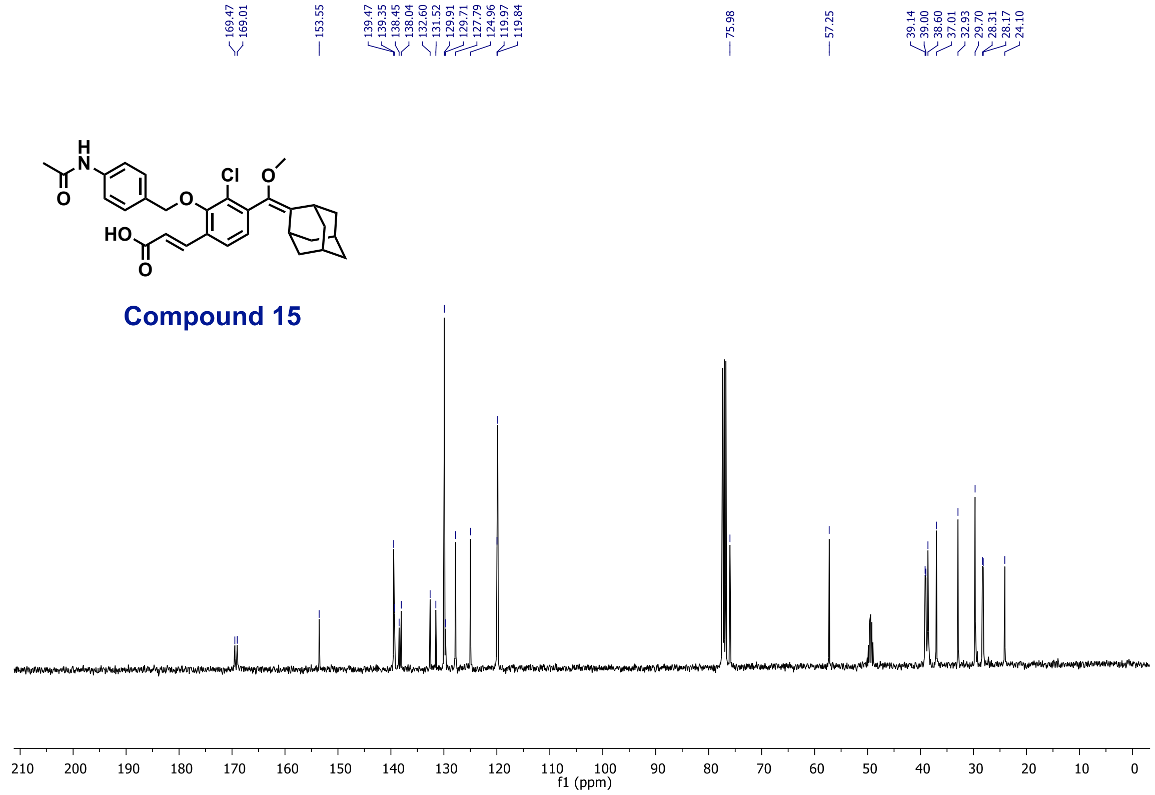

Figure S89. ^{13}C-NMR (100 MHz, $CDCl_3$) spectra of compound **15**.

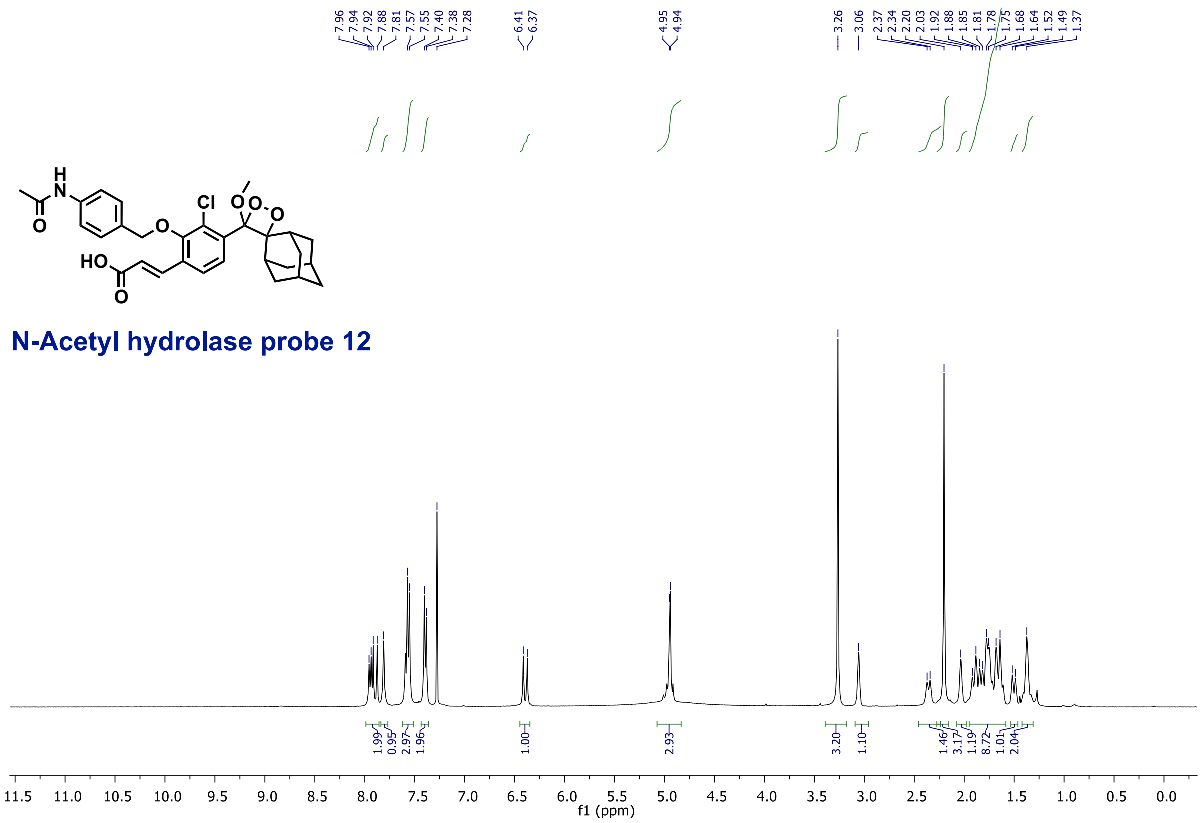

Figure S90. ^{1}H-NMR (400MHz, $CDCl_3$) spectra of **N-acetyl hydrolase probe 12**.

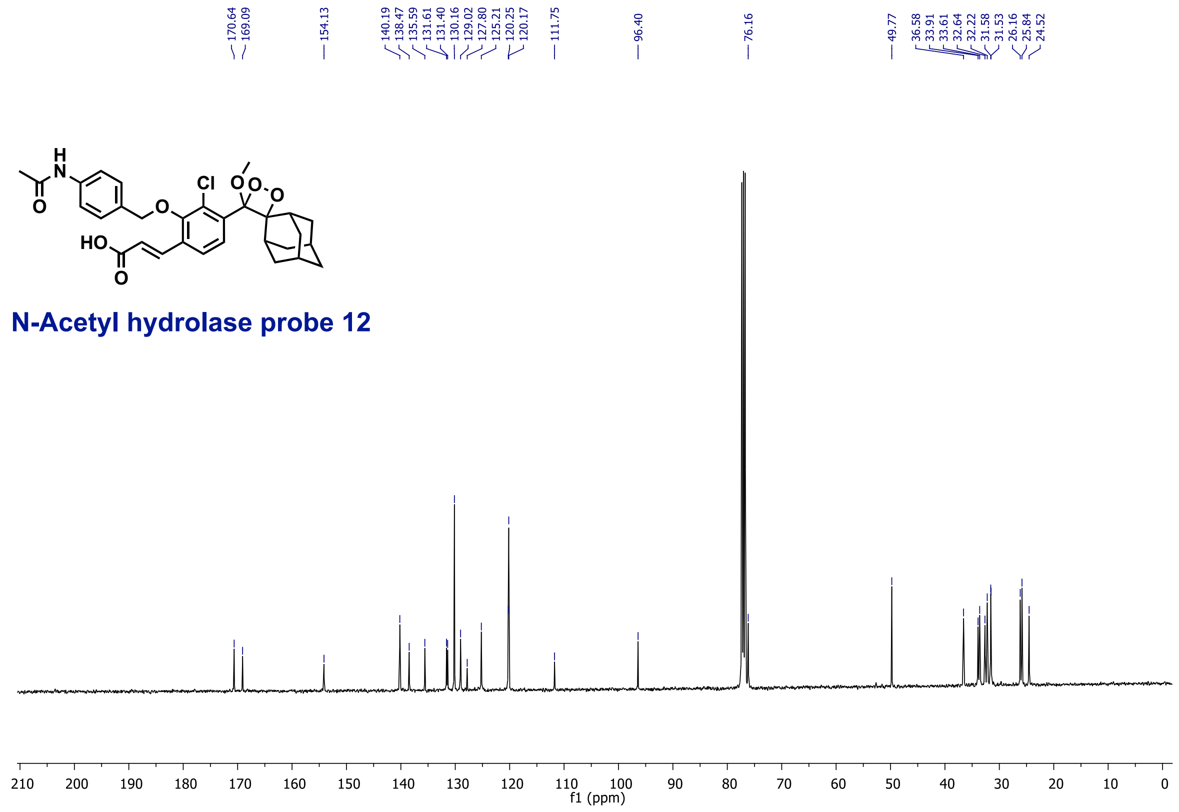

Figure S91. ^{13}C-NMR (100 MHz, $CDCl_3$) spectra of **N-acetyl hydrolase probe 12**.

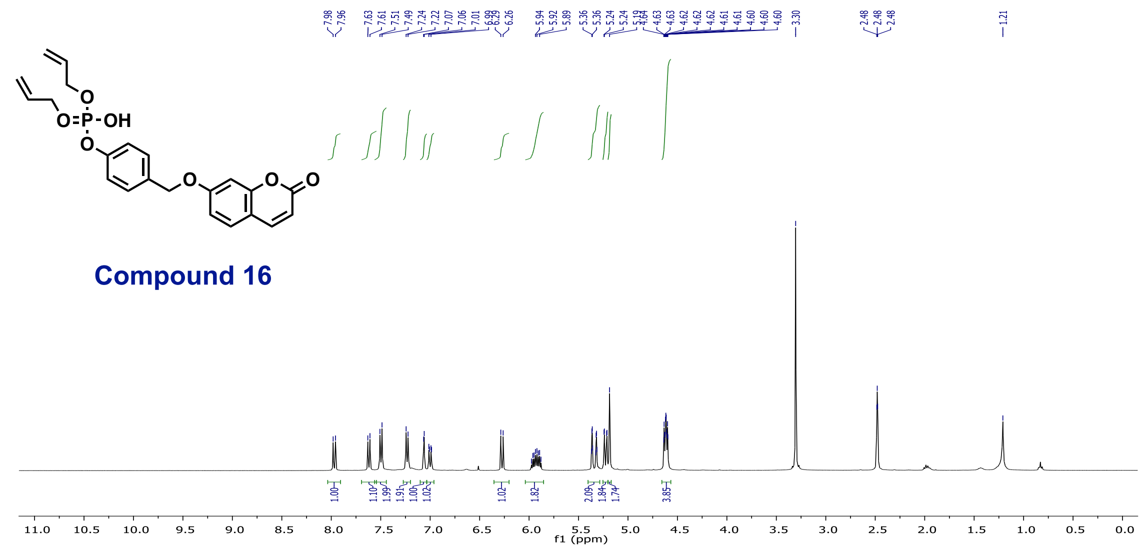

Figure S92. ^{1}H-NMR (400MHz, DMSO) spectra of compound **16**.

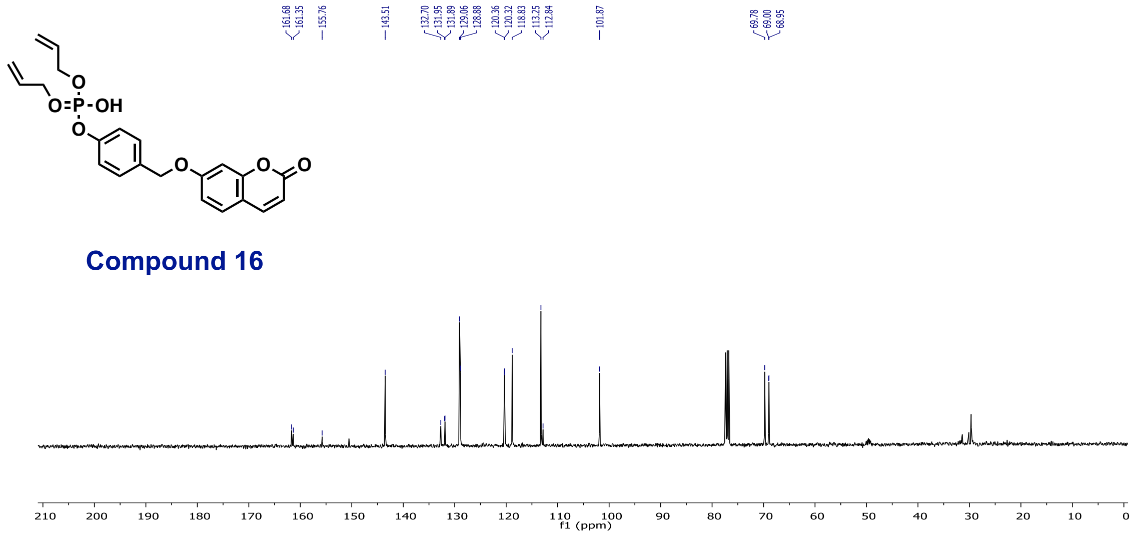

Figure S93. ^{13}C-NMR (100 MHz, $CDCl_3$) spectra of compound **16**.

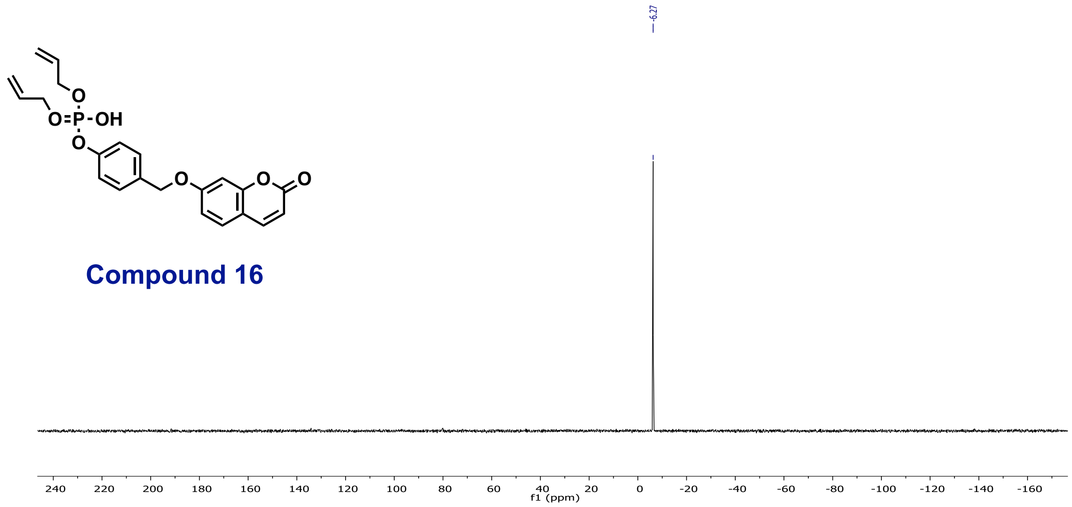

Figure S94. ^{31}P-NMR (100 MHz, DMSO) spectra of compound **16**.

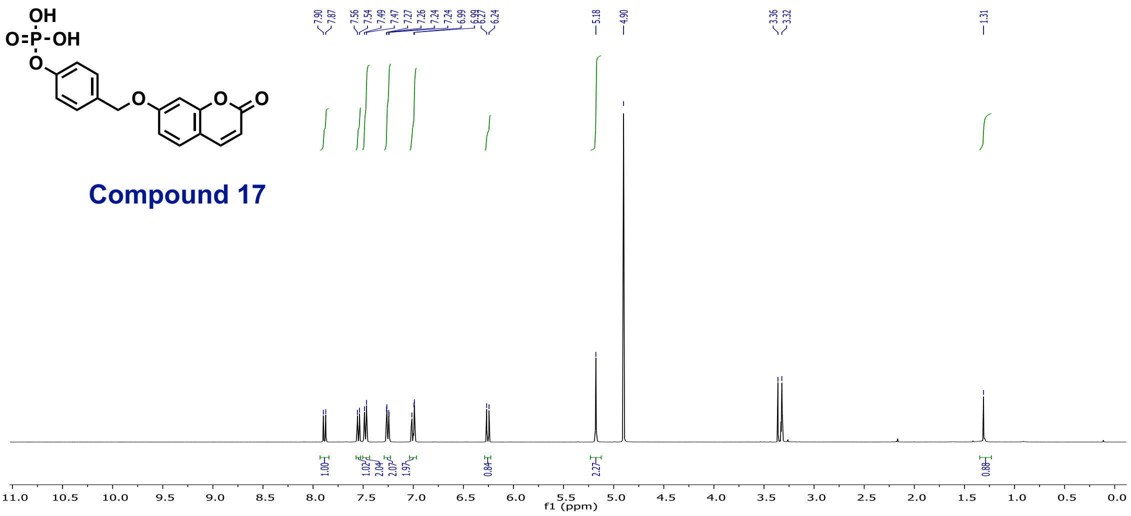

Figure S95. ^{1}H-NMR (400MHz, MeOD) spectra of compound **17**.

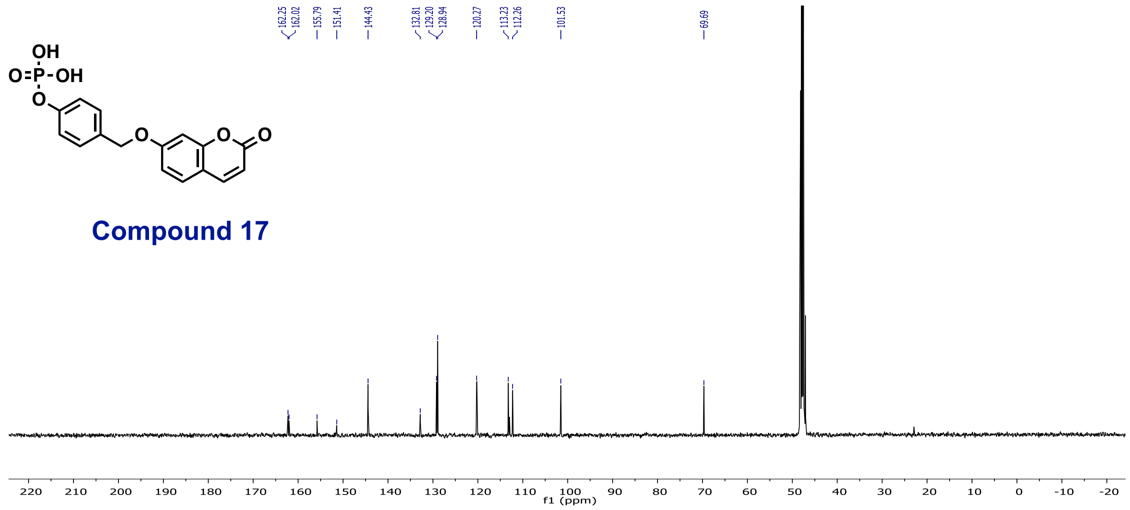

Figure S96. ^{13}C-NMR (100 MHz, MeOD) spectra of compound **17**.

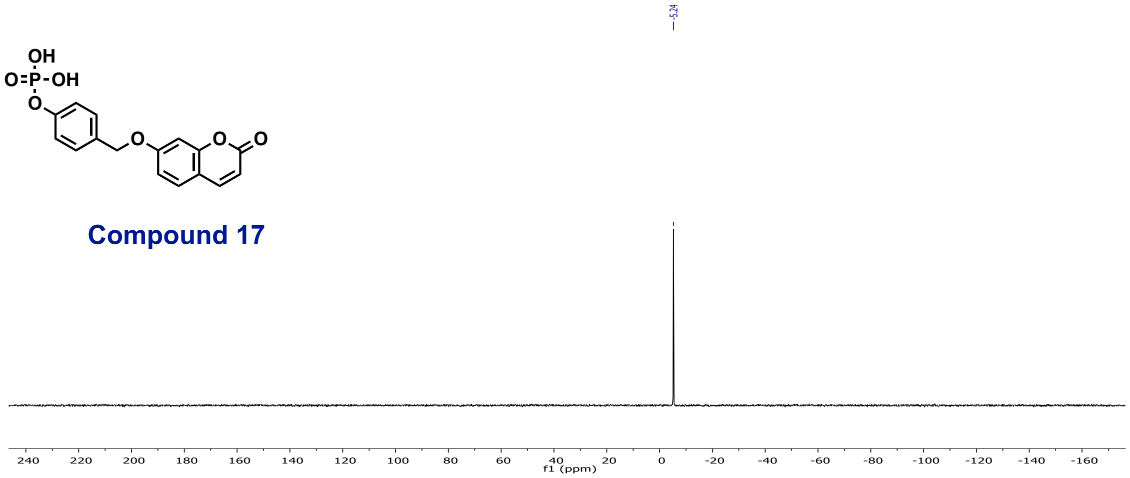

Figure S97. ^{31}P-NMR (162 MHz, MeOD) spectra of compound **17**.

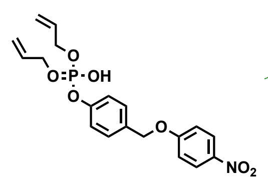

Compound 18

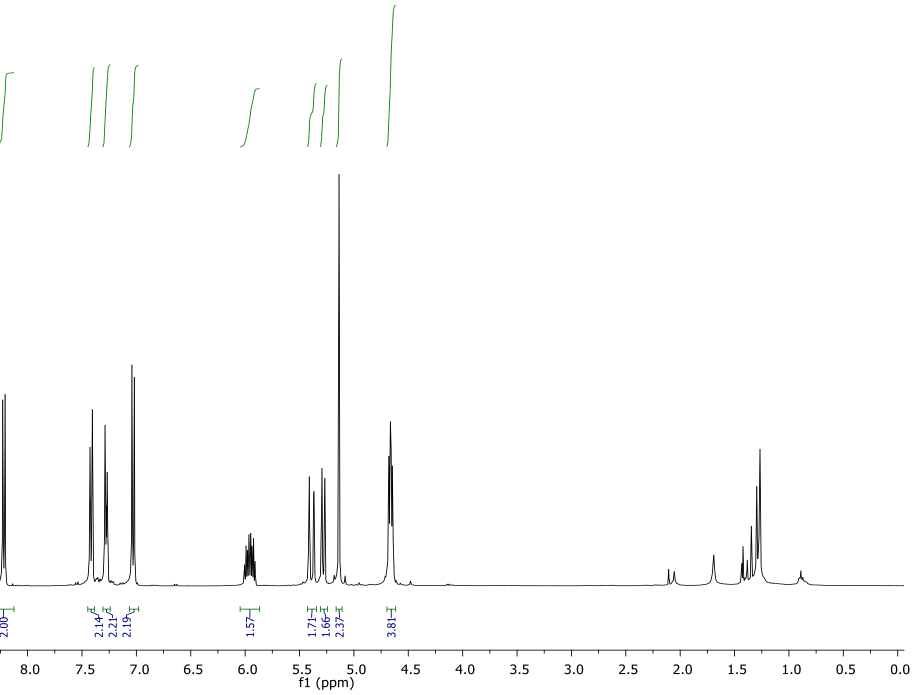

Figure S98. ^{1}H-NMR (400MHz, $CDCl_3$) spectra of compound **18**.

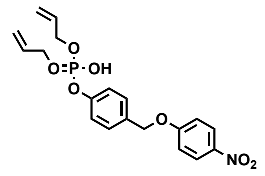

Compound 18

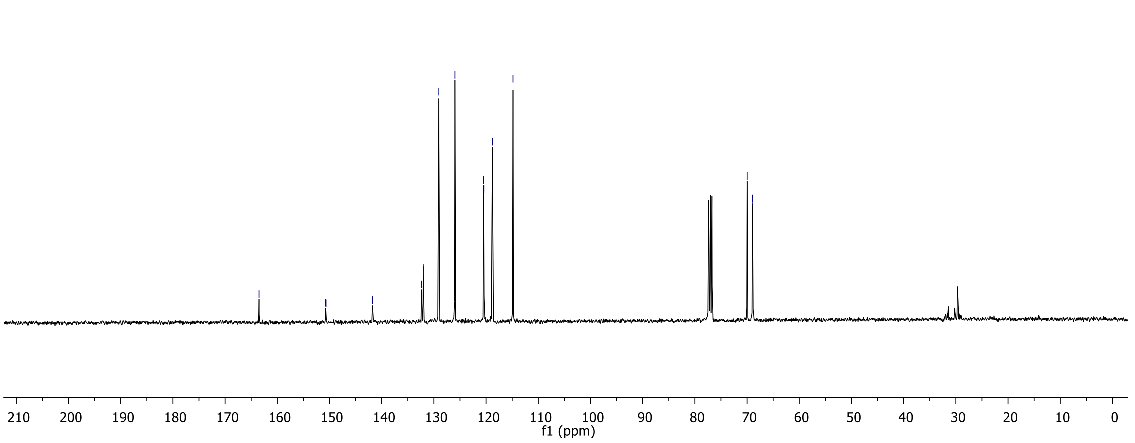

Figure S99. ^{13}C-NMR (100 MHz, $CDCl_3$) spectra of compound **18**.

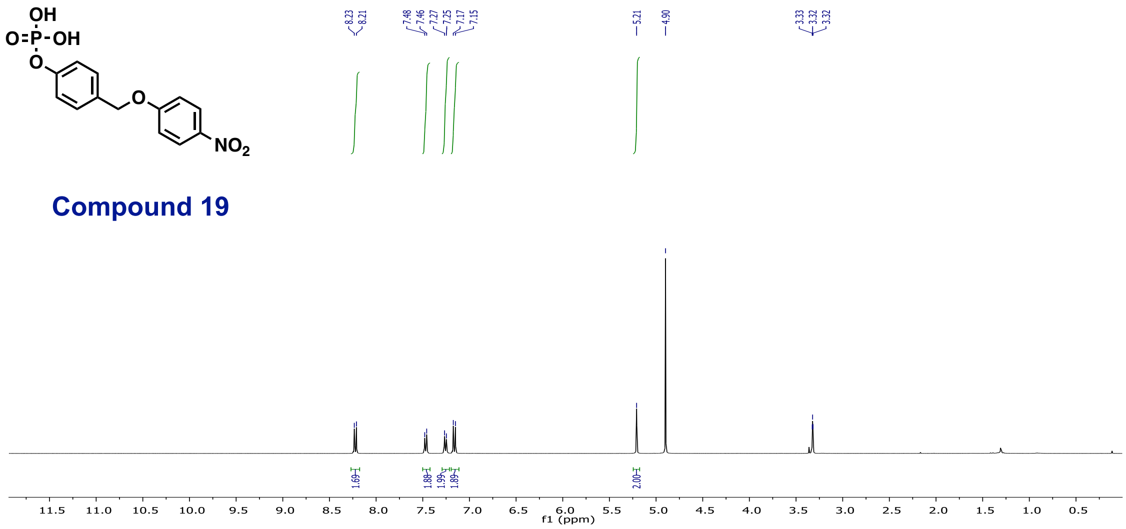

Figure S100. ^{1}H-NMR (400MHz, MeOD) spectra of compound **19**.

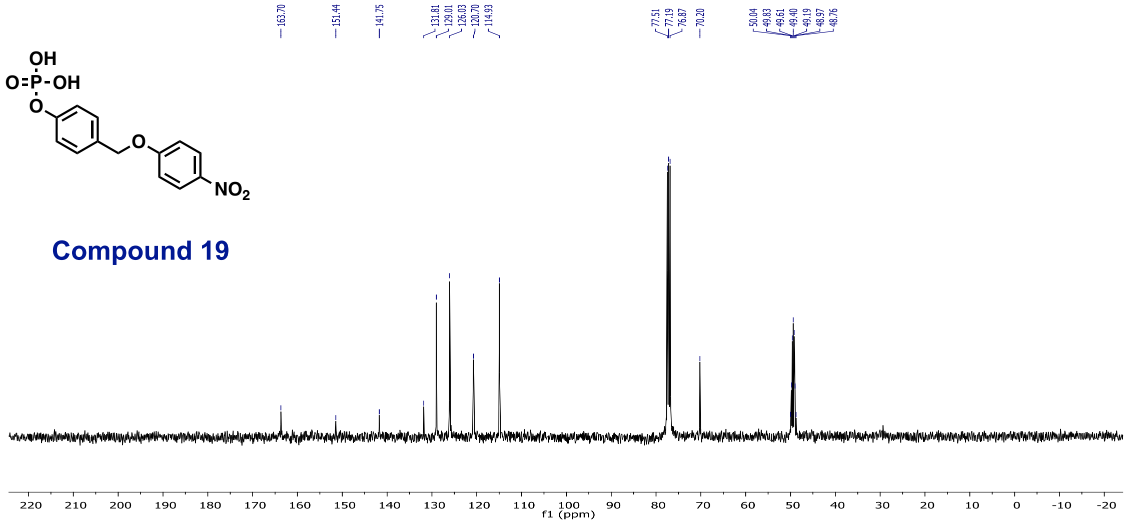

Figure S101. ^{13}C-NMR (100 MHz, MeOD) spectra of compound **19**.

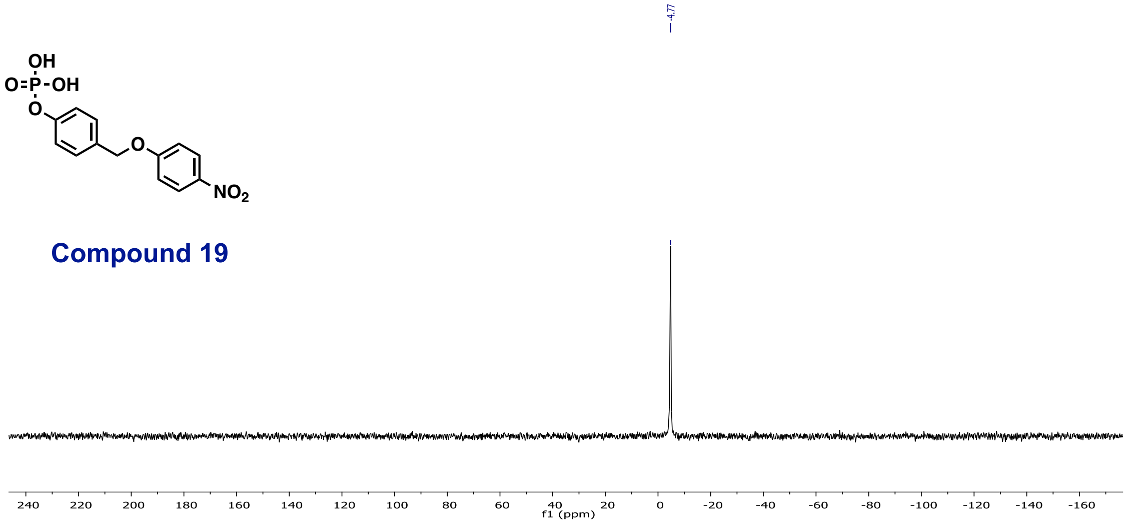

Figure S102. ^{31}P-NMR (162 MHz, MeOD) spectra of compound **19**.

HPLC Spectra of Key Compounds

compound 2

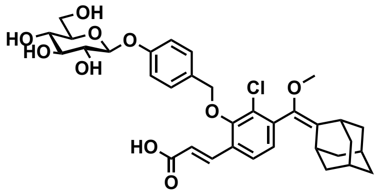

Compound 2

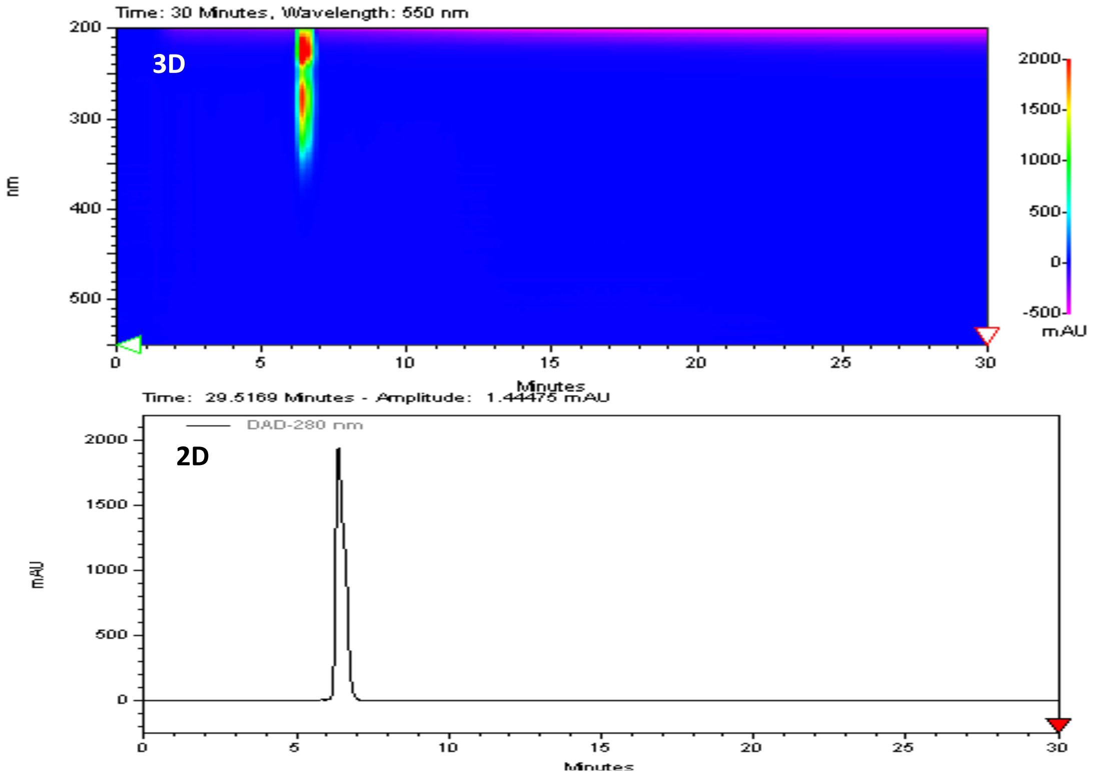

Figure S103. HPLC spectra (70-100% H_2O[0.1%TFA]:ACN gradient) of compound **2** (2D Absorbance measured at 280nm).

β-glucosidase probe 1

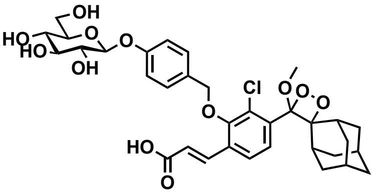

β-Glucosidase probe 1

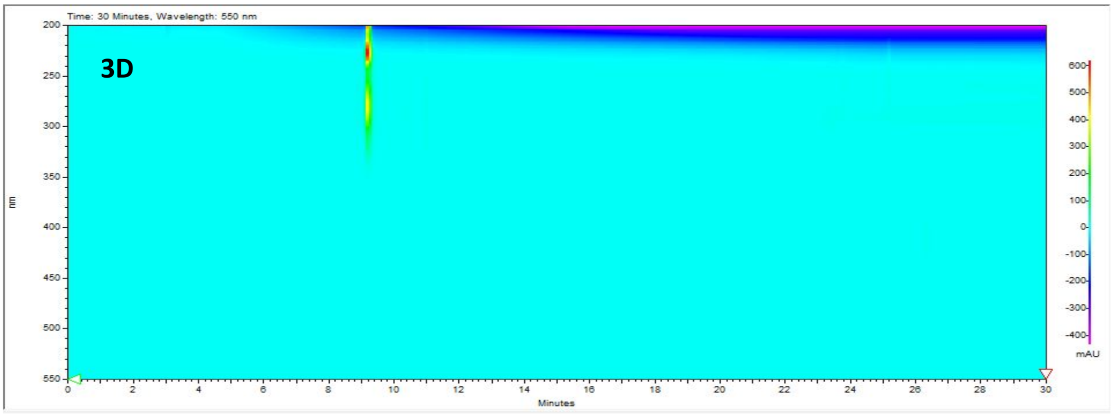

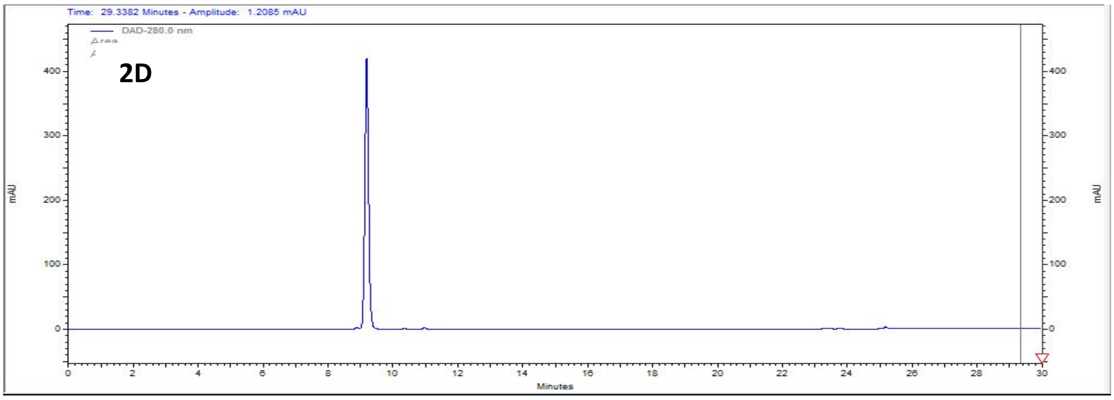

Figure S104. HPLC spectra (70-100% H_2O[0.1%TFA]:ACN gradient) of ***β*-glucosidase probe 1** (2D HPLC spectra absorbance measured at 280nm).

Compound 5

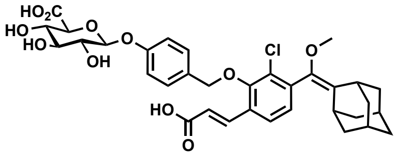

Compound 5

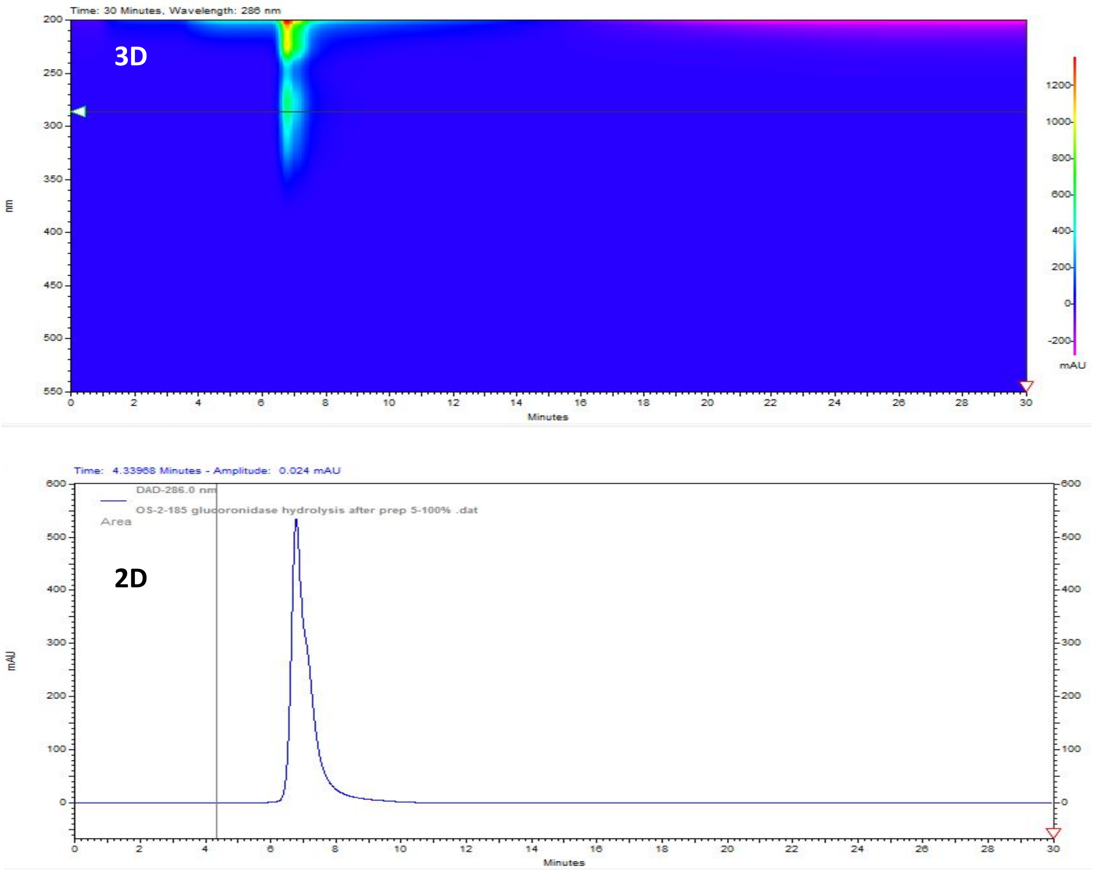

Figure S105. HPLC spectra (30-100% H_2O[0.1%TFA]:ACN gradient) of Compound **5** (2D HPLC spectra absorbance measured at 280nm).

β-glucuronidase probe 2

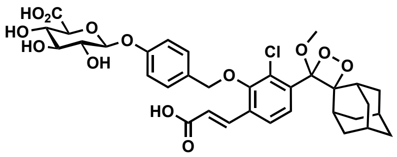

β-Glucuronidase probe 2

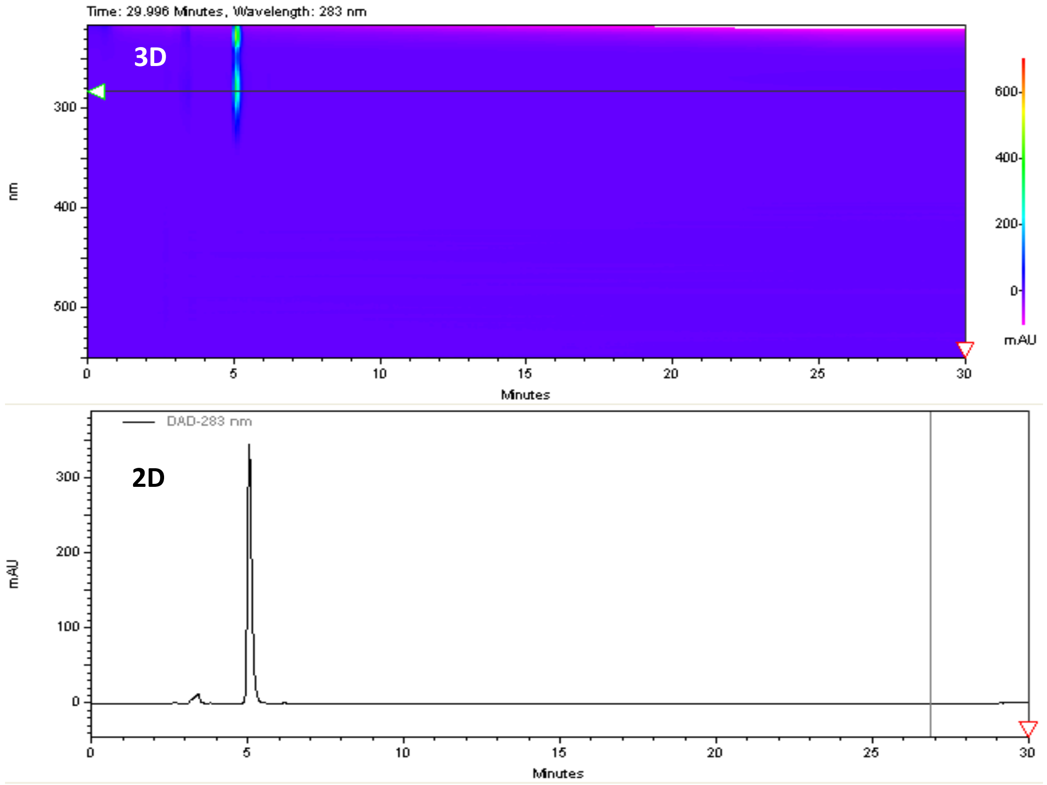

Figure S106. HPLC spectra (30-100% H_2O[0.1%TFA]:ACN gradient) of ***β*-glucuronidase probe 2** (2D HPLC spectra absorbance measured at 280nm).

*The minor peak in HPLC spectra belongs to the benzoate ester, the dioxetane's thermal decomposition product.

β-galactosidase probe 3

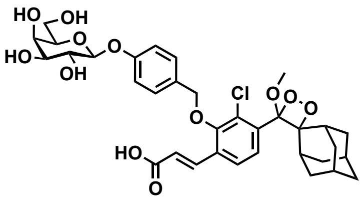

β-Galactosidase probe 3

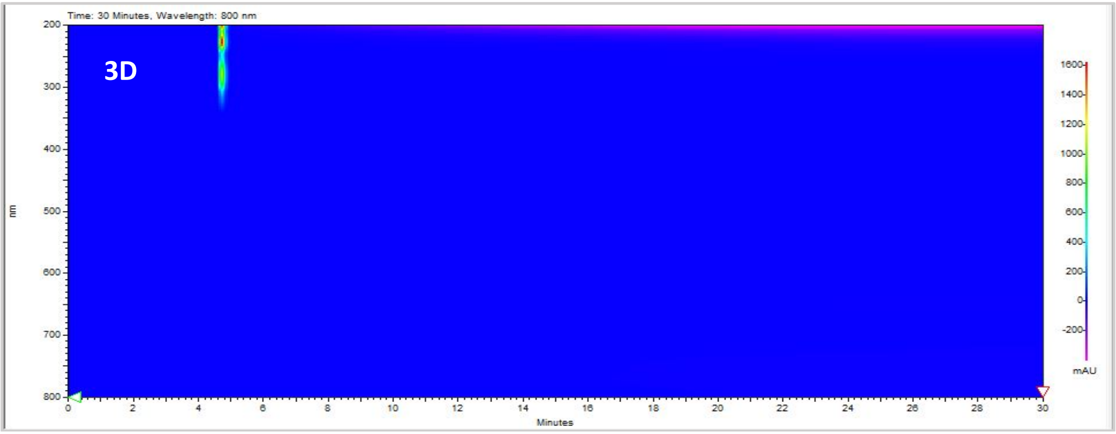

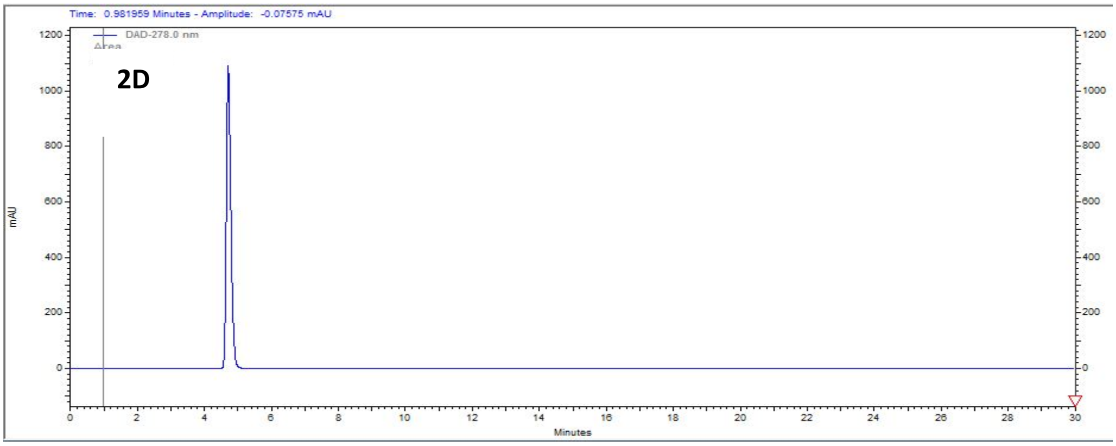

Figure S107. HPLC spectra (70-100% H_2O[0.1%TFA]:ACN gradient) of ***β*-galactosidase probe 3** (2D HPLC spectra absorbance measured at 280nm).

Compound 7

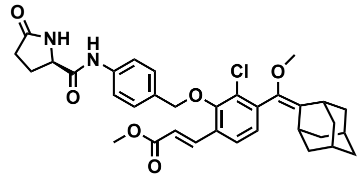

Compound 7

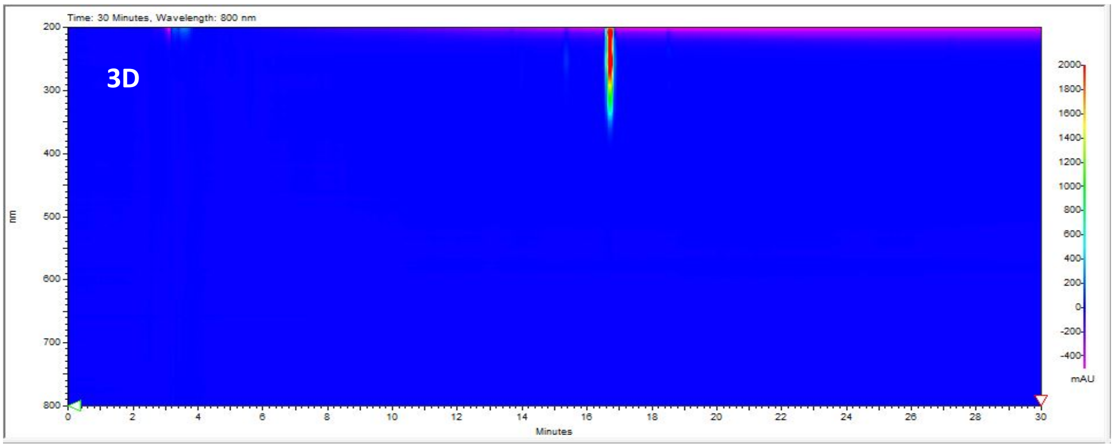

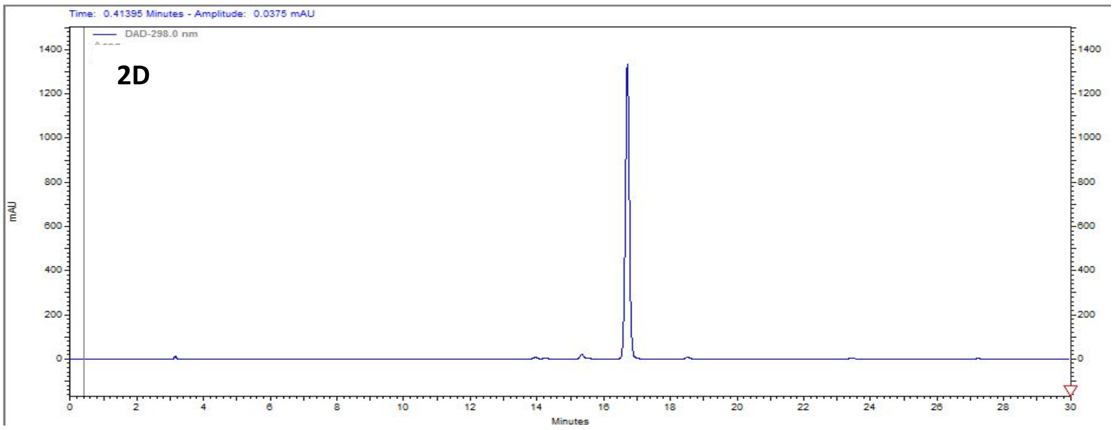

Figure S108. HPLC spectra (50-100% H_2O[0.1%TFA]:ACN gradient) of compound **7** (2D HPLC spectra absorbance measured at 280nm).

Compound 8

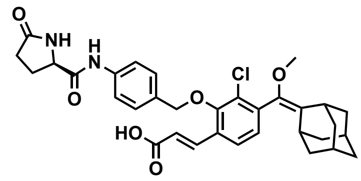

Compound 8

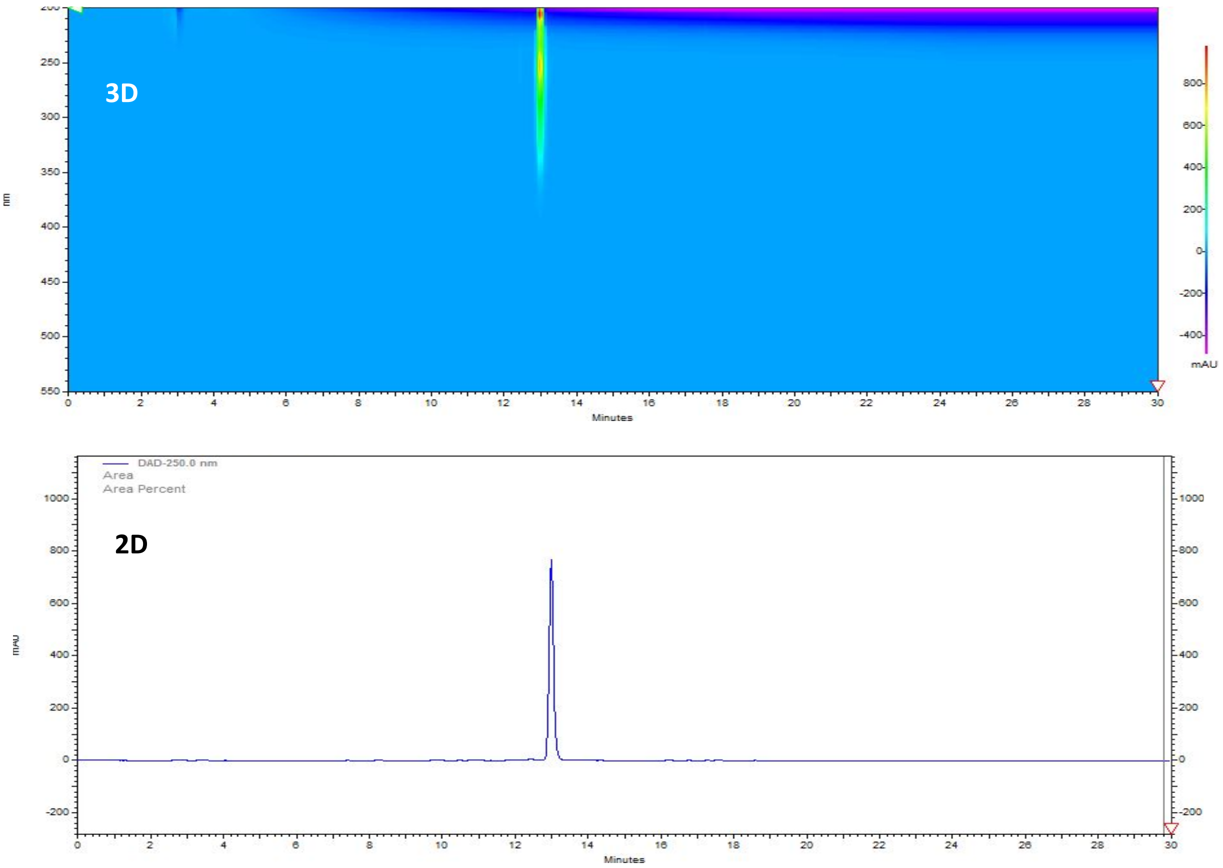

Figure S109. HPLC spectra (50-100% H_2O[0.1%TFA]:ACN gradient) of compound **8** (2D HPLC spectra absorbance measured at 280nm).

Pyroglutamyl aminopeptidase probe 4

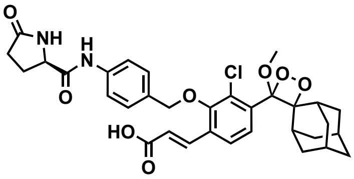

Pyroglutamyl aminopeptidase probe 4

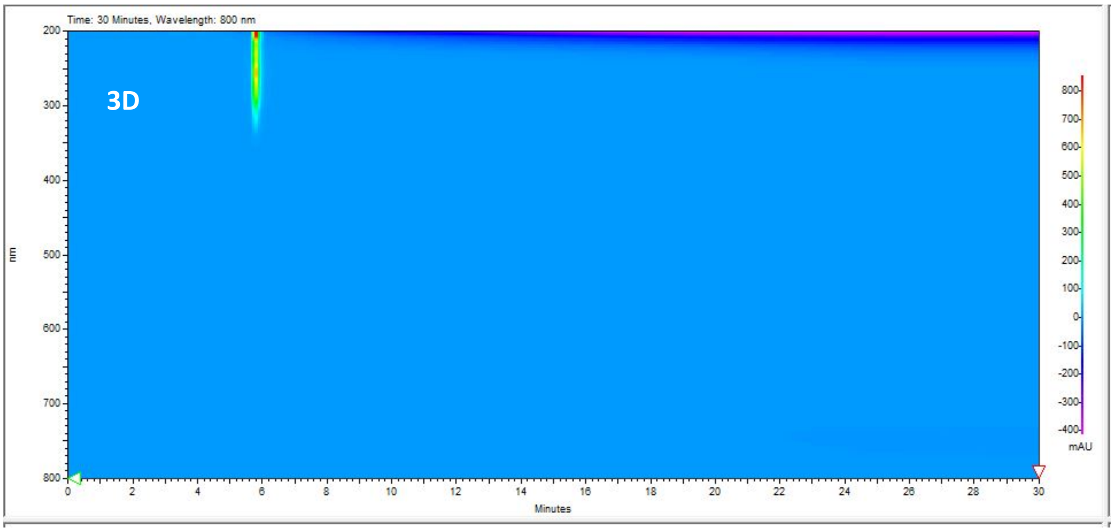

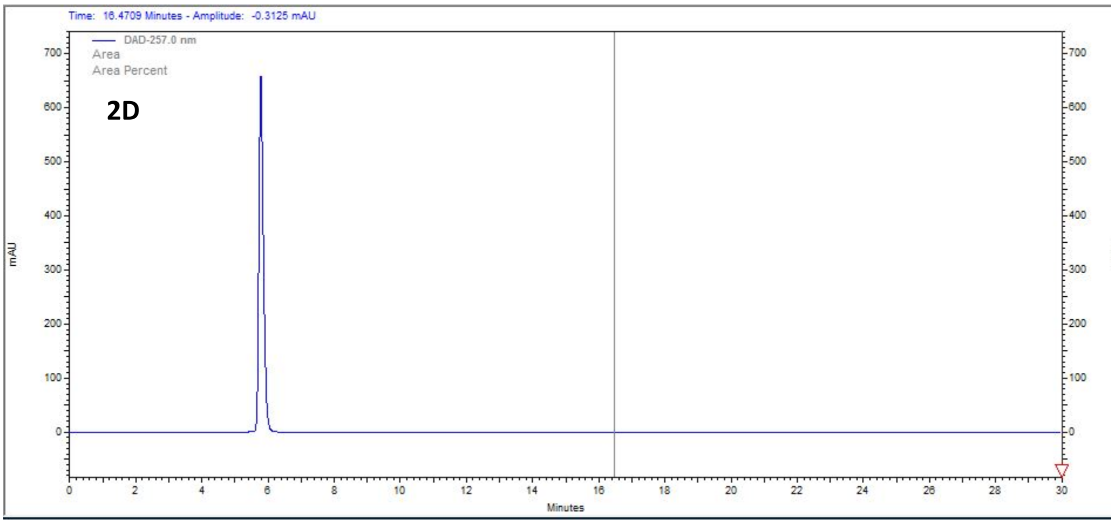

Figure S110. HPLC spectra (70-100% H_2O[0.1%TFA]:ACN gradient) of **pyroglutamyl aminopeptidase probe 4** (2D HPLC spectra absorbance measured at 280nm).

Phosphatase probe 5

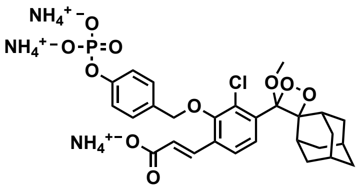

Phosphatase probe 5

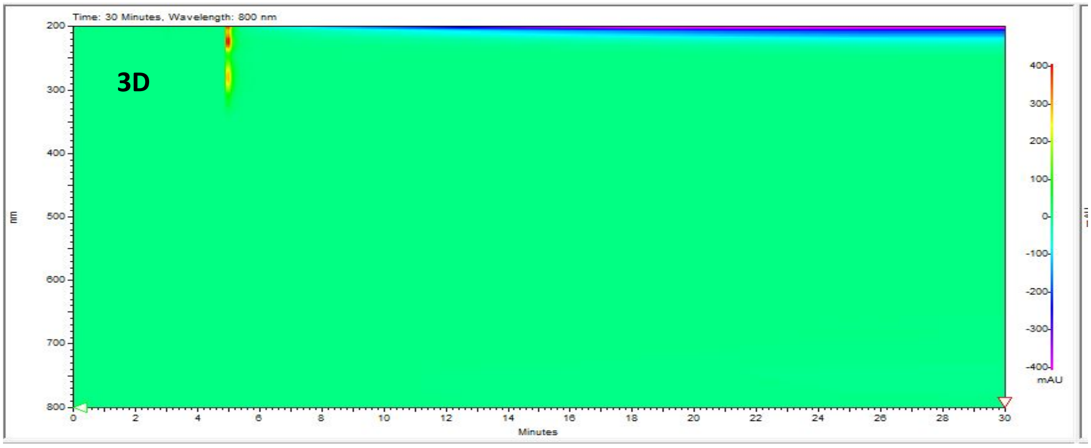

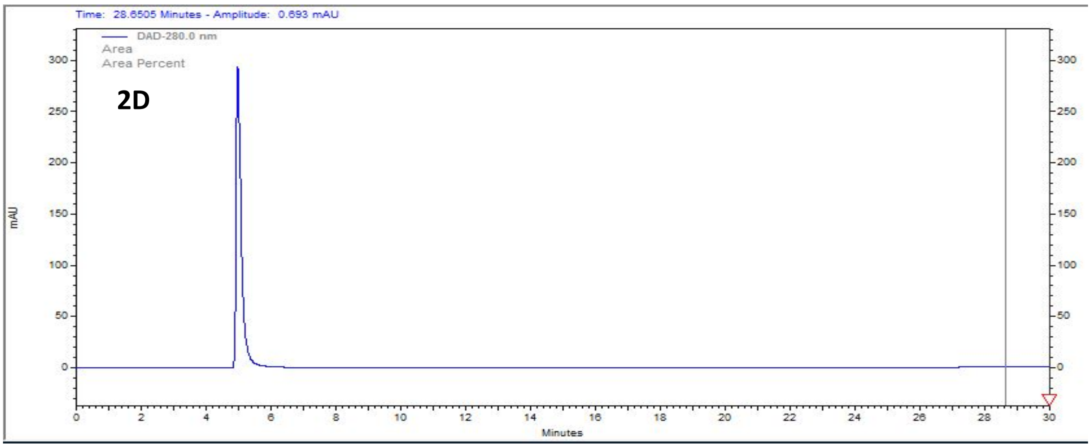

Figure S111. HPLC spectra (70-100% H_2O[0.1%TFA]:ACN gradient) of **phosphatase probe 5** (2D HPLC spectra absorbance measured at 280nm).

Leucine aminopeptidase probe 6

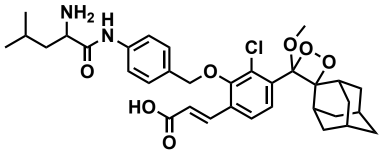

Leucine aminopeptidase probe 6

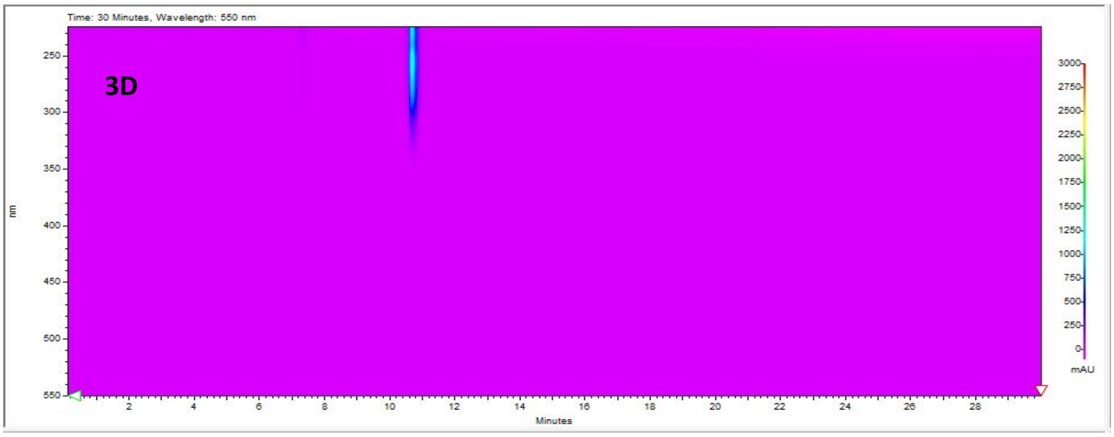

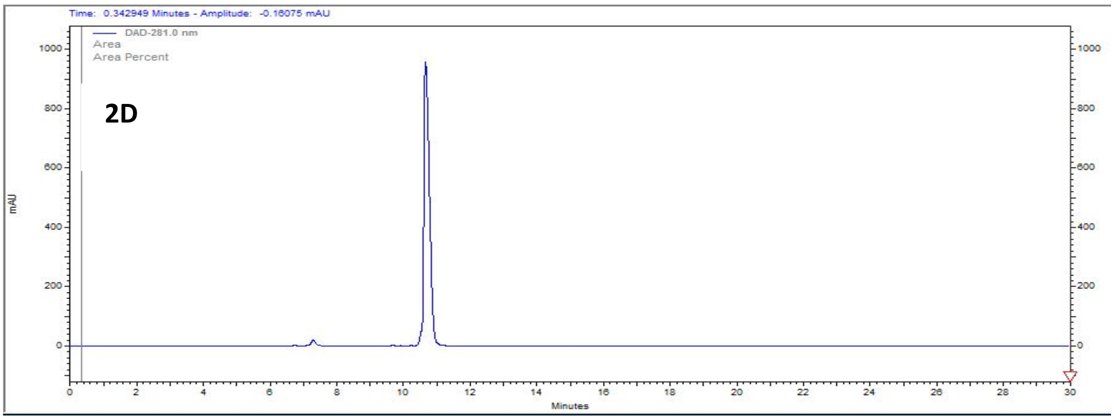

Figure S112. HPLC spectra (50-100% H_2O[0.1%TFA]:ACN gradient) of **leucine aminopeptidase probe 6** (2D HPLC spectra absorbance measured at 280nm).

*The minor peak in HPLC spectra belongs to the benzoate ester, the dioxetane's thermal decomposition product.

Compound 11

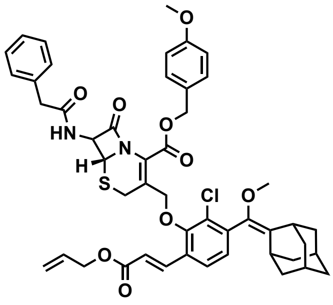

Compound 11

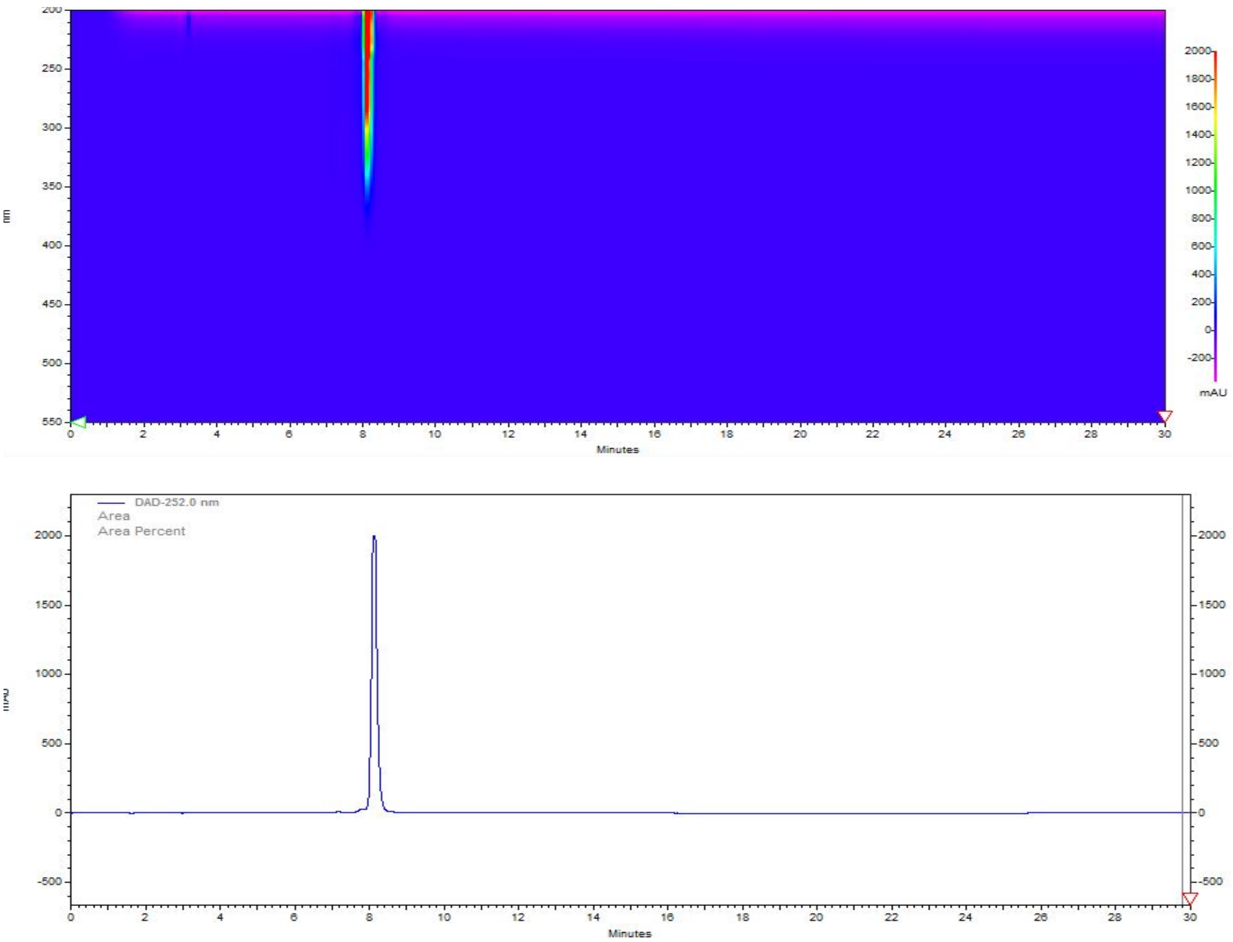

Figure S113. HPLC spectra (90-100% H_2O[0.1%TFA]:ACN gradient) of ***β*-lactamase probe 9** (2D HPLC spectra absorbance measured at 280nm).

β-Lactamase probe 9

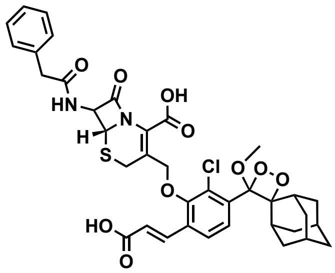

***β*-Lactamase probe 9**

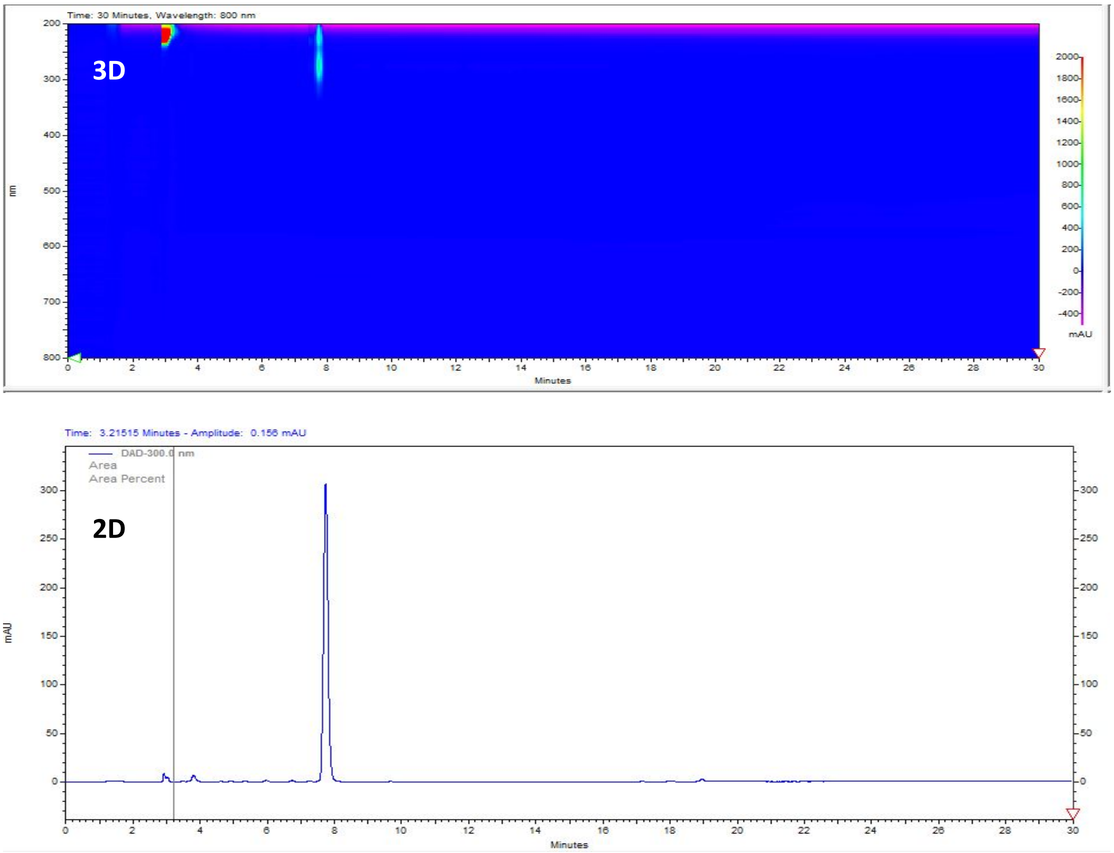

Figure S114. HPLC spectra (70-100% H_2O[0.1%TFA]:ACN gradient) of ***β*-lactamase probe 9** (2D HPLC spectra absorbance measured at 280nm).

Compound 13

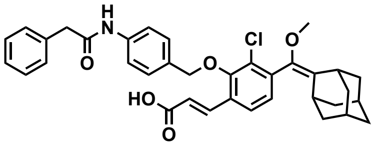

Compound 13

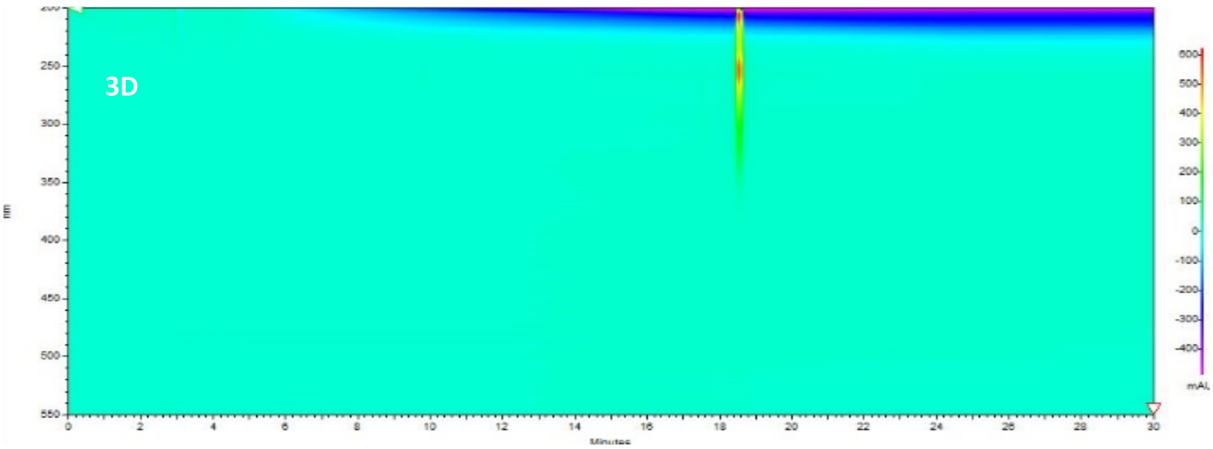

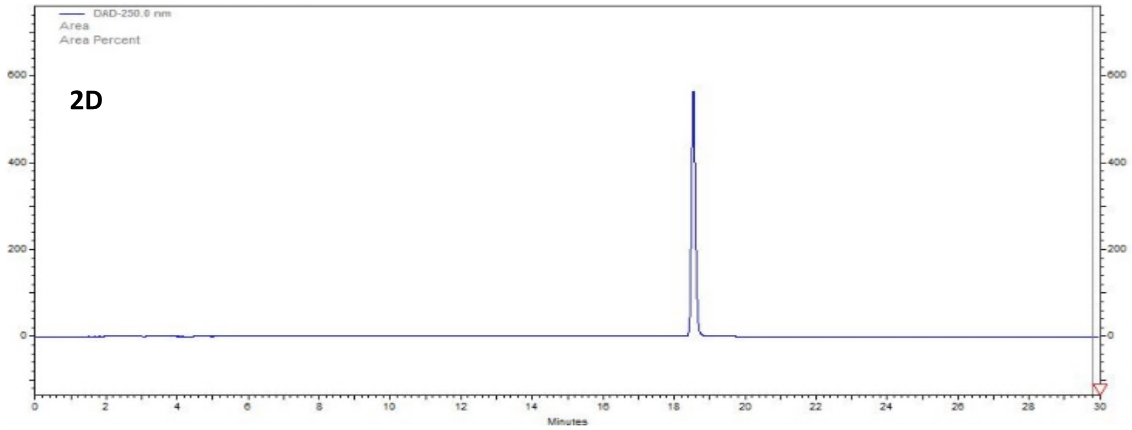

Figure S115. HPLC spectra (70-100% H_2O[0.1%TFA]:ACN gradient) of compound **13** (2D HPLC spectra absorbance measured at 280nm).

Penicillin-G amidase probe 10

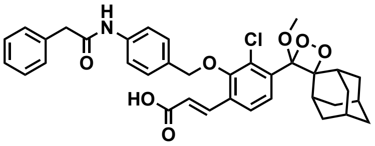

Penicillin-G amidase probe 10

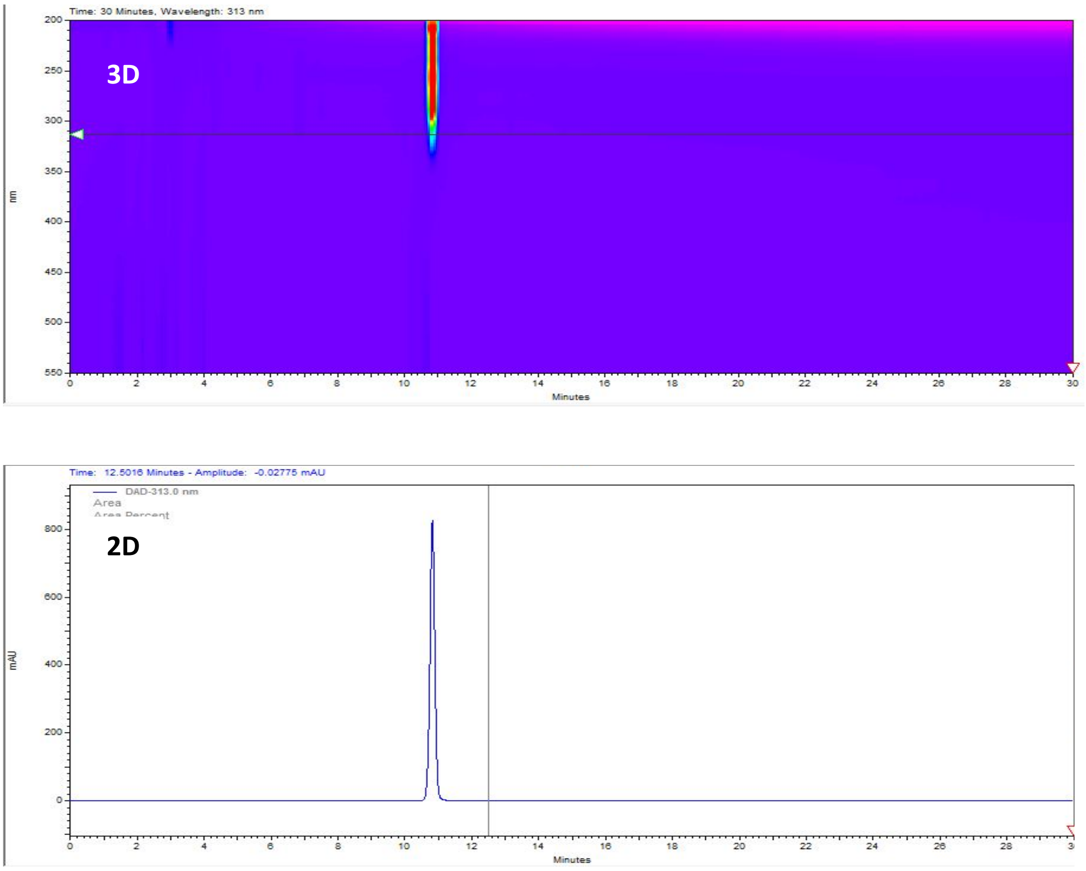

Figure S116. HPLC spectra (70-100% H_2O[0.1%TFA]:ACN gradient) of **penicillin-G amidase probe 10** (2D HPLC spectra absorbance measured at 280nm).

Compound 14

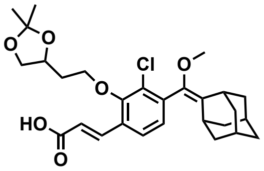

Compound 14

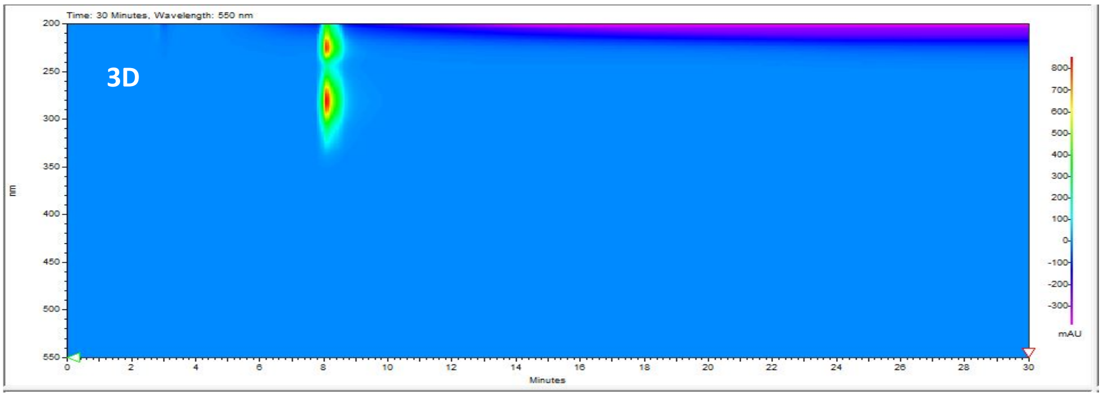

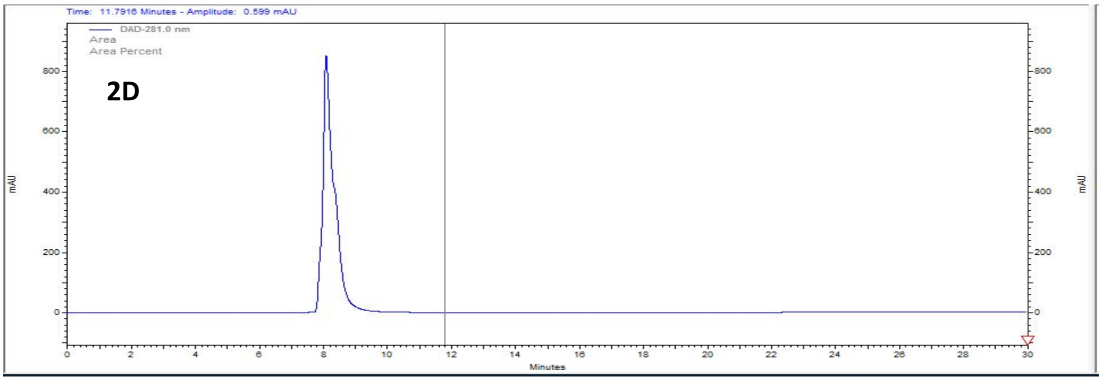

Figure S117. HPLC spectra (50-100% H_2O[0.1%TFA]:ACN gradient) of compound **14** (2D HPLC spectra absorbance measured at 280nm).

Periodate oxidative-cleavage probe 11

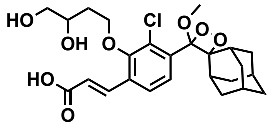

Periodate oxidative-cleavage probe 11

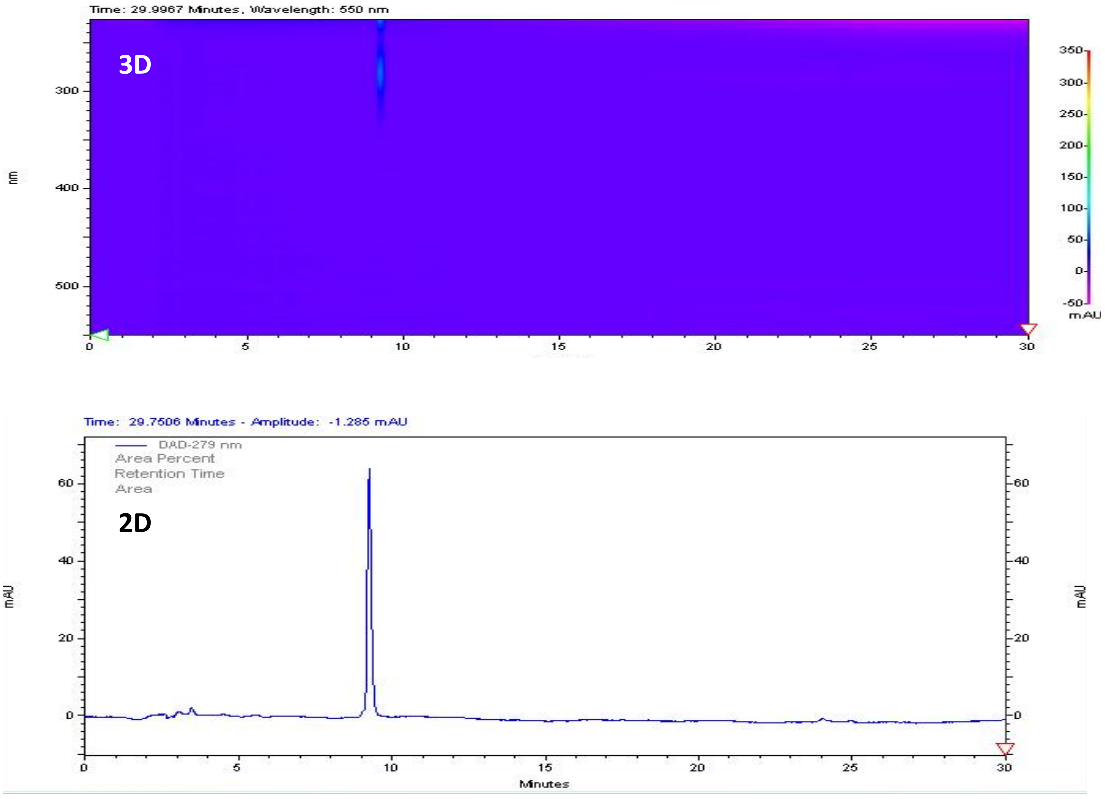

Figure S118. HPLC spectra (50-100% H_2O[0.1%TFA]:ACN gradient) of **periodate oxidative-cleavage probe 11** (2D HPLC spectra absorbance measured at 280nm).

N-acetyl hydrolase probe 12

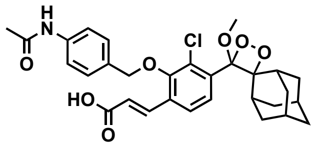

N-Acetyl hydrolase probe 12

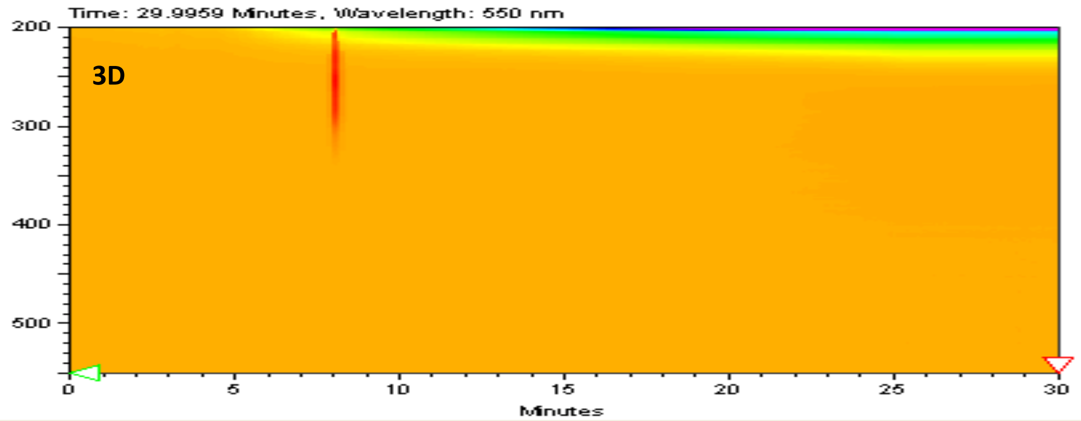

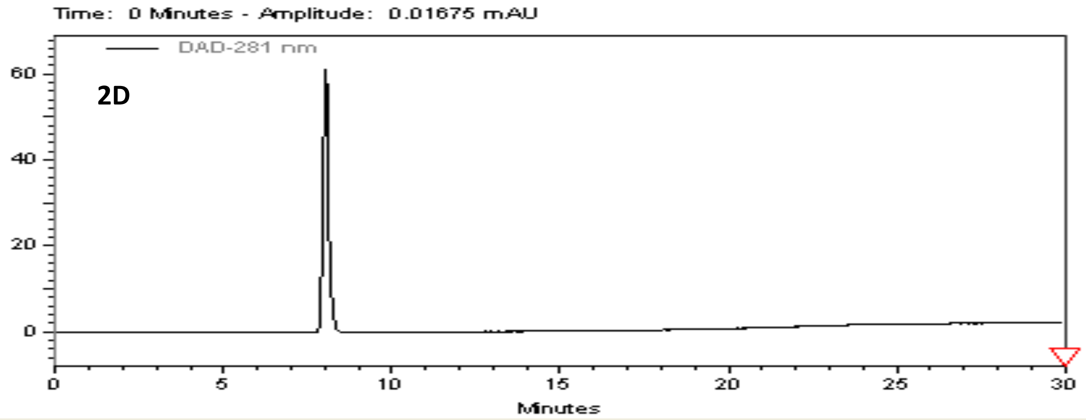

Figure S119. HPLC spectra (70-100% H_2O[0.1%TFA]:ACN gradient) of **N-acetyl hydrolase probe 12** (2D HPLC spectra absorbance measured at 280nm).

Compound 16

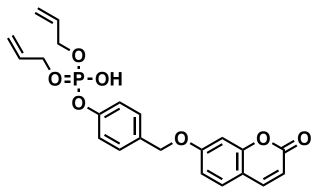

Compound 16

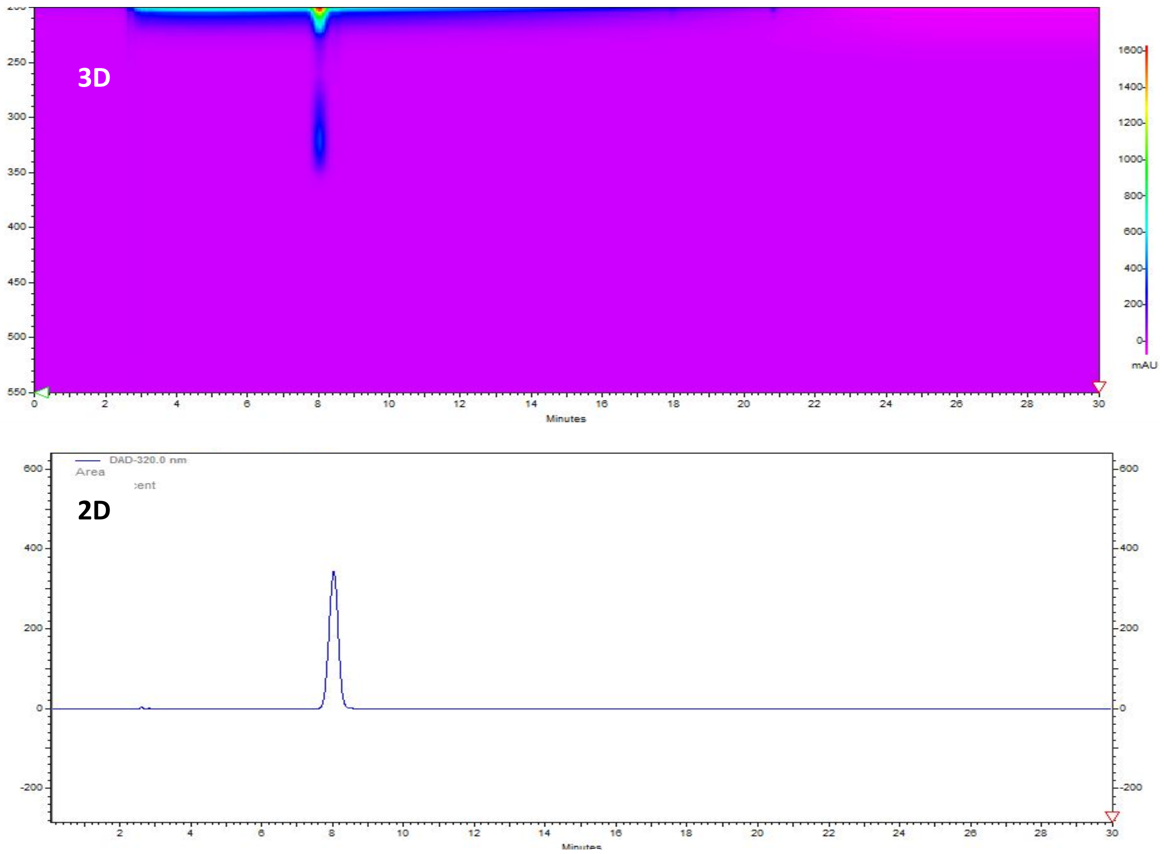

Figure S120. HPLC spectra (50-100% H_2O[0.1%TFA]:ACN gradient) of compound **16** (2D HPLC spectra absorbance measured at 320nm).

Compound 17

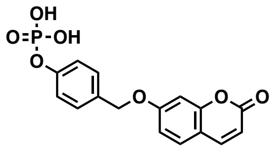

Compound 17

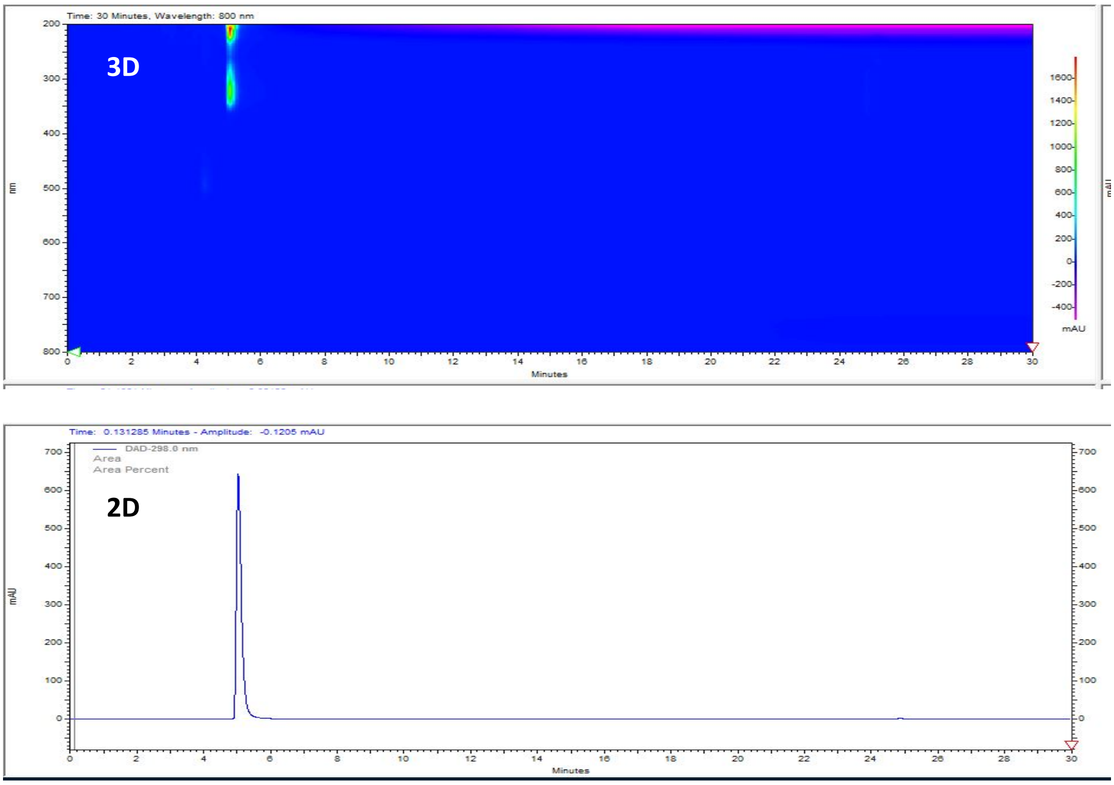

Figure S121. HPLC spectra (30-100% H_2O[0.1%TFA]:ACN gradient) of compound **17** (2D HPLC spectra absorbance measured at 280nm).

Compound 19

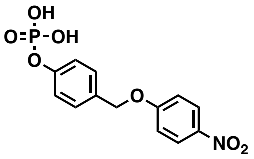

Compound 19

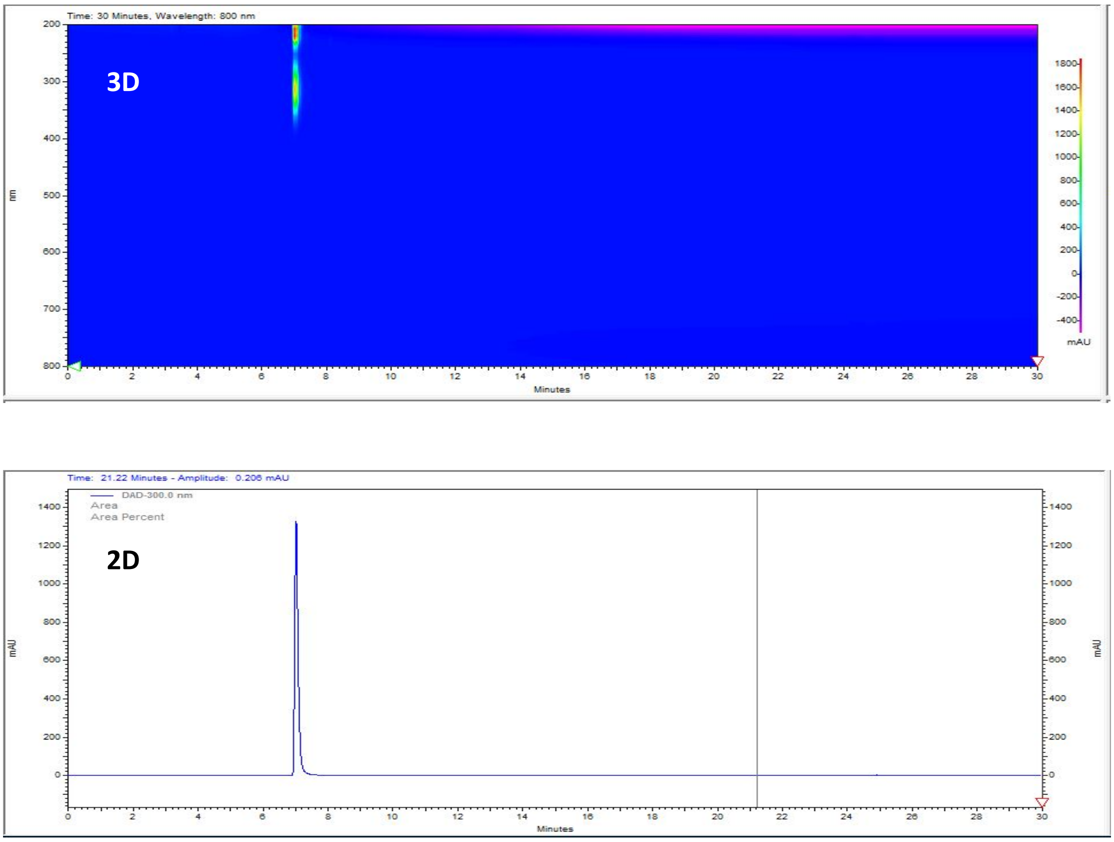

Figure S122. HPLC spectra (30-100% H_2O[0.1%TFA]:ACN gradient) of compound **19** (2D HPLC spectra absorbance measured at 280nm).

References

(1) Ryan, L. S.; Gerberich, J.; Cao, J.; An, W. W.; Jenkins, B. A.; Mason, R. P.; Lippert, A. R. Kinetics-Based Measurement of Hypoxia in Living Cells and Animals Using an Acetoxymethyl Ester Chemiluminescent Probe. *ACS Sens.* **2019**, *4* (5), 1391-1398.

(2) Son, S.; Won, M.; Green, O.; Hananya, N.; Sharma, A.; Jeon, Y.; Kwak, J. H.; Sessler, J. L.; Shabat, D.; Kim, J. S. Chemiluminescent Probe for the In Vitro and In Vivo Imaging of Cancers Over-Expressing NQO1. *Angew. Chem. Int. Ed. Engl.* **2019**, *58* (6), 1739-1743.

(3) Hananya, N.; Reid, J. P.; Green, O.; Sigman, M. S.; Shabat, D. Rapid chemiexcitation of phenoxy-dioxetane luminophores yields ultrasensitive chemiluminescence assays. *Chem. Sci.* **2019**, *10* (5), 1380-1385.

(4) Gnaim, S.; Scomparin, A.; Eldar-Boock, A.; Bauer, C. R.; Satchi-Fainaro, R.; Shabat, D. Light emission enhancement by supramolecular complexation of chemiluminescence probes designed for bioimaging. *Chem. Sci.* **2019**, *10* (10), 2945-2955.

(5) Zhang, H.; Yeung, K.; Robbins, J. S.; Pavlick, R. A.; Wu, M.; Liu, R.; Sen, A.; Phillips, S. T. Self-Powered Microscale Pumps Based on Analyte-Initiated Depolymerization Reactions. *Angew. Chem. Int. Ed.* **2012**, *51* (10), 2400-2404.

(6) Green, O.; Eilon, T.; Hananya, N.; Gutkin, S.; Bauer, C. R.; Shabat, D. Opening a Gateway for Chemiluminescence Cell Imaging: Distinctive Methodology for Design of Bright Chemiluminescent Dioxetane Probes. *ACS Cent. Sci.* **2017**, *3* (4), 349-358.

(7) Wang, B. Q.; Chen, Z. Z.; Cen, X. H.; Liang, Y. Q.; Tan, L. Y.; Liang, E.; Zheng, L.; Zheng, Y. J.; Zhan, Z. K.; Cheng, K. A highly selective and sensitive chemiluminescent probe for leucine aminopeptidase detection in vitro, in vivo and in human liver cancer tissue. *Chem. Sci.* **2022**, *13* (8), 2324-2330.

(8) Zhao, G. Y.; Miller, M. J.; Franzblau, S.; Wan, B. J.; Mollmann, U. Syntheses and studies of quinolone-cephalosporins as potential anti-tuberculosis agents. *Bioorg. Med. Chem. Lett.* **2006**, *16* (21), 5534-5537.

(9) Taber, D. F.; Xu, M.; Hartnett, J. C. Synthesis of the eight enantiomerically pure diastereomers of the 12-F-2-isoprostanes. *J. Am. Chem. Soc.* **2002**, *124* (44), 13121-13126.
